# Deciphering the genetic control of fruit texture in apple by multiple family-based analysis and genome-wide association

**DOI:** 10.1093/jxb/erx017

**Published:** 2017-02-24

**Authors:** Mario Di Guardo, Marco C.A.M. Bink, Walter Guerra, Thomas Letschka, Lidia Lozano, Nicola Busatto, Lara Poles, Alice Tadiello, Luca Bianco, Richard G.F. Visser, Eric van de Weg, Fabrizio Costa

**Affiliations:** 1 Fondazione Edmund Mach, via Mach 1, 38010 San Michele all’Adige, Trento,Italy; 2 Graduate School Experimental Plant Sciences, Wageningen University, PO Box 386, 6700 AJ Wageningen, The Netherlands; 3 Biometris, Wageningen University and Research Centre, Wageningen, The Netherlands; 4 Laimburg Research Centre for Agriculture and Forestry, via Laimburg 6, 39040 Ora (BZ),Italy; 5 Innovation Fruit Consortium (CIF), via Mach 1, 38010 San Michele all’Adige, Trento, Italy; 6 Wageningen UR Plant Breeding, Wageningen University and Research Centre, Droevendaalsesteeg 1, PO Box 386, 6700 AJ Wageningen, The Netherlands

**Keywords:** Apple, Bayesian statistics, fruit texture, genome-wide association study (GWAS), high-resolution phenotyping, pedigree-based analysis (PBA), RT–qPCR, SNP.

## Abstract

Fruit texture is a complex feature composed of mechanical and acoustic properties relying on the modifications occurring in the cell wall throughout fruit development and ripening. Apple is characterized by a large variation in fruit texture behavior that directly impacts both the consumer’s appreciation and post-harvest performance. To decipher the genetic control of fruit texture comprehensively, two complementing quantitative trait locus (QTL) mapping approaches were employed. The first was represented by a pedigree-based analysis (PBA) carried out on six full-sib pedigreed families, while the second was a genome-wide association study (GWAS) performed on a collection of 233 apple accessions. Both plant materials were genotyped with a 20K single nucleotide polymorphism (SNP) array and phenotyped with a sophisticated high-resolution texture analyzer. The overall QTL results indicated the fundamental role of chromosome 10 in controlling the mechanical properties, while chromosomes 2 and 14 were more associated with the acoustic response. The latter QTL, moreover, showed a consistent relationship between the QTL-estimated genotypes and the acoustic performance assessed among seedlings. The *in silico* annotation of these intervals revealed interesting candidate genes potentially involved in fruit texture regulation, as suggested by the gene expression profile. The joint integration of these approaches sheds light on the specific control of fruit texture, enabling important genetic information to assist in the selection of valuable fruit quality apple varieties.

## Highlight

A distinct set of QTLs related to mechanical and acoustic fruit texture features were identified in apple. Through a GWAS approach, the specific genetic control of these subtraits was elucidated.

## Introduction

Fruit ripening is an orchestra of physiological changes occurring to render fruits more attractive and palatable (Giovannoni, 2001). This important quality feature depends on the dismantling of the primary cell wall polysaccharide complex by a series of cell wall-modifying proteins (Brummell and Harpster, 2001; Brummell, 2006). This synergic enzymatic action leads to different types of fruit texture in apple, from soft and mealy to firm and crisp, suggesting that rather than a single trait, fruit texture can therefore be considered as a multiple feature, with distinct specific mechanical and acoustic properties (Szczesniak, 2002; Zdunek *et al.*, 2010; Costa *et al.*, 2011). In the last decades, the control of fruit texture has represented a major goal towards the improvement of shelf-life performance (Matas *et al.*, 2009). This aspect is of crucial importance, especially in the case of year-round fruit marketability and shipping overseas. The use of transgenic lines (Kramer and Redenbaugh, 1994) and whole-transcriptome platforms has in fact identified several genes involved in cell wall metabolism, such as those encoding expansin, pectin acetylesterase, xyloglucan endotransglycosylase, pectin methylesterase, pectate lyase, and polygalacturonase (Rose *et al.*, 2002, 2004; Marín-Rodríguez *et al.*, 2003; Eriksson, 2004; Brummell, 2006; Vicente *et al.*, 2007; Janssen *et al.*, 2008; Moore *et al.*, 2008; Soglio *et al.*, 2009; Costa *et al.*, 2010*a*; Jimènez-Bermudéz *et al*., 2002; Segonne *et al.*, 2014). In support of these studies, genome-wide mapping of quantitative trait loci (QTLs) controlling fruit firmness is also compelling, with the final purpose of identifying the most valuable molecular markers suitable for marker-assisted breeding programs. An exhaustive knowledge of the fruit texture genetic make-up is in fact essential to guide the selection of the most valuable ideotypes in breeding by design approaches (Peleman and Van Der Voort, 2003). In this regard, it is worth emphasizing that the majority of QTLs associated with fruit texture characteristics have been focused mainly on one measurement, fruit firmness, and usually restricted to one or a few bi-parental mapping populations (Harada *et al.*, 2000; King *et al.*, 2000; Liebhard *et al.*, 2003; Oraguzie *et al.*, 2004; Kenis *et al.*, 2008; Costa *et al.*, 2010*b*; Chagné *et al.*, 2014; Ben Sadok *et al.*, 2015). To overcome this constraint, an important effort was represented by the simultaneous analysis of multiple populations connected in a common pedigree scheme, namely pedigree-based analysis (PBA) (Bink *et al.*, 2008). This method has already been successfully employed to target QTLs in apple (Bink *et al.*, 2014; Allard *et al.*, 2016) as well as in cherry (Rosyara *et al.*, 2013) and peach (Fresnedo-Ramírez *et al.*, 2015, 2016). In addition to this, association mapping has also been widely employed as a complementary strategy to classical QTL mapping (Rafalski, 2010). Although this approach was initially employed in annual crops (Thornsberry *et al.*, 2001; Weber *et al.*, 2008; Stracke *et al.*, 2009) and forest trees (Neale and Savolainen, 2004; González-Martínez *et al.*, 2007, 2008; Eckert *et al.*, 2009; Neale and Kremer, 2011), it has also recently been exploited in fruit tree crops, such as grapevine (Cardoso *et al.*, 2012), peach (Micheletti *et al.*, 2015), and apple (Kumar *et al.*, 2013, 2015). Especially in the latter species, a major QTL for fruit firmness was observed on chromosome 10, which coincided with *MdPG1*, a gene known to encode polygalacturonase playing a pivotal role in the depolymerization of pectins (Sitrit and Bennett, 1998; Brummell and Harpster, 2001). These investigations, however, are characterized by a low phenotyping resolution, to date recognized as the major operational bottleneck limiting the power of genetic analysis (Cobb *et al.*, 2013).

To this end, the dissection of the fruit texture complexity was carried out with a texture analyzer, an instrument that has already demonstrated its reliability in dissecting the apple fruit texture into mechanical and acoustic signatures (Costa *et al.*, 2012). To make advances in the deciphering of the genetic control of fruit texture in apple, a double approach was employed. Initially six full-sib pedigreed families were investigated through a PBA approach to detect QTLs associated with mechanical and acoustic fruit texture features. These regions were further complemented and validated by a genome-wide association study (GWAS) performed on a large apple germplasm collection to exploit a much larger range of both genetic and phenotypic variations.

## Materials and methods

### Plant materials

In this study, two groups of plant materials were employed. The first was represented by a pedigree composed of 13 parental lines and six full-sib populations (for a total of 416 individuals). The scheme generated with PediMap (Voorrips *et al.*, 2012; Supplementary Fig. S1; Supplementary Table S1 at *JXB* online) shows the maternal and paternal descendants from founders to progeny. The second group was represented by a collection of 387 apple accessions (*Malus*×*domestica* species; Supplementary Table S2), retrieved from the general apple variety repository available at the Fondazione Edmund Mach (FEM). The germplasm collection and two full-sib progeny (i.e. ‘FjDe’, ‘Fuji’×’Delearly’; and ‘FjPL’, ‘Fuji’×‘Cripps Pink’) were planted at the experimental orchard of FEM, while the other four full-sib families (‘GDFj’, ‘Golden Delicious’×‘Fuji’; ‘GaPL’, ‘Gala’×‘Cripps Pink’; ‘GaPi’, ‘Gala’×‘Pinova’; and ‘FjPi’, ‘Fuji’×‘Pinova’) were chosen from the ongoing breeding program at the Laimburg Research Centre for Agriculture and Forestry. Within each group of plant materials (pedigreed full-sib families and germplasm), trees were at a full fruit-bearing stage at the time of the analysis. Moreover, while individuals from full-sib families were characterized by a single and original tree, the apple accessions included in the germplasm collection were represented by triplicates.

### Fruit harvesting and high-resolution texture phenotyping

Fruit, from both the pedigreed full-sib families and cultivar collection, were harvested at a commercial maturity stage according to typical fruit characteristics, such as starch degradation (selected at 7 on a scale of 1–10) and skin color. After harvest, fruit were stored for 2 months at 2 °C and 95% relative humidity. Prior to phenotyping, apples were maintained at 20 °C overnight.

**Fig. 1.**
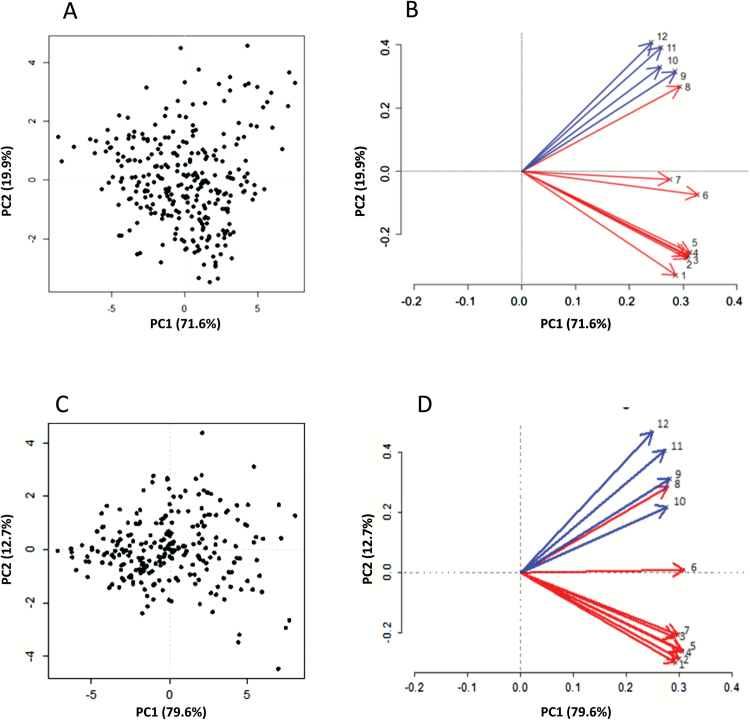
2D-PCA plots depicting the fruit texture variability assessed in the six pedigreed-families (A and B) and germplasm collection (C and D). For both plant materials, the sample distribution over the hyperspace defined by the first two PCs (A and C) and the loading projections (B and D) are illustrated. In the loading panels, red-colored arrows indicate the mechanical parameters, while acoustic parameters are shown with blue arrows. Each parameter is coded with a number as follows: 1, initial force; 2, maximum force; 3, final force; 4, mean force; 5, area; 6, force linear distance; 7, Young’s modulus; 8, number of force peaks; 9, maximum acoustic pressure; 10, mean acoustic pressure; 11, acoustic linear distance; 12, number of acoustic peaks.

The high-resolution phenotyping of fruit texture was carried out with a computer-controlled texture analyzer TAXT*plus* (StableMicroSystem, Godalming, UK), according to the protocol described by Costa *et al.* (2011, 2012). Since the texture analyzer was equipped with an AED (Acoustic Envelope Device), for each sample (flesh disc) a simultaneous profiling of the mechanical displacement and acoustic response was acquired. The combined profile was further processed with an ad hoc macro for the digital definition of 12 parameters, related to both mechanical (eight) and acoustic (four) texture properties (specified in Fig. 1). The phenotype was assessed for two consecutive experimental years and represented by BLUP (Best Linear Unbiased Prediction, ‘Ime4’ R package), which adjusted the mean by reducing the error variance.

### SNP genotyping

Genomic DNA was isolated from young leaves with the Qiagen DNeasy Plant Kit (Qiagen). DNA quantity and quality were measured with a Nanodrop ND-8000 (ThermoScientific, USA). The genotyping was carried out with the 20K Infinium array (Illumina; Bianco *et al.*, 2014; www.fruitbreedomics.com). The SNP data, initially processed with the GenomeStudio Data Analysis software, were finally elaborated with ASSIsT (Di Guardo *et al.*, 2015), a dedicated stand-alone pipeline to filter and re-edit SNP calls with a distorted segregation pattern due to the presence of null alleles (Pikunova *et al.*, 2013).

### Linkage mapping and SNP marker consensus order

The array of SNPs segregating within the six bi-parental pedigreed populations was used to create a bi-parental integrated map for each full-sib family, using the software JoinMap 4.1 (Van Ooijen, 2006). Markers were initially clustered in linkage groups with a minimum LOD value of 3.0 and further ordered with a recombination frequency of 0.45 and Haldane mapping function. The six genetic maps were finally merged into a consensus map using BioMarcator software v4.2 (Sosnowski *et al.*, 2012), through the implementation of the ConsMap module. The new reference and harmonized marker order (Supplementary Table S3) was exploited as a map input file for both PBA and GWAS analysis.

### QTL mapping by pedigree-based analysis

The identification and mapping of QTLs at the genome-wide scale was carried out with FlexQTL™ (Bink and van Eeuwijk, 2009; Bink *et al.*, 2014; www.wur.nl/en/show/flexqtl.htm). The Bayesian approach on which the software is based compares different models, considered as a random variable. The linear model is expressed as follows:

y=μ+Wa+e

where *y* is the observed phenotype, μ is the phenotypic mean, *W* is a matrix of a vector of regression on the QTL covariates (*a*), and *e* is the residual error of the model. The model operated by FlexQTL™ is based on independent assignment of QTL alleles to founders; therefore, the genotype of several individuals is *a priori* unknown. Since the joint posterior distribution for the number of QTLs cannot be analytically computed due to the high number of genotype combinations, a Markov chain Monte Carlo (MCMC) simulation is used. The effective chain size (ECS; Sorensen and Gianola, 2002), used to assess the sensitivity of the posterior inference, was considered statistically significant with values >100 for phenotypic mean (μ), QTL explained variance (σ ^2^_a_), QTL residual variance (σ ^2^_e_), and number of expected QTLs on the *a priori* distribution (*N*_QTL_), respectively. In the analysis carried out here, 500 000 iterations were performed and a thinning of 500 was applied to reduce computation storage. For each run, the convergence quality was represented by a trace plot for visual inspection (Supplementary Fig. S2). For each chromosome, the number of QTL(s) was inferred comparing models with an increased number of QTLs. In FlexQTL™, the most likely number of QTLs was inferred based on Bayes factors (BFs), which represent the ratio of the marginal likelihood under one model compared with the marginal likelihood under a second model. A 2ln(BF) ≥2, 5, or 10 indicates positive, strong, or decisive evidence of a model, respectively. Moreover, each QTL is considered as bi-allelic, with three possible genotypic conformations: ‘*QQ*’, ‘*Qq*’, and ‘*qq*’. The analysis was carried out with an additive genetic model, assigning to ‘*QQ*’ and ‘*qq*’ a value of 1 and –1, respectively, while ‘*Qq*’ was equal to 0. The QTL genotype of each individual included in the pedigree is *a priori* unknown and the alleles are assigned to founders tracing their transmission to offspring. To reduce computation time and increase marker informativeness, the initial set of 10 695 markers was finally converted into 1045 haploblocks with PediHaplotyper (Voorrips *et al.*, 2016)

### Linkage disequilibrium analysis

The SNP markers exploited on the germplasm collection were initially employed to estimate the linkage disequilibrium (LD) decay. From the 20K SNPs, 7378 markers were actually used for LD analysis, excluding rare alleles with a minor allele frequency (MAF) <0.05 and those showing an incongruent physical/genetic position. The pair-wise *r*^2^ between SNP markers was calculated with Plink (Purcell *et al.*, 2007). For both a chromosome-wise and genome-wide scale, the LD decay was depicted by plotting the pair-wise *r*^2^ value against the corresponding physical distance on the genome (bp). The estimation of the LD decay distance was defined by crossing the *r*^2^ baseline (based on the 95th percentile of the marker distribution, according to Breseghello and Sorells, 2006) and the locally weighted polynomial regression-based fitting curve (LOESS) fitted to the plot (‘stats’ R package). For each chromosome, the LD level was also depicted by partitioning the chromosomal regions into segments of strong LD with Haploview (Barrett *et al.*, 2005).

### Population structure and genome-wide association mapping

The level of genetic stratification was assessed with STRUCTURE v2.3.1 (Pritchard *et al.*, 2000). To this end, 17 simple sequence repeats (SSRs; Supplementary Table S4) were amplified according to the protocol reported in Di Guardo *et al.* (2013). The SSR genetic data were further used to compute the posterior probability [Pr(X|K)], given a specific number of group K (ranging from K=2 to K=8). The computation was carried out performing five independent runs of 1 000 000 burn-in generations and considering the admixture model. The most probable number of populations was identified with STRUCTURE HARVESTER (Earl and vonHoldt, 2012), and the final population structure matrix (Q) was further implemented as a covariate in GWAS analysis.

The marker–trait association analysis was carried out with TASSEL v3 software (Bradbury *et al.*, 2007; http://www.maizegenetics.net). The significance of the association was tested implementing both the general linear model (GLM) and the mixed linear model (MLM). The GLM (Pritchard *et al.*, 2000) was computed correcting for population structure. The MLM (Zhang *et al.* 2010), instead, included both fixed and random effects, allowing the incorporation of genetic relationship as follows:

y=Xβ+Zu+e

where *y* represents the phenotype (vector of observation), β is an unknown vector containing fixed effects (marker and Q), *u* is an unknown vector of random additive genetic effects, *X* and *Z* are the known designed matrices, and *e* is the vector of random residuals. The MLM considered both Q (population structure) and K (kinship) matrixes as covariates for population and parental relationship correction (false positive). Significant associations were selected according to a *P*-value ≤0.05, after false discovery rate (FDR) correction for multiple comparison according to the procedure of Benjamini and Hochberg (1995) using the ‘stats’ R package. For each trait considered in the association, the choice of the model was suggested by the visual inspection of the Q–Q plot, obtained with the ‘qqman’ R package.

### Gene expression analysis by RT–qPCR

To assess whether a change in gene expression corresponds to a different QTL estimated genotype, the transcription profiling of *MdACO1* and *MdPG1* was assessed at harvest and after 2 months of cold storage. To achieve this goal, the three parental lines (‘Delearly’, ‘Fuji’, and ‘Cripps Pink’) together with four seedlings for ‘FjDe’ (45, 125, 10, and 14) and ‘FjPL’ (23, 25, 35, and 68) were selected. Fruit mesocarp was cut, frozen in liquid nitrogen, ground into a fine powder, and stored at –80°C until processing. RNA extraction, quantification, and reverse transcription–quantitative PCR (RT–qPCR) were carried out according to the methods described in Busatto *et al.* (2015). The final Ct is represented by the average of three independent normalized expression values for each sample, and an actin gene (*MdACT*) was employed as housekeeping gene (Di Guardo *et al.*, 2013). For each gene, a pair of discriminant and specific primers was designed (Supplementary Table S5), using Primer3 (http://primer3.ut.ee) and Primique (http://cgi-www.daimi.au.dk/cgi-chili/primique/front.py).

## Results and Discussion

### High-resolution phenotyping of fruit texture behavior in apple

The apple fruit texture was assessed with a novel and sophisticated texture analyzer (Costa *et al.*, 2011, 2012). The overall phenotypic fruit texture variability was initially represented by a principal component analysis (PCA) plot (Fig. 1). For both groups of plant materials, the fruit texture parameters were similarly oriented, with a consistent incidence of the two principal components (PCs) chosen to define the PC hyperspace. Comparing the two plots, PC1 explained 71.6% of the total phenotypic variance in the pedigreed full-sib families (Fig. 1A, B) and 79.6% in the germplasm collection (Fig. 1C, D), while PC2 accounted for 19.9% and 12.7%, respectively. Individuals were distributed following the loadings’ projection (Fig. 1B, D) represented by the 12 texture parameters, which clearly discriminated the two signatures. In both scenarios, the variables were in fact distinctly oriented towards two PCA quadrants. The mechanical parameters (highlighted by the numerical code from 1 to 8) were mostly plotted in the PC1 positive/PC2 negative quadrant, while the acoustic parameters (9–12) were projected on the quadrant defined by positive values for both PCs, besides the number of force peaks (8). Although this index is considered a mechanical parameter, it is correlated more with the group acoustic indices, justified by the mechanism behind the generation of the acoustic response and pressure progression (Vincent, 1998).

### Fruit texture QTL discovery through PBA

**Fig. 2.**
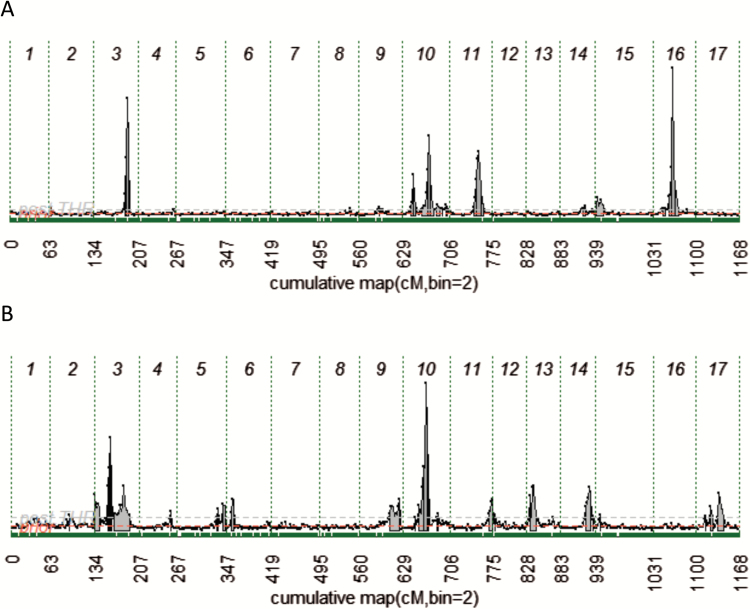
QTL probability pattern for the maximum force (A) and number of acoustic peaks (B). The gray area below each QTL profile indicates the 90% credible region associated with each estimated mean. For both panels, chromosomes are delimited by dashed vertical lines and numbered from 1 to 17. Below each panel the cumulative distance of the consensus genetic map is reported. (This figure is available in colour at *JXB* online.)

Each parameter obtained from the phenotypic dissection of fruit texture was finally exploited in marker–trait association studies. In the attempt to map the QTLs related to this feature, the Bayesian approach was initially employed. QTLs were identified and mapped on 13 chromosomes, on which the posterior QTL intensity exceeded the posterior probability threshold [2ln(BF) >2; Table 1]. The overall genome-wide QTL overview (Supplementary Fig. S3) distinguished specific probability profiles for the two groups of texture-related parameters, acoustic and mechanical. For simplicity, the QTL differences are highlighted in Fig. 2, comparing the profiles of the maximum force (mechanical) with the number of acoustic peaks (acoustic). Chromosomes 3 and 10 showed QTLs commonly shared by both features (Fig. 2; Supplementary Fig. S3). For the maximum force, in particular, the QTL mapped on chromosome 3 (Fig. 2A) is located on a single genomic region [2ln(BF)_1/0_=13.6], with a mode at 55 cM and an allelic effect (AEt1) of 1.54 (Table 1). This position was also similar to the rest of the mechanical parameters, spanning from 55 cM to 57 cM (Supplementary Fig. S3; Table 1). The only difference was observed for the number of force peaks, which showed the QTL at 10 cM. This observation, however, additionally confirms the association of this parameter with the group of acoustic parameters. In the case of the acoustic peaks (Fig. 2B), two QTLs were instead suggested [2ln(BF)_2/1_=2.7], with a mode at 3 cM and 28 cM and an AEt1 of 10.55 and 9.39, respectively. Among the acoustic subtraits, the QTL on chromosome 3 was also identified for the acoustic linear distance, but at 28 cM. On chromosome 10, a single QTL associated with the number of acoustic peaks (Fig. 2B) was shown [2ln(BF)_1/0_=10.6] and located at 40 cM with an AEt1 of 18.25. This position was also similar to the rest of the acoustic parameters (at 40 cM and 42 cM), beside the mean acoustic pressure that showed the QTL peak at 35 cM, as was also observed for the number of force peaks (Table 1). For the maximum force, two QTLs were instead observed on this chromosome [2ln(BF)_2/1_=4.2]. The first was located at 20 cM, with an AEt1 of 0.91, while the second was mapped at 45 cM with an AEt1 of 1.92. These two regions were also consistent across the mechanical parameters (spanning between 19 cM and 20 cM for the first QTL and 44–46 cM for the second), with two exceptions. The force linear distance in fact showed only one QTL (with a low probability and effect) at 49 cM, while for the Young’s modulus (or elastic modulus) no QTL was observed (Table 1; Supplementary Fig. S3). Beside these, other QTLs were identified with a more specific pattern. The two major genomic intervals showing QTLs associated with mechanical parameters were mapped on chromosomes 11 and 16 (Fig. 2; Supplementary Fig. S3). The QTLs positioned on chromosome 11, across the several mechanical parameters, were located between 41 cM and 49 cM (Table 1). On this chromosome it is also interesting to note the QTL positioned at 14 cM [2ln(BF)=7.9 and AEt1 of 0.11] and related to the Young’s modulus. The different and original positioning of this QTL within the class of mechanical parameters can be due to the fact that the Young’s modulus depends on the elasticity of the sample (the ratio between stress and strain) rather than fruit firmness. The particular behavior observed for the Young’s modulus, besides its projection over the PCA plot (Fig. 1B, D), is moreover validated by the QTL profile detected on chromosome 16. As reported for chromosome 11, this QTL is also specifically associated with the mechanical parameters (Supplementary Fig. S3), with a mode located at ~32 cM, with the exception of the Young’s modulus, where this QTL was not detected (similarly to chromosome 10).

**Table 1.** For each trait assessed, the variance (Var), the mean (Mean), and the linkage group (LG) on which the QTL is identified are reported For each QTL, the interval, the length, the mode (in cM), the 2ln Bayes factor (BF) for the presence of one or two QTLs (1/0 and 2/1, respectively), the probability (Prob), the additive effect size (AEt1), the additive variance (AVt1), and the weighted additive variance (wAVt1) are also shown. Each parameter refers to a haploblock, specified in the last column

Trait	Var	Mean	LG	Interval	Length	Mode	2lnBF (1/0)	2lnBF (2/1)	Prob	AEt1	AVt1	wAVt1	Haplotype
Number of acoustic peaks	678.4	1.23	3	1–19	18	3	–	2.7	0.312	10.556	55.691	17.389	fp03_03
			3	21–60	39	28	6.2		0.986	9.393	42.862	42.26	fp03_28
			9	37–69	32	61	2.9		0.527	10.272	51.382	27.084	fp09_61
			10	21–64	43	40	10.6		1.102	18.251	142.511	157.106	fp10_40
			13	7–29	22	13	2.6		0.334	10.361	52.182	17.446	fp13_13
			14	39–56	17	48	2.8		0.466	16.484	134.274	62.553	fp14_47
Acoustic linear distance	1 857 766.77	79.35	1	23–63	40	41	3.9		0.756	618.223	185782.329	140368.871	fp01_36
			3	1–61	60	28	5.2		1.145	484.318	111028.387	127177.97	fp03_28
			10	27–45	18	40	29.4		1.025	1254.328	641967.082	658178.372	fp10_40
Mean acoustic pressure	2.12	0.1	1	23–63	40	36	10.7		1.305	0.996	0.455	0.593	fp01_36
			10	22–44	22	35	29.5		1.036	1.428	0.911	0.944	fp10_35
Maximum acoustic pressure	8.37	0.22	1	21–58	37	33	5.9		0.797	1.86	1.53	1.219	fp01_33
			6	1–34	33	10	2.9		0.621	1.183	0.682	0.423	fp06_10
			10	31–45	14	42	29.6		1.016	2.93	3.459	3.515	fp10_42
			11	51–70	19	67	2.7		0.51	2.057	2.015	1.028	fp11_67
Number of force peaks	0.08	0.02	1	17–63	46	35	7.5		1.07	0.159	0.012	0.012	fp01_35
			3	1–20	19	10	4.6		0.704	0.116	0.007	0.005	fp03_10
			5	62–74	12	71	2.5		0.424	0.167	0.012	0.005	fp05_71
			8	21–65	44	51	3.9		0.705	0.081	0.003	0.002	fp08_51
			9	36–68	32	57	2.2		0.42	0.095	0.004	0.002	fp09_57
			10	25–45	20	35	29.5		1.029	0.271	0.03	0.031	fp10_35
Young’s modulus	0.05	0.02	1	11–43	32	26	4.5		0.628	0.065	0.002	0.001	fp01_26
			3	43–61	18	57	15.7		0.983	0.103	0.005	0.005	fp03_57
			4	1–32	31	8	3.4		0.324	0.095	0.004	0.001	fp04_07
			8	43–60	17	51	13.3		1.009	0.102	0.005	0.005	fp08_51
			11	1–32	31	14	7.9		1.079	0.118	0.007	0.007	fp11_15
Force linear distance	26.85	0.4	3	45–59	14	56	29.2		0.993	2.637	2.91	2.889	fp03_56
			8	32–54	22	50	6.2		0.886	1.726	1.451	1.285	fp08_50
			10	31–77	46	49	4.1		0.85	1.986	1.872	1.591	fp10_45
			11	34–59	25	41	12.6		1.008	2.696	3.614	3.643	fp11_41
			16	1–44	43	32	29.1	2.7	1.294	2.67	3.089	3.998	fp16_32
			16	60–69	9	65	–		0.086	1.44	1.025	0.088	fp16_65
Area	52 001.19	17.2	3	47–58	11	56	29.6		0.996	114.799	5948.646	5924.708	fp03_56
			10	10–30	20	19	–	4.3	0.421	67.952	2289.389	962.741	fp10_19
			10	32–77	45	45	10.1		1.276	125.237	7219	9209.01	fp10_45
			11	37–57	20	49	16		1.014	135.67	9043.087	9170.454	fp11_49
			16	13–45	32	32	29.1	2.7	1.28	167.25	11 316.163	14 481.046	fp16_32
			16	51–68	17	62	–		0.099	55.529	1540.738	151.904	fp16_62
Mean force	7.73	0.2	3	50–58	8	56	29.6		0.978	1.389	0.872	0.853	fp03_56
			10	12–26	14	19	–	4.2	0.399	0.812	0.325	0.13	fp10_19
			10	28–77	49	45	10.9		1.279	1.513	1.072	1.37	fp10_45
			11	34–60	26	47	13.7		1.04	1.664	1.369	1.424	fp11_47
			16	12–43	31	32	28.9	3.1	1.34	1.916	1.565	2.098	fp16_32
			16	56–69	13	69	–		0.072	0.671	0.225	0.016	fp16_68
Final force	9.77	0.21	3	38–60	22	55	15.7		1.024	1.282	0.784	0.803	fp03_56
			10	11–26	15	19	–	5.5	0.629	1.02	0.513	0.323	fp10_19
			10	28–53	25	44	11.1		1.024	2.295	2.279	2.334	fp10_43
			11	35–58	23	45	29.8		1.03	2.012	1.899	1.957	fp11_45
			16	13–59	46	32	28.4	4.2	1.53	2.271	2.245	3.436	fp16_32
			16	61–69	8	64	–		0.054	0.796	0.316	0.017	fp16_64
Maximum force	10.95	0.22	3	41–63	22	55	13.6		1.02	1.543	1.096	1.118	fp03_56
			10	10–29	19	20	–	4.2	0.381	0.914	0.415	0.158	fp10_20
			10	31–77	46	45	10.5		1.207	1.921	1.677	2.024	fp10_45
			11	36–66	30	47	15.9		1.055	2.007	1.963	2.072	fp11_47
			16	10–44	34	32	28.8		1.381	2.434	2.464	3.403	fp16_32
Initial force	7.1	0.17	3	48–59	11	56	29.5		0.977	1.325	0.802	0.783	fp03_56
			10	14–26	12	20	–	3.3	0.155	0.753	0.277	0.043	fp10_20
			10	30–77	47	46	7.4		1.337	1.167	0.678	0.906	fp10_46
			11	38–62	24	49	12.3		1.028	1.552	1.196	1.23	fp11_49
			15	1–19	18	4	2.9		0.545	1.073	0.561	0.306	fp15_04
			16	10–42	32	31	29.4		1.17	1.9	1.463	1.712	fp16_31

**Fig. 3.**
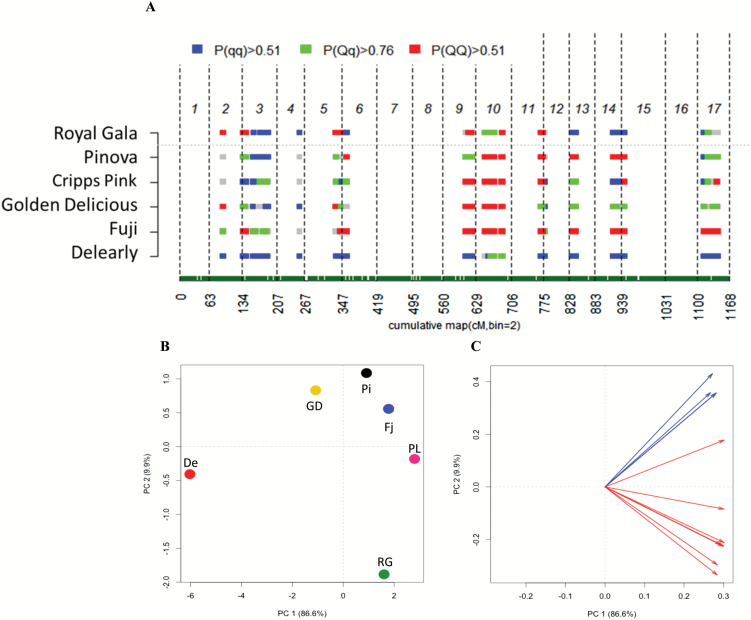
Posterior estimate of the QTL genotype probabilities computed for the number of acoustic peaks for each of the six parental cultivars (A). Each chromosome is indicated with numbers on the top, while on the bottom the cumulative genetic distance is reported. Each row represents a cultivar, named on the left side. Blue, green, and red colored bars indicate the ‘*QQ*’, ‘*Qq*’, and ‘*qq*’ QTL estimated genotype, respectively. In (B) the 2D-PCA plot showing the distribution of the six parental cultivars on the basis of their textural performance is reported. Each cultivar is coded as follows: De, ‘Delearly’; RG, ‘Gala’; GD, ‘Golden Delicious’; PL, ‘Cripps Pink’; Pi, ‘Pinova’; and Fj, ‘Fuji’; In (C), the variable projection is shown. Mechanical and acoustic parameters are depicted with red and blue colored arrows, respectively, according to Fig. 1.

**Fig. 4.**
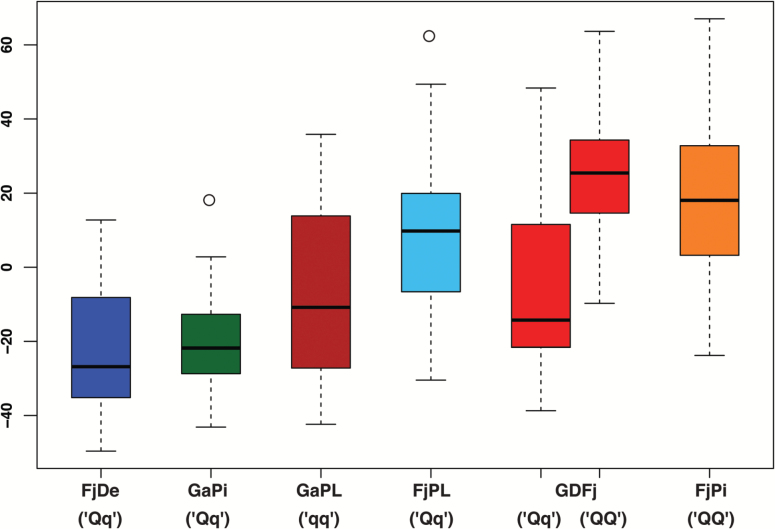
Box plot indicating the phenotype performance of the number of acoustic peaks for the six full-sib families. For each population, the estimated genotype for the QTL mapped on chromosome 14 is also indicated. Each box depicts the upper and lower quantile, with the median being represented by a horizontal solid line. Outliers are pointed by dots.

In the second subset of QTLs (related to the acoustic parameters), several genomic regions located on chromosome 1 and associated with mean and maximum acoustic pressure, number of force peaks, as well as the Young’s modulus were identified. Regarding force peaks, other QTLs were moreover mapped on chromosomes 5, 8, and 9. This latter chromosome, together with 13 and 14, was also associated with the number of acoustic peaks (Fig. 2B; Table 1; Supplementary Fig. S3). The simultaneous presence of QTLs detected on chromosome 9 for both number of force and acoustic peaks strengthens the relationship between these two parameters. Especially for the number of acoustic peaks, chromosome 9 and 14 were found to be the most important, showing an allelic effect of 10.27 and 16.48, respectively (Table 1). In particular, the QTL on chromosome 14 is characterized by an estimated genotype (Fig. 3A) consistent with the acoustic performance (assessed as the number of acoustic peaks) of the six parental cultivars (Fig. 3B, C). Among the group, ‘Pinova’ and ‘Fuji’ were distinguished by the highest acoustic response, as depicted in the 2D-PCA plot (Fig. 3B) and loading projection (Fig. 3C). The superior crispness performance of these two apple cultivars is also confirmed by the homozygous state of the positive estimated QTL allele (‘*QQ*’). In contrast, cultivars with a mealy texture, such as ‘Delearly’ and ‘Royal Gala’, are plotted on the other extreme of the 2D-PCA plot, showing a ‘*qq*’ genotype for this QTL. The effect of the estimated allele for the QTL on chromosome 14 was further investigated on the six progeny (Fig. 4). FlexQTL™ estimated a ‘*QQ*’ genotype for ‘Fuji’ and ‘Pinova’, a heterozygous ‘*Qq*’ genotype only for ‘Golden Delicious’, and a ‘*qq*’ genotype for the other three varieties (‘Delearly’, ‘Royal Gala’, and ‘Cripps Pink’). Taking into account that the ‘*Q*’ allele is known to increase the phenotypic performance, it is worth noting that only the seedlings of ‘FjPi’ and half of the progeny of ‘GDFj’ (with a ‘*QQ*’ genotype) were distinguished by the highest acoustic response (Fig. 4), underlying the role of this QTL in the control of the acoustic properties in apple.

### Analysis of the linkage disequilibrium in domesticated apple

**Fig. 5.**
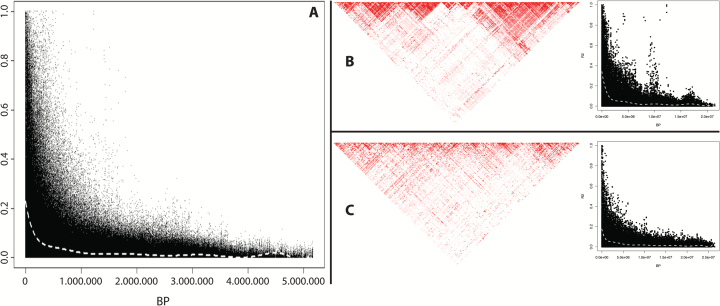
Linkage disequilibrium (LD) decay plot showing the *r*^2^ value between all possible pair-wise marker combinations against their physical distance over the genome. The LD plot is shown at the genome-wide level (A) as well as for chromosomes 16 (B) and 17 (C). For these two chromosomes, the pattern of LD was also depicted with Haploview heatmaps. For the LD decay plot, the white dashed line indicates the LOESS fitting curve.

The level of LD was determined to verify the genetic associations between loci and to scan the LD decay over each chromosome. To this end, from the total set of 10 695 selected SNP markers, 7378 were effectively employed in the computation, after subsequent filtering steps. From the initial data set, besides SNPs with a MAF <0.05, markers showing an inconsistent position between the physical location on the genome and the consensus genetic map were also excluded (Supplementary Fig. S4). In the germplasm collection investigated here (and represented by 387 apple accessions), the LD decay was estimated to extend for an average up to *r*^2^=0.19 at the genome-wide level (Fig. 5A) corresponding to ~400 kb, and spanning from a maximum of *r*^2^=0.28 for chromosome 16 (Fig. 5B) to a minimum of *r*^2^=0.13 for chromosome 17 (Fig. 5C). In parallel, the presence of distinct LD blocks over the genome was illustrated with an LD heatmap (Supplementary Fig. S5), highlighting specific genetic fixation for each apple chromosome. Of all chromosomes, chromosome 16 is characterized by the highest LD value, showing an LD block of 2675 kb (Fig. 5B). These results indicate that the extent of LD in apple is shorter than in peach (Micheletti *et al.*, 2015) but larger than that in other species (e.g. grapevine; Myles *et al.*, 2009).

### Fruit texture genetic dissection by GWAS

The QTLs identified with the PBA approach were further complemented by GWAS. From the entire germplasm collection assessed to estimate the LD decay, 233 accessions were used for both population structure and marker–trait association. Individuals were assigned to three subpopulations (K=3; Supplementary Fig. S6) following the plateau criterion (Falush *et al.*, 2007), the non-parametric Wilcoxon test (Rosenberg *et al.*, 2002), and the ΔK method proposed by Evanno *et al.* (2005). Beyond this point, the mean log-likelihood values tend to a plateau together with an increased SD, which became clearly evident from K=5.

From the 20K SNPs present in the array, 10558 were finally exploited in the GWAS computation, performed with the MLM implemented in TASSEL v3.0. As a first attempt to dissect the genetic control of apple fruit texture, the two principal components (PC1 and PC2) were initially implemented as phenotypic traits. In this case, PC1 was considered to capture the overall texture variability, the entire group of parameters being oriented towards its projection (Fig. 1C, D). PC2, instead, was employed to discriminate the mechanical from the acoustic subset of variables. The MLM module identified for PC1 a major QTL on chromosome 10 (Supplementary Fig. S7A) with 10 markers exceeding the FDR-corrected *P*-value threshold. The phenotypic variability explained by the markers spanned from 9% to 13% and were found positioned on the consensus map between 42 cM and 47 cM (Table 2). Within this marker set, it is worth highlighting five SNPs, coded as FEM_cg_9, 11, 17, 18, and 19, which are custom SNPs specifically designed on polymorphisms discovered on re-sequencing the full length of *MdPG1*, a gene playing a key role in the fruit softening process in apple (Wakasa *et al.*, 2006; Costa *et al.*, 2010*b*; Longhi *et al.*, 2012). In particular, FEM_cg_19, also named Md-PG1SNP (Baumgartner *et al.*, 2016), is an SNP highly correlated with the microsatellite marker Md-PG1_SSR_10kd, previously associated with the fruit texture behavior in apple (Longhi *et al.*, 2013). In contrast, when PC2 was used in the association analysis not a single SNP was identified as statistically significantly associated (Supplementary Fig. S7B). It is moreover worth noting that when single texture subtraits were used as phenotype, the association result was consistent with the profile obtained for PC1, as shown in Supplementary Fig. S7C and D for the maximum force and number of acoustic peaks, respectively. Although the two groups of variables are oriented towards two different PCA quadrants, they are, however, commonly projected along the PC1 orientation (Fig. 1D). Within the panel of apple accessions employed here, PC1 accounts for 79.6% of the total phenotypic variance, thus influencing the genetic association of each single texture parameter. PC2, instead, is orthogonally oriented with respect to PC1 and more related to the difference between the two groups of variables, and therefore more effective in the dissection of the genetic control of the two texture signatures.

**Table 2.** SNPs exceeding the statistical threshold in the association analysis computed with PC1 (depicted in Supplementary Fig. S7A) For each SNP, the chromosome on which the marker is mapped (Chr), the genetic position (cM), the *P*-value, the name (SNP), and the percentage of phenotypic variability explained (*r*^2^) are indicated

Chr	cM	*P*-value	SNP	*r* ^2^
5	4.841	5.19E-06	FB_0597458_L5_PA	0.125730941
10	42.241	2.44E-06	FB_0832819_L10_41_1	0.134019865
10	42.241	4.31E-05	FB_0028781_L10_PA	0.117406088
10	42.241	4.40E-05	FEM_cg_19	0.10254861
10	42.843	2.60E-05	FEM_cg_9	0.108222257
10	42.843	2.60E-05	FB_0832811_L10_41_1	0.108222257
10	42.843	2.60E-05	FEM_cg_11	0.108222257
10	42.843	2.60E-05	FEM_cg_18	0.108222257
10	42.843	2.60E-05	FEM_cg_17	0.108222257
10	47.79775	1.68E-05	FB_0032582_L10_PA	0.094505374

**Fig. 6.**
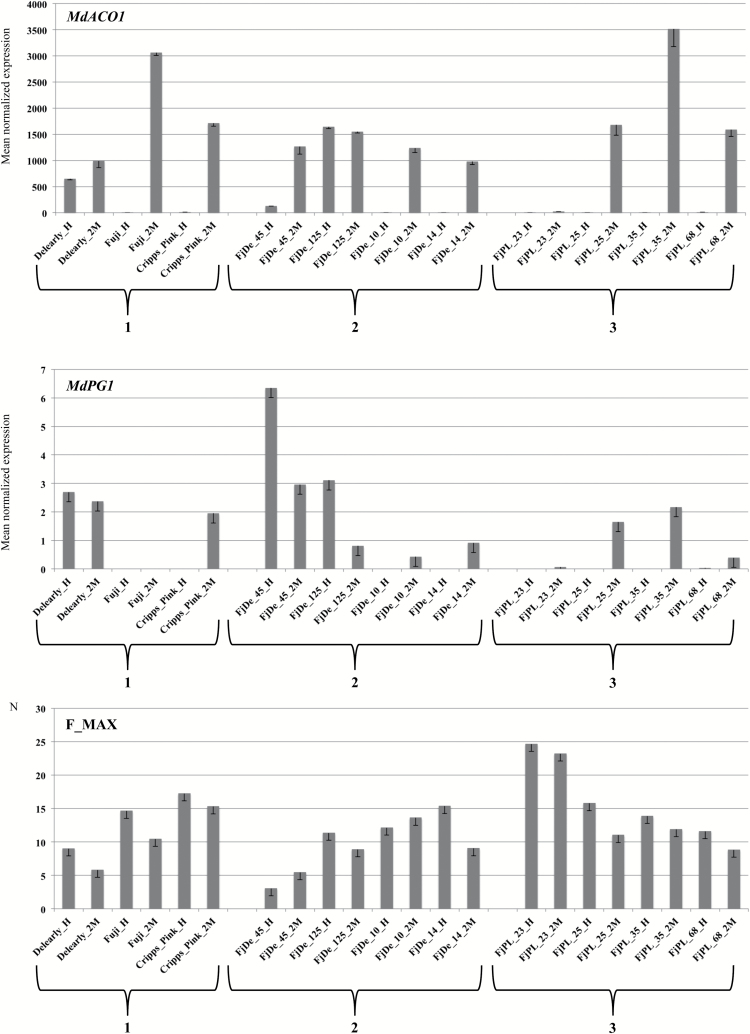
*MdACO1* and *MdPG1* expression profile and fruit firmness assessment. The transcript accumulation together with the phenotype measurement have been performed in three groups of plant materials indicated by numerical codes as 1, parental lines; 2, four individuals of the ‘FjDe’ population; and 3, four individuals of the ‘FjPL’ population. Each genotype is also distinguished by two samples, H, harvest; PH, post-harvest. In the first two panels (referring to *MdACO1* and *MdPG1* expression) the *y*-axes indicate the mean normalized gene expression, while for the last panel, maximum force is reported in Newtons (N).

**Fig. 7.**
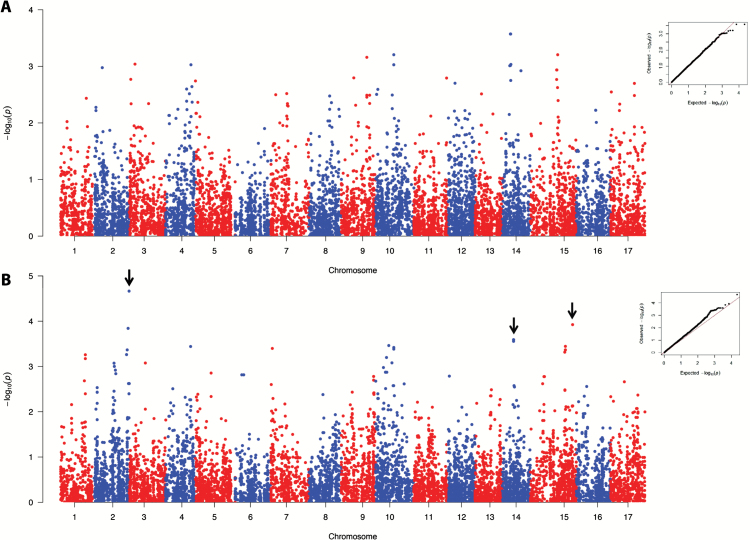
Manhattan plot illustrating the association between SNP markers and two texture subtraits, maximum force (A) and number of acoustic peaks (B), computed in the selected panel of apple accessions. The *x*- and *y*-axes report the number of chromosomes and the –log_10_(*P*-value), respectively. For both panels, the Q–Q plot is also reported. (This figure is available in colour at *JXB* online.)

To decipher more specifically the genetic regulation of fruit texture properties, a second round of association was performed. Since it is already well known that crisp apples are more appreciated by consumers, from the initial set of accessions used in GWAS, genotypes distinguished by the unfavorable homozygous allelic configuration for *MdPG1* and *MdACO1* were removed. The effect of these two genes on the fruit texture in apple largely depends on the interaction of the physiological processes they control. *MdPG1* is involved in the dismantling of the cell wall/middle lamella structure (Brummell and Harpster, 2001; Brummell, 2006) and its effect in apple seems to be more relevant than in other climacteric species. In tomato, in fact, the role of this gene alone does not impact the fruit texture physiology significantly (Sheehy *et al.*, 1988; Smith *et al.*, 1988; Giovannoni *et al.*, 1989). The other gene, *MdACO1*, regulates the last step of ethylene biosynthesis (Bleecker and Kende, 2000). Although in climacteric fruit the amount of this hormone is known to control several processes (Rose *et al.*, 1998; Costa *et al.*, 2005; Wakasa *et al.*, 2006), the co-existence of ethylene-dependent and -independent regulation of fruit texture also has been proposed, as demonstrated in melon (Nishiyama *et al.*, 2007) as well as in apple (Tadiello *et al.*, 2016). This dual mechanisms can explain why QTLs for fruit firmness have been collocated with *MdPG1* but not *MdACO1*. To investigate the consistency between the QTL genotypes estimated by FlexQTL™ and the expression of a gene included in the corresponding genomic interval, the transcript accumulation of *MdPG1*, together with *MdACO1*, was assessed. The transcript profiling was carried out in two groups of seedlings chosen between ‘FjDe’ and ‘FjPL’ populations. Among the four individuals selected in the first population, two (FjDe_10 and FjDe_14) were characterized by a ‘*QQ*’ genotype estimated for the QTL on chromosome 10 (and coincident with the genetic position of *MdPG1*), while the other two (FjDe_45 and FjDe_125) were distinguished by a ‘*Qq*’ genotype. These two QTL genotypes are, moreover, consistent with the allelotype configuration of Md-PG1SNP. The ‘*Q*’ and ‘*q*’ alleles are in fact linked to the ‘C’ and ‘T’ allele of this marker, in agreement with the genotype of the parental lines. ‘Fuji’ (‘*QQ*’ estimated genotype) is indeed distinguished by a ‘CC’ allelic state for Md-PG1SNP, while ‘Delearly’ has an ‘TC’ allelotype. The ‘T’ allele (related to the ‘*q*’ QTL allele) therefore segregates within the ‘FjDe’ population, contributing to a loss of fruit firmness. This association is further validated by the Pearson correlation value (*R*^2^ –0.8) between *MdPG1* expression and the fruit firmness assessment depicted in Fig. 6. In both groups of parental lines (group 1 in Fig. 6) and ‘FjDe’ individuals (group 2), it is clear that high loss of firmness corresponds to high *MdPG1* expression. The genotypes distinguished by a ‘*q*’ allele (FjDe_45, FjDe_125, and the parental variety ‘Delearly’) are in fact characterized by a considerable *MdPG1* expression already at harvest. This observation was additionally confirmed by the analysis carried out on the second population, ‘FjPL’. Since the two parents (‘Fuji’ and ‘Cripps Pink’) do not segregate for this QTL, all four seedlings (group 3) are characterized by a ‘*QQ*’ estimated genotype (MdPG1SNP_CC). Due to the role of these two genes, the breeding activities oriented towards the selection of firm and crisp apples no longer consider cultivars with these unfavorable genotypes as parental lines. Although this second panel of accessions is composed of only 64 individuals, it captures the real phenotypic variability used nowadays by breeders. This second GWAS was carried out with a GLM, selected on the basis of the Q–Q plot inspection. The result of this re-shaped phenotypic variance, obtained by fixing the effect of the two loci, was evident in the association analysis depicted in Fig. 7 and computed for the maximum force (Fig. 7A) as well as the number of acoustic peaks (Fig. 7B). In both associations, no major QTL on chromosome 10 was detected, especially in the case of the mechanical parameter. In contrast, when the number of acoustic peaks was considered, other regions were identified and located on chromosomes 2, 14, and 15 (Fig. 7B; Supplementary Table S6). These results provide evidence about the distinct genetic control for the two texture properties in apple, and suggest the role of chromosome 14 in the determination of acoustic properties, as underlined by the PBA results.

### QTL anchoring and *in silico* gene annotation

To investigate further the role of the QTL mapped on chromosome 14 in the regulation of the acoustic component of fruit texture, an *in silico* search and annotation of the candidate genes included in the genomic interval, together with their transcription profiling, was carried out. Genes were searched and annotated within an interval of 400 kb (LD block) up and downstream from the QTL peak determined by both the PBA and GWAS approach (Table 3). Among them, it is important to highlight categories of importance. The first is represented by proline-rich proteins (PRPs). This class of cell wall-modifying proteins (CWMPs) incluses proline and hydroxyproline peptides and seems to be involved in the cell wall metabolism of several species, such as cotton, carrot, and Arabidopsis (John and Keller, 1995; Fowler *et al.*, 1999; Holk *et al.*, 2002). It is also interesting to note that PRP-related genes are expressed in immature watermelon fruit (Guo *et al.*, 2011), and therefore not related to the late ripening dismantling process of the cell wall. Another important CWMP class is represented by expansin, a type of protein involved in the architectural re-modeling of the cell wall causing a disruption of the non-covalent bonds between the hemicellulose matrix and cellulose microfibrils (Cosgrove, 2000), exposing the cell wall structural polymers to the action of other CWMPs. Within the QTL computed with GWAS, a xylanase (xylo-glucangalactosyltransferase) was also found. Heteroxylans are a divergent group of polymers contributing to the cell wall structure, although in dicots they are less abundant than xyloglucan (Johnston *et al.* 2013). Xylanases are thus involved in the cellulose–non-cellulosic framework, and their role has already been reported in fruit. This gene was in fact expressed during the fruit softening process in papaya, according to an ethylene-dependent pattern (Manenoi and Paull, 2007). Finally, it is also worth mentioning the identification of a fucosyltransferase, an enzyme involved in xyloglucan metabolism, as documented in Arabidopsis (Rocha *et al.*, 2016). To assess further the mode of action of these genes in the regulation of fruit texture, the whole transcriptomic survey presented by Tadiello *et al.* (2016) was re-examined. In this particular case, the expression profile of two expansins (MDP0000423907 and MDP0000193025) and a fucosyltransferase (MDP0000230681) was selected from the analysis carried out with the whole-genome apple array (WGAA). The expression analysis of these elements was retrieved from three specific samples of the reference apple cultivar ‘Golden Delicious’, at harvest and after 1 week of post-harvest shelf-life ripening under both normal and 1-methylcyclopropene (1-MCP)-treated conditions (Supplementary Fig. S8). As described in Tadiello *et al.* (2016), the control post-harvest sample is characterized by an important loss of acoustic performance with regards to harvest, while the application of the ethylene competitor (1-MCP) effectively limited the cell wall degradation. Interestingly, the genes identified here and associated with the regulation of the acoustic component of texture are not stimulated by ethylene and do not participate in the major cell wall-degrading events, since their expression does not change from harvest to post-harvest. One expansin, in particular (MDP0000423907), is induced when the acoustic performance is promoted (1-MCP treatment), meaning that its role, rather than in the dismantling process leading to softening, is an involvement in the maintenance of the cell wall architectural structure.

**Table 3.** Gene annotation within the QTL interval mapped on chromosome 14 and identified through both GWAS (upper part) and PBA (lower part) approaches For each gene, the NCBI and GDR gene ID, gene function, and conting co-ordinates are reported. In addition, the closest SNP with its relative genomic information is also provided

Gene ID	Gene function	MDC name	MDC dtart	MDC dnd	Flanking SNP	MDC name	MDC start	MDC end
gi|658000264|ref|XM_008394357.1| MDP0000126350	Proline-rich protein	MDC011541.208	17995930	18011488	FB_0240837_L14_PA	MDC018357.590	17637582	17670933
gi|658000266|ref|XM_008394359.1| MDP0000155446	Proline-rich protein	MDC013704.625	18033491	18049518	FB_0240837_L14_PA	MDC018357.590	17637582	17670933
gi|658000211|ref|XM_008394331.1| MDP0000423907	Pistil-specific extensin-like protein	MDC010624.545	16734875	16765614	RB_20380789_L14_41_1	MDC021233.163	17000850	17040029
gi|658000230|ref|XM_008394341.1| MDP0000775334	Xylo- glucangalactosyltransferase	MDC007808.273	17603144	17620726	RB_21013914_L14_PA	MDC018357.590	17637582	17670933
gi|658063270|ref|XM_008369329.1| MDP0000151618	Expansin-A1	MDC015296.157	25922508	25924244	FB_0254699_L14_PA	MDC012426.235	26135397	26155901
gi|658001413|ref|XM_008394948.1| MDP0000193025	Expansin-A1	MDC009270.341	25922966	25925030	FB_0254699_L14_PA	MDC012426.235	26135397	26155901
gi|658001426|ref|XM_008394954.1| MDP0000230681	Fucosyltransferase	MDC014060.237	26024637	26027504	FB_0254699_L14_PA	MDC012426.235	26135397	26155901

### Conclusion

Fruit texture in apple is made up of multiple subtraits, most of which (especially the acoustic ones) are poorly investigated, in particular for genetic purposes. So far, this limitation led to the identification of markers suitable to assist in the selection of fruit firmness only, although, for apple, the feature most preferred by consumers is crispness. The coupling of PBA with GWAS enabled the genetic deciphering of fruit texture control, identifying important QTLs associated with both texture features. The comparison of the results obtained by the two genetic approaches highlighted an inventory of genomic intervals specifically associated with mechanical and acoustic parameters, respectively, hypothesizing that these subtraits are effectively controlled by different genetic mechanisms. In the near future, in the new high quality breeding materials, the alleles of the markers currently in use will be quickly fixed as a result of recurrent rounds of marker-informed parental selection (MAPS; marker-assisted parent selection) and the subsequently assisted selection of seedlings (MASS; marker-assisted seedling selection). The information presented here can therefore be taken into consideration to design novel markers useful to identify novel apple accessions distinguished by superior fruit crispness.

## Supplementary Material

Supplementary Data
