# Supplementary material for: Deciphering the genetic control of fruit texture in apple by multiple family-based analysis and genome-wide association
Source: J Exp Bot. 2017 Feb 24;68(7):1451–66. doi: 10.1093/jxb/erx017 (PMC5441909; doi:10.1093/jxb/erx017)

Deciphering the genetic control of fruit texture in apple by multiple-family based analysis and genome-wide association

Mario Di Guardo, Marco Bink, Walter Guerra, Thomas Letschka, Lidia Lozano, Nicola Busatto, Lara Poles, Alice Tadiello, Luca Bianco, Richard Visser, Eric Eric Van de Weg, and Fabrizio Costa

Supplementary Figures

Supplementary Fig. S1
Pedigree structure of the six full-sib families. Maternal and paternal connections are depicted with red and blue colored lines, respectively. Red boxes indicate populations, while parental cultivars and founders are highlighted in purple and blue color, respectively. The six populations are coded as follow; FjDe: 'Fuji' x 'Delearly', GDFj: 'Golden Delicious' x 'Fuji', FjPL: 'Fuji' x 'Cripps Pink', GaPL: 'Gala' x 'Cripps Pink', GaPi: 'Gala' x 'Pinova', FjPi: 'Fuji' x 'Pinova'.

Supplementary Fig. S2
QTL trace plot position output of FlexQTL showing the convergence of each single run. On the x-axes is reported the cumulative genetic distance (in cM), while on the y-axes the trace is illustrated.

Supplementary Fig. S3
Overall QTL probability profile defined for each parameter over the entire genome. Each parameter is indicated by a numerical code (on the right side) according to the caption of Fig. 1.

Supplementary Fig. S4
For each chromosome the correlation between the physical and genetic position of each SNP marker employed in the analysis is shown.

Supplementary Fig. S5
LD decay plot and Haploview LD pattern for each apple chromosome.

Supplementary Fig. S6
Inferred population structure. In panel A the plot generated by STRUCTURE according to the K=3 model, as suggested by the K method illustrated in panel B, is reported.

Supplementary Fig. S7
Genome-wide association scan of the association between SNP marker loci and PC1 (panel A), PC2 (panel B), maximum force (panel C) and number of acoustic peaks (panel D) computed with the collection of 233 apple accessions. For each Manhattan plot, chromosomes are indicated on the x-axes, while on the y-axes the –log10(P-value) is reported. The dashed horizontal line points the threshold for declaring a SNP as significant on the base of the adjusted P-value (FDR<0.05). On the top right part of each plot, the quantile-quantile (Q-Q) plot distribution is also illustrated.

Supplementary Fig. S8
Expression profile of three genes (MDP0000423907 in blue, MDP0000193025 in red and MDP0000230681 in green) in three samples of 'Golden Delicious', according to the work of Tadiello et al. (2016). Samples are coded as: H (harvest), PH (1 week of shelf-life postharvest ripening) and PHM (1 week of shelf-life postharvest ripening treated with 1-MCP).

Supplementary Fig. S1

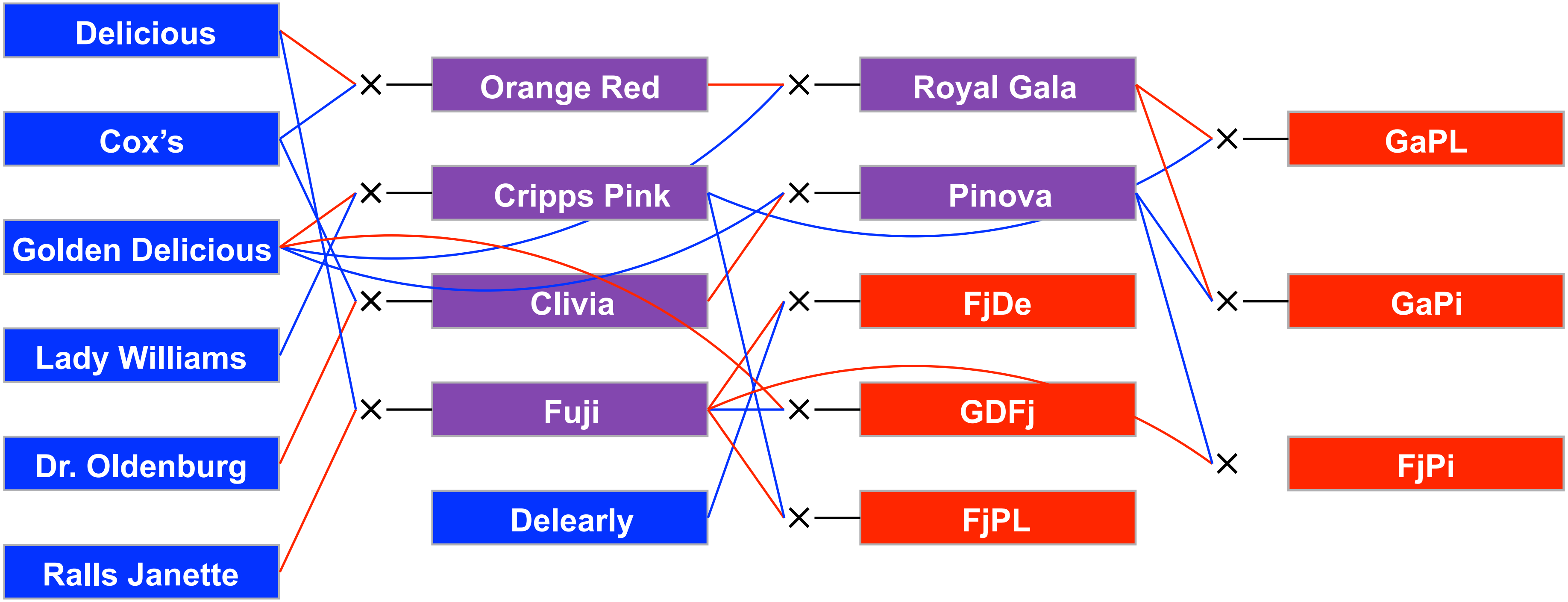

Supplementary Fig. S2

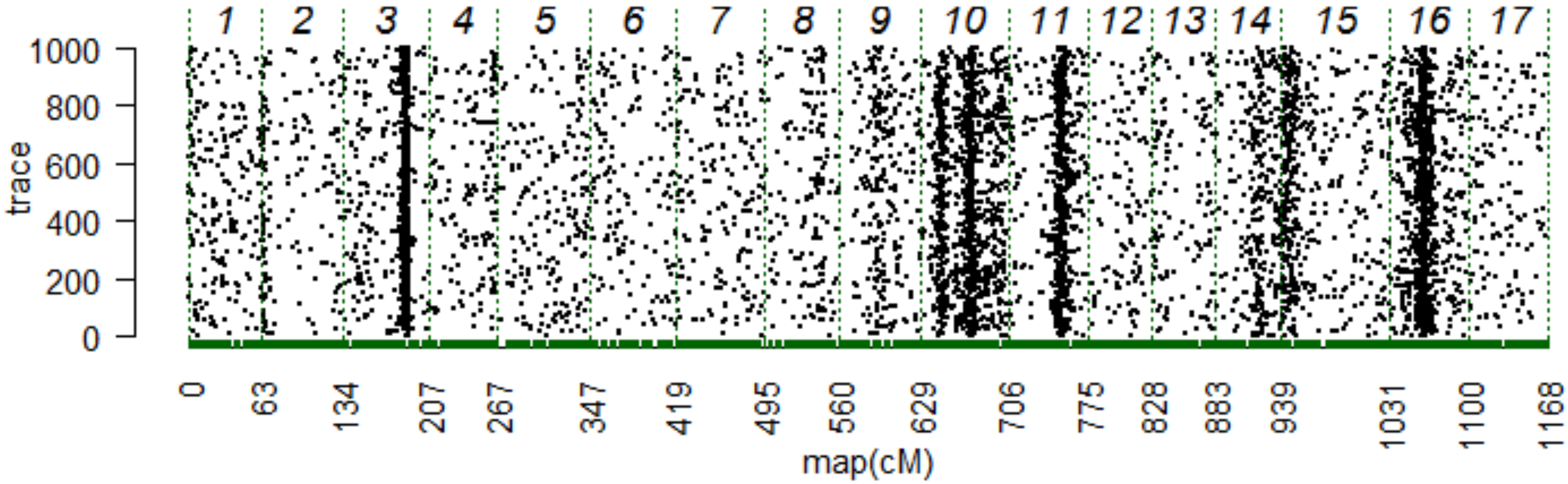

Supplementary Fig. S3

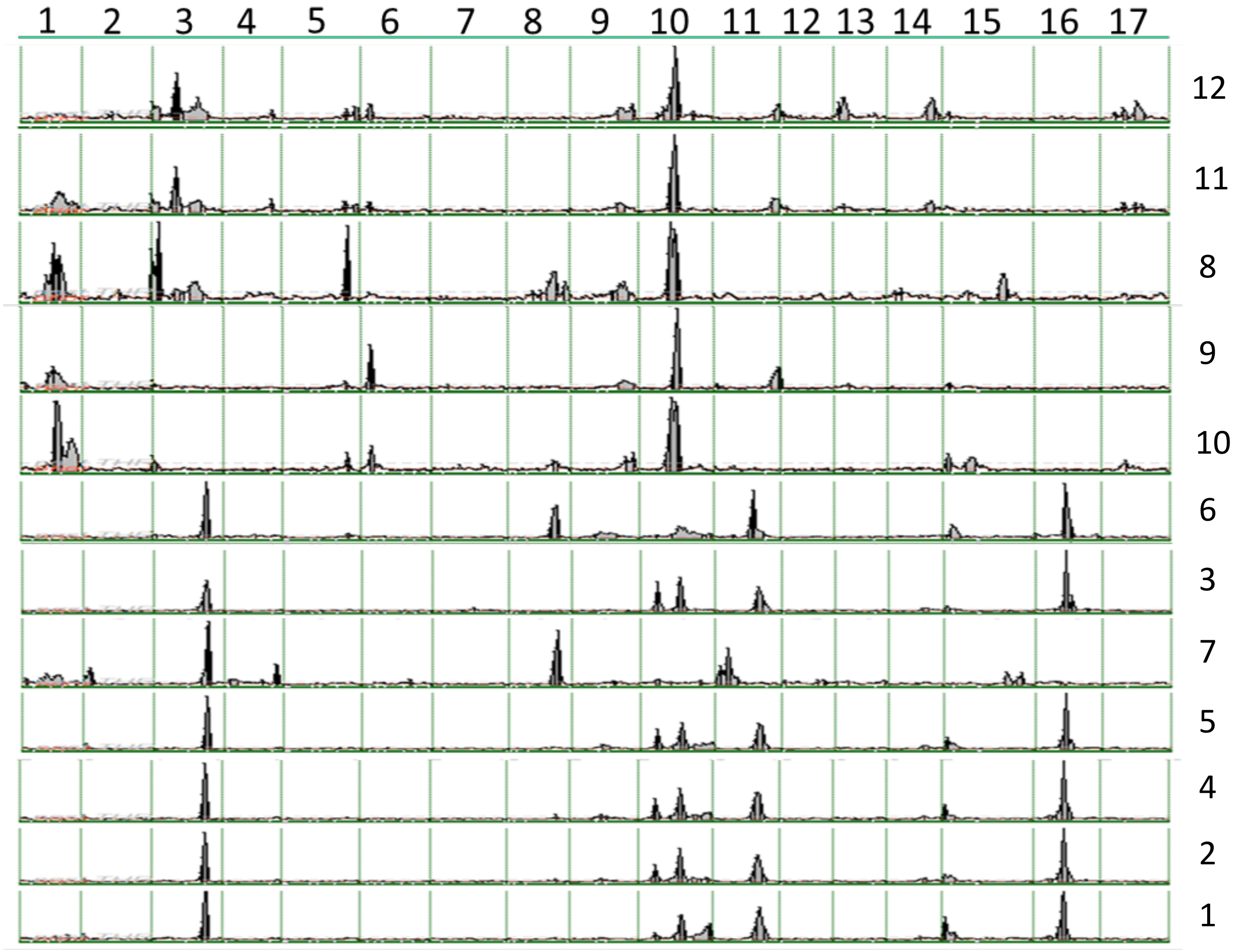

Supplementary Fig. S4

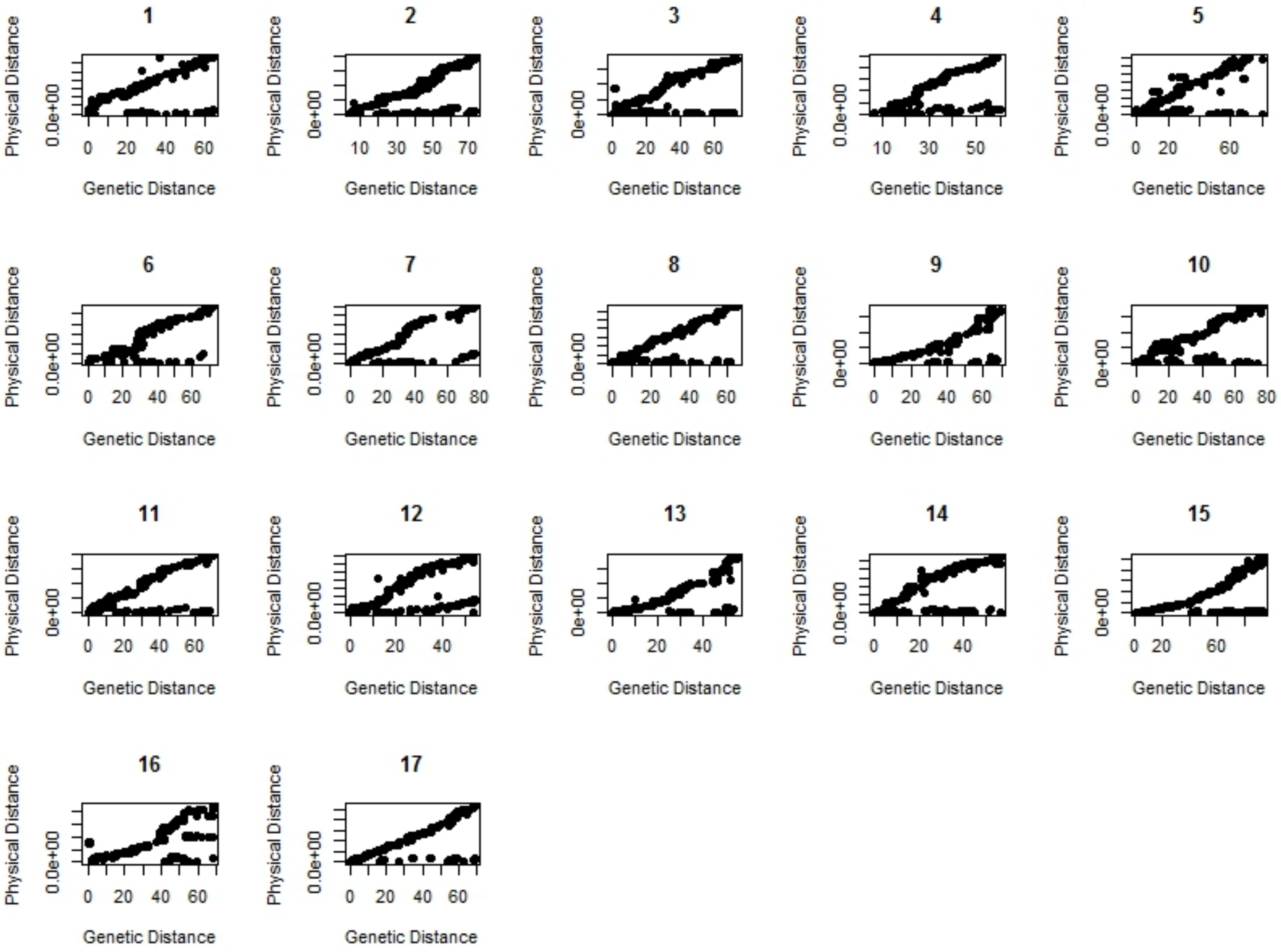

Supplementary Fig. S5

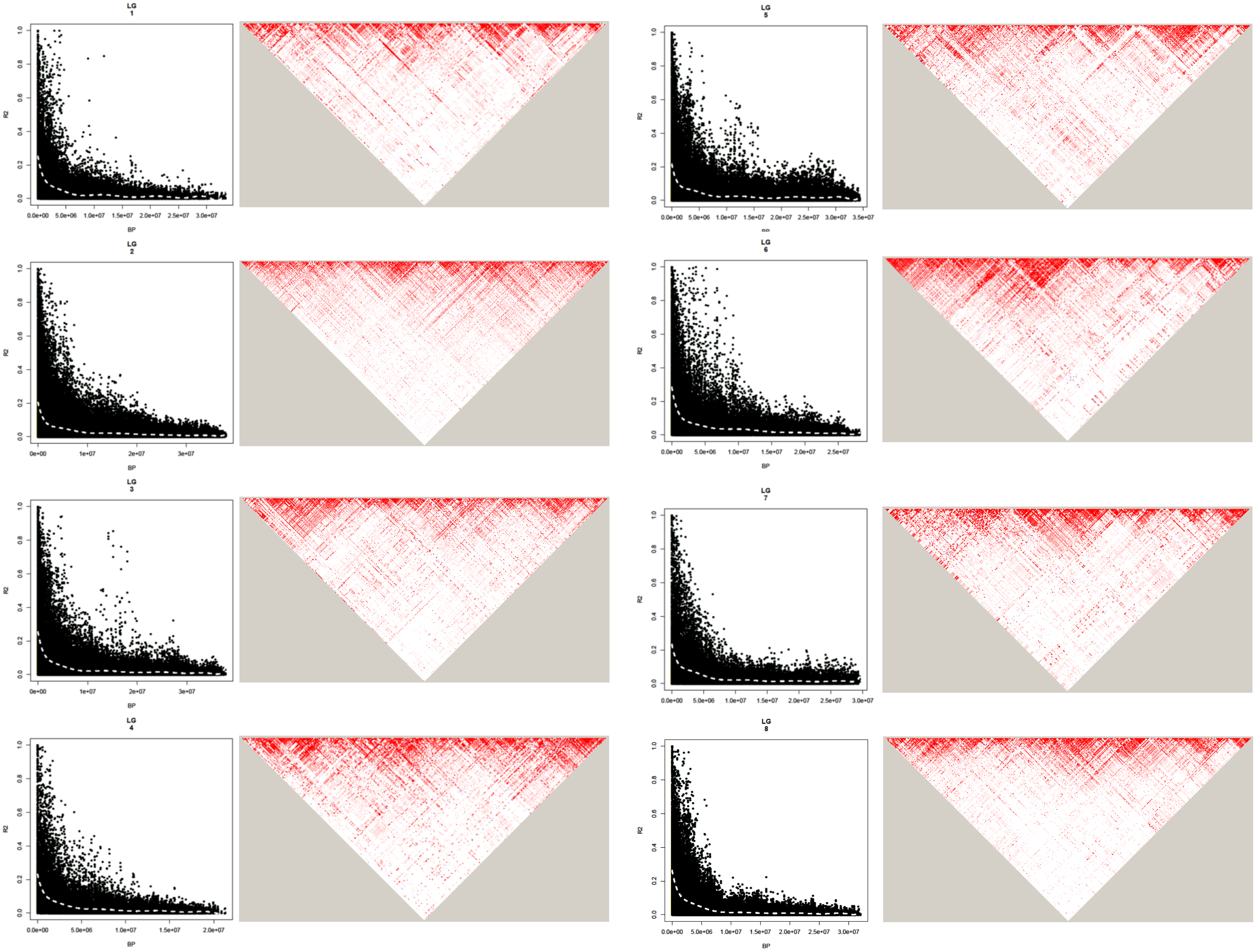

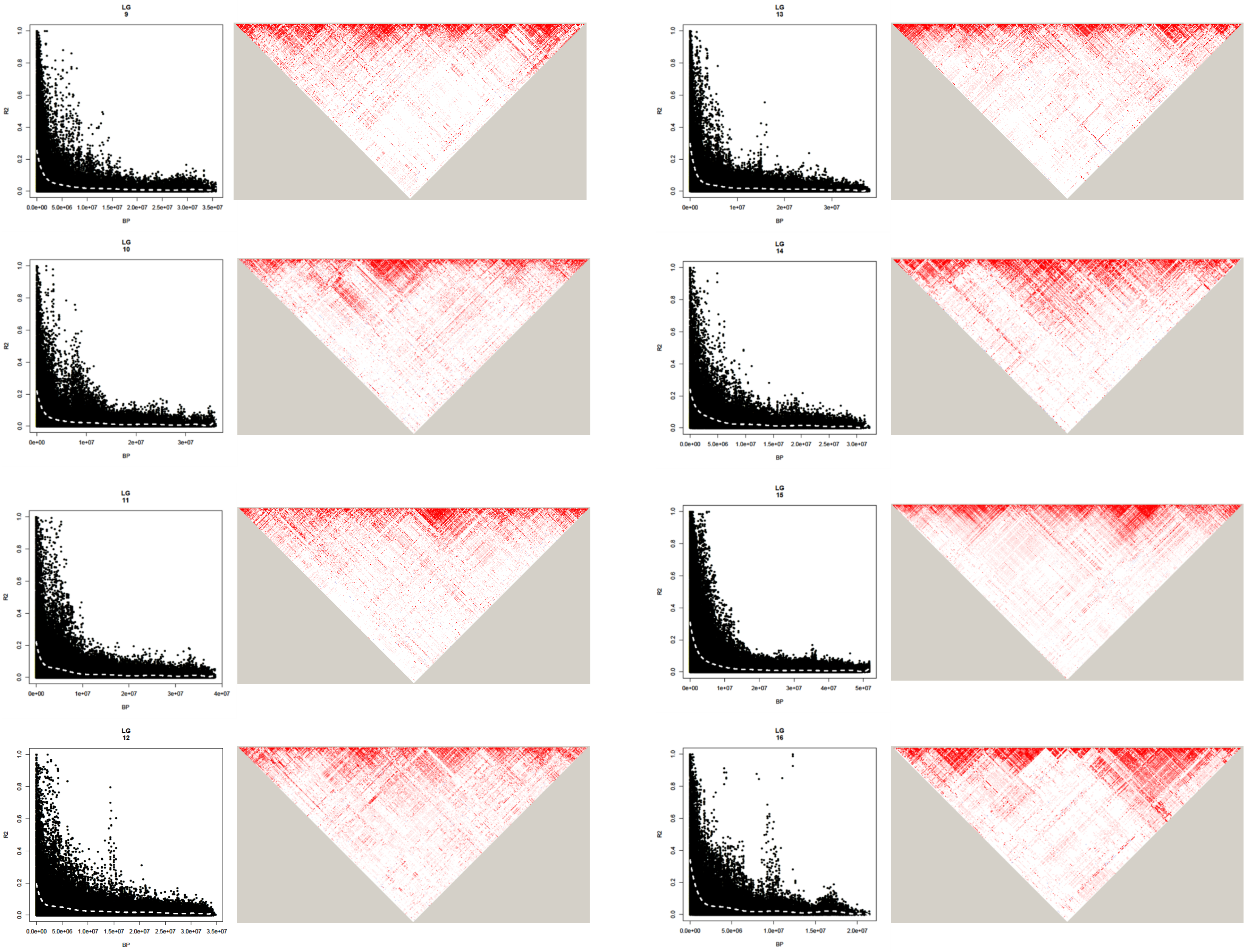

LG 9
LG 10
LG 11
LG 12
LG 13
LG 14
LG 15
LG 16
R2
BP
0.0
0.2
0.4
0.6
0.8
1.0
0.0e+00 5.0e+06 1.0e+07 1.5e+07 2.0e+07 2.5e+07 3.0e+07 3.5e+07
0e+00 1e+07 2e+07 3e+07
0e+00 1e+07 2e+07 3e+07 4e+07
0.0e+00 5.0e+06 1.0e+07 1.5e+07 2.0e+07 2.5e+07 3.0e+07 3.5e+07
0e+00 1e+07 2e+07 3e+07
0.0e+00 5.0e+06 1.0e+07 1.5e+07 2.0e+07 2.5e+07 3.0e+07
0e+00 1e+07 2e+07 3e+07 4e+07 5e+07
0.0e+00 5.0e+06 1.0e+07 1.5e+07 2.0e+07

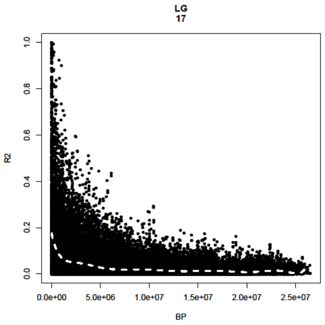
LG
17
R2
1.0
0.8
0.6
0.4
0.2
0.0
0.0e+00
5.0e+06
1.0e+07
1.5e+07
2.0e+07
2.5e+07
BP

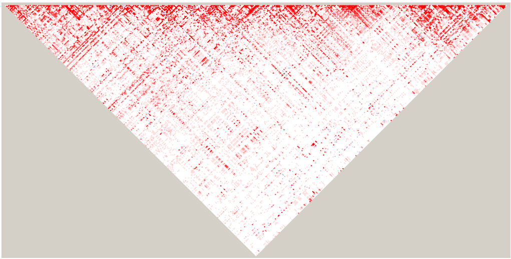

Supplementary Fig. S6

A

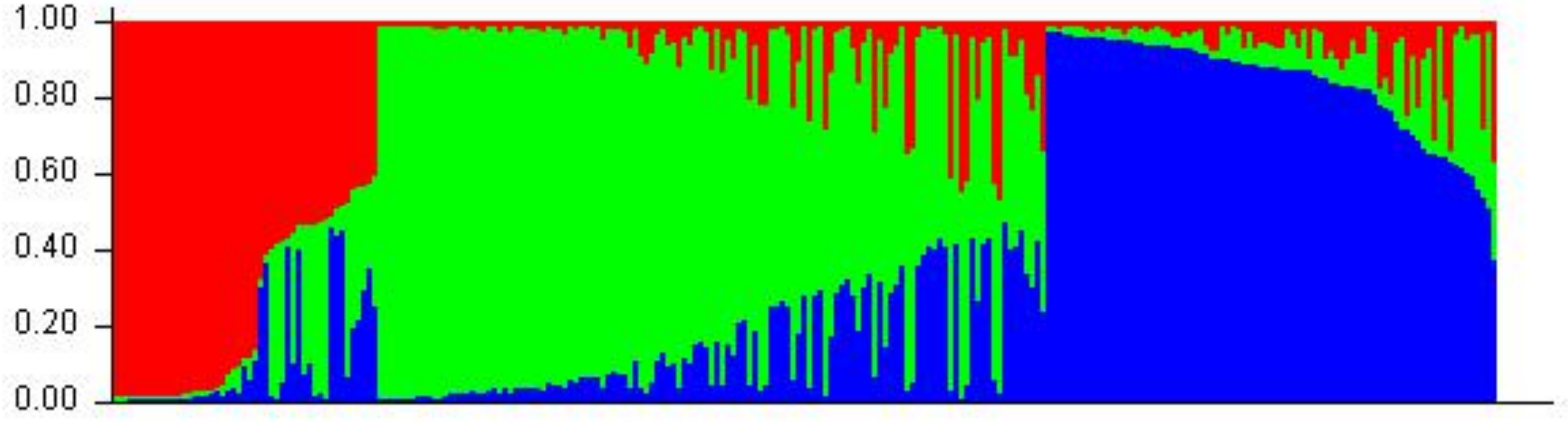

B

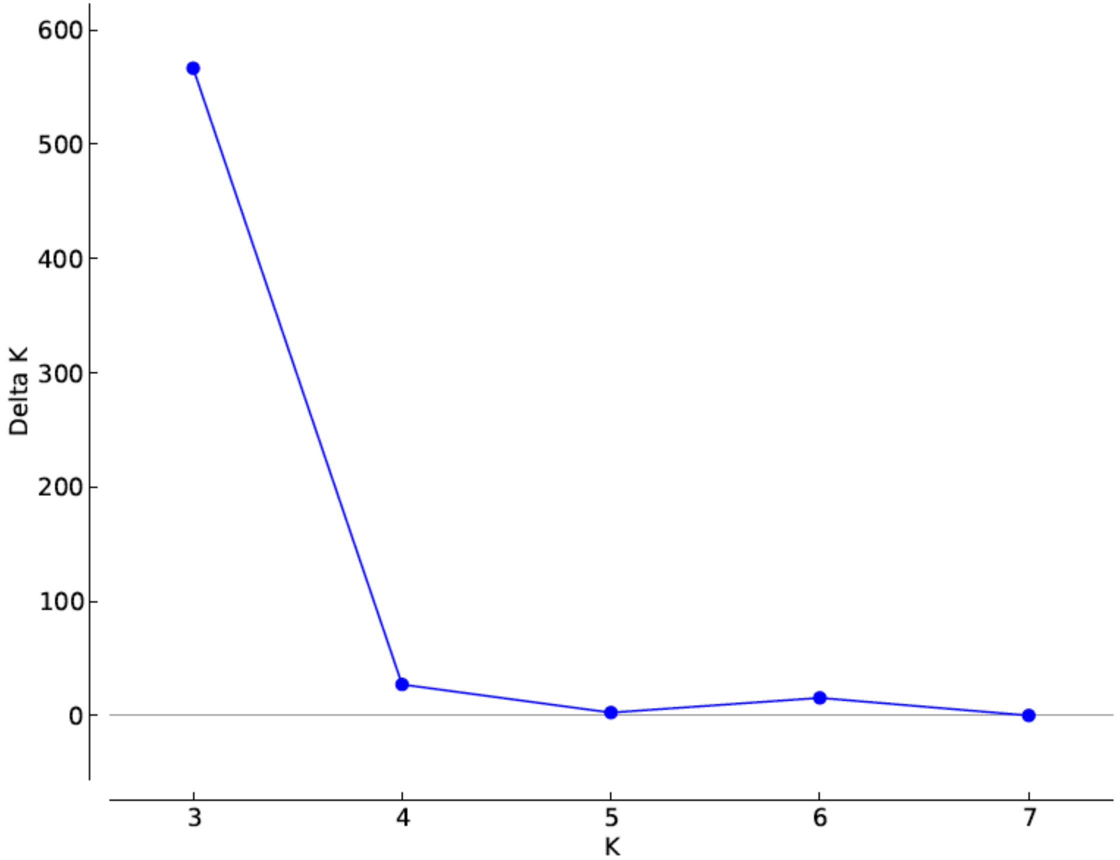

Supplementary Fig. S7

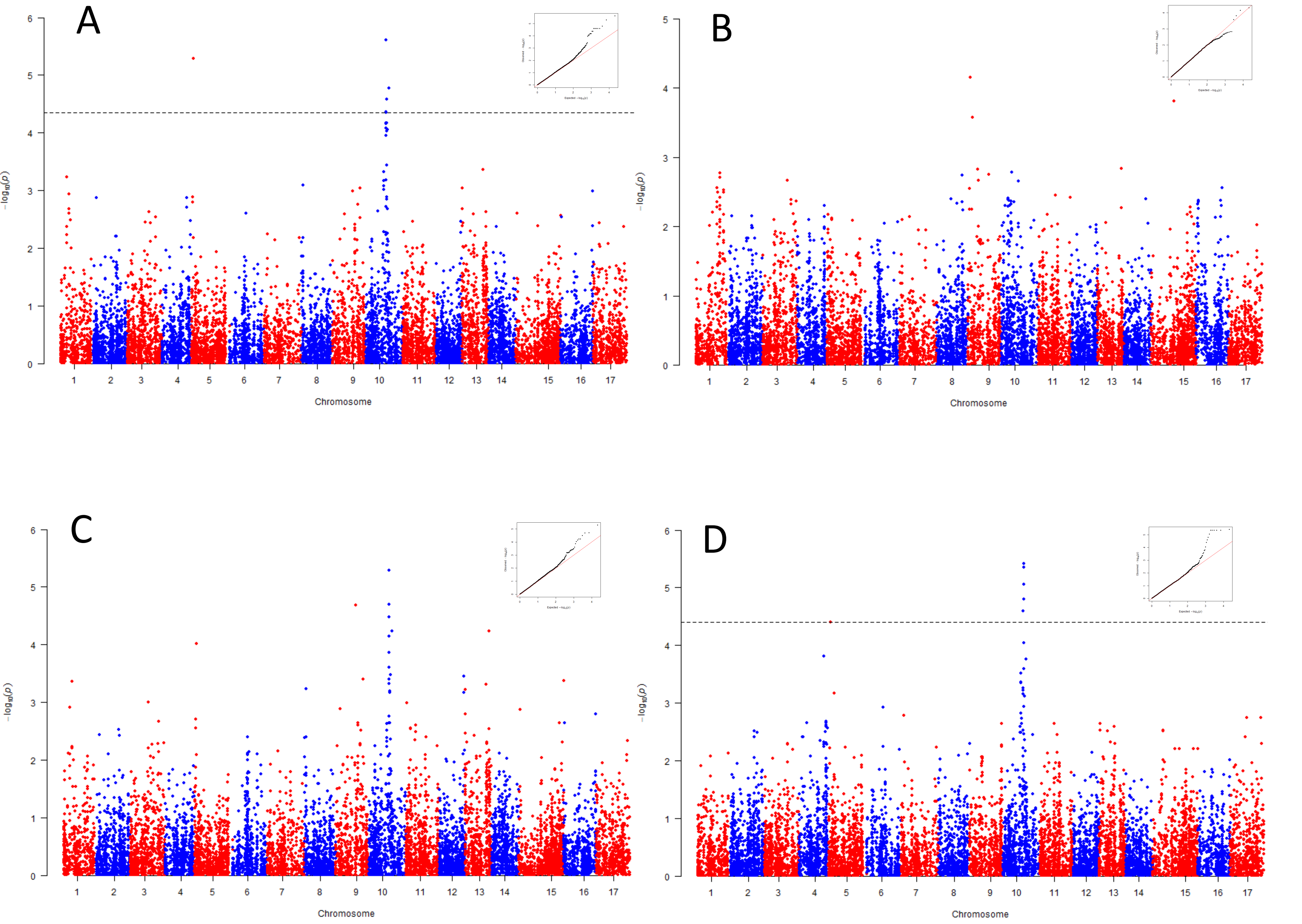

Supplementary Fig. S8

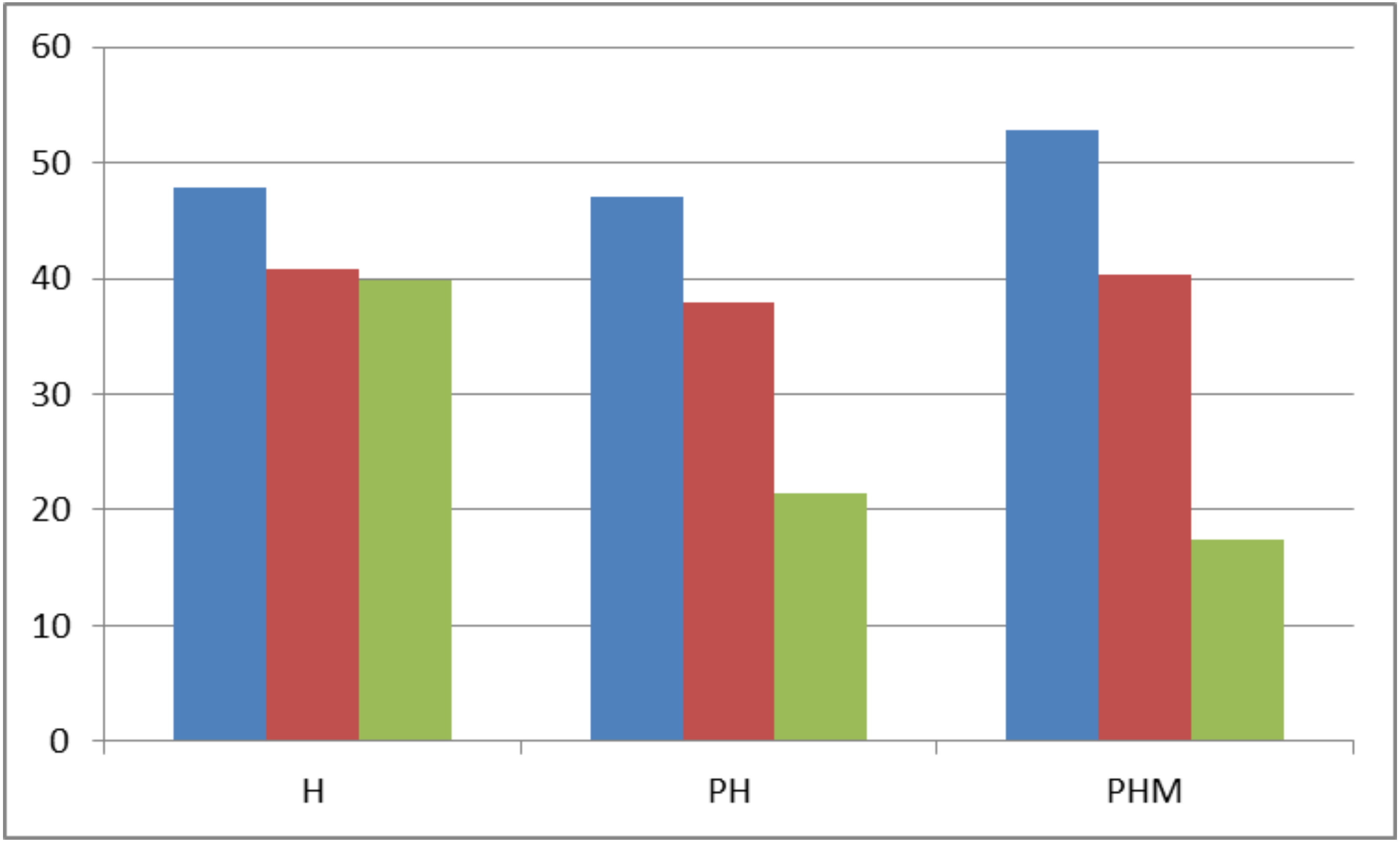

Supplement: Supplementary Data [file erx017_Supplementary_Data.zip › supplementary_figures_S1_S8.pdf]
