# Supplementary material for: Deciphering the genetic control of fruit texture in apple by multiple family-based analysis and genome-wide association
Source: J Exp Bot. 2017 Feb 24;68(7):1451–66. doi: 10.1093/jxb/erx017 (PMC5441909; doi:10.1093/jxb/erx017)
Supplement: Supplementary Data [file erx017_Supplementary_Data.zip › supplementary_tables_S1_S6.pdf]

Supplementary Table S1

	**Delearly**	**Fuji**	**Cripps Pink**	**Pinova**
**Fuji**	**FjDe** (69)		**FjPL** (82)	**FjPi** (93)
**Royal Gala**			**GaPL** (32)	**GaPi** (43)
**Golden Del**		**GDFj** (84)		

Supplementary Table S2

Cultivar Name	GWAS Dataset	Selected Dataset
ABBONDANZA	x	x
ADAM APFEL	x	
ADERSLEBER KALVILL		
AKANE	x	
ALICE	x	
ALKA	x	
ALKMENE	x	
ALMAGOLD	x	
ALTER WILDLING		
AMASIA	x	
AMBROSIA		
ANANAS RENETTE	x	x
ANGOLD	x	
ANNA	x	
ANNURCA CASERTA	x	
APOLLO	x	
ARANCIATA DI COX	x	
ARIANE		
ARIWA	x	
ARKANSAS	x	
ARLET	x	x
ASSUMPTA	x	
BABINE	x	
BALDWIN	x	x
BAUJADE	x	
BAUNEN	x	x
BEACON	x	
BEAUTY OF BATH	x	
BELCHARD CHANTECLER	x	x
BELFIORE DI TRENTO	x	
BERNER ROSEN		
BISMARKAPFEL		
BLAIRMONT	x	
BOIKENAPFEL		
BOSKOOP	x	
BOUTABGUIA	x	x
BOZNER APFEL		
BRAMLEYS SEEDLING		
BRINA	x	
BRIXNER PLATTLING		
CAKANSKA	x	x
CALAMARI	x	
CAMEO -CAUDLE	x	x

CAROLA	x	
CATARINA	x	x
CELLINI		
CHAMPAGNER RENETTE		
CHARLOTTE		
CIVG198		
CIVNI		
CLEOPATRA	x	x
CLIVIA	x	
CLOSE		
COMMERCIO	x	
COOP 1	x	
COOP 11	x	
COOP 15	x	x
COOP 16	x	x
COOP 26	x	x
COOP 28	x	
COOP 29	x	
COOP 3	x	
COOP 36	x	
COOP 38	x	
COOP 6	x	
COOP 7	x	
COOP 8	x	x
COOP 9	x	x
COOP 33		
COOP 39		
COOP 43		
CORTLAND	x	
COWICHAN		
COXS ORANGE PIPPIN	x	
CRANDALL	x	x
CRIMSON GOLD	x	
CRIMSON SNOW MC-38	x	x
CRIMSON BEAUTY		
CRIPPS PINK	x	
CRIPPS RED	x	
CRITERION	x	x
CRONCELS	x	
DALINETTE		
DALITRON 46-3		
DANZIGER KANTAPFEL	x	
DELBARD ESTIVALE	x	
DELBLUSH	x	
DELBRUS	x	
DELCOROS	x	

DELEARLY	x	
DELIA 73		
DELICIOUS		
DELPRIM	x	
DELSHEL	x	
DISCOVERY		
DIWA	x	
DJULABIA		
DOLGO		
DOMAINE	x	
DUREZZA VERDE VIENNESE	x	
EARLY GOLD	x	
EARLY RED STAYMAN	x	x
EARLY SMITH	x	
EARLY CORTLAND		
EDELBÖHMER	x	
ELSTAR	x	
EMILIA B9 1-28	x	x
EMPIRE	x	x
EMPRESS		
ENTERPRISE	x	
ERBACHHOFER		
FALCHS GULDERLING		
FANTASIA	x	
FESTIVAL	x	
FIAMMA	x	x
FLORIANER ROSMARIN		
FLORINA	x	
FRAGONI	x	
FREEDOM	x	x
FREIBERG	x	
FREIHERR VON BERLEPSCH		
FUJI MORI HOUFU 3A	x	x
FUJION	x	x
GAIA	x	x
GALICIA	x	
GALIWA		
GEFLAMMTER KARDINAL		
GEHEIMRAT DR. OLDENBURG		
GELBER EDELAPFEL	x	
GELBER RICHARD		
GENEVA	x	
GEWÜRZLUIKEN		
GINGER GOLD	x	

GLOSTER	x	
GOLDCHIEF	x	x
GOLDEN DELICIOUS	x	
GOLDEN ORANGE	x	
GOLDRENETTE VON BLENHEIM		
GORO	x	
GRAHAMS JUBILÄUMSAPFEL		
GRANNY SMITH	x	
GRAVENSTEINER T1606		
GREENSLEVES	x	
GRIMES GOLDEN	x	
GROßER BRÜNNERLING		
GRÜNER STETTINER		
HARALSON	x	x
HARBERTS RENETTE		
HARMONIE DELORINA	x	
HATSUAKI	x	x
HERREN BRÜNNERLING		
HOLIDAY	x	x
HONEY GOLD	x	
IDAGOLD	x	x
ILROD PIGEON	x	
INGRID MARIED	x	
IRISH PEACH		
JAMBA	x	
JAMES GRIEVE	x	
JOANTORPE	x	
JOHN DOWNIE		
JOLLY	x	
JONADEL	x	
JONAFREE	x	x
JONAMAC	x	x
JONASTY	x	
JONATHAN	x	x
JONICA	x	x
JUDELINE	x	
KAISER ALEXANDER		
KAISER WILHELM		
KALTERER BÖHMER	x	
KANADA RENETTE		
KANSAS K 14		
KARMELITTER RENETTE		
KATIA	x	
KENDAL	x	
KIDDS ORANGE RED		

KÖNIGINNENAPFEL		
KÖSTLICHER		
KRIPPELE APFEL		
KRONPRINZ RUDOLPH		
KSB 406-1		
LADY		
LADY WILLIAMS		
LANDSBERGER RENETTE		
LB 04715		
LB 04852		
LB 05338		
LB 08000		
LB 09669		
LB 13995		
LB 17906		
LB 18059		
LIBERTY	x	x
LIMONCELLA		
LIMONCINI	x	
LOBO	x	x
LODI	x	
LUIKENAPFEL		
LYGOL	x	
MACOUN	x	
MAGNOLIA GOLD	x	
MAIRAC - FLAMBOYANTE	x	x
MARCON		
MAYGOLD	x	
MC INTOSH	x	
MEGRÉ	x	
MEGUMI	x	x
MELA D INVERNO	x	
MELA FORESTIERA	x	
MELBA		
MELODY	x	
MELROSE	x	x
MERAN	x	
MERAVIGLIA RIGOTTI	x	
MERTON RUSSET		
MINISTER HAMMERSTEIN		
MONROE	x	
MOSEBAR	x	x
MURRAY	x	
MUSKAT RENETTE		
MUTSU	x	
NABELLA	x	

NAGANO NATHUSIUS TAUBENAPFEL		
NEW GOLD	x	x
NEWTON PIPPIN	x	x
NIAGARA	x	
NICOGREEN	x	
NICOTER		
NORTEN SPY	x	
NOVA EASYGRO	x	x
ODIN	x	
OKANAGAN	x	
OLDEMBURG X COX	x	
ONTARIO		
OPAL		
ORIN	x	x
ORION	x	
ORLEANS	x	x
OTTERSON		
OZARK GOLD	x	x
PACIFIC GOLD	x	
PARAGON WINESAP	x	x
PARKMAN		
PATRIZIA	x	
PEACHBLOW		
PETREL	x	
PFREISLING		
PI 589746 05		
PIATTONA		
PILOT	x	
PINK PERL	x	
PINOVA M9	x	
PIROL-PIRELLA	x	
PIROS	x	
PLATTMANTUANER		
PRECOCE RIGOTTI	x	
PRIAM	x	
PRIMA	x	
PRIME GOLD	x	x
PRIMIERA	x	
PRIMOLD DELJENI	x	
PRINZ ALBERT		
PRINZENAPFEL		
PRISCILLA	x	x
RALLS JANETTE		
RARITAN	x	

REANDA	x	x
RED APPLE	x	
RED BARON	x	
RED EARLY	x	
RED SEKAIICHI	x	x
RED FLESH		
RED IDARED		
RED JACKET		
REDFREE	x	
REGGENT	x	
REMO C	x	
RENOIRE	x	x
RESISTA	x	
REWENDA	x	x
RIBSTON PEPPING		
ROSA DI FONDO		
ROSA DI SEIO		
ROSEMARY RISSET	x	
ROSMARINA ROSA	x	
ROTE STERNRENETTE		
NORD SPEE		
ROTER EISERAPFEL		
ROTER GESTREIFTER ASTRACHAN		
ROTER GRIESAPFEL		
ROTER HERBSTKALVILL		
ROTER PALMAPFEL		
ROTER VON SIMONFI		
ROYAL GALA	x	x
RUBICON	x	x
RUBIN	x	
RUBINETTE RAFZUBIN	x	
RUBINOLA	x	
RUBIS		
RUBRA SEL. ZANZI	x	x
RUBY	x	x
RUSSIAN		
RVI02		
RVI04		
RVI05		
RVI08		
RVI09		
RVI10		
RVI11		
RVI12		
RVI13		

RVI14		
SAMERLING		
SAN LUGANO	x	
SANSA	x	
SANTARED FIRESIDE	x	
SATURN	x	
SCHMIDTBERGER RENETTE		
SCHÖNER VON NORDHAUSEN		
SCHÖNER VON WILTSHIRE		
SCHWEIZER ORANGENAPFEL		
SCIFRESH		
SCILATE		
SCOTIA		
SELENA	x	
SELLA	x	
SEPTEMBER	x	
SERIANA	x	
SHELLY		
SHENANDOAH	x	
SHIZUKA	x	x
SIGNE TILLISCH		
SMERALDA	x	
SOBERANA	x	
SOMMERGEWÜRZAPFEL		
SONYA NEVSON	x	x
SPÄTBLÜHENDER TAFFET		
SPENCER	x	
SPIJON		
SPITZLING		
SPOKANE BEAUTY	x	
SPYGOLD	x	
SPYON	x	
SQ 159		
STARK EARLIEST		
STAYMANRED LB78-1		
STEINPEPPING		
SUMMER CHAMPION	x	
SUMMERFREE	x	
SUMMERRED	x	
SUNGOLD	x	x
SUNRISE	x	
SURPRISE	x	
SÜßAPFEL		

TAVOLA BIANCA	x	
TIOGA	x	
TIROLER SPITZLEDERER		
TOPAZ	x	
TSUGARU	x	
TUNDA	x	
VESNA	x	x
VIKING	x	
VISTABELLA		
WAGNERAPFEL		
WEBERBARTLAPFEL		
WEBSTER	x	x
WEIDNERS GOLDRENETTE		
WEIROUGE		
WEIßER WINTERKALVILL	x	
WEIßER ASTRACHAN		
WEIßER TAFELAPFEL		
WEIßER WIESLING		
WELLINGTON	x	
WELSCH RASONER		
WELSCHBRUNNER		
WHITE ANGEL		
WILLIAM S PRIDE	x	
WINCESTER		
WINSTON	x	
WINTERBANANENAPFEL		
WORCESTER PERMAIN	x	
YELLOW SIBERIAN UK		
YELLOW TRANSPARENT		
ZIGEUNERAPFEL		

Supplementary Table S3

SNP	HAPLOTYPE	LG	cM
FB_0411238_L1_PA	fp01_00	1	0.186
FB_0400909_L17_PA	fp01_00	1	0.186
FB_0400910_L17_PA	fp01_00	1	0.186
FB_0197767_L13_PA	fp01_00	1	0.186
FB_1079972_L1_70_2	fp01_00	1	0.186
FB_0411246_L1_PA	fp01_00	1	0.186
FB_0197753_L13_PA	fp01_00	1	0.186
RB_3094248_L1_PA	fp01_00	1	0.186
FB_0313817_L15_PA	fp01_00	1	0.186
FB_0124504_L12_PA	fp01_00	1	0.186
FB_1079904_L1_67_2	fp01_00	1	0.186
FB_0409798_L1_PA	fp01_00	1	0.186
FB_0400919_L17_PA	fp01_00	1	0.186
FB_1089803_L3_81_2	fp01_00	1	0.186
FB_0400921_L17_PA	fp01_00	1	0.186
FB_0409694_L1_PA	fp01_00	1	0.186
FB_0197405_L13_PA	fp01_00	1	0.186
FB_0197756_L13_PA	fp01_00	1	0.186
FB_0409675_L1_PA	fp01_00	1	0.186
FB_0464009_L2_PA	fp01_00	1	0.186
FB_0764004_L8_PA	fp01_00	1	0.186
FB_0197766_L13_PA	fp01_00	1	0.186
FB_0764007_L8_PA	fp01_00	1	0.186
FB_1035153_L8_56_1	fp01_00	1	0.186
FB_0530049_L3_PA	fp01_00	1	0.186
FB_0530061_L3_PA	fp01_00	1	0.186
FB_0530052_L3_PA	fp01_00	1	0.186
FB_0530079_L3_PA	fp01_00	1	0.186
FB_0764008_L8_PA	fp01_01	1	1.233
FB_0464041_L2_PA	fp01_01	1	1.233
FB_0673802_L6_PA	fp01_01	1	1.233
FB_0411216_L1_PA	fp01_01	1	1.233
FB_0953660_L2_23_1	fp01_01	1	1.233
FB_1099449_L7_62_2	fp01_01	1	1.233
FB_0673806_L6_PA	fp01_01	1	1.233
FB_0673808_L6_PA	fp01_01	1	1.233
FB_0763988_L8_PA	fp01_01	1	1.233
FB_0953652_L2_23_1	fp01_01	1	1.233
FB_0411217_L1_PA	fp01_01	1	1.233
FB_0764324_L8_PA	fp01_01	1	1.233
FB_0464008_L2_PA	fp01_01	1	1.233
FB_1099420_L7_62_2	fp01_01	1	1.233
FB_0673809_L6_PA	fp01_01	1	1.233

FB_1099450_L7_62_2	fp01_01	1	1.233
FB_1111607_L15_129_3	fp01_01	1	1.233
FB_0411211_L1_PA	fp01_01	1	1.233
FB_0463467_L2_PA	fp01_01	1	1.233
FB_0411213_L1_PA	fp01_01	1	1.233
FB_1079977_L1_70_2	fp01_01	1	1.233
FB_0197414_L13_PA	fp01_01	1	1.233
FB_0411478_L1_PA	fp01_02	1	2.428
FB_0411310_L1_PA	fp01_02	1	2.428
FB_0411479_L1_PA	fp01_02	1	2.428
GD_02428_L1_PA	fp01_02	1	2.428
FB_0415516_L1_PA	fp01_02	1	2.428
FB_0464018_L2_PA	fp01_02	1	2.428
FB_0939067_L1_41_1	fp01_02	1	2.428
FB_1049454_L9_35_1	fp01_02	1	2.428
FB_0938919_L1_39_1	fp01_02	1	2.428
FB_0079751_L11_PA	fp01_02	1	2.428
FB_0411306_L1_PA	fp01_02	1	2.428
FB_0079718_L11_PA	fp01_02	1	2.428
FB_0673800_L6_PA	fp01_02	1	2.428
FB_1004093_L6_28_1	fp01_02	1	2.428
FB_0415504_L1_PA	fp01_02	1	2.428
FB_0411206_L1_PA	fp01_02	1	2.428
GD_00152_L1_42_1	fp01_02	1	2.428
FB_0463466_L2_PA	fp01_02	1	2.428
FB_0411491_L1_PA	fp01_02	1	2.428
FB_1114723_L3_103_3	fp01_02	1	2.428
FB_0976787_L3_60_1	fp01_03	1	3.683
FB_0411487_L1_PA	fp01_03	1	3.683
FB_1021083_L7_48_1	fp01_03	1	3.683
FB_1021088_L7_48_1	fp01_03	1	3.683
RB_4746500_L1_PA	fp01_03	1	3.683
FB_1021087_L7_48_1	fp01_03	1	3.683
FB_1114722_L3_103_3	fp01_03	1	3.683
FB_1114720_L3_103_3	fp01_03	1	3.683
FB_1099992_L7_66_2	fp01_03	1	3.683
FB_0411697_L1_PA	fp01_03	1	3.683
FB_1108214_L11_69_3	fp01_03	1	3.683
FB_0411698_L1_PA	fp01_04	1	4.639
FB_0939070_L1_41_1	fp01_04	1	4.639
FB_1049447_L9_35_1	fp01_04	1	4.639
FB_0079708_L11_PA	fp01_04	1	4.639
FB_0415487_L1_PA	fp01_04	1	4.639
FB_0079713_L11_PA	fp01_04	1	4.639
FB_0671554_L6_PA	fp01_04	1	4.639
FB_0079717_L11_PA	fp01_04	1	4.639

FB_1108208_L11_69_3	fp01_04	1	4.639
FB_0415526_L1_PA	fp01_04	1	4.639
FB_0416997_L1_PA	fp01_04	1	4.639
RB_8789324_L1_PA	fp01_05	1	5.489
FB_0418563_L1_PA	fp01_06	1	6.197
FB_0418565_L1_PA	fp01_06	1	6.197
RB_9351463_L1_PA	fp01_06	1	6.197
FB_0418576_L1_PA	fp01_06	1	6.197
FB_0418589_L1_PA	fp01_06	1	6.197
FB_0418586_L1_PA	fp01_06	1	6.197
FB_0417637_L1_PA	fp01_06	1	6.197
FB_0417197_L1_PA	fp01_07	1	7.469
FB_0417642_L1_PA	fp01_07	1	7.469
FB_0417203_L1_PA	fp01_07	1	7.469
FB_0417208_L1_PA	fp01_07	1	7.469
RB_11532952_L1_PA	fp01_07	1	7.469
FB_0948172_L2_21_1	fp01_07	1	7.469
RB_11489706_L1_PA	fp01_07	1	7.469
GD_02580_L1_PA	fp01_07	1	7.469
RB_9907051_L1_PA	fp01_07	1	7.469
FB_0417939_L1_PA	fp01_08	1	8.635
FB_0448220_L2_PA	fp01_08	1	8.635
FB_0448219_L2_PA	fp01_08	1	8.635
FB_0417946_L1_PA	fp01_08	1	8.635
FB_0419286_L1_PA	fp01_10	1	10.007
GD_01815_L1_PA	fp01_10	1	10.007
FB_0419289_L1_PA	fp01_10	1	10.007
RB_11508677_L1_PA	fp01_10	1	10.007
FB_0419285_L1_PA	fp01_10	1	10.007
GD_02334_L1_PA	fp01_12	1	12.788
FB_0420902_L1_PA	fp01_12	1	12.788
FB_0937902_L17_23_1	fp01_12	1	12.788
FB_0937887_L17_23_1	fp01_12	1	12.788
RB_12382050_L1_PA	fp01_12	1	12.788
FB_0420896_L1_PA	fp01_12	1	12.788
FB_0420924_L1_PA	fp01_12	1	12.788
RB_12194485_L1_PA	fp01_12	1	12.788
GD_00420_L1_PA	fp01_12	1	12.788
FB_0937905_L17_23_1	fp01_12	1	12.788
FB_0420895_L1_PA	fp01_12	1	12.788
FB_0420891_L1_PA	fp01_12	1	12.788
FB_0420898_L1_PA	fp01_12	1	12.788
FB_0420884_L1_PA	fp01_12	1	12.788
RB_13093630_L1_PA	fp01_12	1	12.788
RB_13068176_L1_PA	fp01_12	1	12.788
FB_0420881_L1_PA	fp01_12	1	12.788

FB_0419771_L1_PA	fp01_12	1	12.788
FB_0937906_L17_23_1	fp01_12	1	12.788
FB_0422079_L1_PA	fp01_14	1	14.661
FB_0420130_L1_PA	fp01_16	1	16.51
FB_0421614_L1_PA	fp01_16	1	16.51
FB_0421620_L1_PA	fp01_16	1	16.51
FB_0420150_L1_PA	fp01_16	1	16.51
FB_0421638_L1_PA	fp01_16	1	16.51
FB_0420170_L1_PA	fp01_16	1	16.51
FB_0420128_L1_PA	fp01_16	1	16.51
FB_0421625_L1_PA	fp01_16	1	16.51
FB_0421635_L1_PA	fp01_16	1	16.51
FB_0420129_L1_PA	fp01_16	1	16.51
RB_13037789_L1_PA	fp01_17	1	17.624
FB_0535512_L3_PA	fp01_17	1	17.624
RB_14001081_L1_PA	fp01_17	1	17.624
FB_0422078_L1_PA	fp01_18	1	18.454
GD_01889_L1_PA	fp01_19	1	19.195
FB_0941512_L1_49_1	fp01_20	1	20.703
FB_0941510_L1_49_1	fp01_20	1	20.703
FB_0941503_L1_49_1	fp01_20	1	20.703
FB_0423029_L1_PA	fp01_20	1	20.703
RB_14699213_L1_PA	fp01_20	1	20.703
FB_0423030_L1_PA	fp01_20	1	20.703
RB_14529153_L1_PA	fp01_20	1	20.703
RB_14657993_L1_PA	fp01_20	1	20.703
RB_14519247_L1_PA	fp01_20	1	20.703
FB_0423239_L1_PA	fp01_20	1	20.703
RB_14662213_L1_PA	fp01_20	1	20.703
FB_0423637_L1_PA	fp01_21	1	21.814
FB_0423631_L1_PA	fp01_21	1	21.814
RB_14798027_L1_49_1	fp01_21	1	21.814
FB_1080641_L1_74_2	fp01_21	1	21.814
RB_14836061_L1_PA	fp01_21	1	21.814
FB_1080640_L1_74_2	fp01_21	1	21.814
FB_0423617_L1_PA	fp01_21	1	21.814
FB_0423615_L1_PA	fp01_21	1	21.814
FB_0423612_L1_PA	fp01_21	1	21.814
FB_0423614_L1_PA	fp01_21	1	21.814
GD_01500_L1_49_1	fp01_21	1	21.814
FB_0425614_L1_PA	fp01_22	1	22.639
FB_0974460_L3_55_1	fp01_22	1	22.639
FB_0974443_L3_55_1	fp01_22	1	22.639
FB_0974449_L3_55_1	fp01_22	1	22.639
FB_0974448_L3_55_1	fp01_22	1	22.639
FB_0425793_L1_PA	fp01_22	1	22.639

FB_0424562_L1_PA	fp01_23	1	23.594
FB_0424563_L1_PA	fp01_23	1	23.594
FB_0424557_L1_PA	fp01_23	1	23.594
FB_0942380_L1_50_1	fp01_23	1	23.594
FB_0428723_L1_PA	fp01_24	1	24.409
RB_17869158_L1_PA	fp01_24	1	24.409
FB_1010747_L6_43_1	fp01_24	1	24.409
RB_17233926_L1_PA	fp01_24	1	24.409
FB_0427282_L1_PA	fp01_24	1	24.409
FB_0426232_L1_PA	fp01_24	1	24.409
GD_02538_L1_PA	fp01_24	1	24.409
RB_17236158_L1_PA	fp01_24	1	24.409
RB_17274400_L1_PA	fp01_24	1	24.409
FB_0425611_L1_PA	fp01_24	1	24.409
FB_0426240_L1_PA	fp01_24	1	24.409
RB_17245435_L1_PA	fp01_24	1	24.409
RB_17242135_L1_PA	fp01_24	1	24.409
RB_16467823_L1_PA	fp01_24	1	24.409
FB_0425610_L1_PA	fp01_24	1	24.409
RB_16501559_L1_50_1	fp01_24	1	24.409
FB_0426471_L1_PA	fp01_24	1	24.409
RB_17871172_L1_PA	fp01_24	1	24.409
FB_1113057_L1_89_3	fp01_25	1	25.386
FB_0426498_L1_PA	fp01_25	1	25.386
RB_17238705_L1_PA	fp01_25	1	25.386
RB_17882923_L1_PA	fp01_26	1	26.579
FB_0942698_L1_51_1	fp01_26	1	26.579
FB_0942990_L1_51_1	fp01_26	1	26.579
FB_0427296_L1_PA	fp01_26	1	26.579
RB_17878675_L1_PA	fp01_26	1	26.579
FB_0942992_L1_51_1	fp01_26	1	26.579
FB_0942974_L1_51_1	fp01_26	1	26.579
FB_0427463_L1_PA	fp01_26	1	26.579
RB_17921038_L1_PA	fp01_26	1	26.579
FB_0427287_L1_PA	fp01_26	1	26.579
FB_0497566_L2_PA	fp01_26	1	26.579
GD_01783_L1_PA	fp01_26	1	26.579
FB_0427473_L1_PA	fp01_26	1	26.579
RB_18067488_L1_PA	fp01_26	1	26.579
RB_18064090_L1_PA	fp01_26	1	26.579
FB_0427459_L1_PA	fp01_26	1	26.579
RB_18093819_L1_PA	fp01_26	1	26.579
FB_0427457_L1_PA	fp01_26	1	26.579
FB_0427467_L1_PA	fp01_27	1	27.382
GD_00183_L1_PA	fp01_28	1	28.241
FB_0428250_L1_PA	fp01_28	1	28.241

FB_0428721_L1_PA	fp01_28	1	28.241
FB_0428720_L1_PA	fp01_28	1	28.241
RB_18791535_L1_PA	fp01_28	1	28.241
RB_18794918_L1_PA	fp01_28	1	28.241
RB_18695269_L1_51_1	fp01_28	1	28.241
FB_0428214_L1_PA	fp01_28	1	28.241
FB_0429182_L1_PA	fp01_28	1	28.241
FB_0428254_L1_PA	fp01_28	1	28.241
FB_0429192_L1_PA	fp01_28	1	28.241
GD_02731_L1_PA	fp01_28	1	28.241
FB_0428220_L1_PA	fp01_28	1	28.241
FB_0428244_L1_PA	fp01_28	1	28.241
RB_18719697_L1_PA	fp01_28	1	28.241
RB_18714053_L1_PA	fp01_28	1	28.241
FB_0429288_L1_PA	fp01_28	1	28.241
FB_0429289_L1_PA	fp01_28	1	28.241
FB_0427306_L1_PA	fp01_28	1	28.241
RB_17912183_L1_PA	fp01_28	1	28.241
FB_0428219_L1_PA	fp01_28	1	28.241
FB_0428229_L1_PA	fp01_28	1	28.241
FB_0428726_L1_PA	fp01_28	1	28.241
FB_0429278_L1_PA	fp01_30	1	30.668
GD_02302_L1_PA	fp01_30	1	30.668
FB_0429982_L1_PA	fp01_30	1	30.668
RB_20951649_L1_PA	fp01_30	1	30.668
FB_0429994_L1_PA	fp01_30	1	30.668
FB_0429981_L1_PA	fp01_30	1	30.668
FB_0429988_L1_PA	fp01_30	1	30.668
FB_0429978_L1_PA	fp01_30	1	30.668
FB_0430001_L1_PA	fp01_30	1	30.668
RB_18831803_L1_PA	fp01_30	1	30.668
FB_0431160_L1_PA	fp01_30	1	30.668
FB_0431151_L1_PA	fp01_30	1	30.668
FB_0431150_L1_PA	fp01_30	1	30.668
FB_0943697_L1_56_1	fp01_30	1	30.668
FB_0943711_L1_56_1	fp01_30	1	30.668
FB_0428728_L1_PA	fp01_30	1	30.668
FB_0943665_L1_56_1	fp01_32	1	32.957
FB_0431171_L1_PA	fp01_32	1	32.957
FB_0431177_L1_PA	fp01_32	1	32.957
GD_00250_L1_PA	fp01_32	1	32.957
FB_0943667_L1_56_1	fp01_32	1	32.957
FB_0431170_L1_PA	fp01_32	1	32.957
FB_0943679_L1_56_1	fp01_32	1	32.957
FB_0943474_L1_56_1	fp01_33	1	33.825
FB_0943493_L1_56_1	fp01_34	1	34.406

FB_0943473_L1_56_1	fp01_34	1	34.406
GD_02518_L1_PA	fp01_34	1	34.406
FB_0943472_L1_56_1	fp01_34	1	34.406
FB_0430251_L1_PA	fp01_34	1	34.406
FB_0432803_L1_PA	fp01_34	1	34.406
FB_0430240_L1_PA	fp01_34	1	34.406
RB_21327009_L1_PA	fp01_34	1	34.406
FB_0430249_L1_PA	fp01_34	1	34.406
FB_0430261_L1_PA	fp01_34	1	34.406
RB_21289759_L1_56_1	fp01_35	1	35.4
FB_0432789_L1_PA	fp01_35	1	35.4
FB_0432792_L1_PA	fp01_35	1	35.4
RB_21291785_L1_56_1	fp01_35	1	35.4
FB_0430238_L1_PA	fp01_35	1	35.4
FB_0432796_L1_PA	fp01_35	1	35.4
FB_0433272_L1_PA	fp01_35	1	35.4
FB_0433287_L1_PA	fp01_36	1	36.797
FB_0433266_L1_PA	fp01_36	1	36.797
FB_0433269_L1_PA	fp01_36	1	36.797
RB_35787897_L1_PA	fp01_37	1	37.484
RB_26256055_L1_PA	fp01_39	1	39.64
FB_0250474_L14_PA	fp01_39	1	39.64
FB_0945108_L1_58_1	fp01_39	1	39.64
RB_26262680_L1_PA	fp01_39	1	39.64
GD_00674_L1_PA	fp01_39	1	39.64
FB_0250486_L14_PA	fp01_39	1	39.64
FB_0435779_L1_PA	fp01_39	1	39.64
FB_0945099_L1_58_1	fp01_39	1	39.64
FB_0945105_L1_58_1	fp01_39	1	39.64
FB_0945097_L1_58_1	fp01_39	1	39.64
FB_0437642_L1_PA	fp01_42	1	42.812
FB_0436482_L1_PA	fp01_42	1	42.812
FB_0437661_L1_PA	fp01_42	1	42.812
FB_0436495_L1_PA	fp01_42	1	42.812
GD_00107_L1_PA	fp01_42	1	42.812
RB_26734911_L1_PA	fp01_42	1	42.812
FB_0436919_L1_PA	fp01_42	1	42.812
FB_0436921_L1_PA	fp01_42	1	42.812
FB_0436465_L1_PA	fp01_42	1	42.812
FB_0436484_L1_PA	fp01_42	1	42.812
FB_0436926_L1_PA	fp01_42	1	42.812
FB_0436492_L1_PA	fp01_43	1	43.588
RB_26729463_L1_PA	fp01_43	1	43.588
FB_0436932_L1_PA	fp01_44	1	44.626
GD_01693_L1_PA	fp01_44	1	44.626
FB_0436943_L1_PA	fp01_44	1	44.626

FB_0436914_L1_PA	fp01_44	1	44.626
FB_0437669_L1_PA	fp01_44	1	44.626
FB_0436910_L1_PA	fp01_44	1	44.626
FB_0437687_L1_PA	fp01_44	1	44.626
FB_0437644_L1_PA	fp01_44	1	44.626
FB_0945847_L1_58_1	fp01_44	1	44.626
FB_0437666_L1_PA	fp01_44	1	44.626
FB_0436912_L1_PA	fp01_44	1	44.626
FB_0436917_L1_PA	fp01_44	1	44.626
FB_0437662_L1_PA	fp01_44	1	44.626
FB_0436927_L1_PA	fp01_44	1	44.626
RB_27060614_L1_PA	fp01_44	1	44.626
RB_27095198_L1_PA	fp01_44	1	44.626
FB_0945909_L1_60_1	fp01_47	1	47.734
FB_0945897_L1_60_1	fp01_47	1	47.734
FB_0438227_L1_PA	fp01_47	1	47.734
FB_0438222_L1_PA	fp01_47	1	47.734
FB_0945907_L1_60_1	fp01_47	1	47.734
FB_0438220_L1_PA	fp01_47	1	47.734
FB_0438233_L1_PA	fp01_47	1	47.734
FB_0945902_L1_60_1	fp01_47	1	47.734
FB_0438218_L1_PA	fp01_47	1	47.734
FB_0442048_L1_PA	fp01_48	1	48.294
FB_0439106_L1_PA	fp01_49	1	49.556
RB_29167924_L1_PA	fp01_49	1	49.556
RB_29120475_L1_PA	fp01_49	1	49.556
FB_0439095_L1_PA	fp01_49	1	49.556
RB_30246914_L1_PA	fp01_49	1	49.556
RB_29128835_L1_PA	fp01_49	1	49.556
FB_0439076_L1_PA	fp01_49	1	49.556
FB_0441015_L1_PA	fp01_49	1	49.556
RB_29163276_L1_PA	fp01_49	1	49.556
FB_0439065_L1_PA	fp01_49	1	49.556
FB_0440721_L1_PA	fp01_49	1	49.556
FB_0439066_L1_PA	fp01_49	1	49.556
FB_0440719_L1_PA	fp01_49	1	49.556
FB_0439071_L1_PA	fp01_49	1	49.556
FB_0439059_L1_PA	fp01_49	1	49.556
FB_0441010_L1_PA	fp01_49	1	49.556
FB_0441006_L1_PA	fp01_49	1	49.556
GD_00782_L1_PA	fp01_49	1	49.556
RB_30217061_L1_82_2	fp01_49	1	49.556
FB_0439075_L1_PA	fp01_49	1	49.556
FB_0439088_L1_PA	fp01_49	1	49.556
FB_0442342_L1_PA	fp01_51	1	51.504
FB_0442329_L1_PA	fp01_51	1	51.504

RB_31659789_L1_PA	fp01_51	1	51.504
FB_0441428_L1_PA	fp01_52	1	52.51
MdSOC1.like_L1_PA	fp01_52	1	52.51
FB_0440723_L1_PA	fp01_52	1	52.51
FB_0442482_L1_PA	fp01_53	1	53.854
FB_0442972_L1_PA	fp01_54	1	54.711
FB_0444691_L1_PA	fp01_54	1	54.711
FB_0444702_L1_PA	fp01_54	1	54.711
FB_0441434_L1_PA	fp01_54	1	54.711
RB_31359482_L1_PA	fp01_54	1	54.711
RB_30590177_L1_PA	fp01_54	1	54.711
RB_31736288_L1_PA	fp01_54	1	54.711
RB_31681861_L1_PA	fp01_54	1	54.711
RB_31657598_L1_PA	fp01_55	1	55.492
MdBFTb_L1_PA	fp01_55	1	55.492
FB_0442475_L1_PA	fp01_55	1	55.492
RB_31626705_L1_PA	fp01_55	1	55.492
RB_31642444_L1_PA	fp01_55	1	55.492
RB_31736133_L1_PA	fp01_55	1	55.492
FB_0442473_L1_PA	fp01_55	1	55.492
FB_0442481_L1_PA	fp01_55	1	55.492
RB_31531477_L1_PA	fp01_55	1	55.492
RB_31945251_L1_PA	fp01_55	1	55.492
FB_0444437_L1_PA	fp01_55	1	55.492
FB_0444432_L1_PA	fp01_55	1	55.492
FB_0442968_L1_PA	fp01_55	1	55.492
FB_0444693_L1_PA	fp01_55	1	55.492
FB_0444714_L1_PA	fp01_56	1	56.527
FB_0946989_L1_65_1	fp01_56	1	56.527
RB_33663934_L1_PA	fp01_56	1	56.527
FB_0444707_L1_PA	fp01_56	1	56.527
FB_0946997_L1_65_1	fp01_57	1	57.229
FB_0946984_L1_65_1	fp01_57	1	57.229
FB_0445359_L1_PA	fp01_57	1	57.229
FB_0445366_L1_PA	fp01_57	1	57.229
FB_0947004_L1_65_1	fp01_57	1	57.229
FB_0445358_L1_PA	fp01_57	1	57.229
FB_1082254_L1_84_2	fp01_57	1	57.229
FB_0445360_L1_PA	fp01_57	1	57.229
FB_0946982_L1_65_1	fp01_57	1	57.229
FB_0442963_L1_PA	fp01_57	1	57.229
RB_31941783_L1_PA	fp01_57	1	57.229
FB_0442970_L1_PA	fp01_57	1	57.229
RB_35422934_L1_PA	fp01_58	1	58.44
FB_0446904_L1_PA	fp01_58	1	58.44
FB_0445367_L1_PA	fp01_58	1	58.44

RB_34439805_L1_PA	fp01_59	1	59.632
FB_0445938_L1_PA	fp01_59	1	59.632
RB_34421998_L1_PA	fp01_59	1	59.632
GD_02371_L1_PA	fp01_59	1	59.632
FB_0447053_L1_PA	fp01_59	1	59.632
FB_0445933_L1_PA	fp01_59	1	59.632
FB_0445931_L1_PA	fp01_59	1	59.632
FB_0445935_L1_PA	fp01_59	1	59.632
GD_00495_L1_PA	fp01_59	1	59.632
RB_35251020_L1_PA	fp01_59	1	59.632
FB_0445939_L1_PA	fp01_59	1	59.632
FB_0447065_L1_PA	fp01_59	1	59.632
FB_0447033_L1_PA	fp01_59	1	59.632
FB_0447050_L1_PA	fp01_59	1	59.632
FB_0446917_L1_PA	fp01_59	1	59.632
GD_00583_L1_PA	fp01_60	1	60.619
FB_1082423_L1_84_2	fp01_60	1	60.619
FB_0947636_L1_65_1	fp01_60	1	60.619
RB_35468899_L1_65_1	fp01_60	1	60.619
FB_1082449_L1_84_2	fp01_60	1	60.619
FB_0447591_L1_PA	fp01_60	1	60.619
RB_35199556_L1_PA	fp01_61	1	61.407
FB_0447040_L1_PA	fp01_61	1	61.407
RB_35195077_L1_PA	fp01_61	1	61.407
RB_35202609_L1_PA	fp01_61	1	61.407
RB_35192538_L1_PA	fp01_61	1	61.407
FB_0446920_L1_PA	fp01_61	1	61.407
FB_0446921_L1_PA	fp01_61	1	61.407
FB_1082438_L1_84_2	fp01_61	1	61.407
FB_1082439_L1_84_2	fp01_61	1	61.407
FB_0447351_L1_PA	fp01_61	1	61.407
GD_02092_L1_PA	fp01_61	1	61.407
FB_1082420_L1_84_2	fp01_62	1	62.085
FB_0447581_L1_PA	fp01_62	1	62.085
FB_1082431_L1_84_2	fp01_62	1	62.085
FB_1082432_L1_84_2	fp01_62	1	62.085
FB_0947613_L1_65_1	fp01_62	1	62.085
FB_1082453_L1_84_2	fp01_62	1	62.085
FB_0947619_L1_65_1	fp01_62	1	62.085
FB_0947842_L1_65_1	fp01_63	1	63.47
FB_0947830_L1_65_1	fp01_63	1	63.47
FB_0947841_L1_65_1	fp01_63	1	63.47
FB_0947835_L1_65_1	fp01_63	1	63.47
FB_0947848_L1_65_1	fp01_63	1	63.47
FB_0948090_L1_65_1	fp01_63	1	63.47
RB_35939850_L1_65_1	fp01_63	1	63.47

FB_0448111_L1_PA	fp01_63	1	63.47
FB_0947856_L1_65_1	fp01_63	1	63.47
FB_0504009_L2_PA	fp02_00	2	0.502
FB_0505185_L2_PA	fp02_00	2	0.502
FB_0505183_L2_PA	fp02_00	2	0.502
RB_39505783_L2_PA	fp02_00	2	0.502
FB_0503622_L2_PA	fp02_00	2	0.502
RB_39010781_L2_PA	fp02_00	2	0.502
RB_40079809_L2_PA	fp02_00	2	0.502
FB_0503613_L2_PA	fp02_00	2	0.502
FB_0504011_L2_PA	fp02_00	2	0.502
RB_39497984_L2_PA	fp02_00	2	0.502
RB_39014029_L2_PA	fp02_00	2	0.502
FB_0503614_L2_PA	fp02_00	2	0.502
RB_39526012_L2_38_1	fp02_00	2	0.502
FB_0503998_L2_PA	fp02_00	2	0.502
RB_40092827_L2_PA	fp02_00	2	0.502
GD_00167_L2_38_1	fp02_00	2	0.502
GD_02535_L2_PA	fp02_00	2	0.502
FB_0504436_L2_PA	fp02_00	2	0.502
FB_0504437_L2_PA	fp02_00	2	0.502
RB_40085225_L2_PA	fp02_00	2	0.502
FB_0503627_L2_PA	fp02_00	2	0.502
FB_0503334_L2_PA	fp02_00	2	0.502
FB_0505187_L2_PA	fp02_00	2	0.502
FB_0503983_L2_PA	fp02_00	2	0.502
FB_0504444_L2_PA	fp02_00	2	0.502
FB_0503611_L2_PA	fp02_00	2	0.502
FB_0502932_L2_PA	fp02_00	2	0.502
FB_0503640_L2_PA	fp02_01	2	1.359
FB_0503332_L2_PA	fp02_01	2	1.359
FB_0503322_L2_PA	fp02_01	2	1.359
RB_38921890_L2_PA	fp02_02	2	2.417
FB_0502410_L2_PA	fp02_02	2	2.417
RB_39523918_L2_38_1	fp02_03	2	3.471
FB_1087149_L2_53_2	fp02_03	2	3.471
FB_1087142_L2_53_2	fp02_03	2	3.471
RB_39549680_L2_PA	fp02_03	2	3.471
FB_0502949_L2_PA	fp02_03	2	3.471
RB_39576068_L2_PA	fp02_03	2	3.471
FB_0502954_L2_PA	fp02_03	2	3.471
FB_0502948_L2_PA	fp02_03	2	3.471
RB_39558990_L2_PA	fp02_03	2	3.471
RB_39271679_L2_PA	fp02_03	2	3.471
RB_36448217_L2_PA	fp02_03	2	3.471
FB_1087157_L2_53_2	fp02_04	2	4.327

GD_00624_L2_37_1	fp02_04	2	4.327
FB_0502952_L2_PA	fp02_04	2	4.327
FB_0503323_L2_PA	fp02_04	2	4.327
FB_0503335_L2_PA	fp02_04	2	4.327
FB_0502933_L2_PA	fp02_04	2	4.327
RB_38008622_L2_PA	fp02_04	2	4.327
FB_0503320_L2_PA	fp02_04	2	4.327
FB_0502408_L2_PA	fp02_04	2	4.327
FB_0502400_L2_PA	fp02_04	2	4.327
RB_36217156_L2_PA	fp02_04	2	4.327
FB_0501816_L2_PA	fp02_04	2	4.327
FB_0499635_L2_PA	fp02_04	2	4.327
FB_0502401_L2_PA	fp02_04	2	4.327
GD_00914_L2_53_2	fp02_04	2	4.327
FB_1063207_L13_50_2	fp02_04	2	4.327
RB_36492148_L2_PA	fp02_04	2	4.327
RB_38977655_L2_PA	fp02_04	2	4.327
FB_0499624_L2_PA	fp02_04	2	4.327
GD_00669_L2_PA	fp02_04	2	4.327
GD_10007_L2_PA	fp02_04	2	4.327
RB_36473196_L2_PA	fp02_04	2	4.327
FB_0499629_L2_PA	fp02_04	2	4.327
RB_36465694_L2_PA	fp02_04	2	4.327
FB_0501809_L2_PA	fp02_04	2	4.327
FB_0214511_L14_PA	fp02_04	2	4.327
FB_0499610_L2_PA	fp02_04	2	4.327
RB_36462247_L2_PA	fp02_04	2	4.327
FB_1063203_L13_50_2	fp02_04	2	4.327
RB_37999186_L2_PA	fp02_04	2	4.327
FB_0501810_L2_PA	fp02_04	2	4.327
RB_35446282_L2_PA	fp02_04	2	4.327
RB_36178435_L2_PA	fp02_04	2	4.327
FB_0501806_L2_PA	fp02_04	2	4.327
RB_36357685_L2_PA	fp02_04	2	4.327
FB_0501805_L2_PA	fp02_04	2	4.327
RB_36421423_L2_PA	fp02_04	2	4.327
FB_0498500_L2_PA	fp02_04	2	4.327
FB_0498491_L2_PA	fp02_04	2	4.327
FB_0498492_L2_PA	fp02_04	2	4.327
GD_00267_L2_PA	fp02_04	2	4.327
FB_0498497_L2_PA	fp02_04	2	4.327
RB_36362741_L2_PA	fp02_04	2	4.327
FB_1086648_L2_52_2	fp02_05	2	5.462
GD_01322_L2_PA	fp02_05	2	5.462
RB_38924310_L2_PA	fp02_05	2	5.462
FB_1063199_L13_50_2	fp02_06	2	6.146

RB_36329748_L2_PA	fp02_06	2	6.146
RB_35487462_L2_PA	fp02_07	2	7.422
RB_36185368_L2_PA	fp02_07	2	7.422
RB_36182763_L2_PA	fp02_08	2	8.664
RB_35490042_L2_PA	fp02_09	2	9.379
FB_0499639_L2_PA	fp02_09	2	9.379
RB_34225113_L2_PA	fp02_09	2	9.379
RB_34194147_L2_PA	fp02_10	2	10.541
FB_0961869_L2_36_1	fp02_10	2	10.541
FB_0961868_L2_36_1	fp02_10	2	10.541
FB_0497066_L2_PA	fp02_10	2	10.541
FB_0961854_L2_36_1	fp02_10	2	10.541
FB_0497077_L2_PA	fp02_10	2	10.541
FB_0497074_L2_PA	fp02_10	2	10.541
FB_0496348_L2_PA	fp02_10	2	10.541
FB_0496336_L2_PA	fp02_11	2	11.389
FB_0496343_L2_PA	fp02_11	2	11.389
FB_0496340_L2_PA	fp02_11	2	11.389
FB_0495949_L2_PA	fp02_11	2	11.389
FB_0495964_L2_PA	fp02_11	2	11.389
GD_01349_L2_PA	fp02_11	2	11.389
FB_0495955_L2_PA	fp02_11	2	11.389
FB_0495942_L2_PA	fp02_11	2	11.389
FB_0495984_L2_PA	fp02_11	2	11.389
FB_0496337_L2_PA	fp02_11	2	11.389
FB_0961817_L2_36_1	fp02_11	2	11.389
FB_0495972_L2_PA	fp02_11	2	11.389
FB_0495951_L2_PA	fp02_11	2	11.389
RB_33896449_L2_PA	fp02_11	2	11.389
FB_0659636_L6_PA	fp02_11	2	11.389
FB_0497067_L2_PA	fp02_11	2	11.389
FB_0496338_L2_PA	fp02_11	2	11.389
RB_34231106_L2_PA	fp02_11	2	11.389
FB_0961812_L2_36_1	fp02_11	2	11.389
RB_34233785_L2_PA	fp02_11	2	11.389
RB_35458744_L2_PA	fp02_11	2	11.389
FB_0659593_L6_PA	fp02_11	2	11.389
FB_0659583_L6_PA	fp02_11	2	11.389
FB_0659606_L6_PA	fp02_11	2	11.389
FB_0659635_L6_PA	fp02_11	2	11.389
FB_0659619_L6_PA	fp02_11	2	11.389
FB_0961624_L2_36_1	fp02_11	2	11.389
FB_0495649_L2_PA	fp02_11	2	11.389
RB_33891250_L2_PA	fp02_11	2	11.389
FB_0495616_L2_PA	fp02_11	2	11.389
FB_0961629_L2_36_1	fp02_11	2	11.389

FB_0659592_L6_PA	fp02_11	2	11.389
FB_0961850_L2_36_1	fp02_12	2	12.79
FB_0497082_L2_PA	fp02_12	2	12.79
FB_0495625_L2_PA	fp02_12	2	12.79
RB_34228187_L2_PA	fp02_12	2	12.79
FB_0961621_L2_36_1	fp02_12	2	12.79
FB_1086523_L2_51_2	fp02_12	2	12.79
FB_1086530_L2_51_2	fp02_12	2	12.79
FB_0961619_L2_36_1	fp02_12	2	12.79
FB_1086525_L2_51_2	fp02_12	2	12.79
FB_0961638_L2_36_1	fp02_12	2	12.79
FB_0961628_L2_36_1	fp02_13	2	13.956
FB_0961305_L2_36_1	fp02_14	2	14.166
RB_32997136_L2_PA	fp02_14	2	14.166
FB_0495005_L2_PA	fp02_14	2	14.166
FB_0495006_L2_PA	fp02_14	2	14.166
FB_0495018_L2_PA	fp02_14	2	14.166
GD_02223_L2_PA	fp02_14	2	14.166
FB_0961298_L2_36_1	fp02_14	2	14.166
FB_0494999_L2_PA	fp02_14	2	14.166
RB_33257933_L2_PA	fp02_14	2	14.166
Rvi2_region23_R366	fp02_14	2	14.166
GD_00629_L2_PA	fp02_14	2	14.166
FB_0495016_L2_PA	fp02_14	2	14.166
RB_33262034_L2_PA	fp02_14	2	14.166
FB_0495014_L2_PA	fp02_14	2	14.166
FB_0495004_L2_PA	fp02_14	2	14.166
FB_0494250_L2_PA	fp02_14	2	14.166
FB_0495002_L2_PA	fp02_14	2	14.166
Rvi2_reg27.2_M133b	fp02_14	2	14.166
RB_33873171_L2_PA	fp02_14	2	14.166
FB_0961632_L2_36_1	fp02_15	2	15.861
FB_0659613_L6_PA	fp02_15	2	15.861
FB_0961635_L2_36_1	fp02_15	2	15.861
FB_0493186_L2_PA	fp02_15	2	15.861
FB_0493175_L2_PA	fp02_15	2	15.861
FB_0493179_L2_PA	fp02_15	2	15.861
FB_0493180_L2_PA	fp02_15	2	15.861
FB_0493532_L2_PA	fp02_15	2	15.861
FB_0493534_L2_PA	fp02_15	2	15.861
FB_0493193_L2_PA	fp02_15	2	15.861
FB_0493196_L2_PA	fp02_15	2	15.861
FB_0960786_L2_36_1	fp02_15	2	15.861
Rvi2_region23_R468	fp02_16	2	16.468
FB_0492193_L2_PA	fp02_16	2	16.468
RB_33002490_L2_PA	fp02_16	2	16.468

RB_30846581_L2_PA	fp02_16	2	16.468
RB_32999908_L2_PA	fp02_16	2	16.468
GD_01251_L2_PA	fp02_17	2	17.463
RB_32121227_L2_PA	fp02_17	2	17.463
RB_33272338_L2_51_2	fp02_17	2	17.463
FB_0493526_L2_PA	fp02_17	2	17.463
RB_32205884_L2_PA	fp02_17	2	17.463
RB_32203002_L2_PA	fp02_17	2	17.463
FB_0961303_L2_36_1	fp02_17	2	17.463
FB_0493182_L2_PA	fp02_18	2	18.681
Rvi2_region21_Y693	fp02_18	2	18.681
FB_0492220_L2_PA	fp02_18	2	18.681
FB_0492194_L2_PA	fp02_18	2	18.681
Rvi2_region41_Y341	fp02_18	2	18.681
RB_32123551_L2_PA	fp02_18	2	18.681
RB_32171222_L2_PA	fp02_18	2	18.681
FB_0492190_L2_PA	fp02_19	2	19.401
FB_0492225_L2_PA	fp02_19	2	19.401
FB_0492208_L2_PA	fp02_19	2	19.401
RB_32854243_L2_PA	fp02_19	2	19.401
FB_0493192_L2_PA	fp02_19	2	19.401
RB_32854273_L2_PA	fp02_19	2	19.401
RB_32422332_L2_PA	fp02_19	2	19.401
FB_0492218_L2_PA	fp02_19	2	19.401
FB_0487282_L2_PA	fp02_19	2	19.401
FB_0493535_L2_PA	fp02_19	2	19.401
FB_0487049_L2_PA	fp02_19	2	19.401
FB_0490927_L2_PA	fp02_19	2	19.401
FB_0493187_L2_PA	fp02_20	2	20.597
FB_0489674_L2_PA	fp02_20	2	20.597
FB_0489789_L2_PA	fp02_20	2	20.597
RB_29827721_L2_PA	fp02_20	2	20.597
RB_30846235_L2_PA	fp02_20	2	20.597
GD_01935_L2_PA	fp02_20	2	20.597
FB_0486161_L2_PA	fp02_20	2	20.597
FB_0667266_L6_PA	fp02_20	2	20.597
FB_0960305_L2_36_1	fp02_20	2	20.597
RB_27286399_L2_PA	fp02_20	2	20.597
RB_26390694_L2_PA	fp02_20	2	20.597
FB_1092108_L4_33_2	fp02_20	2	20.597
FB_0487276_L2_PA	fp02_20	2	20.597
FB_0487281_L2_PA	fp02_20	2	20.597
FB_0487045_L2_PA	fp02_20	2	20.597
FB_0489774_L2_PA	fp02_20	2	20.597
FB_0486156_L2_PA	fp02_20	2	20.597
RB_27306234_L2_PA	fp02_20	2	20.597

RB_30846663_L2_PA	fp02_20	2	20.597
RB_30846147_L2_PA	fp02_20	2	20.597
RB_32168123_L2_PA	fp02_20	2	20.597
RB_30314379_L2_PA	fp02_20	2	20.597
FB_0489772_L2_PA	fp02_20	2	20.597
RB_30299235_L2_PA	fp02_20	2	20.597
FB_0490067_L2_PA	fp02_20	2	20.597
FB_0959076_L2_34_1	fp02_20	2	20.597
FB_0487035_L2_PA	fp02_20	2	20.597
RB_27303643_L2_PA	fp02_20	2	20.597
FB_0927569_L16_30_1	fp02_20	2	20.597
FB_1092101_L4_33_2	fp02_20	2	20.597
RB_27298641_L2_PA	fp02_20	2	20.597
FB_1092109_L4_33_2	fp02_20	2	20.597
RB_27335209_L2_PA	fp02_20	2	20.597
FB_0490931_L2_PA	fp02_20	2	20.597
FB_0490930_L2_PA	fp02_20	2	20.597
RB_30846189_L2_PA	fp02_20	2	20.597
RB_30377236_L2_PA	fp02_20	2	20.597
FB_1093755_L5_63_2	fp02_21	2	21.444
FB_0667273_L6_PA	fp02_21	2	21.444
FB_0490928_L2_PA	fp02_21	2	21.444
FB_0487284_L2_PA	fp02_21	2	21.444
FB_0487268_L2_PA	fp02_21	2	21.444
RB_27281402_L2_PA	fp02_21	2	21.444
FB_0960065_L2_36_1	fp02_21	2	21.444
RB_25674246_L2_PA	fp02_21	2	21.444
FB_0490085_L2_PA	fp02_21	2	21.444
FB_0486750_L2_PA	fp02_21	2	21.444
FB_0667267_L6_PA	fp02_21	2	21.444
FB_1093754_L5_63_2	fp02_21	2	21.444
FB_0132758_L12_PA	fp02_21	2	21.444
RB_25680654_L2_PA	fp02_21	2	21.444
FB_0667258_L6_PA	fp02_21	2	21.444
RB_26342765_L2_PA	fp02_21	2	21.444
FB_0958922_L2_34_1	fp02_21	2	21.444
FB_0484398_L2_PA	fp02_21	2	21.444
RB_24747820_L2_PA	fp02_21	2	21.444
RB_24792350_L2_PA	fp02_21	2	21.444
FB_0485376_L2_PA	fp02_21	2	21.444
RB_24744626_L2_PA	fp02_21	2	21.444
FB_0483284_L2_PA	fp02_22	2	22.688
FB_0487500_L2_PA	fp02_22	2	22.688
FB_0487487_L2_PA	fp02_22	2	22.688
FB_1093760_L5_63_2	fp02_22	2	22.688
FB_0487486_L2_PA	fp02_22	2	22.688

FB_0487488_L2_PA	fp02_22	2	22.688
GD_02155_L2_32_1	fp02_22	2	22.688
FB_1093757_L5_63_2	fp02_22	2	22.688
FB_0485385_L2_PA	fp02_22	2	22.688
GD_02376_L2_PA	fp02_22	2	22.688
FB_0958353_L2_32_1	fp02_22	2	22.688
FB_0485378_L2_PA	fp02_22	2	22.688
FB_0958615_L2_32_1	fp02_22	2	22.688
FB_0958357_L2_32_1	fp02_23	2	23.292
FB_0489795_L2_PA	fp02_23	2	23.292
FB_0487051_L2_PA	fp02_23	2	23.292
FB_0487042_L2_PA	fp02_23	2	23.292
FB_0489775_L2_PA	fp02_23	2	23.292
RB_30305383_L2_PA	fp02_23	2	23.292
FB_0487044_L2_PA	fp02_23	2	23.292
FB_0483064_L2_PA	fp02_23	2	23.292
FB_0487462_L2_PA	fp02_23	2	23.292
FB_0484405_L2_PA	fp02_23	2	23.292
RB_24790175_L2_PA	fp02_24	2	24.65
FB_0482226_L2_PA	fp02_24	2	24.65
FB_0481284_L2_PA	fp02_24	2	24.65
GD_00833_L2_PA	fp02_24	2	24.65
RB_24821574_L2_PA	fp02_25	2	25.252
RB_19301602_L2_PA	fp02_26	2	26.461
RB_19428938_L2_PA	fp02_26	2	26.461
RB_18843281_L2_PA	fp02_26	2	26.461
FB_0476519_L2_PA	fp02_27	2	27.393
RB_18834678_L2_PA	fp02_27	2	27.393
RB_18837099_L2_PA	fp02_27	2	27.393
RB_19258873_L2_PA	fp02_27	2	27.393
FB_0477203_L2_PA	fp02_27	2	27.393
RB_19261463_L2_PA	fp02_27	2	27.393
FB_0480282_L2_PA	fp02_27	2	27.393
FB_0477927_L2_PA	fp02_27	2	27.393
RB_18882549_L2_PA	fp02_27	2	27.393
RB_19241986_L2_PA	fp02_27	2	27.393
FB_0957124_L2_28_1	fp02_27	2	27.393
RB_18848241_L2_PA	fp02_27	2	27.393
RB_19239039_L2_PA	fp02_27	2	27.393
FB_0136084_L12_PA	fp02_27	2	27.393
FB_0996172_L5_43_1	fp02_27	2	27.393
FB_0480275_L2_PA	fp02_27	2	27.393
FB_0477914_L2_PA	fp02_27	2	27.393
FB_0477932_L2_PA	fp02_27	2	27.393
FB_0136078_L12_PA	fp02_28	2	28.597
FB_0480732_L2_PA	fp02_28	2	28.597

RB_23088754_L2_PA	fp02_28	2	28.597
FB_0480287_L2_PA	fp02_28	2	28.597
FB_0996173_L5_43_1	fp02_28	2	28.597
FB_0481309_L2_PA	fp02_28	2	28.597
FB_0482224_L2_PA	fp02_28	2	28.597
FB_0483831_L2_PA	fp02_28	2	28.597
FB_0482208_L2_PA	fp02_28	2	28.597
FB_0481285_L2_PA	fp02_28	2	28.597
FB_0481315_L2_PA	fp02_28	2	28.597
FB_0482230_L2_PA	fp02_28	2	28.597
FB_0481312_L2_PA	fp02_28	2	28.597
RB_23078001_L2_PA	fp02_28	2	28.597
FB_0481306_L2_PA	fp02_28	2	28.597
FB_0481301_L2_PA	fp02_28	2	28.597
RB_19606107_L2_PA	fp02_29	2	29.193
FB_0476526_L2_PA	fp02_30	2	30.664
FB_0957128_L2_28_1	fp02_30	2	30.664
FB_0424924_L1_PA	fp02_30	2	30.664
FB_0483840_L2_PA	fp02_30	2	30.664
FB_0623601_L5_PA	fp02_30	2	30.664
FB_0480754_L2_PA	fp02_30	2	30.664
FB_0480264_L2_PA	fp02_30	2	30.664
FB_0480741_L2_PA	fp02_30	2	30.664
FB_0996171_L5_43_1	fp02_30	2	30.664
FB_0480730_L2_PA	fp02_30	2	30.664
GD_02076_L2_30_1	fp02_30	2	30.664
FB_0482647_L2_PA	fp02_30	2	30.664
FB_0480259_L2_PA	fp02_30	2	30.664
FB_0996182_L5_43_1	fp02_30	2	30.664
RB_21105289_L2_PA	fp02_30	2	30.664
RB_18005035_L2_PA	fp02_30	2	30.664
RB_18004558_L2_PA	fp02_30	2	30.664
FB_0480759_L2_PA	fp02_30	2	30.664
FB_0482662_L2_PA	fp02_30	2	30.664
FB_0480288_L2_PA	fp02_30	2	30.664
FB_0482637_L2_PA	fp02_30	2	30.664
RB_24104888_L2_PA	fp02_31	2	31.62
FB_0475668_L2_PA	fp02_31	2	31.62
RB_18003967_L2_PA	fp02_31	2	31.62
FB_0956985_L2_27_1	fp02_32	2	32.589
RB_17179555_L2_PA	fp02_32	2	32.589
FB_0474195_L2_PA	fp02_32	2	32.589
FB_0474201_L2_PA	fp02_32	2	32.589
FB_0475051_L2_PA	fp02_32	2	32.589
FB_0474199_L2_PA	fp02_32	2	32.589
FB_0474197_L2_PA	fp02_32	2	32.589

FB_0474198_L2_PA	fp02_32	2	32.589
FB_0474178_L2_PA	fp02_32	2	32.589
RB_17179731_L2_PA	fp02_32	2	32.589
FB_0136086_L12_PA	fp02_33	2	33.248
FB_0136067_L12_PA	fp02_33	2	33.248
RB_15214773_L2_PA	fp02_33	2	33.248
FB_0471072_L2_PA	fp02_33	2	33.248
FB_0471602_L2_PA	fp02_33	2	33.248
FB_0206557_L13_PA	fp02_33	2	33.248
FB_0956215_L2_27_1	fp02_33	2	33.248
FB_0472612_L2_PA	fp02_33	2	33.248
FB_0473495_L2_PA	fp02_33	2	33.248
RB_16803165_L2_PA	fp02_33	2	33.248
FB_0473493_L2_PA	fp02_33	2	33.248
RB_16174412_L2_PA	fp02_33	2	33.248
FB_0474192_L2_PA	fp02_33	2	33.248
FB_0472023_L2_PA	fp02_33	2	33.248
FB_0471071_L2_PA	fp02_33	2	33.248
FB_0888754_L14_37_1	fp02_33	2	33.248
FB_0476523_L2_PA	fp02_33	2	33.248
FB_0474189_L2_PA	fp02_33	2	33.248
FB_0888731_L14_37_1	fp02_33	2	33.248
FB_0473496_L2_PA	fp02_33	2	33.248
GD_00222_L2_PA	fp02_33	2	33.248
FB_0473506_L2_PA	fp02_33	2	33.248
FB_0472605_L2_PA	fp02_33	2	33.248
RB_15273312_L2_PA	fp02_33	2	33.248
FB_0888741_L14_37_1	fp02_33	2	33.248
FB_0206551_L13_PA	fp02_33	2	33.248
FB_0475647_L2_PA	fp02_33	2	33.248
FB_0474177_L2_PA	fp02_33	2	33.248
FB_0424926_L1_PA	fp02_33	2	33.248
FB_0477200_L2_PA	fp02_33	2	33.248
FB_0476515_L2_PA	fp02_33	2	33.248
FB_0477197_L2_PA	fp02_33	2	33.248
FB_0471065_L2_PA	fp02_33	2	33.248
FB_0472604_L2_PA	fp02_33	2	33.248
FB_0471604_L2_PA	fp02_33	2	33.248
FB_0471077_L2_PA	fp02_33	2	33.248
FB_0475059_L2_PA	fp02_33	2	33.248
FB_0476291_L2_PA	fp02_33	2	33.248
FB_0475045_L2_PA	fp02_33	2	33.248
MdCCD7a_L2_PA	fp02_33	2	33.248
FB_0206556_L13_PA	fp02_33	2	33.248
FB_0471069_L2_PA	fp02_33	2	33.248
FB_0206553_L13_PA	fp02_33	2	33.248

FB_0472022_L2_PA	fp02_33	2	33.248
FB_0477201_L2_PA	fp02_33	2	33.248
FB_0956216_L2_27_1	fp02_33	2	33.248
FB_0888730_L14_37_1	fp02_33	2	33.248
GD_00934_L2_PA	fp02_33	2	33.248
FB_0957301_L2_28_1	fp02_33	2	33.248
FB_0477196_L2_PA	fp02_33	2	33.248
FB_0888770_L14_37_1	fp02_33	2	33.248
RB_14588942_L2_PA	fp02_33	2	33.248
RB_15184323_L2_PA	fp02_33	2	33.248
RB_15211202_L2_PA	fp02_34	2	34.364
FB_0468919_L2_PA	fp02_34	2	34.364
FB_0470620_L2_PA	fp02_34	2	34.364
FB_0470613_L2_PA	fp02_34	2	34.364
FB_0470622_L2_PA	fp02_34	2	34.364
FB_0470609_L2_PA	fp02_34	2	34.364
FB_0469793_L2_PA	fp02_34	2	34.364
FB_0470616_L2_PA	fp02_34	2	34.364
GD_00002_L2_PA	fp02_34	2	34.364
FB_0470617_L2_PA	fp02_34	2	34.364
RB_16177297_L2_PA	fp02_34	2	34.364
FB_0471591_L2_PA	fp02_34	2	34.364
RB_15230080_L2_PA	fp02_35	2	35.466
FB_0472029_L2_PA	fp02_35	2	35.466
RB_14512918_L2_PA	fp02_35	2	35.466
FB_0469356_L2_PA	fp02_35	2	35.466
FB_0471598_L2_PA	fp02_35	2	35.466
FB_0473501_L2_PA	fp02_35	2	35.466
FB_0471603_L2_PA	fp02_36	2	36.288
RB_14599407_L2_PA	fp02_36	2	36.288
FB_0469788_L2_PA	fp02_36	2	36.288
FB_0469345_L2_PA	fp02_36	2	36.288
RB_13921344_L2_PA	fp02_37	2	37.233
FB_1084878_L2_42_2	fp02_37	2	37.233
FB_0469786_L2_PA	fp02_37	2	37.233
FB_0469340_L2_PA	fp02_37	2	37.233
RB_14484409_L2_PA	fp02_37	2	37.233
FB_0469373_L2_PA	fp02_38	2	38.269
GD_00174_L2_26_1	fp02_38	2	38.269
FB_0469366_L2_PA	fp02_38	2	38.269
FB_0469341_L2_PA	fp02_38	2	38.269
FB_0469368_L2_PA	fp02_38	2	38.269
FB_0469367_L2_PA	fp02_38	2	38.269
FB_0469344_L2_PA	fp02_38	2	38.269
FB_0468913_L2_PA	fp02_39	2	39.52
FB_0468911_L2_PA	fp02_39	2	39.52

RB_13646169_L2_PA	fp02_39	2	39.52
RB_14503620_L2_PA	fp02_39	2	39.52
RB_14573434_L2_PA	fp02_39	2	39.52
FB_0468907_L2_PA	fp02_40	2	40.479
FB_1084869_L2_42_2	fp02_41	2	41.306
RB_13673501_L2_PA	fp02_41	2	41.306
FB_0468392_L2_PA	fp02_42	2	42.62
GD_00214_L2_PA	fp02_42	2	42.62
FB_0468895_L2_PA	fp02_42	2	42.62
FB_0467370_L2_PA	fp02_42	2	42.62
FB_0954531_L2_25_1	fp02_42	2	42.62
RB_13641108_L2_PA	fp02_43	2	43.377
RB_12926120_L2_PA	fp02_43	2	43.377
FB_0627922_L5_PA	fp02_43	2	43.377
FB_0468393_L2_PA	fp02_43	2	43.377
FB_0468399_L2_PA	fp02_44	2	44.786
GD_01552_L2_PA	fp02_44	2	44.786
FB_0954530_L2_25_1	fp02_44	2	44.786
FB_0467398_L2_PA	fp02_44	2	44.786
FB_0467379_L2_PA	fp02_44	2	44.786
FB_0467378_L2_PA	fp02_44	2	44.786
RB_12933833_L2_25_1	fp02_45	2	45.612
RB_13676580_L2_PA	fp02_45	2	45.612
RB_13055225_L2_25_1	fp02_45	2	45.612
RB_13683046_L2_PA	fp02_45	2	45.612
RB_12903929_L2_PA	fp02_45	2	45.612
FB_0467373_L2_PA	fp02_45	2	45.612
FB_0627932_L5_PA	fp02_45	2	45.612
RB_13651288_L2_PA	fp02_45	2	45.612
FB_0954525_L2_25_1	fp02_45	2	45.612
FB_0467387_L2_PA	fp02_45	2	45.612
FB_0627911_L5_PA	fp02_46	2	46.61
RB_11996518_L2_PA	fp02_48	2	48.408
RB_11969851_L2_PA	fp02_48	2	48.408
FB_0466123_L2_PA	fp02_48	2	48.408
FB_0466107_L2_PA	fp02_48	2	48.408
FB_0466103_L2_PA	fp02_48	2	48.408
GD_01005_L2_PA	fp02_48	2	48.408
FB_0466092_L2_PA	fp02_48	2	48.408
RB_11512294_L2_PA	fp02_49	2	49.292
RB_11965366_L2_PA	fp02_49	2	49.292
FB_0466113_L2_PA	fp02_49	2	49.292
RB_11777704_L2_PA	fp02_49	2	49.292
RB_11806852_L2_PA	fp02_49	2	49.292
RB_11546546_L2_PA	fp02_49	2	49.292
RB_11993525_L2_PA	fp02_49	2	49.292

RB_11799850_L2_PA	fp02_49	2	49.292
RB_11972340_L2_PA	fp02_49	2	49.292
RB_11771410_L2_PA	fp02_49	2	49.292
FB_0466109_L2_PA	fp02_49	2	49.292
FB_0466115_L2_PA	fp02_50	2	50.42
FB_0465714_L2_PA	fp02_50	2	50.42
RB_11503103_L2_PA	fp02_50	2	50.42
FB_0465730_L2_PA	fp02_50	2	50.42
FB_0465710_L2_PA	fp02_50	2	50.42
RB_11409481_L2_PA	fp02_50	2	50.42
FB_0465732_L2_PA	fp02_50	2	50.42
RB_11438433_L2_PA	fp02_50	2	50.42
FB_0465726_L2_PA	fp02_50	2	50.42
FB_0465713_L2_PA	fp02_50	2	50.42
RB_11509973_L2_PA	fp02_50	2	50.42
FB_1084297_L2_40_2	fp02_50	2	50.42
GD_00991_L2_PA	fp02_50	2	50.42
RB_11414678_L2_PA	fp02_50	2	50.42
RB_11411515_L2_PA	fp02_50	2	50.42
FB_0464916_L2_PA	fp02_50	2	50.42
RB_11950668_L2_PA	fp02_51	2	51.641
RB_8928904_L2_PA	fp02_51	2	51.641
FB_0597564_L5_PA	fp02_51	2	51.641
RB_8945207_L2_PA	fp02_51	2	51.641
RB_10654569_L2_PA	fp02_51	2	51.641
FB_0953388_L2_23_1	fp02_51	2	51.641
RB_11758165_L2_PA	fp02_51	2	51.641
GD_00505_L2_PA	fp02_51	2	51.641
FB_0597559_L5_PA	fp02_51	2	51.641
FB_0908657_L15_59_1	fp02_51	2	51.641
FB_0597567_L5_PA	fp02_51	2	51.641
FB_1084306_L2_40_2	fp02_51	2	51.641
GD_00159_L2_PA	fp02_51	2	51.641
FB_0464920_L2_PA	fp02_51	2	51.641
RB_8931588_L2_PA	fp02_51	2	51.641
FB_0465725_L2_PA	fp02_51	2	51.641
FB_0461479_L2_PA	fp02_52	2	52.793
GD_00614_L2_PA	fp02_52	2	52.793
FB_0462359_L2_PA	fp02_52	2	52.793
FB_0953369_L2_23_1	fp02_52	2	52.793
RB_8922212_L2_PA	fp02_52	2	52.793
FB_0953837_L2_24_1	fp02_52	2	52.793
RB_8936738_L2_23_1	fp02_52	2	52.793
FB_1084116_L2_38_2	fp02_52	2	52.793
FB_0953386_L2_23_1	fp02_52	2	52.793
FB_0462357_L2_PA	fp02_52	2	52.793

FB_0953831_L2_24_1	fp02_52	2	52.793
FB_0461769_L2_PA	fp02_52	2	52.793
FB_0461772_L2_PA	fp02_52	2	52.793
FB_1084296_L2_40_2	fp02_52	2	52.793
FB_1084117_L2_38_2	fp02_52	2	52.793
FB_0464926_L2_PA	fp02_52	2	52.793
FB_0953394_L2_23_1	fp02_52	2	52.793
FB_1084305_L2_40_2	fp02_52	2	52.793
FB_0461755_L2_PA	fp02_52	2	52.793
FB_0464918_L2_PA	fp02_52	2	52.793
FB_0461773_L2_PA	fp02_53	2	53.609
FB_0461770_L2_PA	fp02_53	2	53.609
FB_1084311_L2_40_2	fp02_53	2	53.609
RB_7255188_L2_PA	fp02_53	2	53.609
FB_0461760_L2_PA	fp02_53	2	53.609
RB_9337414_L2_PA	fp02_53	2	53.609
RB_11451663_L2_PA	fp02_54	2	54.439
FB_0458614_L2_PA	fp02_54	2	54.439
FB_0461758_L2_PA	fp02_54	2	54.439
RB_7318523_L2_PA	fp02_54	2	54.439
RB_7035308_L2_PA	fp02_54	2	54.439
FB_1084022_L2_38_2	fp02_54	2	54.439
FB_1084034_L2_38_2	fp02_54	2	54.439
FB_1084031_L2_38_2	fp02_54	2	54.439
FB_1084024_L2_38_2	fp02_54	2	54.439
RB_10678809_L2_PA	fp02_54	2	54.439
RB_7279497_L2_21_1	fp02_54	2	54.439
GD_01223_L2_24_1	fp02_54	2	54.439
FB_1113871_L2_54_3	fp02_55	2	55.09
FB_1113873_L2_54_3	fp02_55	2	55.09
FB_1113880_L2_54_3	fp02_55	2	55.09
FB_1084118_L2_38_2	fp02_55	2	55.09
RB_10669023_L2_24_1	fp02_55	2	55.09
RB_6567574_L2_PA	fp02_56	2	56.287
FB_0459441_L2_PA	fp02_56	2	56.287
RB_6522059_L2_PA	fp02_56	2	56.287
FB_0459455_L2_PA	fp02_56	2	56.287
GD_01162_L2_PA	fp02_56	2	56.287
RB_6616886_L2_PA	fp02_56	2	56.287
FB_0459468_L2_PA	fp02_56	2	56.287
FB_0459462_L2_PA	fp02_56	2	56.287
RB_6573609_L2_PA	fp02_56	2	56.287
RB_6499131_L2_PA	fp02_56	2	56.287
GD_00308_L2_38_2	fp02_57	2	57.582
FB_0458041_L2_PA	fp02_57	2	57.582
FB_1083541_L2_38_2	fp02_57	2	57.582

FB_1083547_L2_38_2	fp02_57	2	57.582
FB_1083561_L2_38_2	fp02_57	2	57.582
FB_1083564_L2_38_2	fp02_57	2	57.582
RB_6466363_L2_PA	fp02_57	2	57.582
FB_1083538_L2_38_2	fp02_57	2	57.582
FB_0458664_L2_PA	fp02_57	2	57.582
RB_6482710_L2_PA	fp02_57	2	57.582
FB_0458633_L2_PA	fp02_58	2	58.151
FB_0458613_L2_PA	fp02_58	2	58.151
FB_0457723_L2_PA	fp02_58	2	58.151
RB_5931646_L2_PA	fp02_58	2	58.151
RB_6266265_L2_PA	fp02_58	2	58.151
FB_1113586_L2_54_3	fp02_58	2	58.151
RB_5926620_L2_PA	fp02_58	2	58.151
FB_0457233_L2_PA	fp02_59	2	59.687
FB_0457219_L2_PA	fp02_59	2	59.687
FB_0457262_L2_PA	fp02_59	2	59.687
FB_0457224_L2_PA	fp02_59	2	59.687
FB_0457225_L2_PA	fp02_59	2	59.687
FB_1113567_L2_54_3	fp02_60	2	60.474
RB_5335844_L2_PA	fp02_60	2	60.474
FB_0456739_L2_PA	fp02_60	2	60.474
FB_0456732_L2_PA	fp02_60	2	60.474
FB_0951112_L2_21_1	fp02_60	2	60.474
RB_5074647_L2_PA	fp02_60	2	60.474
FB_1113577_L2_54_3	fp02_60	2	60.474
FB_0456727_L2_PA	fp02_60	2	60.474
FB_1113562_L2_54_3	fp02_60	2	60.474
FB_0456743_L2_PA	fp02_60	2	60.474
FB_0456703_L2_PA	fp02_60	2	60.474
GD_00077_L2_PA	fp02_60	2	60.474
FB_1113583_L2_54_3	fp02_60	2	60.474
FB_0456724_L2_PA	fp02_60	2	60.474
RB_5314728_L2_PA	fp02_60	2	60.474
FB_0456731_L2_PA	fp02_60	2	60.474
FB_0456735_L2_PA	fp02_61	2	61.454
FB_0456226_L2_PA	fp02_62	2	62.439
FB_0950614_L2_21_1	fp02_62	2	62.439
FB_0950604_L2_21_1	fp02_62	2	62.439
FB_0950619_L2_21_1	fp02_62	2	62.439
FB_0950607_L2_21_1	fp02_62	2	62.439
FB_0456228_L2_PA	fp02_62	2	62.439
FB_0456233_L2_PA	fp02_62	2	62.439
FB_0950621_L2_21_1	fp02_62	2	62.439
RB_4873695_L2_PA	fp02_62	2	62.439
FB_0457244_L2_PA	fp02_62	2	62.439

FB_0455806_L2_PA	fp02_62	2	62.439
FB_0455810_L2_PA	fp02_62	2	62.439
FB_0455789_L2_PA	fp02_63	2	63.581
FB_0455800_L2_PA	fp02_63	2	63.581
RB_4554201_L2_PA	fp02_63	2	63.581
FB_0455794_L2_PA	fp02_63	2	63.581
RB_4373183_L2_PA	fp02_63	2	63.581
RB_4012052_L2_21_1	fp02_63	2	63.581
FB_0455077_L2_PA	fp02_63	2	63.581
FB_0950389_L2_21_1	fp02_63	2	63.581
RB_4352570_L2_PA	fp02_64	2	64.439
RB_4345275_L2_PA	fp02_64	2	64.439
RB_4227094_L2_PA	fp02_64	2	64.439
RB_4411200_L2_PA	fp02_64	2	64.439
Rvi4_region3_K9356	fp02_64	2	64.439
FB_1083231_L2_38_2	fp02_64	2	64.439
FB_1083243_L2_38_2	fp02_64	2	64.439
RB_4194273_L2_PA	fp02_64	2	64.439
FB_0950392_L2_21_1	fp02_64	2	64.439
RB_4176344_L2_PA	fp02_64	2	64.439
RB_4554251_L2_PA	fp02_64	2	64.439
RB_4030165_L2_PA	fp02_64	2	64.439
FB_0950385_L2_21_1	fp02_64	2	64.439
FB_0454647_L2_PA	fp02_64	2	64.439
FB_0455080_L2_PA	fp02_64	2	64.439
FB_1083203_L2_38_2	fp02_64	2	64.439
RB_3463154_L2_PA	fp02_64	2	64.439
GD_01476_L2_PA	fp02_64	2	64.439
FB_1083204_L2_38_2	fp02_64	2	64.439
RB_3243976_L2_PA	fp02_64	2	64.439
FB_0949981_L2_21_1	fp02_64	2	64.439
RB_3307093_L2_21_1	fp02_64	2	64.439
FB_1083197_L2_38_2	fp02_64	2	64.439
RB_2995367_L2_PA	fp02_64	2	64.439
FB_1083196_L2_38_2	fp02_64	2	64.439
FB_0949978_L2_21_1	fp02_64	2	64.439
FB_0452865_L2_PA	fp02_64	2	64.439
RB_3428296_L2_PA	fp02_64	2	64.439
FB_1083130_L2_38_2	fp02_64	2	64.439
GD_01641_L2_PA	fp02_64	2	64.439
FB_0950382_L2_21_1	fp02_64	2	64.439
FB_0455073_L2_PA	fp02_64	2	64.439
RB_3420282_L2_PA	fp02_64	2	64.439
FB_1083131_L2_38_2	fp02_65	2	65.446
FB_0949693_L2_21_1	fp02_65	2	65.446
FB_1083115_L2_38_2	fp02_65	2	65.446

FB_1083121_L2_38_2	fp02_65	2	65.446
FB_1083134_L2_38_2	fp02_65	2	65.446
RB_4173601_L2_PA	fp02_65	2	65.446
RB_4554235_L2_PA	fp02_65	2	65.446
FB_0455065_L2_PA	fp02_65	2	65.446
FB_0452878_L2_PA	fp02_66	2	66.227
RB_3273690_L2_PA	fp02_67	2	67.451
FB_0950003_L2_21_1	fp02_67	2	67.451
FB_0452371_L2_PA	fp02_67	2	67.451
FB_0452379_L2_PA	fp02_67	2	67.451
FB_0452383_L2_PA	fp02_67	2	67.451
FB_0452358_L2_PA	fp02_67	2	67.451
FB_1083139_L2_38_2	fp02_67	2	67.451
RB_2987146_L2_PA	fp02_67	2	67.451
RB_3303584_L2_21_1	fp02_67	2	67.451
FB_0454637_L2_PA	fp02_67	2	67.451
FB_0949267_L2_21_1	fp02_67	2	67.451
FB_0452018_L2_PA	fp02_67	2	67.451
FB_0452368_L2_PA	fp02_67	2	67.451
RB_2425858_L2_38_2	fp02_67	2	67.451
FB_0949261_L2_21_1	fp02_67	2	67.451
GD_01565_L2_21_1	fp02_67	2	67.451
FB_0949262_L2_21_1	fp02_67	2	67.451
FB_0452019_L2_PA	fp02_67	2	67.451
RB_2408882_L2_PA	fp02_67	2	67.451
FB_1083118_L2_38_2	fp02_68	2	68.221
FB_1083123_L2_38_2	fp02_68	2	68.221
FB_0452407_L2_PA	fp02_68	2	68.221
FB_0450663_L2_PA	fp02_68	2	68.221
FB_0450665_L2_PA	fp02_68	2	68.221
CO_903605_L2_38_2	fp02_68	2	68.221
FB_0451026_L2_PA	fp02_68	2	68.221
FB_0452022_L2_PA	fp02_68	2	68.221
FB_0460377_L2_PA	fp02_68	2	68.221
FB_0460371_L2_PA	fp02_68	2	68.221
RB_1958621_L2_PA	fp02_68	2	68.221
FB_0451016_L2_PA	fp02_68	2	68.221
FB_0450676_L2_PA	fp02_68	2	68.221
RB_1270705_L2_PA	fp02_68	2	68.221
FB_0451356_L2_PA	fp02_68	2	68.221
RB_1979331_L2_PA	fp02_68	2	68.221
FB_0451351_L2_PA	fp02_68	2	68.221
FB_0451008_L2_PA	fp02_68	2	68.221
RB_1869486_L2_PA	fp02_68	2	68.221
FB_0450673_L2_PA	fp02_68	2	68.221
FB_0451346_L2_PA	fp02_68	2	68.221

RB_1926287_L2_21_1	fp02_68	2	68.221
FB_0451368_L2_PA	fp02_68	2	68.221
RB_1908444_L2_21_1	fp02_68	2	68.221
FB_0460384_L2_PA	fp02_68	2	68.221
FB_0450669_L2_PA	fp02_68	2	68.221
RB_1910740_L2_21_1	fp02_68	2	68.221
FB_0450010_L2_PA	fp02_69	2	69.555
FB_0450019_L2_PA	fp02_69	2	69.555
FB_1082837_L2_38_2	fp02_69	2	69.555
GD_01134_L2_PA	fp02_69	2	69.555
FB_0449396_L2_PA	fp02_69	2	69.555
RB_1272762_L2_PA	fp02_69	2	69.555
FB_0449998_L2_PA	fp02_69	2	69.555
FB_0450005_L2_PA	fp02_69	2	69.555
FB_0449395_L2_PA	fp02_69	2	69.555
RB_1219423_L2_PA	fp02_70	2	70.76
FB_0666833_L6_PA	fp03_00	3	0.269
RB_744263_L3_87_3	fp03_00	3	0.269
FB_0666815_L6_PA	fp03_00	3	0.269
FB_0666829_L6_PA	fp03_00	3	0.269
FB_0666801_L6_PA	fp03_00	3	0.269
RB_1142062_L3_PA	fp03_00	3	0.269
RB_982733_L3_PA	fp03_01	3	1.682
FB_0531586_L3_PA	fp03_01	3	1.682
FB_0506616_L3_PA	fp03_01	3	1.682
FB_0506601_L3_PA	fp03_01	3	1.682
GD_00506_L3_PA	fp03_01	3	1.682
FB_0506611_L3_PA	fp03_01	3	1.682
RB_999208_L3_PA	fp03_01	3	1.682
RB_693011_L3_PA	fp03_01	3	1.682
RB_705112_L3_PA	fp03_01	3	1.682
GD_00866_L3_PA	fp03_01	3	1.682
FB_0171139_L13_PA	fp03_01	3	1.682
FB_0053133_L10_PA	fp03_01	3	1.682
RB_5111953_L3_PA	fp03_01	3	1.682
FB_0510372_L3_PA	fp03_01	3	1.682
FB_0507630_L3_PA	fp03_01	3	1.682
FB_0507632_L3_PA	fp03_01	3	1.682
FB_0171124_L13_PA	fp03_01	3	1.682
RB_3879835_L3_PA	fp03_01	3	1.682
FB_0171130_L13_PA	fp03_01	3	1.682
RB_1122860_L3_PA	fp03_01	3	1.682
FB_0171126_L13_PA	fp03_01	3	1.682
RB_1035920_L3_PA	fp03_01	3	1.682
FB_1055492_L10_71_2	fp03_01	3	1.682
RB_2659646_L3_PA	fp03_01	3	1.682

RB_3834966_L3_33_1	fp03_01	3	1.682
RB_1759940_L3_PA	fp03_01	3	1.682
FB_0507616_L3_PA	fp03_01	3	1.682
FB_0171157_L13_PA	fp03_01	3	1.682
RB_1101058_L3_PA	fp03_01	3	1.682
FB_0508594_L3_PA	fp03_01	3	1.682
FB_0507619_L3_PA	fp03_01	3	1.682
FB_0060688_L11_PA	fp03_01	3	1.682
FB_0508599_L3_PA	fp03_01	3	1.682
FB_0508582_L3_PA	fp03_01	3	1.682
FB_0507628_L3_PA	fp03_01	3	1.682
FB_0171143_L13_PA	fp03_01	3	1.682
RB_1131825_L3_PA	fp03_01	3	1.682
RB_1057933_L3_PA	fp03_01	3	1.682
GD_02300_L3_PA	fp03_01	3	1.682
RB_2462827_L3_PA	fp03_01	3	1.682
FB_0508590_L3_PA	fp03_01	3	1.682
FB_0531581_L3_PA	fp03_01	3	1.682
RB_1948548_L3_PA	fp03_02	3	2.374
RB_1996499_L3_PA	fp03_02	3	2.374
FB_0666811_L6_PA	fp03_02	3	2.374
RB_708042_L3_PA	fp03_02	3	2.374
RB_6160259_L3_PA	fp03_02	3	2.374
RB_5923532_L3_35_1	fp03_02	3	2.374
FB_0510256_L3_PA	fp03_02	3	2.374
FB_0508640_L3_PA	fp03_02	3	2.374
FB_0508639_L3_PA	fp03_02	3	2.374
FB_0510370_L3_PA	fp03_02	3	2.374
FB_0510270_L3_PA	fp03_02	3	2.374
FB_1055478_L10_71_2	fp03_02	3	2.374
FB_0964046_L3_33_1	fp03_03	3	3.235
FB_0964042_L3_33_1	fp03_03	3	3.235
FB_0964039_L3_33_1	fp03_03	3	3.235
FB_0964034_L3_33_1	fp03_03	3	3.235
FB_0509863_L3_PA	fp03_03	3	3.235
FB_0964033_L3_33_1	fp03_03	3	3.235
GD_00124_L3_PA	fp03_03	3	3.235
FB_0509864_L3_PA	fp03_03	3	3.235
FB_0964043_L3_33_1	fp03_03	3	3.235
FB_0509752_L3_PA	fp03_03	3	3.235
FB_0509750_L3_PA	fp03_03	3	3.235
FB_0053141_L10_PA	fp03_03	3	3.235
FB_1055498_L10_71_2	fp03_03	3	3.235
FB_0964044_L3_33_1	fp03_03	3	3.235
FB_0510273_L3_PA	fp03_03	3	3.235
GD_00276_L3_35	fp03_03	3	3.235

FB_0510271_L3_PA	fp03_03	3	3.235
FB_1108527_L11_72_3	fp03_03	3	3.235
FB_1108526_L11_72_3	fp03_03	3	3.235
FB_0510280_L3_PA	fp03_03	3	3.235
FB_0964036_L3_33_1	fp03_04	3	4.498
FB_0510361_L3_PA	fp03_04	3	4.498
FB_0510363_L3_PA	fp03_04	3	4.498
FB_0510359_L3_PA	fp03_04	3	4.498
FB_0510368_L3_PA	fp03_04	3	4.498
FB_0510353_L3_PA	fp03_04	3	4.498
RB_4958565_L3_PA	fp03_04	3	4.498
FB_0510362_L3_PA	fp03_04	3	4.498
GD_00073_L3_35_1	fp03_04	3	4.498
FB_0510764_L3_PA	fp03_04	3	4.498
FB_0510714_L3_PA	fp03_04	3	4.498
FB_0510716_L3_PA	fp03_04	3	4.498
GD_01957_L3_PA	fp03_04	3	4.498
FB_0510712_L3_PA	fp03_04	3	4.498
FB_0510715_L3_PA	fp03_04	3	4.498
FB_0510724_L3_PA	fp03_04	3	4.498
FB_0512519_L3_PA	fp03_06	3	6.648
RB_5949706_L3_PA	fp03_06	3	6.648
RB_5968340_L3_PA	fp03_06	3	6.648
FB_0512516_L3_PA	fp03_06	3	6.648
FB_0512515_L3_PA	fp03_06	3	6.648
RB_6162720_L3_PA	fp03_07	3	7.656
FB_1082527_L1_84_2	fp03_07	3	7.656
FB_0514815_L3_PA	fp03_07	3	7.656
FB_0513181_L3_PA	fp03_07	3	7.656
RB_6181881_L3_53_1	fp03_07	3	7.656
FB_1082529_L1_84_2	fp03_08	3	8.47
RB_6543192_L3_PA	fp03_08	3	8.47
FB_0513160_L3_PA	fp03_08	3	8.47
RB_7138274_L3_35_1	fp03_08	3	8.47
FB_0513165_L3_PA	fp03_08	3	8.47
FB_0514188_L3_PA	fp03_08	3	8.47
FB_0513152_L3_PA	fp03_08	3	8.47
FB_0513171_L3_PA	fp03_08	3	8.47
FB_0513161_L3_PA	fp03_08	3	8.47
FB_0513157_L3_PA	fp03_08	3	8.47
RB_6212477_L3_PA	fp03_08	3	8.47
FB_0513187_L3_PA	fp03_08	3	8.47
RB_5926913_L3_35_1	fp03_08	3	8.47
FB_1082525_L1_84_2	fp03_08	3	8.47
RB_6242616_L3_PA	fp03_08	3	8.47
RB_6539382_L3_PA	fp03_09	3	9.63

FB_0515961_L3_PA	fp03_09	3	9.63
FB_0513966_L3_PA	fp03_09	3	9.63
FB_1082540_L1_84_2	fp03_09	3	9.63
RB_7140782_L3_35_1	fp03_09	3	9.63
FB_1082526_L1_84_2	fp03_09	3	9.63
RB_6561113_L3_PA	fp03_09	3	9.63
FB_0513956_L3_PA	fp03_09	3	9.63
FB_1082537_L1_84_2	fp03_09	3	9.63
FB_0514189_L3_PA	fp03_09	3	9.63
FB_1082531_L1_84_2	fp03_09	3	9.63
FB_0514816_L3_PA	fp03_09	3	9.63
FB_1082532_L1_84_2	fp03_09	3	9.63
FB_1082539_L1_84_2	fp03_09	3	9.63
FB_0514811_L3_PA	fp03_09	3	9.63
FB_0514203_L3_PA	fp03_09	3	9.63
FB_0514812_L3_PA	fp03_09	3	9.63
FB_0514810_L3_PA	fp03_09	3	9.63
FB_1082550_L1_84_2	fp03_09	3	9.63
FB_1082533_L1_84_2	fp03_09	3	9.63
RB_7146440_L3_PA	fp03_10	3	10.698
GD_01493_L3_PA	fp03_10	3	10.698
FB_0515445_L3_PA	fp03_10	3	10.698
FB_0514826_L3_PA	fp03_10	3	10.698
FB_0515442_L3_PA	fp03_10	3	10.698
FB_0515428_L3_PA	fp03_10	3	10.698
FB_0515435_L3_PA	fp03_10	3	10.698
FB_0515444_L3_PA	fp03_10	3	10.698
FB_0515431_L3_PA	fp03_10	3	10.698
FB_1058176_L11_59_2	fp03_11	3	11.486
RB_7164921_L3_PA	fp03_11	3	11.486
RB_7171067_L3_PA	fp03_11	3	11.486
RB_7178316_L3_PA	fp03_12	3	12.434
RB_8503130_L3_PA	fp03_12	3	12.434
FB_0516384_L3_PA	fp03_12	3	12.434
FB_0516385_L3_PA	fp03_12	3	12.434
RB_8516804_L3_PA	fp03_12	3	12.434
FB_0516374_L3_PA	fp03_12	3	12.434
RB_8507285_L3_PA	fp03_12	3	12.434
FB_0516386_L3_PA	fp03_12	3	12.434
RB_8499613_L3_PA	fp03_12	3	12.434
FB_0966321_L3_36_1	fp03_12	3	12.434
FB_0966313_L3_36_1	fp03_13	3	13.395
FB_0517256_L3_PA	fp03_13	3	13.395
FB_0516399_L3_PA	fp03_13	3	13.395
FB_0516380_L3_PA	fp03_13	3	13.395
FB_1114994_L3_91_3	fp03_13	3	13.395

FB_0515938_L3_PA	fp03_13	3	13.395
FB_0516982_L3_PA	fp03_13	3	13.395
FB_0966311_L3_36_1	fp03_13	3	13.395
FB_0516397_L3_PA	fp03_13	3	13.395
RB_9101679_L3_PA	fp03_13	3	13.395
RB_9091637_L3_PA	fp03_13	3	13.395
FB_0516392_L3_PA	fp03_13	3	13.395
FB_0966316_L3_36_1	fp03_13	3	13.395
FB_0516970_L3_PA	fp03_13	3	13.395
FB_0966558_L3_36_1	fp03_14	3	14.389
FB_0966560_L3_36_1	fp03_14	3	14.389
RB_9544220_L3_PA	fp03_14	3	14.389
FB_0518011_L3_PA	fp03_14	3	14.389
FB_0518016_L3_PA	fp03_14	3	14.389
FB_0966555_L3_36_1	fp03_14	3	14.389
FB_0518032_L3_PA	fp03_14	3	14.389
FB_0966546_L3_36_1	fp03_14	3	14.389
FB_0966551_L3_36_1	fp03_14	3	14.389
FB_0518012_L3_PA	fp03_14	3	14.389
FB_0966543_L3_36_1	fp03_14	3	14.389
FB_0967290_L3_36_1	fp03_15	3	15.8
FB_1115016_L3_91_3	fp03_15	3	15.8
FB_1115015_L3_91_3	fp03_15	3	15.8
FB_0515934_L3_PA	fp03_15	3	15.8
FB_0515937_L3_PA	fp03_15	3	15.8
FB_1088358_L3_66_2	fp03_15	3	15.8
FB_1115013_L3_91_3	fp03_15	3	15.8
FB_1088361_L3_66_2	fp03_15	3	15.8
FB_1115033_L3_91_3	fp03_15	3	15.8
FB_0515965_L3_PA	fp03_15	3	15.8
FB_0519397_L3_PA	fp03_15	3	15.8
FB_1115014_L3_91_3	fp03_15	3	15.8
RB_10498941_L3_PA	fp03_15	3	15.8
FB_0515956_L3_PA	fp03_15	3	15.8
FB_0966808_L3_36_1	fp03_15	3	15.8
FB_0519986_L3_PA	fp03_15	3	15.8
FB_1115020_L3_91_3	fp03_15	3	15.8
RB_9121744_L3_PA	fp03_15	3	15.8
RB_10537812_L3_PA	fp03_16	3	16.282
RB_10518958_L3_PA	fp03_16	3	16.282
FB_0518727_L3_PA	fp03_16	3	16.282
FB_0518719_L3_PA	fp03_16	3	16.282
FB_0518723_L3_PA	fp03_16	3	16.282
FB_0518713_L3_PA	fp03_16	3	16.282
RB_9974411_L3_PA	fp03_17	3	17.02
FB_0519403_L3_PA	fp03_18	3	18.24

FB_0519392_L3_PA	fp03_18	3	18.24
FB_0519418_L3_PA	fp03_18	3	18.24
FB_0967304_L3_36_1	fp03_18	3	18.24
FB_0519398_L3_PA	fp03_18	3	18.24
FB_0519976_L3_PA	fp03_18	3	18.24
FB_0519988_L3_PA	fp03_18	3	18.24
FB_0519985_L3_PA	fp03_18	3	18.24
FB_0519395_L3_PA	fp03_18	3	18.24
FB_0519428_L3_PA	fp03_18	3	18.24
FB_0967988_L3_36_1	fp03_19	3	19.476
RB_10540206_L3_PA	fp03_20	3	20.169
GD_00874_L3_36_1	fp03_21	3	21.192
FB_0967968_L3_36_1	fp03_22	3	22.212
GD_00194_L3_PA	fp03_22	3	22.212
RB_10707191_L3_36_1	fp03_22	3	22.212
FB_0909627_L15_60_1	fp03_22	3	22.212
FB_0909633_L15_60_1	fp03_22	3	22.212
FB_1084121_L2_38_2	fp03_22	3	22.212
FB_0521105_L3_PA	fp03_22	3	22.212
FB_0522112_L3_PA	fp03_22	3	22.212
RB_11552808_L3_PA	fp03_22	3	22.212
FB_1088748_L3_67_2	fp03_23	3	23.396
FB_0522939_L3_PA	fp03_23	3	23.396
FB_0522937_L3_PA	fp03_23	3	23.396
GD_01885_L3_PA	fp03_23	3	23.396
FB_0967957_L3_36_1	fp03_23	3	23.396
FB_0522930_L3_PA	fp03_23	3	23.396
RB_14445035_L3_PA	fp03_23	3	23.396
RB_11933435_L3_PA	fp03_23	3	23.396
FB_0522941_L3_PA	fp03_23	3	23.396
GD_02020_L3_PA	fp03_24	3	24.436
FB_0524741_L3_PA	fp03_24	3	24.436
FB_0739977_L8_PA	fp03_24	3	24.436
FB_0739957_L8_PA	fp03_24	3	24.436
FB_0739942_L8_PA	fp03_24	3	24.436
FB_0524740_L3_PA	fp03_24	3	24.436
FB_0525289_L3_PA	fp03_24	3	24.436
FB_0441638_L1_PA	fp03_24	3	24.436
RB_13512352_L3_38_1	fp03_24	3	24.436
FB_0739943_L8_PA	fp03_24	3	24.436
FB_0524777_L3_PA	fp03_24	3	24.436
FB_0525277_L3_PA	fp03_24	3	24.436
FB_0524762_L3_PA	fp03_25	3	25.737
RB_15369623_L3_PA	fp03_25	3	25.737
RB_12760479_L3_PA	fp03_25	3	25.737
FB_0441636_L1_PA	fp03_25	3	25.737

FB_0525286_L3_PA	fp03_25	3	25.737
FB_0524745_L3_PA	fp03_25	3	25.737
FB_0526085_L3_PA	fp03_25	3	25.737
FB_0526347_L3_PA	fp03_25	3	25.737
FB_0226249_L14_PA	fp03_25	3	25.737
FB_0226247_L14_PA	fp03_25	3	25.737
FB_0226262_L14_PA	fp03_25	3	25.737
FB_0526105_L3_PA	fp03_25	3	25.737
FB_0226250_L14_PA	fp03_25	3	25.737
FB_1088745_L3_67_2	fp03_25	3	25.737
RB_11994788_L3_PA	fp03_25	3	25.737
FB_0529680_L3_PA	fp03_25	3	25.737
RB_11572647_L3_PA	fp03_25	3	25.737
FB_0750655_L8_PA	fp03_25	3	25.737
FB_0528609_L3_PA	fp03_25	3	25.737
FB_0523626_L3_PA	fp03_25	3	25.737
FB_0522934_L3_PA	fp03_25	3	25.737
FB_0528610_L3_PA	fp03_26	3	26.312
FB_0526754_L3_PA	fp03_26	3	26.312
FB_0529522_L3_PA	fp03_26	3	26.312
FB_0526748_L3_PA	fp03_26	3	26.312
FB_0528613_L3_PA	fp03_26	3	26.312
GD_02546_L3_PA	fp03_26	3	26.312
FB_0524775_L3_PA	fp03_26	3	26.312
FB_0989077_L5_34_1	fp03_27	3	27.494
FB_0989098_L5_34_1	fp03_27	3	27.494
FB_0989088_L5_34_1	fp03_27	3	27.494
FB_0989070_L5_34_1	fp03_27	3	27.494
FB_0750664_L8_PA	fp03_27	3	27.494
RB_16704479_L3_PA	fp03_27	3	27.494
RB_14457107_L3_PA	fp03_27	3	27.494
FB_0989069_L5_34_1	fp03_27	3	27.494
FB_0989094_L5_34_1	fp03_27	3	27.494
FB_0528089_L3_PA	fp03_27	3	27.494
RB_15329250_L3_PA	fp03_27	3	27.494
FB_0528620_L3_PA	fp03_27	3	27.494
FB_0528100_L3_PA	fp03_27	3	27.494
RB_16704372_L3_PA	fp03_27	3	27.494
FB_0528607_L3_PA	fp03_27	3	27.494
FB_0528626_L3_PA	fp03_27	3	27.494
FB_0750662_L8_PA	fp03_28	3	28.515
FB_0528097_L3_PA	fp03_28	3	28.515
FB_0528611_L3_PA	fp03_28	3	28.515
RB_16948009_L3_PA	fp03_28	3	28.515
FB_0750651_L8_PA	fp03_28	3	28.515
FB_0970428_L3_43_1	fp03_28	3	28.515

RB_18399291_L3_PA	fp03_28	3	28.515
RB_16990247_L3_PA	fp03_28	3	28.515
FB_0970302_L3_42_1	fp03_28	3	28.515
GD_00921_L3_PA	fp03_28	3	28.515
GD_02804_L3_43_1	fp03_28	3	28.515
FB_0177204_L13_PA	fp03_29	3	29.876
FB_0531788_L3_PA	fp03_29	3	29.876
FB_0970332_L3_43_1	fp03_29	3	29.876
FB_0532571_L3_PA	fp03_29	3	29.876
FB_0530628_L3_PA	fp03_29	3	29.876
FB_0530654_L3_PA	fp03_29	3	29.876
FB_0487706_L2_PA	fp03_29	3	29.876
RB_16704383_L3_PA	fp03_29	3	29.876
FB_0532324_L3_PA	fp03_29	3	29.876
FB_0970328_L3_43_1	fp03_29	3	29.876
FB_0487722_L2_PA	fp03_29	3	29.876
FB_0530651_L3_PA	fp03_29	3	29.876
FB_0532332_L3_PA	fp03_29	3	29.876
FB_0531795_L3_PA	fp03_29	3	29.876
FB_0702811_L7_PA	fp03_29	3	29.876
FB_0487702_L2_PA	fp03_29	3	29.876
FB_0530627_L3_PA	fp03_29	3	29.876
FB_0532325_L3_PA	fp03_29	3	29.876
RB_18379820_L3_42_1	fp03_29	3	29.876
FB_0487698_L2_PA	fp03_29	3	29.876
FB_0932246_L17_16_1	fp03_29	3	29.876
FB_0970417_L3_43_1	fp03_29	3	29.876
RB_16988161_L3_PA	fp03_29	3	29.876
FB_0532587_L3_PA	fp03_29	3	29.876
FB_0702808_L7_PA	fp03_29	3	29.876
FB_0532569_L3_PA	fp03_29	3	29.876
FB_0532580_L3_PA	fp03_29	3	29.876
FB_0532578_L3_PA	fp03_29	3	29.876
FB_0532335_L3_PA	fp03_30	3	30.407
FB_0532326_L3_PA	fp03_30	3	30.407
FB_0702838_L7_PA	fp03_30	3	30.407
RB_18978889_L3_43_1	fp03_30	3	30.407
FB_0531792_L3_PA	fp03_30	3	30.407
FB_0532568_L3_PA	fp03_30	3	30.407
FB_0532870_L3_PA	fp03_30	3	30.407
FB_0532869_L3_PA	fp03_30	3	30.407
FB_0970299_L3_42_1	fp03_30	3	30.407
FB_0532327_L3_PA	fp03_30	3	30.407
FB_0532328_L3_PA	fp03_30	3	30.407
FB_0532334_L3_PA	fp03_30	3	30.407
FB_0533167_L3_PA	fp03_31	3	31.119

GD_02644_L3_PA	fp03_31	3	31.119
FB_0534175_L3_PA	fp03_32	3	32.412
FB_0536360_L3_PA	fp03_32	3	32.412
FB_0536357_L3_PA	fp03_32	3	32.412
FB_0536359_L3_PA	fp03_32	3	32.412
FB_0536355_L3_PA	fp03_32	3	32.412
FB_1114911_L3_90_3	fp03_32	3	32.412
FB_0535696_L3_PA	fp03_32	3	32.412
FB_0536351_L3_PA	fp03_32	3	32.412
FB_0536356_L3_PA	fp03_32	3	32.412
FB_0987796_L4_25_1	fp03_32	3	32.412
RB_24102468_L3_PA	fp03_32	3	32.412
FB_0536365_L3_PA	fp03_32	3	32.412
FB_0533170_L3_PA	fp03_32	3	32.412
FB_0534189_L3_PA	fp03_32	3	32.412
FB_0533178_L3_PA	fp03_32	3	32.412
FB_0533171_L3_PA	fp03_32	3	32.412
FB_0511284_L3_PA	fp03_33	3	33.489
FB_0535691_L3_PA	fp03_33	3	33.489
FB_0534192_L3_PA	fp03_33	3	33.489
GD_01487_L3_PA	fp03_33	3	33.489
FB_0535687_L3_PA	fp03_33	3	33.489
RB_24111076_L3_PA	fp03_33	3	33.489
RB_25869287_L3_PA	fp03_33	3	33.489
RB_25903444_L3_PA	fp03_33	3	33.489
FB_0537050_L3_PA	fp03_35	3	35.258
RB_23360673_L3_PA	fp03_35	3	35.258
FB_0535697_L3_PA	fp03_35	3	35.258
FB_0539961_L3_PA	fp03_36	3	36.786
RB_25900349_L3_PA	fp03_36	3	36.786
FB_1072225_L15_84_2	fp03_36	3	36.786
FB_0538287_L3_PA	fp03_36	3	36.786
GD_00955_L3_48_1	fp03_36	3	36.786
FB_0537040_L3_PA	fp03_36	3	36.786
FB_0537033_L3_PA	fp03_36	3	36.786
FB_1072223_L15_84_2	fp03_36	3	36.786
FB_1072228_L15_84_2	fp03_36	3	36.786
FB_0915099_L15_75_1	fp03_36	3	36.786
FB_1019701_L7_48_1	fp03_36	3	36.786
FB_0537680_L3_PA	fp03_36	3	36.786
FB_0915100_L15_75_1	fp03_36	3	36.786
RB_24158738_L3_48_1	fp03_36	3	36.786
RB_25471087_L3_PA	fp03_37	3	37.542
FB_0537620_L3_PA	fp03_37	3	37.542
FB_0538278_L3_PA	fp03_37	3	37.542
RB_24524197_L3_PA	fp03_37	3	37.542

FB_0912451_L15_68_1	fp03_37	3	37.542
FB_1105290_L9_39_2	fp03_37	3	37.542
FB_1105286_L9_39_2	fp03_37	3	37.542
FB_0537437_L3_PA	fp03_37	3	37.542
FB_0537599_L3_PA	fp03_37	3	37.542
FB_0075836_L11_PA	fp03_37	3	37.542
FB_0075868_L11_PA	fp03_37	3	37.542
FB_1105289_L9_39_2	fp03_37	3	37.542
FB_1019688_L7_48_1	fp03_37	3	37.542
FB_1019706_L7_48_1	fp03_37	3	37.542
FB_0537677_L3_PA	fp03_37	3	37.542
FB_0537676_L3_PA	fp03_37	3	37.542
FB_0582598_L4_PA	fp03_37	3	37.542
FB_0539115_L3_PA	fp03_38	3	38.851
RB_25474108_L3_PA	fp03_38	3	38.851
RB_27212656_L3_PA	fp03_38	3	38.851
FB_0537608_L3_PA	fp03_38	3	38.851
FB_0537682_L3_PA	fp03_38	3	38.851
FB_0537054_L3_PA	fp03_39	3	39.413
FB_0539106_L3_PA	fp03_40	3	40.535
FB_0537678_L3_PA	fp03_40	3	40.535
RB_23307044_L3_PA	fp03_41	3	41.316
FB_1105299_L9_39_2	fp03_41	3	41.316
FB_0539505_L3_PA	fp03_42	3	42.297
FB_0539117_L3_PA	fp03_42	3	42.297
FB_0421299_L1_PA	fp03_42	3	42.297
FB_0538282_L3_PA	fp03_42	3	42.297
FB_0539481_L3_PA	fp03_42	3	42.297
FB_0421310_L1_PA	fp03_42	3	42.297
FB_0385336_L17_PA	fp03_42	3	42.297
FB_0538851_L3_PA	fp03_42	3	42.297
FB_0539100_L3_PA	fp03_42	3	42.297
FB_0539119_L3_PA	fp03_42	3	42.297
FB_0932003_L17_15_1	fp03_42	3	42.297
GD_01642_L3_PA	fp03_42	3	42.297
FB_0421316_L1_PA	fp03_42	3	42.297
FB_0421295_L1_PA	fp03_42	3	42.297
FB_0538856_L3_PA	fp03_42	3	42.297
FB_0538850_L3_PA	fp03_42	3	42.297
FB_0539107_L3_PA	fp03_42	3	42.297
FB_0539118_L3_PA	fp03_42	3	42.297
RB_27745711_L3_PA	fp03_44	3	44.401
FB_1089473_L3_79_2	fp03_44	3	44.401
FB_0972381_L3_52_1	fp03_44	3	44.401
FB_0540929_L3_PA	fp03_44	3	44.401
FB_0972394_L3_52_1	fp03_44	3	44.401

FB_1089469_L3_79_2	fp03_44	3	44.401
FB_1089463_L3_79_2	fp03_44	3	44.401
FB_0972396_L3_52_1	fp03_44	3	44.401
FB_0539974_L3_PA	fp03_44	3	44.401
FB_0539978_L3_PA	fp03_44	3	44.401
GD_00607_L3_PA	fp03_44	3	44.401
FB_0539968_L3_PA	fp03_44	3	44.401
FB_0539980_L3_PA	fp03_44	3	44.401
FB_0539966_L3_PA	fp03_44	3	44.401
FB_0972401_L3_52_1	fp03_45	3	45.925
FB_0540949_L3_PA	fp03_45	3	45.925
GD_02143_L3_PA	fp03_45	3	45.925
FB_0540940_L3_PA	fp03_45	3	45.925
FB_1089477_L3_79_2	fp03_45	3	45.925
RB_29362868_L3_PA	fp03_46	3	46.516
FB_0540943_L3_PA	fp03_46	3	46.516
FB_0540935_L3_PA	fp03_47	3	47.712
FB_0598053_L5_PA	fp03_48	3	48.805
FB_0988856_L5_34_1	fp03_48	3	48.805
FB_0988854_L5_34_1	fp03_48	3	48.805
FB_0988860_L5_34_1	fp03_48	3	48.805
FB_0988868_L5_34_1	fp03_48	3	48.805
FB_0988852_L5_34_1	fp03_48	3	48.805
RB_29678862_L3_PA	fp03_48	3	48.805
RB_29645640_L3_PA	fp03_48	3	48.805
FB_0988858_L5_34_1	fp03_48	3	48.805
FB_0972706_L3_54_1	fp03_48	3	48.805
FB_0541816_L3_PA	fp03_48	3	48.805
FB_0541809_L3_PA	fp03_48	3	48.805
FB_0542265_L3_PA	fp03_48	3	48.805
FB_0542277_L3_PA	fp03_48	3	48.805
RB_29674295_L3_PA	fp03_48	3	48.805
FB_0542263_L3_PA	fp03_48	3	48.805
FB_0541806_L3_PA	fp03_48	3	48.805
FB_0972927_L3_54_1	fp03_48	3	48.805
FB_0541828_L3_PA	fp03_48	3	48.805
FB_0541821_L3_PA	fp03_48	3	48.805
FB_0541807_L3_PA	fp03_48	3	48.805
GD_01969_L3_PA	fp03_48	3	48.805
GD_02463_L3_PA	fp03_48	3	48.805
FB_0972924_L3_54_1	fp03_48	3	48.805
RB_29370809_L3_PA	fp03_48	3	48.805
FB_0541819_L3_PA	fp03_49	3	49.632
FB_0541811_L3_PA	fp03_49	3	49.632
FB_0542275_L3_PA	fp03_49	3	49.632
FB_0973327_L3_55_1	fp03_49	3	49.632

FB_0973333_L3_55_1	fp03_49	3	49.632
RB_29946084_L3_PA	fp03_49	3	49.632
RB_29843361_L3_PA	fp03_49	3	49.632
FB_0542655_L3_PA	fp03_49	3	49.632
RB_29875324_L3_PA	fp03_49	3	49.632
FB_0973262_L3_54_1	fp03_51	3	51.475
FB_0973266_L3_54_1	fp03_51	3	51.475
FB_0544628_L3_PA	fp03_51	3	51.475
FB_0544635_L3_PA	fp03_51	3	51.475
RB_31100089_L3_PA	fp03_51	3	51.475
FB_0543926_L3_PA	fp03_51	3	51.475
FB_0973318_L3_55_1	fp03_53	3	53.444
FB_0973331_L3_55_1	fp03_53	3	53.444
GD_00663_L3_PA	fp03_53	3	53.444
FB_0543932_L3_PA	fp03_53	3	53.444
RB_31160644_L3_PA	fp03_53	3	53.444
FB_0543923_L3_PA	fp03_53	3	53.444
FB_0543921_L3_PA	fp03_53	3	53.444
FB_0546079_L3_PA	fp03_56	3	56.563
FB_0545607_L3_PA	fp03_56	3	56.563
FB_0546834_L3_PA	fp03_56	3	56.563
FB_0546827_L3_PA	fp03_56	3	56.563
FB_0546475_L3_PA	fp03_57	3	57.447
FB_0546054_L3_PA	fp03_57	3	57.447
FB_0546491_L3_PA	fp03_57	3	57.447
FB_0546502_L3_PA	fp03_57	3	57.447
FB_0546506_L3_PA	fp03_57	3	57.447
FB_0546478_L3_PA	fp03_57	3	57.447
FB_0546485_L3_PA	fp03_57	3	57.447
FB_0546089_L3_PA	fp03_58	3	58.446
FB_0547161_L3_PA	fp03_58	3	58.446
FB_0546811_L3_PA	fp03_58	3	58.446
RB_33670772_L3_PA	fp03_58	3	58.446
FB_0548710_L3_PA	fp03_58	3	58.446
FB_0010613_L10_PA	fp03_58	3	58.446
FB_0551755_L3_PA	fp03_58	3	58.446
FB_1118253_L9_54_3	fp03_58	3	58.446
FB_1090245_L3_83_2	fp03_58	3	58.446
FB_0810272_L9_PA	fp03_58	3	58.446
FB_0810261_L9_PA	fp03_58	3	58.446
FB_0010610_L10_PA	fp03_59	3	59.565
FB_0548717_L3_PA	fp03_59	3	59.565
GD_01329_L3_PA	fp03_59	3	59.565
RB_33286299_L3_PA	fp03_59	3	59.565
FB_0011074_L10_PA	fp03_59	3	59.565
FB_0011104_L10_PA	fp03_59	3	59.565

FB_0011075_L10_PA	fp03_59	3	59.565
FB_0974551_L3_56_1	fp03_59	3	59.565
FB_0974556_L3_56_1	fp03_59	3	59.565
FB_0011101_L10_PA	fp03_59	3	59.565
FB_0011077_L10_PA	fp03_59	3	59.565
FB_0010624_L10_PA	fp03_59	3	59.565
RB_32737388_L3_PA	fp03_59	3	59.565
FB_0010622_L10_PA	fp03_59	3	59.565
FB_0010626_L10_PA	fp03_59	3	59.565
FB_0551735_L3_PA	fp03_59	3	59.565
FB_0011059_L10_PA	fp03_59	3	59.565
FB_0810295_L9_PA	fp03_59	3	59.565
GD_01014_L3_PA	fp03_59	3	59.565
FB_0548708_L3_PA	fp03_59	3	59.565
FB_0010630_L10_PA	fp03_59	3	59.565
FB_0010620_L10_PA	fp03_60	3	60.632
FB_0551729_L3_PA	fp03_60	3	60.632
FB_0546805_L3_PA	fp03_60	3	60.632
FB_0547650_L3_PA	fp03_60	3	60.632
FB_0551734_L3_PA	fp03_60	3	60.632
RB_33672910_L3_PA	fp03_61	3	61.594
FB_0550349_L3_PA	fp03_61	3	61.594
FB_1090256_L3_83_2	fp03_61	3	61.594
RB_34437600_L3_101_3	fp03_61	3	61.594
FB_0548155_L3_PA	fp03_61	3	61.594
FB_1106695_L9_50_2	fp03_61	3	61.594
FB_0810256_L9_PA	fp03_61	3	61.594
FB_1106694_L9_50_2	fp03_61	3	61.594
FB_1118258_L9_54_3	fp03_61	3	61.594
FB_0810254_L9_PA	fp03_61	3	61.594
FB_1118251_L9_54_3	fp03_61	3	61.594
FB_1118250_L9_54_3	fp03_61	3	61.594
FB_0810253_L9_PA	fp03_62	3	62.875
FB_1090254_L3_83_2	fp03_62	3	62.875
FB_0975465_L3_59_1	fp03_62	3	62.875
FB_0549670_L3_PA	fp03_62	3	62.875
FB_1090287_L3_83_2	fp03_62	3	62.875
FB_0552121_L3_PA	fp03_62	3	62.875
GD_02030_L3_PA	fp03_62	3	62.875
FB_0549674_L3_PA	fp03_62	3	62.875
FB_0552103_L3_PA	fp03_62	3	62.875
FB_1090288_L3_83_2	fp03_62	3	62.875
RB_34442034_L3_PA	fp03_62	3	62.875
FB_0552107_L3_PA	fp03_62	3	62.875
RB_36486314_L3_PA	fp03_62	3	62.875
FB_1090246_L3_83_2	fp03_62	3	62.875

RB_34459777_L3_PA	fp03_62	3	62.875
FB_1118252_L9_54_3	fp03_62	3	62.875
FB_0551724_L3_PA	fp03_63	3	63.52
FB_0550350_L3_PA	fp03_64	3	64.286
FB_0975467_L3_59_1	fp03_64	3	64.286
FB_0550351_L3_PA	fp03_64	3	64.286
FB_0552080_L3_PA	fp03_64	3	64.286
FB_0552104_L3_PA	fp03_64	3	64.286
RB_36483536_L3_PA	fp03_64	3	64.286
FB_0975460_L3_59_1	fp03_64	3	64.286
FB_0552082_L3_PA	fp03_64	3	64.286
FB_0552782_L3_PA	fp03_64	3	64.286
FB_0552081_L3_PA	fp03_64	3	64.286
RB_36468422_L3_PA	fp03_65	3	65.348
RB_36768517_L3_PA	fp03_65	3	65.348
FB_0553327_L3_PA	fp03_65	3	65.348
FB_0553338_L3_PA	fp03_65	3	65.348
FB_0976063_L3_59_1	fp03_65	3	65.348
FB_0976061_L3_59_1	fp03_65	3	65.348
GD_00664_L3_PA	fp03_65	3	65.348
RB_37110581_L3_PA	fp03_65	3	65.348
RB_37144010_L3_PA	fp03_65	3	65.348
FB_0553336_L3_PA	fp03_65	3	65.348
FB_0552771_L3_PA	fp03_65	3	65.348
RB_37496618_L3_PA	fp03_67	3	67.837
FB_0553343_L3_PA	fp03_67	3	67.837
FB_1090550_L3_85_2	fp03_67	3	67.837
FB_0552775_L3_PA	fp03_67	3	67.837
RB_36780856_L3_PA	fp03_67	3	67.837
FB_0553340_L3_PA	fp03_67	3	67.837
FB_0552753_L3_PA	fp03_67	3	67.837
FB_0553342_L3_PA	fp03_67	3	67.837
RB_37069303_L3_PA	fp03_67	3	67.837
FB_0553333_L3_PA	fp03_67	3	67.837
FB_0553348_L3_PA	fp03_67	3	67.837
FB_0552734_L3_PA	fp03_67	3	67.837
FB_0553344_L3_PA	fp03_67	3	67.837
RB_37548828_L3_PA	fp03_69	3	69.497
FB_0554355_L3_PA	fp03_69	3	69.497
FB_0554357_L3_PA	fp03_69	3	69.497
RB_37545191_L3_PA	fp03_69	3	69.497
FB_0554347_L3_PA	fp03_69	3	69.497
GD_01822_L3_PA	fp03_69	3	69.497
FB_1090645_L3_85_2	fp03_69	3	69.497
FB_0554339_L3_PA	fp03_70	3	70.587
FB_0554341_L3_PA	fp03_70	3	70.587

FB_0554332_L3_PA	fp03_70	3	70.587
FB_0554331_L3_PA	fp03_70	3	70.587
FB_0554340_L3_PA	fp03_70	3	70.587
FB_0554981_L3_PA	fp03_70	3	70.587
FB_0554983_L3_PA	fp03_70	3	70.587
RB_38243554_L3_PA	fp03_70	3	70.587
RB_38257090_L3_PA	fp03_71	3	71.899
FB_0555681_L3_PA	fp03_72	3	72.132
FB_0557669_L3_PA	fp03_72	3	72.132
FB_0556161_L3_PA	fp03_72	3	72.132
FB_0557649_L3_PA	fp03_72	3	72.132
FB_0976587_L3_60_1	fp03_72	3	72.132
FB_1090863_L3_86_2	fp03_72	3	72.132
GD_01604_L3_86_2	fp03_72	3	72.132
FB_0556179_L3_PA	fp03_72	3	72.132
FB_1090865_L3_86_2	fp03_72	3	72.132
FB_0556174_L3_PA	fp03_72	3	72.132
FB_0976351_L3_60_1	fp03_72	3	72.132
FB_1090867_L3_86_2	fp03_72	3	72.132
FB_0554488_L3_PA	fp03_72	3	72.132
FB_0556152_L3_PA	fp03_72	3	72.132
GD_00319_L3_PA	fp03_72	3	72.132
FB_0554546_L3_PA	fp03_72	3	72.132
FB_0556171_L3_PA	fp03_72	3	72.132
FB_0976350_L3_60_1	fp03_72	3	72.132
FB_0557651_L3_PA	fp03_72	3	72.132
FB_0557658_L3_PA	fp03_72	3	72.132
FB_0557652_L3_PA	fp03_72	3	72.132
FB_1090859_L3_86_2	fp03_72	3	72.132
FB_0556155_L3_PA	fp03_72	3	72.132
FB_0556864_L3_PA	fp03_72	3	72.132
FB_0556901_L3_PA	fp03_72	3	72.132
FB_1090857_L3_86_2	fp03_72	3	72.132
RB_38459727_L3_PA	fp03_72	3	72.132
FB_0557663_L3_PA	fp03_72	3	72.132
FB_0556866_L3_PA	fp03_72	3	72.132
FB_0976353_L3_60_1	fp03_72	3	72.132
FB_0557678_L3_PA	fp03_72	3	72.132
FB_0554591_L3_PA	fp03_72	3	72.132
FB_0556882_L3_PA	fp03_72	3	72.132
FB_0554586_L3_PA	fp03_72	3	72.132
RB_38252402_L3_PA	fp03_72	3	72.132
RB_38273471_L3_PA	fp03_73	3	73.572
FB_0555705_L3_PA	fp03_73	3	73.572
FB_0555682_L3_PA	fp03_73	3	73.572
FB_0797940_L9_PA	fp04_00	4	0.05

FB_0797504_L9_PA	fp04_00	4	0.05
FB_0797213_L9_PA	fp04_00	4	0.05
FB_0796952_L9_PA	fp04_00	4	0.05
FB_0797211_L9_PA	fp04_00	4	0.05
FB_1045751_L9_25_1	fp04_00	4	0.05
FB_0797221_L9_PA	fp04_00	4	0.05
FB_0797520_L9_PA	fp04_00	4	0.05
FB_0797947_L9_PA	fp04_00	4	0.05
FB_0797233_L9_PA	fp04_00	4	0.05
FB_0464433_L2_PA	fp04_00	4	0.05
FB_0796949_L9_PA	fp04_00	4	0.05
FB_0797528_L9_PA	fp04_00	4	0.05
FB_1046081_L9_25_1	fp04_00	4	0.05
FB_0797511_L9_PA	fp04_00	4	0.05
FB_1045741_L9_25_1	fp04_00	4	0.05
FB_0464430_L2_PA	fp04_00	4	0.05
FB_0797232_L9_PA	fp04_00	4	0.05
FB_1045743_L9_25_1	fp04_00	4	0.05
FB_0797965_L9_PA	fp04_00	4	0.05
FB_0797932_L9_PA	fp04_00	4	0.05
FB_0797934_L9_PA	fp04_00	4	0.05
FB_1046079_L9_25_1	fp04_00	4	0.05
FB_1046101_L9_25_1	fp04_00	4	0.05
FB_0797930_L9_PA	fp04_00	4	0.05
FB_1045736_L9_25_1	fp04_00	4	0.05
FB_0797507_L9_PA	fp04_00	4	0.05
FB_0797962_L9_PA	fp04_00	4	0.05
FB_0797218_L9_PA	fp04_00	4	0.05
FB_1046078_L9_25_1	fp04_00	4	0.05
FB_1045734_L9_25_1	fp04_00	4	0.05
FB_0797952_L9_PA	fp04_00	4	0.05
FB_0796947_L9_PA	fp04_00	4	0.05
FB_0904741_L15_48_1	fp04_01	4	1.351
FB_0795162_L9_PA	fp04_01	4	1.351
FB_1046084_L9_25_1	fp04_02	4	2.409
FB_0795169_L9_PA	fp04_02	4	2.409
FB_0795854_L9_PA	fp04_02	4	2.409
FB_0795195_L9_PA	fp04_02	4	2.409
FB_0795847_L9_PA	fp04_02	4	2.409
FB_0795168_L9_PA	fp04_02	4	2.409
FB_0904796_L15_48_1	fp04_02	4	2.409
FB_0795165_L9_PA	fp04_02	4	2.409
FB_0795840_L9_PA	fp04_02	4	2.409
FB_0795858_L9_PA	fp04_02	4	2.409
FB_0795199_L9_PA	fp04_02	4	2.409
FB_0905063_L15_48_1	fp04_04	4	4.859

FB_0798152_L9_PA	fp04_04	4	4.859
FB_0905064_L15_48_1	fp04_04	4	4.859
FB_0798158_L9_PA	fp04_04	4	4.859
FB_0798165_L9_PA	fp04_04	4	4.859
FB_0940643_L1_45_1	fp04_04	4	4.859
FB_0798159_L9_PA	fp04_06	4	6.527
FB_0798173_L9_PA	fp04_06	4	6.527
RB_849833_L4_PA	fp04_07	4	7.32
FB_0558624_L4_PA	fp04_07	4	7.32
FB_0558611_L4_PA	fp04_07	4	7.32
FB_0558629_L4_PA	fp04_07	4	7.32
RB_830930_L4_PA	fp04_07	4	7.32
FB_0558628_L4_PA	fp04_07	4	7.32
FB_0558614_L4_PA	fp04_07	4	7.32
FB_0977372_L4_14_1	fp04_07	4	7.32
FB_0798161_L9_PA	fp04_07	4	7.32
FB_1091001_L4_26_2	fp04_09	4	9.758
FB_0559404_L4_PA	fp04_09	4	9.758
FB_0559406_L4_PA	fp04_09	4	9.758
FB_0559423_L4_PA	fp04_09	4	9.758
FB_0559416_L4_PA	fp04_09	4	9.758
FB_0559419_L4_PA	fp04_09	4	9.758
FB_0559414_L4_PA	fp04_09	4	9.758
FB_0559825_L4_PA	fp04_10	4	10.963
FB_0559807_L4_PA	fp04_10	4	10.963
FB_0559805_L4_PA	fp04_10	4	10.963
FB_0559833_L4_PA	fp04_10	4	10.963
FB_0246379_L14_PA	fp04_10	4	10.963
FB_0559829_L4_PA	fp04_10	4	10.963
FB_0559832_L4_PA	fp04_12	4	12.547
FB_0352718_L16_PA	fp04_12	4	12.547
FB_0560731_L4_PA	fp04_14	4	14.302
FB_0353160_L16_PA	fp04_14	4	14.302
FB_0560739_L4_PA	fp04_14	4	14.302
FB_0560738_L4_PA	fp04_14	4	14.302
FB_0353164_L16_PA	fp04_14	4	14.302
FB_1091117_L4_27_2	fp04_14	4	14.302
FB_1091121_L4_27_2	fp04_14	4	14.302
FB_1091119_L4_27_2	fp04_14	4	14.302
FB_0562311_L4_PA	fp04_14	4	14.302
RB_2760781_L4_PA	fp04_14	4	14.302
FB_1091270_L4_28_2	fp04_14	4	14.302
FB_0560740_L4_PA	fp04_14	4	14.302
FB_0276809_L15_PA	fp04_15	4	15.954
FB_0276810_L15_PA	fp04_15	4	15.954
FB_1091276_L4_28_2	fp04_15	4	15.954

FB_1091278_L4_28_2	fp04_15	4	15.954
FB_0276835_L15_PA	fp04_15	4	15.954
FB_0563198_L4_PA	fp04_16	4	16.499
FB_1115390_L4_39_3	fp04_16	4	16.499
FB_0561837_L4_PA	fp04_16	4	16.499
FB_0561832_L4_PA	fp04_16	4	16.499
RB_3942532_L4_PA	fp04_16	4	16.499
FB_1115384_L4_39_3	fp04_16	4	16.499
FB_0561843_L4_PA	fp04_16	4	16.499
FB_1115385_L4_39_3	fp04_16	4	16.499
FB_1091272_L4_28_2	fp04_17	4	17.716
FB_0353159_L16_PA	fp04_17	4	17.716
RB_4625426_L4_PA	fp04_17	4	17.716
FB_0563202_L4_PA	fp04_17	4	17.716
FB_0562324_L4_PA	fp04_17	4	17.716
FB_0562306_L4_PA	fp04_17	4	17.716
FB_0562298_L4_PA	fp04_17	4	17.716
FB_0562289_L4_PA	fp04_17	4	17.716
FB_1091277_L4_28_2	fp04_17	4	17.716
RB_4621611_L4_PA	fp04_17	4	17.716
RB_4605949_L4_PA	fp04_17	4	17.716
RB_4580974_L4_PA	fp04_18	4	18.78
RB_4578560_L4_PA	fp04_18	4	18.78
FB_0563187_L4_PA	fp04_18	4	18.78
RB_4838321_L4_PA	fp04_18	4	18.78
FB_0563348_L4_PA	fp04_18	4	18.78
FB_0563191_L4_PA	fp04_18	4	18.78
FB_0563353_L4_PA	fp04_18	4	18.78
FB_0563366_L4_PA	fp04_18	4	18.78
RB_4635144_L4_PA	fp04_18	4	18.78
FB_0563369_L4_PA	fp04_18	4	18.78
FB_0563367_L4_PA	fp04_18	4	18.78
FB_0979603_L4_16_1	fp04_18	4	18.78
FB_0979340_L4_16_1	fp04_18	4	18.78
RB_4849635_L4_PA	fp04_19	4	19.508
FB_0005958_L10_PA	fp04_19	4	19.508
FB_0564738_L4_PA	fp04_19	4	19.508
FB_0564250_L4_PA	fp04_19	4	19.508
FB_0564737_L4_PA	fp04_19	4	19.508
FB_0564745_L4_PA	fp04_19	4	19.508
FB_0564749_L4_PA	fp04_19	4	19.508
FB_0564748_L4_PA	fp04_19	4	19.508
FB_0825855_L10_32_1	fp04_20	4	20.299
FB_0005966_L10_PA	fp04_20	4	20.299
FB_0005974_L10_PA	fp04_20	4	20.299
FB_0005963_L10_PA	fp04_20	4	20.299

FB_0825851_L10_32_1	fp04_20	4	20.299
FB_0005956_L10_PA	fp04_20	4	20.299
FB_1083396_L2_38_2	fp04_20	4	20.299
FB_1061181_L12_61_2	fp04_20	4	20.299
FB_0663393_L6_PA	fp04_20	4	20.299
RB_5920541_L4_17_1	fp04_20	4	20.299
FB_0005980_L10_PA	fp04_20	4	20.299
FB_0005957_L10_PA	fp04_20	4	20.299
FB_0564740_L4_PA	fp04_20	4	20.299
FB_0563362_L4_PA	fp04_21	4	21.549
RB_2768879_L4_27_2	fp04_21	4	21.549
FB_0566756_L4_PA	fp04_21	4	21.549
FB_0826185_L10_32_1	fp04_22	4	22.057
FB_0826219_L10_32_1	fp04_22	4	22.057
FB_0826218_L10_32_1	fp04_22	4	22.057
FB_0663333_L6_PA	fp04_22	4	22.057
FB_0826221_L10_32_1	fp04_22	4	22.057
FB_1061169_L12_61_2	fp04_22	4	22.057
FB_0663356_L6_PA	fp04_22	4	22.057
FB_0826186_L10_32_1	fp04_22	4	22.057
FB_0826183_L10_32_1	fp04_22	4	22.057
FB_1061167_L12_61_2	fp04_22	4	22.057
FB_1061180_L12_61_2	fp04_22	4	22.057
FB_1061168_L12_61_2	fp04_22	4	22.057
FB_0663352_L6_PA	fp04_22	4	22.057
FB_0826200_L10_32_1	fp04_22	4	22.057
FB_0826180_L10_32_1	fp04_22	4	22.057
FB_0663330_L6_PA	fp04_22	4	22.057
RB_4844832_L4_PA	fp04_22	4	22.057
FB_0702487_L7_PA	fp04_22	4	22.057
FB_0663394_L6_PA	fp04_23	4	23.449
FB_0980439_L4_19_1	fp04_23	4	23.449
FB_0615599_L5_PA	fp04_23	4	23.449
RB_5884301_L4_17_1	fp04_23	4	23.449
FB_0811632_L9_PA	fp04_23	4	23.449
FB_0811258_L9_PA	fp04_23	4	23.449
FB_0812208_L9_PA	fp04_23	4	23.449
FB_0565094_L4_PA	fp04_23	4	23.449
FB_0812226_L9_PA	fp04_23	4	23.449
FB_0812238_L9_PA	fp04_23	4	23.449
FB_0980068_L4_17_1	fp04_23	4	23.449
FB_0565104_L4_PA	fp04_23	4	23.449
FB_1083405_L2_38_2	fp04_23	4	23.449
FB_0565138_L4_PA	fp04_23	4	23.449
FB_0566775_L4_PA	fp04_23	4	23.449
FB_0565086_L4_PA	fp04_23	4	23.449

FB_1083414_L2_38_2	fp04_23	4	23.449
FB_0567493_L4_PA	fp04_23	4	23.449
RB_7204908_L4_PA	fp04_23	4	23.449
FB_0565434_L4_PA	fp04_23	4	23.449
FB_0980060_L4_17_1	fp04_23	4	23.449
FB_0812223_L9_PA	fp04_23	4	23.449
FB_0812237_L9_PA	fp04_23	4	23.449
FB_0812225_L9_PA	fp04_23	4	23.449
FB_0811256_L9_PA	fp04_23	4	23.449
FB_0615594_L5_PA	fp04_24	4	24.425
FB_0569649_L4_PA	fp04_24	4	24.425
FB_0980065_L4_17_1	fp04_24	4	24.425
FB_0663376_L6_PA	fp04_24	4	24.425
FB_0569950_L4_PA	fp04_24	4	24.425
FB_0567469_L4_PA	fp04_24	4	24.425
FB_0811264_L9_PA	fp04_24	4	24.425
FB_0615584_L5_PA	fp04_24	4	24.425
RB_7202056_L4_PA	fp04_24	4	24.425
FB_0812239_L9_PA	fp04_24	4	24.425
FB_0567480_L4_PA	fp04_24	4	24.425
FB_0565420_L4_PA	fp04_24	4	24.425
FB_0615598_L5_PA	fp04_24	4	24.425
FB_0569689_L4_PA	fp04_24	4	24.425
FB_0811643_L9_PA	fp04_24	4	24.425
FB_0811248_L9_PA	fp04_24	4	24.425
FB_0567463_L4_PA	fp04_24	4	24.425
FB_0568386_L4_PA	fp04_24	4	24.425
FB_0568365_L4_PA	fp04_24	4	24.425
FB_0712574_L7_PA	fp04_24	4	24.425
FB_0566765_L4_PA	fp04_24	4	24.425
FB_0811631_L9_PA	fp04_24	4	24.425
FB_0567548_L4_PA	fp04_24	4	24.425
FB_0569675_L4_PA	fp04_24	4	24.425
FB_0567531_L4_PA	fp04_24	4	24.425
FB_0811624_L9_PA	fp04_24	4	24.425
FB_0811291_L9_PA	fp04_24	4	24.425
RB_5930421_L4_17_1	fp04_24	4	24.425
FB_0566754_L4_PA	fp04_24	4	24.425
FB_0569932_L4_PA	fp04_24	4	24.425
FB_0566774_L4_PA	fp04_24	4	24.425
FB_0565419_L4_PA	fp04_24	4	24.425
FB_0569953_L4_PA	fp04_24	4	24.425
FB_0569930_L4_PA	fp04_24	4	24.425
FB_0981505_L4_20_1	fp04_25	4	25.356
FB_0981503_L4_20_1	fp04_25	4	25.356
FB_0615586_L5_PA	fp04_25	4	25.356

FB_0981290_L4_20_1	fp04_25	4	25.356
RB_7180850_L4_PA	fp04_25	4	25.356
FB_0572741_L4_PA	fp04_25	4	25.356
FB_0569650_L4_PA	fp04_25	4	25.356
FB_0569648_L4_PA	fp04_25	4	25.356
FB_0567466_L4_PA	fp04_25	4	25.356
FB_0980766_L4_19_1	fp04_25	4	25.356
GD_01886_L4_PA	fp04_25	4	25.356
FB_0570692_L4_PA	fp04_25	4	25.356
GD_00363_L4_PA	fp04_25	4	25.356
FB_0572022_L4_PA	fp04_25	4	25.356
FB_0980763_L4_19_1	fp04_25	4	25.356
FB_0572009_L4_PA	fp04_25	4	25.356
FB_0572726_L4_PA	fp04_25	4	25.356
FB_0572727_L4_PA	fp04_25	4	25.356
FB_0570639_L4_PA	fp04_26	4	26.449
FB_0562308_L4_PA	fp04_26	4	26.449
FB_0980862_L4_20_1	fp04_26	4	26.449
FB_0573244_L4_PA	fp04_26	4	26.449
FB_0573208_L4_PA	fp04_26	4	26.449
FB_0573237_L4_PA	fp04_26	4	26.449
RB_12296545_L4_PA	fp04_27	4	27.387
FB_0573888_L4_PA	fp04_27	4	27.387
FB_0573850_L4_PA	fp04_28	4	28.279
FB_0573236_L4_PA	fp04_28	4	28.279
FB_0573245_L4_PA	fp04_28	4	28.279
FB_0575070_L4_PA	fp04_28	4	28.279
FB_0573890_L4_PA	fp04_29	4	29.665
FB_0573855_L4_PA	fp04_29	4	29.665
FB_0574574_L4_PA	fp04_29	4	29.665
FB_0982337_L4_22_1	fp04_29	4	29.665
FB_0575069_L4_PA	fp04_29	4	29.665
FB_0575085_L4_PA	fp04_29	4	29.665
FB_0982338_L4_22_1	fp04_29	4	29.665
FB_0575073_L4_PA	fp04_29	4	29.665
FB_0573886_L4_PA	fp04_29	4	29.665
FB_0573224_L4_PA	fp04_30	4	30.159
FB_0574570_L4_PA	fp04_31	4	31.251
FB_0575775_L4_PA	fp04_31	4	31.251
FB_0576408_L4_PA	fp04_31	4	31.251
RB_13524873_L4_PA	fp04_31	4	31.251
RB_14084215_L4_PA	fp04_31	4	31.251
FB_0576391_L4_PA	fp04_31	4	31.251
FB_0575748_L4_PA	fp04_31	4	31.251
FB_0576394_L4_PA	fp04_31	4	31.251
FB_0575762_L4_PA	fp04_31	4	31.251

RB_13073783_L4_22_1	fp04_31	4	31.251
RB_14065892_L4_PA	fp04_31	4	31.251
FB_0575077_L4_PA	fp04_31	4	31.251
FB_0575075_L4_PA	fp04_31	4	31.251
RB_13312189_L4_PA	fp04_31	4	31.251
RB_13348603_L4_PA	fp04_31	4	31.251
FB_0574572_L4_PA	fp04_32	4	32.376
RB_14061369_L4_PA	fp04_32	4	32.376
FB_0576370_L4_PA	fp04_32	4	32.376
GD_01095_L4_PA	fp04_32	4	32.376
FB_0576373_L4_PA	fp04_32	4	32.376
FB_0576365_L4_PA	fp04_32	4	32.376
FB_0576371_L4_PA	fp04_32	4	32.376
FB_0575776_L4_PA	fp04_32	4	32.376
FB_0982990_L4_22_1	fp04_33	4	33.443
FB_0577067_L4_PA	fp04_33	4	33.443
FB_0577051_L4_PA	fp04_33	4	33.443
FB_0983006_L4_22_1	fp04_33	4	33.443
FB_0577063_L4_PA	fp04_33	4	33.443
FB_0982992_L4_22_1	fp04_33	4	33.443
FB_0578309_L4_PA	fp04_33	4	33.443
FB_0577066_L4_PA	fp04_33	4	33.443
FB_0577060_L4_PA	fp04_33	4	33.443
FB_0576389_L4_PA	fp04_33	4	33.443
FB_0578310_L4_PA	fp04_33	4	33.443
FB_0577535_L4_PA	fp04_33	4	33.443
FB_0577563_L4_PA	fp04_34	4	34.455
FB_0577531_L4_PA	fp04_34	4	34.455
FB_0982991_L4_22_1	fp04_34	4	34.455
GD_01292_L4_PA	fp04_34	4	34.455
FB_0577555_L4_PA	fp04_34	4	34.455
FB_0577533_L4_PA	fp04_34	4	34.455
RB_14580745_L4_PA	fp04_34	4	34.455
FB_0577545_L4_PA	fp04_34	4	34.455
RB_14559093_L4_PA	fp04_34	4	34.455
FB_0578277_L4_PA	fp04_34	4	34.455
FB_0578266_L4_PA	fp04_34	4	34.455
FB_0578272_L4_PA	fp04_34	4	34.455
FB_0578271_L4_PA	fp04_34	4	34.455
FB_0578306_L4_PA	fp04_34	4	34.455
RB_16134794_L4_PA	fp04_35	4	35.865
FB_0581122_L4_PA	fp04_35	4	35.865
RB_16719136_L4_PA	fp04_35	4	35.865
FB_0579320_L4_PA	fp04_35	4	35.865
FB_0577540_L4_PA	fp04_35	4	35.865
GD_01777_L4_PA	fp04_35	4	35.865

RB_16050155_L4_PA	fp04_36	4	36.549
RB_16168045_L4_PA	fp04_36	4	36.549
RB_16093765_L4_PA	fp04_36	4	36.549
FB_0579793_L4_PA	fp04_36	4	36.549
FB_0579292_L4_PA	fp04_36	4	36.549
FB_0983814_L4_23_1	fp04_36	4	36.549
RB_16090074_L4_PA	fp04_36	4	36.549
FB_0983811_L4_23_1	fp04_36	4	36.549
FB_0983816_L4_23_1	fp04_36	4	36.549
RB_16191177_L4_PA	fp04_36	4	36.549
FB_0983813_L4_23_1	fp04_36	4	36.549
RB_16067316_L4_PA	fp04_36	4	36.549
FB_0579789_L4_PA	fp04_36	4	36.549
FB_0579287_L4_PA	fp04_36	4	36.549
RB_16184302_L4_PA	fp04_36	4	36.549
FB_0579405_L4_PA	fp04_36	4	36.549
FB_0579790_L4_PA	fp04_36	4	36.549
FB_0579290_L4_PA	fp04_37	4	37.708
FB_1092631_L4_34_2	fp04_37	4	37.708
FB_0579296_L4_PA	fp04_37	4	37.708
FB_0583225_L4_PA	fp04_37	4	37.708
FB_0583221_L4_PA	fp04_37	4	37.708
FB_0730412_L8_PA	fp04_37	4	37.708
FB_0730433_L8_PA	fp04_37	4	37.708
FB_0730481_L8_PA	fp04_37	4	37.708
FB_0580722_L4_PA	fp04_37	4	37.708
FB_0582613_L4_PA	fp04_37	4	37.708
FB_0730461_L8_PA	fp04_37	4	37.708
FB_0983762_L4_23_1	fp04_37	4	37.708
FB_0581642_L4_PA	fp04_37	4	37.708
RB_16719789_L4_PA	fp04_37	4	37.708
FB_0983946_L4_23_1	fp04_38	4	38.42
FB_0983938_L4_23_1	fp04_38	4	38.42
FB_0581648_L4_PA	fp04_38	4	38.42
FB_0863169_L12_45_1	fp04_38	4	38.42
FB_0984223_L4_23_1	fp04_38	4	38.42
FB_0581116_L4_PA	fp04_38	4	38.42
FB_0582198_L4_PA	fp04_38	4	38.42
FB_0581623_L4_PA	fp04_38	4	38.42
FB_0581643_L4_PA	fp04_38	4	38.42
FB_0582209_L4_PA	fp04_38	4	38.42
FB_0581120_L4_PA	fp04_38	4	38.42
FB_0581130_L4_PA	fp04_38	4	38.42
RB_18418044_L4_PA	fp04_38	4	38.42
FB_0581633_L4_PA	fp04_38	4	38.42
FB_0581628_L4_PA	fp04_38	4	38.42

GD_00321_L4_PA	fp04_38	4	38.42
FB_0581135_L4_PA	fp04_38	4	38.42
FB_0581112_L4_PA	fp04_38	4	38.42
FB_0863175_L12_45_1	fp04_38	4	38.42
FB_0983939_L4_23_1	fp04_38	4	38.42
FB_0983936_L4_23_1	fp04_38	4	38.42
FB_0863172_L12_45_1	fp04_38	4	38.42
RB_18428562_L4_PA	fp04_38	4	38.42
RB_18544856_L4_PA	fp04_38	4	38.42
FB_0581139_L4_PA	fp04_38	4	38.42
FB_0581142_L4_PA	fp04_38	4	38.42
FB_0863177_L12_45_1	fp04_38	4	38.42
FB_1092629_L4_34_2	fp04_38	4	38.42
FB_0984217_L4_23_1	fp04_39	4	39.232
RB_18756586_L4_24_1	fp04_39	4	39.232
RB_18750621_L4_24_1	fp04_39	4	39.232
FB_1092697_L4_35_2	fp04_39	4	39.232
FB_0984230_L4_23_1	fp04_39	4	39.232
FB_0984208_L4_23_1	fp04_39	4	39.232
GD_02277_L4_PA	fp04_39	4	39.232
FB_0984211_L4_23_1	fp04_39	4	39.232
FB_0984215_L4_23_1	fp04_39	4	39.232
FB_0583214_L4_PA	fp04_40	4	40.215
GD_00148_L4_24_1	fp04_40	4	40.215
FB_0583233_L4_PA	fp04_40	4	40.215
GD_00324_L4_PA	fp04_40	4	40.215
FB_0583247_L4_PA	fp04_40	4	40.215
FB_0583229_L4_PA	fp04_40	4	40.215
FB_0584669_L4_PA	fp04_42	4	42.229
RB_19752961_L4_PA	fp04_42	4	42.229
RB_18748448_L4_24_1	fp04_42	4	42.229
FB_0584680_L4_PA	fp04_42	4	42.229
RB_19730224_L4_PA	fp04_42	4	42.229
FB_0867968_L12_46_1	fp04_42	4	42.229
RB_18754232_L4_24_1	fp04_42	4	42.229
FB_0867977_L12_46_1	fp04_42	4	42.229
FB_0583852_L4_PA	fp04_42	4	42.229
FB_0584657_L4_PA	fp04_42	4	42.229
FB_0583855_L4_PA	fp04_42	4	42.229
RB_19520932_L4_PA	fp04_42	4	42.229
GD_00881_L4_PA	fp04_42	4	42.229
FB_0583227_L4_PA	fp04_42	4	42.229
RB_19480551_L4_PA	fp04_42	4	42.229
FB_0584693_L4_PA	fp04_42	4	42.229
FB_0584687_L4_PA	fp04_42	4	42.229
RB_18771771_L4_24_1	fp04_42	4	42.229

FB_0985132_L4_24_1	fp04_42	4	42.229
RB_19727005_L4_PA	fp04_42	4	42.229
FB_0985133_L4_24_1	fp04_42	4	42.229
FB_0584684_L4_PA	fp04_42	4	42.229
FB_0584659_L4_PA	fp04_43	4	43.606
FB_0586209_L4_PA	fp04_45	4	45.465
FB_0586236_L4_PA	fp04_46	4	46.519
FB_0586211_L4_PA	fp04_46	4	46.519
FB_0586232_L4_PA	fp04_46	4	46.519
FB_0586217_L4_PA	fp04_46	4	46.519
FB_0587135_L4_PA	fp04_46	4	46.519
RB_20463993_L4_PA	fp04_47	4	47.718
RB_20929090_L4_PA	fp04_47	4	47.718
FB_0587707_L4_PA	fp04_47	4	47.718
FB_0587139_L4_PA	fp04_48	4	48.263
GD_01773_L4_PA	fp04_48	4	48.263
FB_0587138_L4_PA	fp04_48	4	48.263
FB_0586245_L4_PA	fp04_48	4	48.263
RB_20552378_L4_35_2	fp04_48	4	48.263
FB_0586877_L4_PA	fp04_48	4	48.263
RB_20512923_L4_PA	fp04_48	4	48.263
FB_0586212_L4_PA	fp04_48	4	48.263
FB_0586221_L4_PA	fp04_48	4	48.263
RB_20963489_L4_PA	fp04_50	4	50.632
FB_0587735_L4_PA	fp04_50	4	50.632
FB_0985973_L4_24_1	fp04_50	4	50.632
FB_0587725_L4_PA	fp04_50	4	50.632
FB_0985962_L4_24_1	fp04_50	4	50.632
FB_0985972_L4_24_1	fp04_50	4	50.632
FB_0985966_L4_24_1	fp04_50	4	50.632
GD_00619_L4_PA	fp04_50	4	50.632
GD_01558_L4_PA	fp04_50	4	50.632
FB_0588480_L4_PA	fp04_50	4	50.632
FB_0588469_L4_PA	fp04_50	4	50.632
FB_0588457_L4_PA	fp04_50	4	50.632
FB_0588475_L4_PA	fp04_50	4	50.632
RB_21133802_L4_PA	fp04_50	4	50.632
RB_21163375_L4_PA	fp04_50	4	50.632
RB_21147726_L4_PA	fp04_50	4	50.632
RB_21176452_L4_PA	fp04_50	4	50.632
RB_21172343_L4_PA	fp04_50	4	50.632
FB_0588466_L4_PA	fp04_51	4	51.675
RB_20984611_L4_PA	fp04_51	4	51.675
RB_21956655_L4_24_1	fp04_51	4	51.675
FB_0986140_L4_24_1	fp04_51	4	51.675
RB_21422131_L4_PA	fp04_51	4	51.675

GD_01965_L4_24_1	fp04_51	4	51.675
RB_21456371_L4_PA	fp04_51	4	51.675
FB_0588458_L4_PA	fp04_52	4	52.701
FB_0589674_L4_PA	fp04_52	4	52.701
FB_0589681_L4_PA	fp04_52	4	52.701
FB_0589670_L4_PA	fp04_52	4	52.701
FB_0589660_L4_PA	fp04_52	4	52.701
FB_0589657_L4_PA	fp04_52	4	52.701
FB_0589677_L4_PA	fp04_52	4	52.701
FB_0589671_L4_PA	fp04_52	4	52.701
RB_22208785_L4_PA	fp04_53	4	53.441
RB_21774157_L4_PA	fp04_53	4	53.441
FB_0589672_L4_PA	fp04_53	4	53.441
FB_0986590_L4_24_1	fp04_53	4	53.441
FB_0591258_L4_PA	fp04_53	4	53.441
FB_0590207_L4_PA	fp04_54	4	54.85
RB_22741563_L4_PA	fp04_54	4	54.85
FB_0986579_L4_24_1	fp04_54	4	54.85
FB_0986600_L4_24_1	fp04_54	4	54.85
FB_0986582_L4_24_1	fp04_54	4	54.85
FB_0590205_L4_PA	fp04_54	4	54.85
FB_0590209_L4_PA	fp04_54	4	54.85
FB_0590704_L4_PA	fp04_54	4	54.85
FB_0986767_L4_24_1	fp04_54	4	54.85
FB_0590714_L4_PA	fp04_54	4	54.85
GD_00388_L4_PA	fp04_54	4	54.85
FB_0590721_L4_PA	fp04_54	4	54.85
FB_0589665_L4_PA	fp04_54	4	54.85
FB_0986764_L4_24_1	fp04_54	4	54.85
RB_23751350_L4_PA	fp04_54	4	54.85
RB_23867559_L4_PA	fp04_54	4	54.85
FB_0590199_L4_PA	fp04_54	4	54.85
FB_0591954_L4_PA	fp04_54	4	54.85
FB_0986886_L4_25_1	fp04_54	4	54.85
FB_0590201_L4_PA	fp04_54	4	54.85
GD_01026_L4_25_1	fp04_54	4	54.85
FB_0986758_L4_24_1	fp04_54	4	54.85
FB_0590700_L4_PA	fp04_54	4	54.85
FB_0986882_L4_25_1	fp04_54	4	54.85
FB_0590716_L4_PA	fp04_54	4	54.85
FB_0986762_L4_24_1	fp04_54	4	54.85
FB_0590190_L4_PA	fp04_54	4	54.85
FB_0986770_L4_24_1	fp04_54	4	54.85
FB_0590701_L4_PA	fp04_54	4	54.85
GD_00681_L4_PA	fp04_54	4	54.85
FB_0591703_L4_PA	fp04_54	4	54.85

FB_0591256_L4_PA	fp04_54	4	54.85
FB_0591947_L4_PA	fp04_54	4	54.85
RB_23837022_L4_PA	fp04_54	4	54.85
FB_0591255_L4_PA	fp04_54	4	54.85
RB_23735217_L4_PA	fp04_54	4	54.85
RB_23421514_L4_PA	fp04_54	4	54.85
FB_0591670_L4_PA	fp04_54	4	54.85
FB_0591266_L4_PA	fp04_54	4	54.85
FB_0590192_L4_PA	fp04_54	4	54.85
FB_0986603_L4_24_1	fp04_55	4	55.444
FB_0590194_L4_PA	fp04_55	4	55.444
RB_23354901_L4_PA	fp04_55	4	55.444
RB_23359599_L4_PA	fp04_55	4	55.444
RB_23383939_L4_25_1	fp04_55	4	55.444
FB_0986588_L4_24_1	fp04_55	4	55.444
RB_23839160_L4_PA	fp04_55	4	55.444
RB_24223602_L4_PA	fp04_55	4	55.444
FB_0592595_L4_PA	fp04_55	4	55.444
FB_0591953_L4_PA	fp04_55	4	55.444
RB_24133020_L4_PA	fp04_55	4	55.444
FB_0591290_L4_PA	fp04_55	4	55.444
RB_23921212_L4_25_1	fp04_55	4	55.444
FB_0591935_L4_PA	fp04_55	4	55.444
FB_0591685_L4_PA	fp04_56	4	56.699
RB_23824815_L4_PA	fp04_56	4	56.699
RB_25047666_L4_25_1	fp04_56	4	56.699
FB_0592590_L4_PA	fp04_56	4	56.699
RB_24104684_L4_25_1	fp04_56	4	56.699
GD_00283_L4_PA	fp04_56	4	56.699
FB_0592615_L4_PA	fp04_56	4	56.699
FB_0592593_L4_PA	fp04_56	4	56.699
FB_0987636_L4_25_1	fp04_56	4	56.699
RB_24228399_L4_PA	fp04_56	4	56.699
FB_0987652_L4_25_1	fp04_56	4	56.699
FB_0593790_L4_PA	fp04_56	4	56.699
RB_25043574_L4_25_1	fp04_56	4	56.699
FB_0592592_L4_PA	fp04_56	4	56.699
RB_24262161_L4_PA	fp04_56	4	56.699
FB_0593320_L4_PA	fp04_56	4	56.699
RB_24174303_L4_PA	fp04_56	4	56.699
FB_0593786_L4_PA	fp04_56	4	56.699
FB_0987721_L4_25_1	fp04_56	4	56.699
FB_0592602_L4_PA	fp04_56	4	56.699
GD_00136_L4_25_1	fp04_56	4	56.699
GD_00232_L4_PA	fp04_56	4	56.699
FB_0593798_L4_PA	fp04_56	4	56.699

RB_23902093_L4_PA	fp04_56	4	56.699
FB_0593777_L4_PA	fp04_56	4	56.699
FB_0593776_L4_PA	fp04_56	4	56.699
RB_24140264_L4_PA	fp04_56	4	56.699
GD_00523_L4_36_2	fp04_56	4	56.699
FB_0987720_L4_25_1	fp04_56	4	56.699
FB_0593780_L4_PA	fp04_56	4	56.699
FB_0593792_L4_PA	fp04_56	4	56.699
RB_24231394_L4_PA	fp04_56	4	56.699
FB_0593788_L4_PA	fp04_57	4	57.706
FB_0987734_L4_25_1	fp04_57	4	57.706
RB_25055592_L4_PA	fp04_57	4	57.706
MdFT2_L4_PA	fp04_58	4	58.64
FB_0987644_L4_25_1	fp04_58	4	58.64
FB_0987649_L4_25_1	fp04_58	4	58.64
RB_25012657_L4_PA	fp04_58	4	58.64
RB_25034592_L4_25_1	fp04_59	4	59.728
FB_0824878_L10_27_1	fp05_00	5	0
GD_00100_L5_PA	fp05_00	5	0
FB_0644556_L5_PA	fp05_00	5	0
RB_2474048_L5_32_1	fp05_00	5	0
FB_0664321_L6_PA	fp05_07	5	7.957
FB_0325717_L15_PA	fp05_08	5	8.52
FB_0645336_L5_PA	fp05_08	5	8.52
FB_0645481_L5_PA	fp05_08	5	8.52
FB_0325322_L15_PA	fp05_08	5	8.52
FB_0664309_L6_PA	fp05_08	5	8.52
FB_0664340_L6_PA	fp05_09	5	9.283
FB_0824843_L10_27_1	fp05_09	5	9.283
RB_37429382_L5_PA	fp05_09	5	9.283
FB_0824847_L10_27_1	fp05_09	5	9.283
FB_0645332_L5_PA	fp05_09	5	9.283
FB_0645329_L5_PA	fp05_09	5	9.283
FB_0531419_L3_PA	fp05_09	5	9.283
FB_0824877_L10_27_1	fp05_09	5	9.283
RB_36546287_L5_PA	fp05_09	5	9.283
FB_0645472_L5_PA	fp05_09	5	9.283
GD_02674_L5_PA	fp05_09	5	9.283
FB_0644563_L5_PA	fp05_09	5	9.283
FB_0645480_L5_PA	fp05_09	5	9.283
FB_0531424_L3_PA	fp05_09	5	9.283
RB_37469684_L5_PA	fp05_09	5	9.283
FB_0644558_L5_PA	fp05_09	5	9.283
FB_0824811_L10_27_1	fp05_09	5	9.283
FB_0644557_L5_PA	fp05_09	5	9.283
RB_36498250_L5_PA	fp05_09	5	9.283

RB_36487489_L5_PA	fp05_09	5	9.283
FB_0531416_L3_PA	fp05_09	5	9.283
FB_0645341_L5_PA	fp05_09	5	9.283
RB_36490942_L5_PA	fp05_09	5	9.283
FB_0324904_L15_PA	fp05_09	5	9.283
FB_0664349_L6_PA	fp05_09	5	9.283
FB_0325321_L15_PA	fp05_10	5	10.748
FB_0325330_L15_PA	fp05_10	5	10.748
FB_0325710_L15_PA	fp05_10	5	10.748
FB_0325706_L15_PA	fp05_10	5	10.748
FB_0822456_L9_PA	fp05_10	5	10.748
FB_0558305_L4_PA	fp05_10	5	10.748
FB_0324593_L15_PA	fp05_10	5	10.748
FB_0324606_L15_PA	fp05_10	5	10.748
FB_0822460_L9_PA	fp05_10	5	10.748
FB_0915270_L15_76_1	fp05_11	5	11.471
FB_0808617_L9_PA	fp05_11	5	11.471
FB_0825629_L10_31_1	fp05_11	5	11.471
FB_0627059_L5_PA	fp05_11	5	11.471
FB_0627054_L5_PA	fp05_11	5	11.471
FB_0807980_L9_PA	fp05_11	5	11.471
FB_0099980_L11_PA	fp05_11	5	11.471
FB_0808627_L9_PA	fp05_11	5	11.471
FB_0324933_L15_PA	fp05_11	5	11.471
RB_23203278_L5_PA	fp05_11	5	11.471
RB_36510795_L5_82_2	fp05_11	5	11.471
RB_36503731_L5_PA	fp05_11	5	11.471
FB_0626671_L5_PA	fp05_11	5	11.471
FB_0324580_L15_PA	fp05_11	5	11.471
FB_0324907_L15_PA	fp05_11	5	11.471
FB_0324598_L15_PA	fp05_11	5	11.471
FB_0807976_L9_PA	fp05_11	5	11.471
FB_0558282_L4_PA	fp05_11	5	11.471
FB_0807981_L9_PA	fp05_11	5	11.471
FB_0324599_L15_PA	fp05_11	5	11.471
FB_0558300_L4_PA	fp05_11	5	11.471
FB_0324607_L15_PA	fp05_11	5	11.471
FB_0324908_L15_PA	fp05_11	5	11.471
FB_0809220_L9_PA	fp05_11	5	11.471
RB_36495464_L5_PA	fp05_11	5	11.471
FB_0324586_L15_PA	fp05_11	5	11.471
FB_0808004_L9_PA	fp05_11	5	11.471
FB_0099995_L11_PA	fp05_11	5	11.471
FB_0809228_L9_PA	fp05_11	5	11.471
FB_0825631_L10_31_1	fp05_11	5	11.471
FB_0825618_L10_31_1	fp05_11	5	11.471

FB_0627067_L5_PA	fp05_11	5	11.471
FB_0822451_L9_PA	fp05_11	5	11.471
FB_0915235_L15_76_1	fp05_11	5	11.471
FB_0915240_L15_76_1	fp05_11	5	11.471
FB_0627057_L5_PA	fp05_11	5	11.471
FB_0627068_L5_PA	fp05_11	5	11.471
FB_0626637_L5_PA	fp05_11	5	11.471
FB_0643779_L5_PA	fp05_11	5	11.471
FB_0643396_L5_PA	fp05_12	5	12.384
FB_0643438_L5_PA	fp05_12	5	12.384
FB_0643410_L5_PA	fp05_12	5	12.384
FB_0807175_L9_PA	fp05_12	5	12.384
FB_0641695_L5_PA	fp05_12	5	12.384
FB_0642155_L5_PA	fp05_12	5	12.384
FB_0826691_L10_33_1	fp05_12	5	12.384
RB_34488739_L5_PA	fp05_12	5	12.384
FB_0643419_L5_PA	fp05_12	5	12.384
FB_0643392_L5_PA	fp05_12	5	12.384
FB_0807144_L9_PA	fp05_12	5	12.384
FB_0099976_L11_PA	fp05_12	5	12.384
FB_0642835_L5_PA	fp05_12	5	12.384
FB_1001565_L5_56_1	fp05_12	5	12.384
FB_0642172_L5_PA	fp05_12	5	12.384
FB_1001581_L5_56_1	fp05_12	5	12.384
FB_0825632_L10_31_1	fp05_13	5	13.715
FB_0825617_L10_31_1	fp05_13	5	13.715
RB_34451670_L5_PA	fp05_13	5	13.715
FB_0642157_L5_PA	fp05_14	5	14.483
FB_1077963_L17_27_2	fp05_14	5	14.483
FB_1001561_L5_56_1	fp05_14	5	14.483
FB_0807170_L9_PA	fp05_14	5	14.483
FB_0807155_L9_PA	fp05_14	5	14.483
FB_0639909_L5_PA	fp05_14	5	14.483
RB_33278360_L5_PA	fp05_14	5	14.483
FB_0531228_L3_PA	fp05_14	5	14.483
FB_1117227_L8_84_3	fp05_15	5	15.744
FB_0640526_L5_PA	fp05_15	5	15.744
FB_0088908_L11_PA	fp05_16	5	16.749
FB_0703878_L7_PA	fp05_16	5	16.749
FB_0088142_L11_PA	fp05_16	5	16.749
FB_0531224_L3_PA	fp05_16	5	16.749
FB_0013300_L10_PA	fp05_16	5	16.749
FB_0088455_L11_PA	fp05_16	5	16.749
FB_0088555_L11_PA	fp05_16	5	16.749
FB_0703886_L7_PA	fp05_16	5	16.749
FB_0088149_L11_PA	fp05_16	5	16.749

FB_0703903_L7_PA	fp05_16	5	16.749
RB_33260850_L5_PA	fp05_16	5	16.749
FB_0640957_L5_PA	fp05_16	5	16.749
FB_0640953_L5_PA	fp05_16	5	16.749
FB_0088910_L11_PA	fp05_17	5	17.782
FB_0088917_L11_PA	fp05_17	5	17.782
FB_0640949_L5_PA	fp05_17	5	17.782
RB_34453757_L5_PA	fp05_17	5	17.782
RB_34448952_L5_PA	fp05_17	5	17.782
FB_0642163_L5_PA	fp05_17	5	17.782
FB_0643775_L5_PA	fp05_17	5	17.782
FB_0641701_L5_PA	fp05_17	5	17.782
RB_33263993_L5_PA	fp05_17	5	17.782
FB_0533665_L3_PA	fp05_18	5	18.423
FB_0533663_L3_PA	fp05_18	5	18.423
FB_0533675_L3_PA	fp05_18	5	18.423
FB_0533669_L3_PA	fp05_18	5	18.423
FB_0088543_L11_PA	fp05_18	5	18.423
FB_0640955_L5_PA	fp05_18	5	18.423
FB_0640960_L5_PA	fp05_18	5	18.423
FB_0640500_L5_PA	fp05_19	5	19.659
FB_0703921_L7_PA	fp05_19	5	19.659
FB_0222648_L14_PA	fp05_19	5	19.659
FB_0088460_L11_PA	fp05_19	5	19.659
FB_0222660_L14_PA	fp05_19	5	19.659
FB_0703890_L7_PA	fp05_19	5	19.659
FB_0533684_L3_PA	fp05_19	5	19.659
FB_1054172_L10_64_2	fp05_19	5	19.659
FB_0013292_L10_PA	fp05_19	5	19.659
FB_0642830_L5_PA	fp05_20	5	20.533
FB_0642167_L5_PA	fp05_20	5	20.533
FB_1001596_L5_56_1	fp05_20	5	20.533
FB_1099146_L7_58_2	fp05_20	5	20.533
FB_0531233_L3_PA	fp05_20	5	20.533
FB_0088481_L11_PA	fp05_20	5	20.533
FB_0222659_L14_PA	fp05_20	5	20.533
FB_0088483_L11_PA	fp05_20	5	20.533
FB_0640491_L5_PA	fp05_21	5	21.667
FB_0638568_L5_PA	fp05_21	5	21.667
FB_0640495_L5_PA	fp05_21	5	21.667
FB_0640490_L5_PA	fp05_21	5	21.667
FB_1001173_L5_55_1	fp05_21	5	21.667
FB_0639913_L5_PA	fp05_21	5	21.667
FB_1001140_L5_55_1	fp05_21	5	21.667
FB_0639923_L5_PA	fp05_21	5	21.667
RB_32907222_L5_PA	fp05_21	5	21.667

FB_0639921_L5_PA	fp05_21	5	21.667
FB_1001139_L5_55_1	fp05_21	5	21.667
FB_0639914_L5_PA	fp05_21	5	21.667
FB_0639906_L5_PA	fp05_21	5	21.667
FB_1000694_L5_53_1	fp05_22	5	22.59
FB_0640497_L5_PA	fp05_22	5	22.59
GD_02729_L5_54_1	fp05_22	5	22.59
FB_1000746_L5_54_1	fp05_22	5	22.59
FB_1000748_L5_54_1	fp05_22	5	22.59
FB_1000749_L5_54_1	fp05_22	5	22.59
FB_0088562_L11_PA	fp05_22	5	22.59
FB_1000670_L5_53_1	fp05_23	5	23.739
FB_0082102_L11_PA	fp05_23	5	23.739
FB_0082098_L11_PA	fp05_23	5	23.739
FB_1000654_L5_53_1	fp05_23	5	23.739
FB_0637158_L5_PA	fp05_23	5	23.739
RB_31321013_L5_53_1	fp05_23	5	23.739
FB_0956866_L2_27_1	fp05_23	5	23.739
FB_0434578_L1_PA	fp05_23	5	23.739
FB_1000652_L5_53_1	fp05_23	5	23.739
FB_0637491_L5_PA	fp05_23	5	23.739
FB_0637484_L5_PA	fp05_23	5	23.739
RB_31372866_L5_PA	fp05_23	5	23.739
GD_01601_L5_PA	fp05_23	5	23.739
RB_31358728_L5_PA	fp05_23	5	23.739
RB_31365126_L5_53_1	fp05_23	5	23.739
FB_0637510_L5_PA	fp05_23	5	23.739
RB_31342659_L5_PA	fp05_23	5	23.739
FB_0638033_L5_PA	fp05_23	5	23.739
RB_31334004_L5_PA	fp05_23	5	23.739
FB_0637656_L5_PA	fp05_23	5	23.739
FB_0082072_L11_PA	fp05_23	5	23.739
FB_1000662_L5_53_1	fp05_23	5	23.739
FB_1000671_L5_53_1	fp05_23	5	23.739
FB_0082081_L11_PA	fp05_23	5	23.739
FB_0639637_L5_PA	fp05_23	5	23.739
FB_1005388_L6_34_1	fp05_23	5	23.739
FB_0434582_L1_PA	fp05_23	5	23.739
FB_1005398_L6_34_1	fp05_23	5	23.739
FB_0661874_L6_PA	fp05_23	5	23.739
FB_0639593_L5_PA	fp05_23	5	23.739
FB_0956873_L2_27_1	fp05_23	5	23.739
FB_0639636_L5_PA	fp05_23	5	23.739
GD_01539_L5_PA	fp05_23	5	23.739
FB_1000658_L5_53_1	fp05_23	5	23.739
FB_0956872_L2_27_1	fp05_23	5	23.739

FB_0082066_L11_PA	fp05_23	5	23.739
FB_0403676_L17_PA	fp05_23	5	23.739
FB_1000653_L5_53_1	fp05_23	5	23.739
FB_0639925_L5_PA	fp05_23	5	23.739
FB_1001146_L5_55_1	fp05_23	5	23.739
FB_0640524_L5_PA	fp05_23	5	23.739
FB_0637482_L5_PA	fp05_24	5	24.532
FB_0638567_L5_PA	fp05_24	5	24.532
FB_0637163_L5_PA	fp05_24	5	24.532
FB_0636422_L5_PA	fp05_24	5	24.532
FB_1096061_L5_77_2	fp05_24	5	24.532
FB_0637483_L5_PA	fp05_24	5	24.532
FB_0636402_L5_PA	fp05_24	5	24.532
FB_0636421_L5_PA	fp05_24	5	24.532
FB_0637165_L5_PA	fp05_24	5	24.532
FB_0637645_L5_PA	fp05_24	5	24.532
FB_0637162_L5_PA	fp05_24	5	24.532
FB_1096064_L5_77_2	fp05_24	5	24.532
FB_0637661_L5_PA	fp05_24	5	24.532
FB_0637513_L5_PA	fp05_24	5	24.532
FB_0637155_L5_PA	fp05_24	5	24.532
FB_0636409_L5_PA	fp05_24	5	24.532
FB_0637626_L5_PA	fp05_24	5	24.532
FB_0637659_L5_PA	fp05_24	5	24.532
FB_0636906_L5_PA	fp05_24	5	24.532
FB_0637159_L5_PA	fp05_24	5	24.532
RB_30331070_L5_PA	fp05_24	5	24.532
RB_30366787_L5_PA	fp05_24	5	24.532
RB_30355439_L5_PA	fp05_24	5	24.532
FB_1096051_L5_77_2	fp05_24	5	24.532
FB_0637156_L5_PA	fp05_24	5	24.532
FB_1000677_L5_53_1	fp05_25	5	25.466
FB_0636908_L5_PA	fp05_25	5	25.466
FB_0636923_L5_PA	fp05_25	5	25.466
FB_0636913_L5_PA	fp05_25	5	25.466
FB_0636924_L5_PA	fp05_25	5	25.466
FB_0999645_L5_50_1	fp05_25	5	25.466
FB_0056441_L11_PA	fp05_25	5	25.466
FB_0056445_L11_PA	fp05_25	5	25.466
FB_0632664_L5_PA	fp05_25	5	25.466
FB_0999650_L5_50_1	fp05_25	5	25.466
FB_0999654_L5_50_1	fp05_25	5	25.466
FB_0636441_L5_PA	fp05_25	5	25.466
FB_0633630_L5_PA	fp05_26	5	26.744
RB_27698167_L5_PA	fp05_26	5	26.744
FB_0633770_L5_PA	fp05_26	5	26.744

FB_0079036_L11_PA	fp05_26	5	26.744
FB_0079063_L11_PA	fp05_26	5	26.744
FB_0633777_L5_PA	fp05_27	5	27.411
RB_27907615_L5_PA	fp05_27	5	27.411
RB_27828226_L5_PA	fp05_27	5	27.411
RB_27703600_L5_PA	fp05_27	5	27.411
RB_27841836_L5_PA	fp05_27	5	27.411
FB_0632685_L5_PA	fp05_27	5	27.411
RB_26990664_L5_PA	fp05_27	5	27.411
RB_26970454_L5_PA	fp05_27	5	27.411
RB_26979906_L5_PA	fp05_27	5	27.411
RB_27675794_L5_PA	fp05_27	5	27.411
RB_27680788_L5_PA	fp05_27	5	27.411
FB_0633112_L5_PA	fp05_27	5	27.411
FB_0632684_L5_PA	fp05_27	5	27.411
FB_0631238_L5_PA	fp05_27	5	27.411
RB_27700882_L5_PA	fp05_27	5	27.411
RB_14945861_L5_PA	fp05_27	5	27.411
FB_0631240_L5_PA	fp05_27	5	27.411
FB_0303530_L15_PA	fp05_27	5	27.411
FB_0633634_L5_PA	fp05_28	5	28.588
FB_0633631_L5_PA	fp05_28	5	28.588
FB_0633642_L5_PA	fp05_28	5	28.588
FB_0631225_L5_PA	fp05_28	5	28.588
FB_0631215_L5_PA	fp05_28	5	28.588
RB_26877863_L5_48_1	fp05_28	5	28.588
FB_0633110_L5_PA	fp05_28	5	28.588
FB_0190383_L13_PA	fp05_28	5	28.588
FB_0632676_L5_PA	fp05_28	5	28.588
FB_0631226_L5_PA	fp05_28	5	28.588
FB_1063419_L13_50_2	fp05_28	5	28.588
RB_27677928_L5_PA	fp05_29	5	29.363
FB_0737955_L8_PA	fp05_29	5	29.363
FB_0737365_L8_PA	fp05_33	5	33.407
FB_1019149_L7_47_1	fp05_33	5	33.407
FB_1019162_L7_47_1	fp05_33	5	33.407
FB_0998718_L5_46_1	fp05_33	5	33.407
FB_0737357_L8_PA	fp05_33	5	33.407
FB_0630565_L5_PA	fp05_33	5	33.407
FB_0630559_L5_PA	fp05_33	5	33.407
FB_1019144_L7_47_1	fp05_33	5	33.407
FB_0737356_L8_PA	fp05_33	5	33.407
FB_0737977_L8_PA	fp05_33	5	33.407
FB_1019147_L7_47_1	fp05_33	5	33.407
FB_0713645_L7_PA	fp05_33	5	33.407
FB_0737379_L8_PA	fp05_33	5	33.407

FB_0737376_L8_PA	fp05_33	5	33.407
FB_0737348_L8_PA	fp05_33	5	33.407
FB_0630573_L5_PA	fp05_33	5	33.407
FB_0998712_L5_46_1	fp05_33	5	33.407
FB_0630567_L5_PA	fp05_33	5	33.407
FB_0737349_L8_PA	fp05_33	5	33.407
FB_0736817_L8_PA	fp05_34	5	34.345
FB_0630574_L5_PA	fp05_34	5	34.345
RB_25133972_L5_PA	fp05_34	5	34.345
FB_0736819_L8_PA	fp05_34	5	34.345
FB_0019758_L10_PA	fp05_34	5	34.345
RB_25120947_L5_46_1	fp05_34	5	34.345
FB_0736801_L8_PA	fp05_34	5	34.345
FB_0736812_L8_PA	fp05_35	5	35.515
FB_0019780_L10_PA	fp05_35	5	35.515
FB_0020204_L10_PA	fp05_35	5	35.515
FB_0019784_L10_PA	fp05_36	5	36.334
FB_0021119_L10_PA	fp05_36	5	36.334
FB_0021100_L10_PA	fp05_36	5	36.334
FB_0351907_L16_PA	fp05_36	5	36.334
FB_0739090_L8_PA	fp05_36	5	36.334
FB_0351898_L16_PA	fp05_36	5	36.334
FB_0021102_L10_PA	fp05_36	5	36.334
FB_0021101_L10_PA	fp05_36	5	36.334
GD_01304_L5_PA	fp05_36	5	36.334
FB_0020199_L10_PA	fp05_36	5	36.334
FB_0630019_L5_PA	fp05_36	5	36.334
FB_0020198_L10_PA	fp05_36	5	36.334
FB_0019767_L10_PA	fp05_36	5	36.334
FB_0020215_L10_PA	fp05_36	5	36.334
FB_0630020_L5_PA	fp05_36	5	36.334
FB_0630017_L5_PA	fp05_36	5	36.334
FB_0630028_L5_PA	fp05_36	5	36.334
FB_0630025_L5_PA	fp05_36	5	36.334
FB_0019762_L10_PA	fp05_37	5	37.393
FB_0739091_L8_PA	fp05_37	5	37.393
FB_0739076_L8_PA	fp05_37	5	37.393
FB_1027577_L8_37_1	fp05_37	5	37.393
FB_0739085_L8_PA	fp05_37	5	37.393
FB_0621437_L5_PA	fp05_37	5	37.393
RB_19498219_L5_PA	fp05_37	5	37.393
RB_19493983_L5_PA	fp05_37	5	37.393
FB_0622685_L5_PA	fp05_37	5	37.393
RB_19699729_L5_PA	fp05_37	5	37.393
RB_19678308_L5_PA	fp05_37	5	37.393
FB_0572921_L4_PA	fp05_37	5	37.393

FB_0021109_L10_PA	fp05_37	5	37.393
FB_0572914_L4_PA	fp05_39	5	39.585
FB_0738685_L8_PA	fp05_39	5	39.585
FB_0572917_L4_PA	fp05_39	5	39.585
FB_1027576_L8_37_1	fp05_39	5	39.585
FB_0624325_L5_PA	fp05_39	5	39.585
FB_0624345_L5_PA	fp05_39	5	39.585
FB_0624320_L5_PA	fp05_39	5	39.585
RB_21402981_L5_PA	fp05_39	5	39.585
FB_0624326_L5_PA	fp05_39	5	39.585
GD_01612_L5_PA	fp05_39	5	39.585
FB_0679077_L6_PA	fp05_40	5	40.517
FB_0624312_L5_PA	fp05_40	5	40.517
FB_0679070_L6_PA	fp05_41	5	41.65
FB_0572913_L4_PA	fp05_41	5	41.65
FB_0679097_L6_PA	fp05_41	5	41.65
FB_0679046_L6_PA	fp05_41	5	41.65
FB_0679062_L6_PA	fp05_41	5	41.65
RB_21377879_L5_PA	fp05_41	5	41.65
FB_0679063_L6_PA	fp05_41	5	41.65
FB_0679078_L6_PA	fp05_41	5	41.65
FB_0624339_L5_PA	fp05_41	5	41.65
FB_0679092_L6_PA	fp05_41	5	41.65
FB_0572927_L4_PA	fp05_41	5	41.65
FB_0679084_L6_PA	fp05_41	5	41.65
RB_21367438_L5_PA	fp05_41	5	41.65
GD_00867_L5_PA	fp05_44	5	44.609
RB_20287262_L5_PA	fp05_45	5	45.246
RB_20289532_L5_PA	fp05_45	5	45.246
FB_0623228_L5_PA	fp05_45	5	45.246
FB_0623232_L5_PA	fp05_45	5	45.246
FB_0623235_L5_PA	fp05_45	5	45.246
FB_0572922_L4_PA	fp05_45	5	45.246
FB_0621430_L5_PA	fp05_46	5	46.364
FB_0995817_L5_42_1	fp05_46	5	46.364
FB_0622204_L5_PA	fp05_46	5	46.364
FB_0621455_L5_PA	fp05_46	5	46.364
FB_0622217_L5_PA	fp05_46	5	46.364
FB_0621431_L5_PA	fp05_46	5	46.364
FB_0622222_L5_PA	fp05_46	5	46.364
FB_0622201_L5_PA	fp05_46	5	46.364
GD_01478_L5_PA	fp05_46	5	46.364
FB_0995712_L5_42_1	fp05_46	5	46.364
FB_0621436_L5_PA	fp05_46	5	46.364
FB_0995708_L5_42_1	fp05_46	5	46.364
FB_0622207_L5_PA	fp05_46	5	46.364

FB_0621432_L5_PA	fp05_46	5	46.364
FB_0620376_L5_PA	fp05_46	5	46.364
FB_0620347_L5_PA	fp05_46	5	46.364
FB_0620374_L5_PA	fp05_46	5	46.364
FB_0621448_L5_PA	fp05_47	5	47.512
FB_0621446_L5_PA	fp05_47	5	47.512
RB_18559967_L5_PA	fp05_47	5	47.512
FB_0995242_L5_42_1	fp05_47	5	47.512
GD_00323_L5_PA	fp05_47	5	47.512
FB_0995249_L5_42_1	fp05_47	5	47.512
FB_0619585_L5_PA	fp05_47	5	47.512
FB_0619602_L5_PA	fp05_47	5	47.512
FB_0617605_L5_PA	fp05_48	5	48.449
RB_18232556_L5_PA	fp05_48	5	48.449
FB_0618490_L5_PA	fp05_48	5	48.449
FB_0618990_L5_PA	fp05_48	5	48.449
RB_18586613_L5_PA	fp05_48	5	48.449
RB_18232690_L5_PA	fp05_48	5	48.449
RB_18232808_L5_PA	fp05_48	5	48.449
FB_0618472_L5_PA	fp05_48	5	48.449
FB_0619012_L5_PA	fp05_48	5	48.449
RB_18043587_L5_PA	fp05_48	5	48.449
FB_0618478_L5_PA	fp05_48	5	48.449
RB_18053412_L5_PA	fp05_48	5	48.449
FB_0618997_L5_PA	fp05_48	5	48.449
FB_0619609_L5_PA	fp05_48	5	48.449
FB_0619029_L5_PA	fp05_48	5	48.449
FB_0619002_L5_PA	fp05_48	5	48.449
FB_0619601_L5_PA	fp05_48	5	48.449
RB_18579919_L5_PA	fp05_48	5	48.449
FB_0618489_L5_PA	fp05_48	5	48.449
FB_0323088_L15_PA	fp05_48	5	48.449
FB_0323092_L15_PA	fp05_48	5	48.449
RB_18757493_L5_PA	fp05_49	5	49.527
RB_24525168_L5_45_1	fp05_49	5	49.527
RB_15491385_L5_40_1	fp05_49	5	49.527
RB_24417621_L5_PA	fp05_49	5	49.527
FB_0462931_L2_PA	fp05_49	5	49.527
FB_0323102_L15_PA	fp05_49	5	49.527
FB_0323101_L15_PA	fp05_49	5	49.527
RB_24528761_L5_45_1	fp05_50	5	50.649
FB_1115903_L5_87_3	fp05_50	5	50.649
RB_17565719_L5_PA	fp05_50	5	50.649
FB_0875724_L13_35_1	fp05_51	5	51.478
FB_1095716_L5_73_2	fp05_51	5	51.478
GD_00978_L5_PA	fp05_51	5	51.478

GD_01616_L5_40_1	fp05_51	5	51.478
RB_13662840_L5_PA	fp05_51	5	51.478
FB_0613606_L5_PA	fp05_51	5	51.478
RB_13685952_L5_PA	fp05_51	5	51.478
FB_0617608_L5_PA	fp05_51	5	51.478
FB_1064279_L13_58_2	fp05_51	5	51.478
FB_0993479_L5_40_1	fp05_51	5	51.478
FB_0993442_L5_40_1	fp05_51	5	51.478
FB_0993439_L5_40_1	fp05_51	5	51.478
FB_0462943_L2_PA	fp05_51	5	51.478
RB_24403609_L5_PA	fp05_51	5	51.478
FB_0462920_L2_PA	fp05_51	5	51.478
FB_0619019_L5_PA	fp05_51	5	51.478
FB_0612592_L5_PA	fp05_52	5	52.634
RB_11972840_L5_PA	fp05_52	5	52.634
FB_0992516_L5_38_1	fp05_52	5	52.634
RB_11985564_L5_38_1	fp05_52	5	52.634
FB_0611411_L5_PA	fp05_52	5	52.634
FB_0611428_L5_PA	fp05_52	5	52.634
RB_13680239_L5_PA	fp05_52	5	52.634
FB_0611412_L5_PA	fp05_52	5	52.634
FB_0611418_L5_PA	fp05_52	5	52.634
RB_11970050_L5_38_1	fp05_52	5	52.634
FB_0611405_L5_PA	fp05_52	5	52.634
FB_0611422_L5_PA	fp05_52	5	52.634
FB_0611434_L5_PA	fp05_52	5	52.634
FB_0611417_L5_PA	fp05_52	5	52.634
FB_0617614_L5_PA	fp05_52	5	52.634
RB_13669196_L5_PA	fp05_53	5	53.354
FB_0613612_L5_PA	fp05_53	5	53.354
FB_1095726_L5_73_2	fp05_53	5	53.354
FB_0992519_L5_38_1	fp05_53	5	53.354
FB_0612616_L5_PA	fp05_53	5	53.354
FB_0992523_L5_38_1	fp05_53	5	53.354
FB_1094228_L5_66_2	fp05_53	5	53.354
FB_1095720_L5_73_2	fp05_53	5	53.354
GD_01319_L5_38_1	fp05_53	5	53.354
FB_0612618_L5_PA	fp05_53	5	53.354
FB_0629339_L5_PA	fp05_53	5	53.354
FB_0629328_L5_PA	fp05_53	5	53.354
FB_0462933_L2_PA	fp05_53	5	53.354
FB_0993451_L5_40_1	fp05_53	5	53.354
FB_1094448_L5_69_2	fp05_53	5	53.354
FB_0616707_L5_PA	fp05_53	5	53.354
FB_0616715_L5_PA	fp05_54	5	54.711
FB_1094449_L5_69_2	fp05_54	5	54.711

FB_0616698_L5_PA	fp05_54	5	54.711
FB_0616690_L5_PA	fp05_54	5	54.711
GD_02809_L5_PA	fp05_54	5	54.711
FB_0991822_L5_38_1	fp05_57	5	57.553
FB_1094221_L5_66_2	fp05_57	5	57.553
FB_0610493_L5_PA	fp05_57	5	57.553
FB_0610499_L5_PA	fp05_57	5	57.553
RB_24406956_L5_PA	fp05_57	5	57.553
FB_0612624_L5_PA	fp05_57	5	57.553
RB_11479429_L5_PA	fp05_57	5	57.553
RB_11403959_L5_PA	fp05_57	5	57.553
FB_0462934_L2_PA	fp05_57	5	57.553
FB_0610487_L5_PA	fp05_57	5	57.553
FB_0462945_L2_PA	fp05_57	5	57.553
FB_0629345_L5_PA	fp05_57	5	57.553
RB_11445563_L5_PA	fp05_57	5	57.553
RB_12016362_L5_PA	fp05_57	5	57.553
FB_0608388_L5_PA	fp05_58	5	58.994
FB_0608364_L5_PA	fp05_58	5	58.994
FB_0991819_L5_38_1	fp05_59	5	59.428
FB_0608982_L5_PA	fp05_59	5	59.428
FB_0991828_L5_38_1	fp05_59	5	59.428
FB_0991808_L5_38_1	fp05_59	5	59.428
FB_0991821_L5_38_1	fp05_59	5	59.428
FB_0991829_L5_38_1	fp05_59	5	59.428
FB_0609795_L5_PA	fp05_59	5	59.428
FB_0609815_L5_PA	fp05_59	5	59.428
FB_0991834_L5_38_1	fp05_59	5	59.428
RB_9499250_L5_PA	fp05_60	5	60.585
FB_0608389_L5_PA	fp05_60	5	60.585
GD_01651_L5_PA	fp05_60	5	60.585
RB_10296435_L5_PA	fp05_60	5	60.585
FB_0608382_L5_PA	fp05_60	5	60.585
FB_0608356_L5_PA	fp05_60	5	60.585
RB_10289990_L5_PA	fp05_60	5	60.585
FB_0608373_L5_PA	fp05_60	5	60.585
FB_0608384_L5_PA	fp05_60	5	60.585
FB_0991827_L5_38_1	fp05_61	5	61.364
FB_0991838_L5_38_1	fp05_61	5	61.364
RB_10293726_L5_PA	fp05_61	5	61.364
RB_10281385_L5_PA	fp05_61	5	61.364
FB_0607347_L5_PA	fp05_62	5	62.701
RB_9503447_L5_PA	fp05_62	5	62.701
FB_0607134_L5_PA	fp05_63	5	63.705
FB_0607137_L5_PA	fp05_63	5	63.705
GD_01781_L5_PA	fp05_63	5	63.705

RB_9267886_L5_PA	fp05_63	5	63.705
RB_9507490_L5_PA	fp05_63	5	63.705
GD_01896_L5_PA	fp05_63	5	63.705
FB_0863475_L12_46_1	fp05_63	5	63.705
RB_7087864_L5_PA	fp05_65	5	65.683
FB_0993155_L5_39_1	fp05_65	5	65.683
FB_0606414_L5_PA	fp05_65	5	65.683
GD_01208_L5_PA	fp05_65	5	65.683
FB_0606425_L5_PA	fp05_65	5	65.683
FB_0993142_L5_39_1	fp05_65	5	65.683
FB_0606420_L5_PA	fp05_65	5	65.683
FB_0993176_L5_39_1	fp05_65	5	65.683
GD_01942_L5_PA	fp05_65	5	65.683
FB_0606404_L5_PA	fp05_65	5	65.683
FB_0993175_L5_39_1	fp05_65	5	65.683
FB_0606406_L5_PA	fp05_65	5	65.683
FB_0607136_L5_PA	fp05_65	5	65.683
FB_0607328_L5_PA	fp05_65	5	65.683
RB_14674789_L5_PA	fp05_65	5	65.683
FB_0993158_L5_39_1	fp05_65	5	65.683
FB_0606417_L5_PA	fp05_65	5	65.683
RB_14520956_L5_PA	fp05_65	5	65.683
FB_0993153_L5_39_1	fp05_65	5	65.683
RB_14520985_L5_PA	fp05_65	5	65.683
RB_14412254_L5_PA	fp05_65	5	65.683
RB_14437846_L5_PA	fp05_65	5	65.683
RB_14437669_L5_PA	fp05_65	5	65.683
RB_14520884_L5_PA	fp05_65	5	65.683
RB_14365897_L5_PA	fp05_65	5	65.683
RB_9251763_L5_PA	fp05_65	5	65.683
FB_0863476_L12_46_1	fp05_65	5	65.683
FB_0993145_L5_39_1	fp05_66	5	66.322
FB_0605910_L5_PA	fp05_66	5	66.322
FB_0605921_L5_PA	fp05_66	5	66.322
FB_0605906_L5_PA	fp05_66	5	66.322
FB_0605899_L5_PA	fp05_66	5	66.322
FB_0614211_L5_PA	fp05_66	5	66.322
FB_0614232_L5_PA	fp05_66	5	66.322
RB_8643407_L5_PA	fp05_66	5	66.322
FB_0606412_L5_PA	fp05_66	5	66.322
FB_0993146_L5_39_1	fp05_67	5	67.459
FB_0605932_L5_PA	fp05_67	5	67.459
FB_0604576_L5_PA	fp05_67	5	67.459
FB_0604593_L5_PA	fp05_67	5	67.459
FB_0604619_L5_PA	fp05_67	5	67.459
FB_0604624_L5_PA	fp05_67	5	67.459

FB_0604582_L5_PA	fp05_67	5	67.459
RB_7062450_L5_PA	fp05_67	5	67.459
FB_0604599_L5_PA	fp05_67	5	67.459
RB_8666295_L5_PA	fp05_67	5	67.459
FB_0604611_L5_PA	fp05_67	5	67.459
FB_0603896_L5_PA	fp05_67	5	67.459
GD_00238_L5_PA	fp05_67	5	67.459
RB_14376292_L5_PA	fp05_68	5	68.62
FB_0990635_L5_34_1	fp05_68	5	68.62
FB_0603392_L5_PA	fp05_68	5	68.62
RB_8345524_L5_PA	fp05_68	5	68.62
FB_0602985_L5_PA	fp05_69	5	69.571
RB_6192018_L5_PA	fp05_69	5	69.571
FB_0990254_L5_34_1	fp05_69	5	69.571
FB_0600229_L5_PA	fp05_69	5	69.571
RB_6165596_L5_PA	fp05_69	5	69.571
RB_8173286_L5_PA	fp05_69	5	69.571
FB_0603394_L5_PA	fp05_69	5	69.571
FB_0603384_L5_PA	fp05_69	5	69.571
FB_0603389_L5_PA	fp05_69	5	69.571
FB_0989862_L5_34_1	fp05_69	5	69.571
RB_14354679_L5_PA	fp05_69	5	69.571
FB_0993156_L5_39_1	fp05_69	5	69.571
FB_0606428_L5_PA	fp05_69	5	69.571
RB_8361026_L5_PA	fp05_69	5	69.571
RB_8682553_L5_PA	fp05_69	5	69.571
FB_0602993_L5_PA	fp05_70	5	70.893
RB_6801757_L5_PA	fp05_70	5	70.893
FB_0990257_L5_34_1	fp05_70	5	70.893
FB_0602364_L5_PA	fp05_70	5	70.893
FB_0602968_L5_PA	fp05_70	5	70.893
FB_0990024_L5_34_1	fp05_70	5	70.893
FB_0989856_L5_34_1	fp05_70	5	70.893
FB_0602357_L5_PA	fp05_70	5	70.893
FB_0601836_L5_PA	fp05_70	5	70.893
FB_0601652_L5_PA	fp05_70	5	70.893
RB_5713160_L5_PA	fp05_70	5	70.893
FB_0990258_L5_34_1	fp05_70	5	70.893
FB_0602955_L5_PA	fp05_70	5	70.893
FB_0601839_L5_PA	fp05_70	5	70.893
FB_0990256_L5_34_1	fp05_70	5	70.893
FB_0601646_L5_PA	fp05_70	5	70.893
GD_01697_L5_PA	fp05_70	5	70.893
FB_0990008_L5_34_1	fp05_70	5	70.893
FB_0601642_L5_PA	fp05_70	5	70.893
FB_0989861_L5_34_1	fp05_70	5	70.893

FB_0990278_L5_34_1	fp05_70	5	70.893
FB_0601672_L5_PA	fp05_70	5	70.893
FB_0990003_L5_34_1	fp05_70	5	70.893
FB_0600943_L5_PA	fp05_70	5	70.893
FB_0601840_L5_PA	fp05_70	5	70.893
FB_0601654_L5_PA	fp05_70	5	70.893
FB_0601833_L5_PA	fp05_70	5	70.893
FB_0601837_L5_PA	fp05_70	5	70.893
FB_0601661_L5_PA	fp05_70	5	70.893
FB_0990005_L5_34_1	fp05_70	5	70.893
RB_6230318_L5_PA	fp05_70	5	70.893
FB_0990020_L5_34_1	fp05_70	5	70.893
FB_0601842_L5_PA	fp05_70	5	70.893
FB_0600917_L5_PA	fp05_70	5	70.893
FB_0600934_L5_PA	fp05_70	5	70.893
FB_0990011_L5_34_1	fp05_70	5	70.893
RB_5472549_L5_PA	fp05_70	5	70.893
FB_0600940_L5_PA	fp05_70	5	70.893
FB_0601671_L5_PA	fp05_70	5	70.893
FB_0600920_L5_PA	fp05_70	5	70.893
FB_0603895_L5_PA	fp05_71	5	71.591
FB_0603892_L5_PA	fp05_71	5	71.591
RB_7110030_L5_PA	fp05_71	5	71.591
FB_0600226_L5_PA	fp05_71	5	71.591
FB_0600222_L5_PA	fp05_71	5	71.591
FB_0989855_L5_34_1	fp05_71	5	71.591
RB_6078855_L5_PA	fp05_71	5	71.591
RB_5391928_L5_PA	fp05_71	5	71.591
FB_0600256_L5_PA	fp05_71	5	71.591
RB_5394062_L5_PA	fp05_71	5	71.591
RB_5246563_L5_PA	fp05_71	5	71.591
FB_0600252_L5_PA	fp05_71	5	71.591
FB_0601659_L5_PA	fp05_71	5	71.591
FB_0601829_L5_PA	fp05_71	5	71.591
FB_0601660_L5_PA	fp05_72	5	72.581
FB_0599604_L5_PA	fp05_72	5	72.581
FB_0599606_L5_PA	fp05_72	5	72.581
FB_0599610_L5_PA	fp05_72	5	72.581
RB_4303901_L5_PA	fp05_73	5	73.932
RB_4277208_L5_34_1	fp05_73	5	73.932
RB_4312438_L5_PA	fp05_73	5	73.932
RB_4171437_L5_PA	fp05_74	5	74.516
FB_0599007_L5_PA	fp05_74	5	74.516
FB_0989306_L5_34_1	fp05_74	5	74.516
RB_4273093_L5_34_1	fp05_74	5	74.516
RB_4173950_L5_PA	fp05_74	5	74.516

FB_0598996_L5_PA	fp05_74	5	74.516
FB_0599586_L5_PA	fp05_74	5	74.516
GD_01566_L5_PA	fp05_74	5	74.516
RB_4178848_L5_PA	fp05_74	5	74.516
RB_2446003_L5_PA	fp05_74	5	74.516
RB_4345700_L5_PA	fp05_74	5	74.516
RB_2426406_L5_PA	fp05_74	5	74.516
FB_0599005_L5_PA	fp05_74	5	74.516
FB_0989296_L5_34_1	fp05_74	5	74.516
FB_0599006_L5_PA	fp05_74	5	74.516
FB_0598994_L5_PA	fp05_74	5	74.516
RB_4192787_L5_34_1	fp05_74	5	74.516
FB_0599012_L5_PA	fp05_74	5	74.516
RB_4183536_L5_PA	fp05_74	5	74.516
FB_0987787_L4_25_1	fp05_75	5	75.181
GD_00179_L5_PA	fp05_75	5	75.181
FB_0597161_L5_PA	fp05_75	5	75.181
FB_0597166_L5_PA	fp05_75	5	75.181
RB_2404175_L5_PA	fp05_75	5	75.181
FB_0597459_L5_PA	fp05_75	5	75.181
FB_0597457_L5_PA	fp05_75	5	75.181
RB_2406346_L5_PA	fp05_75	5	75.181
FB_0597173_L5_PA	fp05_75	5	75.181
FB_0597458_L5_PA	fp05_75	5	75.181
RB_1216653_L5_PA	fp05_76	5	76.695
FB_0596225_L5_PA	fp05_76	5	76.695
FB_1115769_L5_84_3	fp05_76	5	76.695
RB_1270220_L5_PA	fp05_76	5	76.695
RB_1213508_L5_PA	fp05_76	5	76.695
FB_0596223_L5_PA	fp05_76	5	76.695
RB_1227390_L5_PA	fp05_76	5	76.695
FB_1115770_L5_84_3	fp05_76	5	76.695
RB_2399839_L5_PA	fp05_77	5	77.408
FB_0595281_L5_PA	fp05_77	5	77.408
RB_602526_L5_PA	fp05_77	5	77.408
FB_0987800_L4_25_1	fp05_77	5	77.408
RB_781089_L5_PA	fp05_77	5	77.408
FB_0595277_L5_PA	fp05_77	5	77.408
RB_477972_L5_PA	fp05_77	5	77.408
RB_478063_L5_PA	fp05_77	5	77.408
RB_555246_L5_PA	fp05_77	5	77.408
RB_788558_L5_PA	fp05_77	5	77.408
RB_477752_L5_PA	fp05_77	5	77.408
RB_551887_L5_PA	fp05_77	5	77.408
RB_585755_L5_PA	fp05_77	5	77.408
RB_575860_L5_PA	fp05_78	5	78.372

RB_984342_L5_PA	fp05_78	5	78.372
FB_0595454_L5_PA	fp05_78	5	78.372
GD_00737_L5_PA	fp05_78	5	78.372
GD_01694_L5_PA	fp05_78	5	78.372
FB_0595731_L5_PA	fp05_78	5	78.372
FB_0595455_L5_PA	fp05_78	5	78.372
FB_1115765_L5_84_3	fp05_78	5	78.372
FB_0595768_L5_PA	fp05_78	5	78.372
FB_0595736_L5_PA	fp05_78	5	78.372
FB_0595451_L5_PA	fp05_78	5	78.372
GD_01956_L5_31_1	fp05_78	5	78.372
FB_0595765_L5_PA	fp05_78	5	78.372
FB_0596228_L5_PA	fp05_78	5	78.372
FB_0596233_L5_PA	fp05_78	5	78.372
FB_0596226_L5_PA	fp05_78	5	78.372
FB_0595766_L5_PA	fp05_78	5	78.372
FB_0839155_L10_50_1	fp05_78	5	78.372
FB_0596217_L5_PA	fp05_78	5	78.372
RB_978082_L5_PA	fp05_78	5	78.372
RB_557375_L5_PA	fp05_78	5	78.372
RB_957743_L5_PA	fp05_78	5	78.372
RB_981417_L5_PA	fp05_78	5	78.372
RB_592309_L5_PA	fp05_78	5	78.372
FB_0595468_L5_PA	fp05_78	5	78.372
RB_606585_L5_PA	fp05_78	5	78.372
FB_0053586_L10_PA	fp05_78	5	78.372
RB_993478_L5_PA	fp05_78	5	78.372
RB_1218831_L5_PA	fp05_78	5	78.372
FB_1115766_L5_84_3	fp05_78	5	78.372
FB_0596221_L5_PA	fp05_78	5	78.372
FB_0595457_L5_PA	fp05_78	5	78.372
FB_0595289_L5_PA	fp05_79	5	79.926
GD_02457_L5_PA	fp05_80	5	80.022
FB_0865633_L12_46_1	fp06_00	6	0.126
FB_0646178_L6_PA	fp06_00	6	0.126
FB_0646192_L6_PA	fp06_00	6	0.126
FB_0646175_L6_PA	fp06_00	6	0.126
FB_0645784_L6_PA	fp06_00	6	0.126
RB_361822_L6_PA	fp06_00	6	0.126
FB_0645785_L6_PA	fp06_00	6	0.126
FB_0646195_L6_PA	fp06_00	6	0.126
FB_0645793_L6_PA	fp06_00	6	0.126
FB_0865630_L12_46_1	fp06_00	6	0.126
FB_0646177_L6_PA	fp06_00	6	0.126
FB_0646173_L6_PA	fp06_00	6	0.126
GD_00347_L6_PA	fp06_00	6	0.126

RB_392356_L6_PA	fp06_00	6	0.126
FB_0645797_L6_PA	fp06_00	6	0.126
RB_372637_L6_25_1	fp06_00	6	0.126
FB_0830026_L10_39_1	fp06_00	6	0.126
FB_0646172_L6_PA	fp06_01	6	1.229
FB_0647869_L6_PA	fp06_01	6	1.229
FB_0302312_L15_PA	fp06_01	6	1.229
FB_0647371_L6_PA	fp06_01	6	1.229
FB_0647374_L6_PA	fp06_01	6	1.229
FB_0647255_L6_PA	fp06_01	6	1.229
GD_02169_L6_PA	fp06_01	6	1.229
GD_01087_L6_PA	fp06_01	6	1.229
FB_1096435_L6_46_2	fp06_01	6	1.229
FB_0647372_L6_PA	fp06_01	6	1.229
FB_0302311_L15_PA	fp06_01	6	1.229
FB_0647249_L6_PA	fp06_01	6	1.229
FB_1096463_L6_47_2	fp06_01	6	1.229
FB_1096444_L6_46_2	fp06_01	6	1.229
RB_1307443_L6_PA	fp06_01	6	1.229
FB_0647379_L6_PA	fp06_01	6	1.229
FB_0647892_L6_PA	fp06_03	6	3.473
FB_0648454_L6_PA	fp06_04	6	4.91
RB_2553338_L6_PA	fp06_04	6	4.91
FB_0648485_L6_PA	fp06_04	6	4.91
FB_0648446_L6_PA	fp06_04	6	4.91
FB_0648821_L6_PA	fp06_05	6	5.336
FB_0880726_L13_46_1	fp06_09	6	9.885
GD_01747_L6_PA	fp06_09	6	9.885
FB_0648798_L6_PA	fp06_09	6	9.885
FB_0648811_L6_PA	fp06_09	6	9.885
FB_0648809_L6_PA	fp06_09	6	9.885
FB_0648823_L6_PA	fp06_09	6	9.885
FB_0648815_L6_PA	fp06_09	6	9.885
FB_0648803_L6_PA	fp06_09	6	9.885
FB_0648817_L6_PA	fp06_09	6	9.885
FB_0649006_L6_PA	fp06_10	6	10.372
FB_0649460_L6_PA	fp06_10	6	10.372
FB_0880721_L13_46_1	fp06_10	6	10.372
FB_0648828_L6_PA	fp06_10	6	10.372
FB_0648999_L6_PA	fp06_10	6	10.372
RB_3299580_L6_PA	fp06_10	6	10.372
FB_0649709_L6_PA	fp06_10	6	10.372
FB_0880710_L13_46_1	fp06_10	6	10.372
FB_0649734_L6_PA	fp06_10	6	10.372
FB_0648985_L6_PA	fp06_10	6	10.372
FB_0649454_L6_PA	fp06_10	6	10.372

FB_0648987_L6_PA	fp06_10	6	10.372
FB_0648994_L6_PA	fp06_10	6	10.372
GD_02819_L6_PA	fp06_10	6	10.372
FB_0649719_L6_PA	fp06_11	6	11.24
FB_0650070_L6_PA	fp06_11	6	11.24
RB_3922847_L6_PA	fp06_11	6	11.24
FB_1096547_L6_48_2	fp06_11	6	11.24
FB_0649711_L6_PA	fp06_11	6	11.24
FB_0650073_L6_PA	fp06_11	6	11.24
RB_3942180_L6_PA	fp06_11	6	11.24
FB_0649736_L6_PA	fp06_11	6	11.24
FB_0650068_L6_PA	fp06_11	6	11.24
FB_0650074_L6_PA	fp06_11	6	11.24
FB_1002939_L6_28_1	fp06_12	6	12.538
FB_1002937_L6_28_1	fp06_12	6	12.538
FB_1096542_L6_48_2	fp06_12	6	12.538
FB_0650072_L6_PA	fp06_12	6	12.538
FB_1002964_L6_28_1	fp06_12	6	12.538
FB_0650067_L6_PA	fp06_12	6	12.538
FB_1002943_L6_28_1	fp06_12	6	12.538
FB_1002983_L6_28_1	fp06_12	6	12.538
FB_1002933_L6_28_1	fp06_12	6	12.538
FB_0651342_L6_PA	fp06_12	6	12.538
FB_1002982_L6_28_1	fp06_12	6	12.538
RB_4578702_L6_PA	fp06_12	6	12.538
FB_1003209_L6_28_1	fp06_12	6	12.538
FB_1003217_L6_28_1	fp06_12	6	12.538
RB_4554020_L6_PA	fp06_12	6	12.538
RB_4558281_L6_PA	fp06_12	6	12.538
FB_1003091_L6_28_1	fp06_12	6	12.538
FB_1003210_L6_28_1	fp06_12	6	12.538
RB_4161930_L6_PA	fp06_12	6	12.538
FB_0650937_L6_PA	fp06_12	6	12.538
GD_00181_L6_28_1	fp06_12	6	12.538
FB_0650931_L6_PA	fp06_12	6	12.538
FB_1003094_L6_28_1	fp06_12	6	12.538
FB_1003215_L6_28_1	fp06_12	6	12.538
FB_0652393_L6_PA	fp06_13	6	13.321
FB_0652380_L6_PA	fp06_13	6	13.321
FB_0651349_L6_PA	fp06_14	6	14.267
FB_0651351_L6_PA	fp06_14	6	14.267
GD_00501_L6_PA	fp06_14	6	14.267
FB_0657008_L6_PA	fp06_14	6	14.267
FB_0651361_L6_PA	fp06_14	6	14.267
FB_0651360_L6_PA	fp06_14	6	14.267
FB_0651340_L6_PA	fp06_14	6	14.267

RB_4566174_L6_PA	fp06_14	6	14.267
RB_4782545_L6_PA	fp06_14	6	14.267
RB_4782504_L6_PA	fp06_14	6	14.267
RB_7877366_L6_PA	fp06_17	6	17.251
FB_0657021_L6_PA	fp06_17	6	17.251
RB_7892794_L6_PA	fp06_17	6	17.251
FB_0657025_L6_PA	fp06_17	6	17.251
RB_4993076_L6_PA	fp06_17	6	17.251
RB_5232792_L6_PA	fp06_18	6	18.464
RB_5243425_L6_PA	fp06_18	6	18.464
FB_0651373_L6_PA	fp06_19	6	19.497
FB_0657032_L6_PA	fp06_19	6	19.497
RB_5249377_L6_PA	fp06_20	6	20.665
FB_0653076_L6_PA	fp06_20	6	20.665
FB_1002959_L6_28_1	fp06_20	6	20.665
FB_0650932_L6_PA	fp06_20	6	20.665
RB_5261397_L6_PA	fp06_20	6	20.665
GD_01954_L6_PA	fp06_20	6	20.665
RB_5246231_L6_PA	fp06_20	6	20.665
RB_5306462_L6_PA	fp06_22	6	22.131
RB_5287964_L6_PA	fp06_22	6	22.131
FB_0653091_L6_PA	fp06_22	6	22.131
FB_0653090_L6_PA	fp06_22	6	22.131
FB_0653078_L6_PA	fp06_22	6	22.131
RB_6615652_L6_PA	fp06_24	6	24.544
RB_6220043_L6_PA	fp06_24	6	24.544
FB_0655917_L6_PA	fp06_25	6	25.878
FB_0653107_L6_PA	fp06_25	6	25.878
FB_0656447_L6_PA	fp06_25	6	25.878
FB_0655287_L6_PA	fp06_25	6	25.878
FB_0654636_L6_PA	fp06_25	6	25.878
FB_0655280_L6_PA	fp06_25	6	25.878
FB_0654624_L6_PA	fp06_25	6	25.878
FB_1063494_L13_50_2	fp06_25	6	25.878
FB_0655936_L6_PA	fp06_25	6	25.878
FB_0654631_L6_PA	fp06_25	6	25.878
FB_0655281_L6_PA	fp06_25	6	25.878
FB_1063506_L13_50_2	fp06_25	6	25.878
FB_0654628_L6_PA	fp06_25	6	25.878
FB_1063518_L13_50_2	fp06_25	6	25.878
FB_1063499_L13_50_2	fp06_25	6	25.878
FB_0655916_L6_PA	fp06_25	6	25.878
FB_0655926_L6_PA	fp06_25	6	25.878
FB_0654630_L6_PA	fp06_25	6	25.878
FB_0655298_L6_PA	fp06_25	6	25.878
FB_0655906_L6_PA	fp06_25	6	25.878

GD_01575_L6_PA	fp06_25	6	25.878
FB_0654638_L6_PA	fp06_25	6	25.878
FB_0655284_L6_PA	fp06_25	6	25.878
FB_1096714_L6_48_2	fp06_25	6	25.878
RB_7311903_L6_PA	fp06_26	6	26.689
FB_0656437_L6_PA	fp06_26	6	26.689
FB_1003995_L6_28_1	fp06_26	6	26.689
FB_1003986_L6_28_1	fp06_26	6	26.689
FB_1003968_L6_28_1	fp06_26	6	26.689
RB_9378475_L6_PA	fp06_27	6	27.48
FB_1063496_L13_50_2	fp06_27	6	27.48
FB_1046552_L9_26_1	fp06_27	6	27.48
FB_0656440_L6_PA	fp06_27	6	27.48
FB_0225031_L14_PA	fp06_27	6	27.48
FB_0225020_L14_PA	fp06_27	6	27.48
FB_0225032_L14_PA	fp06_27	6	27.48
FB_0225016_L14_PA	fp06_27	6	27.48
FB_1070517_L15_108_2	fp06_27	6	27.48
FB_0662268_L6_PA	fp06_28	6	28.568
FB_0662279_L6_PA	fp06_28	6	28.568
RB_10948075_L6_PA	fp06_28	6	28.568
FB_0660470_L6_PA	fp06_28	6	28.568
FB_1097202_L6_55_2	fp06_28	6	28.568
FB_1106141_L9_45_2	fp06_28	6	28.568
FB_0701454_L7_PA	fp06_28	6	28.568
FB_0666652_L6_PA	fp06_28	6	28.568
RB_12007655_L6_PA	fp06_28	6	28.568
FB_0657889_L6_PA	fp06_28	6	28.568
RB_8857634_L6_PA	fp06_28	6	28.568
FB_1006513_L6_37_1	fp06_28	6	28.568
FB_0658098_L6_PA	fp06_28	6	28.568
FB_0658097_L6_PA	fp06_28	6	28.568
FB_0659108_L6_PA	fp06_28	6	28.568
FB_0660573_L6_PA	fp06_28	6	28.568
FB_0225029_L14_PA	fp06_29	6	29.572
FB_0837844_L10_48_1	fp06_29	6	29.572
FB_0659777_L6_PA	fp06_29	6	29.572
FB_0667727_L6_PA	fp06_29	6	29.572
FB_0659119_L6_PA	fp06_30	6	30.528
FB_0659142_L6_PA	fp06_30	6	30.528
FB_0660540_L6_PA	fp06_30	6	30.528
FB_0403176_L17_PA	fp06_30	6	30.528
FB_1097597_L6_57_2	fp06_30	6	30.528
FB_0659786_L6_PA	fp06_30	6	30.528
FB_0659793_L6_PA	fp06_30	6	30.528
FB_0660574_L6_PA	fp06_30	6	30.528

RB_9344917_L6_31_1	fp06_30	6	30.528
FB_0658100_L6_PA	fp06_30	6	30.528
FB_0659121_L6_PA	fp06_30	6	30.528
FB_0659143_L6_PA	fp06_30	6	30.528
FB_0658091_L6_PA	fp06_30	6	30.528
FB_0714630_L7_PA	fp06_30	6	30.528
FB_0403182_L17_PA	fp06_30	6	30.528
FB_0659123_L6_PA	fp06_30	6	30.528
FB_0658099_L6_PA	fp06_30	6	30.528
GD_02258_L6_PA	fp06_30	6	30.528
FB_0659774_L6_PA	fp06_30	6	30.528
FB_0659785_L6_PA	fp06_30	6	30.528
FB_0659794_L6_PA	fp06_30	6	30.528
FB_0658096_L6_PA	fp06_30	6	30.528
FB_0403222_L17_PA	fp06_30	6	30.528
FB_0937056_L17_21_1	fp06_30	6	30.528
FB_0660576_L6_PA	fp06_30	6	30.528
FB_0658087_L6_PA	fp06_30	6	30.528
FB_0403192_L17_PA	fp06_30	6	30.528
FB_1097570_L6_57_2	fp06_30	6	30.528
FB_0659798_L6_PA	fp06_30	6	30.528
FB_0666646_L6_PA	fp06_30	6	30.528
FB_0666662_L6_PA	fp06_30	6	30.528
FB_1006045_L6_36_1	fp06_30	6	30.528
FB_0666725_L6_PA	fp06_30	6	30.528
FB_1106150_L9_45_2	fp06_30	6	30.528
FB_0839829_L11_26_1	fp06_30	6	30.528
FB_1097191_L6_55_2	fp06_30	6	30.528
FB_0660103_L6_PA	fp06_30	6	30.528
FB_0049708_L10_PA	fp06_30	6	30.528
FB_0667724_L6_PA	fp06_30	6	30.528
FB_0701438_L7_PA	fp06_30	6	30.528
FB_1006504_L6_37_1	fp06_30	6	30.528
FB_0667739_L6_PA	fp06_30	6	30.528
FB_1106144_L9_45_2	fp06_30	6	30.528
FB_0666723_L6_PA	fp06_30	6	30.528
FB_0662266_L6_PA	fp06_30	6	30.528
FB_0049709_L10_PA	fp06_30	6	30.528
FB_0049715_L10_PA	fp06_30	6	30.528
FB_0662270_L6_PA	fp06_30	6	30.528
GD_02763_L6_PA	fp06_30	6	30.528
FB_0839860_L11_26_1	fp06_30	6	30.528
FB_0049210_L10_PA	fp06_30	6	30.528
FB_0701428_L7_PA	fp06_30	6	30.528
FB_0837846_L10_48_1	fp06_30	6	30.528
FB_0657883_L6_PA	fp06_30	6	30.528

FB_1097195_L6_55_2	fp06_30	6	30.528
FB_0241589_L14_PA	fp06_30	6	30.528
FB_0657894_L6_PA	fp06_30	6	30.528
FB_1097187_L6_55_2	fp06_30	6	30.528
FB_0660101_L6_PA	fp06_30	6	30.528
FB_1097188_L6_55_2	fp06_30	6	30.528
FB_1006498_L6_37_1	fp06_30	6	30.528
FB_0657903_L6_PA	fp06_30	6	30.528
GD_01366_L6_PA	fp06_30	6	30.528
FB_0234377_L14_PA	fp06_30	6	30.528
FB_0467077_L2_PA	fp06_30	6	30.528
GD_02779_L6_PA	fp06_30	6	30.528
FB_0666653_L6_PA	fp06_30	6	30.528
FB_0657905_L6_PA	fp06_30	6	30.528
FB_0234418_L14_PA	fp06_30	6	30.528
FB_1097359_L6_55_2	fp06_30	6	30.528
FB_1006526_L6_37_1	fp06_30	6	30.528
FB_0049212_L10_PA	fp06_30	6	30.528
FB_1006500_L6_37_1	fp06_30	6	30.528
FB_0891291_L14_41_1	fp06_30	6	30.528
FB_1106149_L9_45_2	fp06_30	6	30.528
FB_0657895_L6_PA	fp06_30	6	30.528
FB_0657886_L6_PA	fp06_30	6	30.528
FB_0660107_L6_PA	fp06_30	6	30.528
FB_0657890_L6_PA	fp06_30	6	30.528
FB_0839833_L11_26_1	fp06_30	6	30.528
FB_0839830_L11_26_1	fp06_30	6	30.528
FB_0657885_L6_PA	fp06_30	6	30.528
FB_1070508_L15_108_2	fp06_31	6	31.734
FB_1070509_L15_108_2	fp06_31	6	31.734
FB_1070506_L15_108_2	fp06_31	6	31.734
FB_1006503_L6_37_1	fp06_31	6	31.734
RB_11991962_L6_PA	fp06_32	6	32.558
FB_0938744_L1_37_1	fp06_32	6	32.558
FB_0416108_L1_PA	fp06_32	6	32.558
FB_0234824_L14_PA	fp06_33	6	33.458
FB_1008755_L6_42_1	fp06_33	6	33.458
FB_1008752_L6_42_1	fp06_33	6	33.458
FB_1008759_L6_42_1	fp06_33	6	33.458
FB_1116730_L6_67_3	fp06_33	6	33.458
FB_0905542_L15_48_1	fp06_33	6	33.458
FB_0668013_L6_PA	fp06_33	6	33.458
FB_0282796_L15_PA	fp06_33	6	33.458
FB_0282767_L15_PA	fp06_33	6	33.458
FB_0282771_L15_PA	fp06_33	6	33.458
FB_0282798_L15_PA	fp06_33	6	33.458

FB_1008766_L6_42_1	fp06_33	6	33.458
FB_0282776_L15_PA	fp06_33	6	33.458
FB_0705281_L7_PA	fp06_33	6	33.458
FB_0416107_L1_PA	fp06_33	6	33.458
FB_0416103_L1_PA	fp06_33	6	33.458
FB_0416083_L1_PA	fp06_33	6	33.458
FB_0666558_L6_PA	fp06_34	6	34.551
FB_1008747_L6_42_1	fp06_34	6	34.551
FB_0049204_L10_PA	fp06_34	6	34.551
FB_0668029_L6_PA	fp06_34	6	34.551
FB_0416137_L1_PA	fp06_35	6	35.759
FB_0942849_L1_51_1	fp06_35	6	35.759
FB_0938739_L1_37_1	fp06_35	6	35.759
FB_0234817_L14_PA	fp06_35	6	35.759
FB_0668775_L6_PA	fp06_35	6	35.759
FB_0669436_L6_PA	fp06_35	6	35.759
FB_0669433_L6_PA	fp06_35	6	35.759
FB_0668502_L6_PA	fp06_35	6	35.759
FB_0668490_L6_PA	fp06_35	6	35.759
FB_0668480_L6_PA	fp06_35	6	35.759
FB_0668778_L6_PA	fp06_35	6	35.759
FB_1007267_L6_40_1	fp06_35	6	35.759
RB_19929848_L6_PA	fp06_35	6	35.759
FB_0668753_L6_PA	fp06_35	6	35.759
FB_0669437_L6_PA	fp06_35	6	35.759
FB_0668498_L6_PA	fp06_35	6	35.759
FB_0668779_L6_PA	fp06_35	6	35.759
FB_0938735_L1_37_1	fp06_36	6	36.346
RB_18628780_L6_PA	fp06_36	6	36.346
FB_0282757_L15_PA	fp06_37	6	37.644
FB_0670348_L6_PA	fp06_37	6	37.644
FB_0668749_L6_PA	fp06_37	6	37.644
FB_0666549_L6_PA	fp06_37	6	37.644
FB_0666550_L6_PA	fp06_37	6	37.644
RB_19604126_L6_PA	fp06_37	6	37.644
RB_19565705_L6_PA	fp06_37	6	37.644
FB_0668511_L6_PA	fp06_37	6	37.644
RB_20446572_L6_PA	fp06_38	6	38.455
RB_20453026_L6_PA	fp06_38	6	38.455
FB_0670051_L6_PA	fp06_38	6	38.455
FB_0670037_L6_PA	fp06_38	6	38.455
GD_01831_L6_PA	fp06_38	6	38.455
FB_1007351_L6_40_1	fp06_38	6	38.455
FB_0670019_L6_PA	fp06_38	6	38.455
FB_0670017_L6_PA	fp06_38	6	38.455
FB_0670053_L6_PA	fp06_38	6	38.455

FB_1007333_L6_40_1	fp06_38	6	38.455
FB_0693130_L7_PA	fp06_38	6	38.455
FB_0082921_L11_PA	fp06_38	6	38.455
FB_1013525_L7_27_1	fp06_38	6	38.455
FB_1007755_L6_41_1	fp06_38	6	38.455
FB_0693659_L7_PA	fp06_38	6	38.455
FB_0671026_L6_PA	fp06_39	6	39.489
FB_0671030_L6_PA	fp06_39	6	39.489
FB_0692516_L7_PA	fp06_39	6	39.489
FB_1007763_L6_41_1	fp06_39	6	39.489
FB_0692535_L7_PA	fp06_39	6	39.489
RB_21088776_L6_PA	fp06_39	6	39.489
FB_0692547_L7_PA	fp06_39	6	39.489
FB_0671033_L6_PA	fp06_40	6	40.617
FB_0670819_L6_PA	fp06_40	6	40.617
FB_0670821_L6_PA	fp06_40	6	40.617
FB_0670812_L6_PA	fp06_40	6	40.617
FB_0672940_L6_PA	fp06_40	6	40.617
FB_0672157_L6_PA	fp06_40	6	40.617
RB_21699479_L6_PA	fp06_40	6	40.617
RB_21693978_L6_PA	fp06_40	6	40.617
FB_1097691_L6_58_2	fp06_40	6	40.617
RB_21134048_L6_PA	fp06_40	6	40.617
FB_1097692_L6_58_2	fp06_40	6	40.617
RB_21088744_L6_PA	fp06_40	6	40.617
FB_0671133_L6_PA	fp06_40	6	40.617
FB_0672949_L6_PA	fp06_40	6	40.617
FB_1007766_L6_41_1	fp06_40	6	40.617
RB_21677670_L6_PA	fp06_40	6	40.617
FB_1007772_L6_41_1	fp06_40	6	40.617
FB_0672169_L6_PA	fp06_41	6	41.587
FB_0672140_L6_PA	fp06_41	6	41.587
FB_0672143_L6_PA	fp06_41	6	41.587
GD_00094_L6_PA	fp06_41	6	41.587
FB_0962602_L2_37_1	fp06_41	6	41.587
FB_0962583_L2_37_1	fp06_41	6	41.587
GD_02165_L6_PA	fp06_41	6	41.587
RB_22247625_L6_PA	fp06_42	6	42.254
FB_0672962_L6_PA	fp06_42	6	42.254
RB_22245579_L6_PA	fp06_45	6	45.691
RB_23462644_L6_PA	fp06_45	6	45.691
RB_23415983_L6_PA	fp06_45	6	45.691
RB_23422688_L6_PA	fp06_45	6	45.691
FB_0675044_L6_PA	fp06_46	6	46.92
FB_0796354_L9_PA	fp06_46	6	46.92
FB_1009076_L6_42_1	fp06_46	6	46.92

RB_23402231_L6_PA	fp06_47	6	47.489
RB_23409284_L6_PA	fp06_47	6	47.489
FB_0994689_L5_42_1	fp06_47	6	47.489
FB_0796336_L9_PA	fp06_47	6	47.489
FB_0962604_L2_37_1	fp06_48	6	48.437
FB_0358604_L16_PA	fp06_48	6	48.437
FB_0674537_L6_PA	fp06_48	6	48.437
FB_0796334_L9_PA	fp06_49	6	49.448
FB_0675013_L6_PA	fp06_49	6	49.448
RB_23651920_L6_PA	fp06_50	6	50.661
FB_0677176_L6_PA	fp06_50	6	50.661
FB_0677178_L6_PA	fp06_50	6	50.661
FB_1009078_L6_42_1	fp06_50	6	50.661
FB_0675027_L6_PA	fp06_50	6	50.661
FB_0674518_L6_PA	fp06_50	6	50.661
GD_01556_L6_PA	fp06_50	6	50.661
FB_0674547_L6_PA	fp06_50	6	50.661
FB_0675046_L6_PA	fp06_50	6	50.661
FB_0674520_L6_PA	fp06_50	6	50.661
RB_23453666_L6_PA	fp06_50	6	50.661
FB_0674533_L6_PA	fp06_50	6	50.661
FB_0675024_L6_PA	fp06_50	6	50.661
FB_1009077_L6_42_1	fp06_50	6	50.661
FB_0674534_L6_PA	fp06_50	6	50.661
FB_0674514_L6_PA	fp06_50	6	50.661
FB_0674538_L6_PA	fp06_50	6	50.661
FB_1009075_L6_42_1	fp06_50	6	50.661
FB_1009088_L6_42_1	fp06_50	6	50.661
FB_1009080_L6_42_1	fp06_50	6	50.661
FB_1118742_L9_58_3	fp06_50	6	50.661
FB_0675014_L6_PA	fp06_50	6	50.661
FB_1105992_L9_44_2	fp06_51	6	51.82
FB_1106026_L9_44_2	fp06_51	6	51.82
FB_1118736_L9_58_3	fp06_51	6	51.82
FB_1118738_L9_58_3	fp06_51	6	51.82
FB_1106001_L9_44_2	fp06_51	6	51.82
FB_1118743_L9_58_3	fp06_51	6	51.82
FB_0676802_L6_PA	fp06_52	6	52.136
FB_0676097_L6_PA	fp06_53	6	53.833
FB_1009623_L6_43_1	fp06_57	6	57.756
FB_0676793_L6_PA	fp06_58	6	58.703
FB_0676804_L6_PA	fp06_58	6	58.703
FB_0676805_L6_PA	fp06_58	6	58.703
FB_0676791_L6_PA	fp06_58	6	58.703
FB_0676807_L6_PA	fp06_58	6	58.703
FB_1009613_L6_43_1	fp06_59	6	59.17

FB_0677173_L6_PA	fp06_59	6	59.17
FB_0677189_L6_PA	fp06_59	6	59.17
RB_25352404_L6_PA	fp06_59	6	59.17
RB_25205098_L6_PA	fp06_59	6	59.17
FB_0677196_L6_PA	fp06_59	6	59.17
RB_25356570_L6_PA	fp06_59	6	59.17
FB_0676812_L6_PA	fp06_59	6	59.17
FB_0677455_L6_PA	fp06_59	6	59.17
FB_0677200_L6_PA	fp06_59	6	59.17
FB_1098131_L6_60_2	fp06_59	6	59.17
GD_01444_L6_PA	fp06_59	6	59.17
FB_0677198_L6_PA	fp06_59	6	59.17
FB_0677210_L6_PA	fp06_59	6	59.17
GD_01502_L6_PA	fp06_59	6	59.17
RB_25319820_L6_PA	fp06_59	6	59.17
FB_0677206_L6_PA	fp06_59	6	59.17
FB_0677867_L6_PA	fp06_60	6	60.548
FB_0677857_L6_PA	fp06_60	6	60.548
FB_0677868_L6_PA	fp06_60	6	60.548
FB_0677858_L6_PA	fp06_60	6	60.548
FB_0677865_L6_PA	fp06_60	6	60.548
RB_26018605_L6_PA	fp06_60	6	60.548
RB_26013420_L6_PA	fp06_61	6	61.686
RB_26016395_L6_PA	fp06_62	6	62.333
RB_26032137_L6_PA	fp06_62	6	62.333
GD_01682_L6_PA	fp06_62	6	62.333
RB_26007783_L6_PA	fp06_62	6	62.333
FB_0679742_L6_PA	fp06_63	6	63.835
FB_0680630_L6_PA	fp06_63	6	63.835
FB_0680218_L6_PA	fp06_64	6	64.646
FB_0679730_L6_PA	fp06_64	6	64.646
FB_0679410_L6_PA	fp06_64	6	64.646
FB_0679753_L6_PA	fp06_64	6	64.646
FB_0679434_L6_PA	fp06_64	6	64.646
FB_0679420_L6_PA	fp06_64	6	64.646
FB_0679736_L6_PA	fp06_64	6	64.646
FB_0677866_L6_PA	fp06_64	6	64.646
FB_0681947_L6_PA	fp06_64	6	64.646
FB_0681286_L6_PA	fp06_64	6	64.646
FB_0680617_L6_PA	fp06_64	6	64.646
FB_0681295_L6_PA	fp06_64	6	64.646
RB_27462705_L6_PA	fp06_64	6	64.646
RB_27497747_L6_PA	fp06_64	6	64.646
RB_27832806_L6_PA	fp06_64	6	64.646
FB_0680618_L6_PA	fp06_64	6	64.646
RB_27359073_L6_PA	fp06_64	6	64.646

FB_0681682_L6_PA	fp06_64	6	64.646
RB_27796239_L6_PA	fp06_64	6	64.646
RB_27324354_L6_43_1	fp06_64	6	64.646
FB_0681700_L6_PA	fp06_64	6	64.646
FB_0681689_L6_PA	fp06_64	6	64.646
FB_0681944_L6_PA	fp06_64	6	64.646
RB_27866211_L6_PA	fp06_64	6	64.646
RB_27308621_L6_PA	fp06_64	6	64.646
FB_0680619_L6_PA	fp06_64	6	64.646
RB_27468669_L6_PA	fp06_64	6	64.646
RB_27798613_L6_PA	fp06_64	6	64.646
FB_0680998_L6_PA	fp06_64	6	64.646
RB_27322069_L6_43_1	fp06_64	6	64.646
FB_0680999_L6_PA	fp06_64	6	64.646
FB_0680632_L6_PA	fp06_64	6	64.646
FB_0681688_L6_PA	fp06_64	6	64.646
FB_0681965_L6_PA	fp06_65	6	65.77
FB_0681964_L6_PA	fp06_65	6	65.77
FB_0681963_L6_PA	fp06_65	6	65.77
FB_1010944_L6_43_1	fp06_65	6	65.77
FB_1010942_L6_43_1	fp06_65	6	65.77
FB_1010961_L6_43_1	fp06_65	6	65.77
FB_0682399_L6_PA	fp06_65	6	65.77
FB_1010939_L6_43_1	fp06_66	6	66.367
RB_28643044_L6_PA	fp06_66	6	66.367
FB_0682944_L6_PA	fp06_66	6	66.367
FB_1010950_L6_43_1	fp06_66	6	66.367
RB_28643186_L6_PA	fp06_66	6	66.367
RB_28599216_L6_PA	fp06_66	6	66.367
RB_28845868_L6_PA	fp06_66	6	66.367
RB_28643077_L6_PA	fp06_66	6	66.367
FB_0682393_L6_PA	fp06_66	6	66.367
RB_28837573_L6_PA	fp06_66	6	66.367
RB_28870265_L6_PA	fp06_66	6	66.367
FB_0682398_L6_PA	fp06_66	6	66.367
RB_28597456_L6_PA	fp06_66	6	66.367
RB_28848270_L6_PA	fp06_66	6	66.367
RB_28598169_L6_PA	fp06_66	6	66.367
FB_0682391_L6_PA	fp06_66	6	66.367
RB_28598904_L6_PA	fp06_66	6	66.367
FB_0683249_L6_PA	fp06_66	6	66.367
FB_0682946_L6_PA	fp06_66	6	66.367
FB_0682945_L6_PA	fp06_66	6	66.367
FB_0682938_L6_PA	fp06_66	6	66.367
FB_0682939_L6_PA	fp06_66	6	66.367
FB_0682956_L6_PA	fp06_66	6	66.367

FB_0682394_L6_PA	fp06_66	6	66.367
FB_0681691_L6_PA	fp06_67	6	67.608
FB_0682960_L6_PA	fp06_67	6	67.608
FB_0685588_L6_PA	fp06_67	6	67.608
FB_0682940_L6_PA	fp06_67	6	67.608
RB_30141206_L6_PA	fp06_67	6	67.608
FB_0683269_L6_PA	fp06_67	6	67.608
FB_0684769_L6_PA	fp06_67	6	67.608
FB_0683257_L6_PA	fp06_67	6	67.608
FB_0684770_L6_PA	fp06_67	6	67.608
FB_0683272_L6_PA	fp06_67	6	67.608
FB_0683254_L6_PA	fp06_67	6	67.608
RB_30080885_L6_PA	fp06_67	6	67.608
RB_30289424_L6_PA	fp06_67	6	67.608
RB_30289276_L6_PA	fp06_67	6	67.608
FB_0685570_L6_PA	fp06_68	6	68.328
GD_02138_L6_PA	fp06_68	6	68.328
RB_30289681_L6_PA	fp06_68	6	68.328
RB_30154941_L6_PA	fp06_71	6	71.023
RB_30075128_L6_PA	fp06_71	6	71.023
FB_0685573_L6_PA	fp06_72	6	72.228
FB_0685576_L6_PA	fp06_72	6	72.228
FB_0685652_L7_PA	fp07_00	7	0.142
FB_1011799_L7_26_1	fp07_00	7	0.142
FB_1011789_L7_26_1	fp07_00	7	0.142
FB_1011785_L7_26_1	fp07_00	7	0.142
FB_0686545_L7_PA	fp07_00	7	0.142
FB_0686541_L7_PA	fp07_00	7	0.142
FB_0686534_L7_PA	fp07_00	7	0.142
FB_0685669_L7_PA	fp07_00	7	0.142
FB_1011780_L7_26_1	fp07_00	7	0.142
FB_1011776_L7_26_1	fp07_00	7	0.142
FB_0685657_L7_PA	fp07_00	7	0.142
FB_0686538_L7_PA	fp07_00	7	0.142
RB_839755_L7_PA	fp07_00	7	0.142
FB_0685731_L7_PA	fp07_00	7	0.142
FB_0686539_L7_PA	fp07_00	7	0.142
FB_1011781_L7_26_1	fp07_00	7	0.142
FB_0685738_L7_PA	fp07_00	7	0.142
FB_0687241_L7_PA	fp07_00	7	0.142
FB_1011778_L7_26_1	fp07_00	7	0.142
GD_01172_L7_PA	fp07_00	7	0.142
FB_0685741_L7_PA	fp07_00	7	0.142
FB_0685723_L7_PA	fp07_00	7	0.142
RB_1157629_L7_PA	fp07_00	7	0.142
FB_0688056_L7_PA	fp07_00	7	0.142

FB_1011791_L7_26_1	fp07_00	7	0.142
FB_0687224_L7_PA	fp07_00	7	0.142
FB_0687236_L7_PA	fp07_01	7	1.2
FB_0685724_L7_PA	fp07_01	7	1.2
FB_0687237_L7_PA	fp07_01	7	1.2
FB_1011790_L7_26_1	fp07_01	7	1.2
FB_0687225_L7_PA	fp07_01	7	1.2
FB_0688076_L7_PA	fp07_01	7	1.2
FB_0687247_L7_PA	fp07_02	7	2.511
FB_0688429_L7_PA	fp07_02	7	2.511
FB_0688834_L7_PA	fp07_02	7	2.511
FB_0687256_L7_PA	fp07_02	7	2.511
FB_0688837_L7_PA	fp07_03	7	3.684
RB_1804847_L7_PA	fp07_03	7	3.684
FB_0688432_L7_PA	fp07_03	7	3.684
FB_0688049_L7_PA	fp07_03	7	3.684
RB_1145997_L7_PA	fp07_03	7	3.684
RB_1112883_L7_26_1	fp07_03	7	3.684
RB_2183945_L7_PA	fp07_03	7	3.684
FB_0688459_L7_PA	fp07_03	7	3.684
FB_0688075_L7_PA	fp07_04	7	4.201
FB_0688428_L7_PA	fp07_04	7	4.201
FB_0689282_L7_PA	fp07_04	7	4.201
FB_0688045_L7_PA	fp07_04	7	4.201
RB_2838006_L7_PA	fp07_04	7	4.201
FB_0688833_L7_PA	fp07_04	7	4.201
RB_2832830_L7_PA	fp07_04	7	4.201
FB_0689284_L7_PA	fp07_04	7	4.201
FB_0688820_L7_PA	fp07_04	7	4.201
FB_0688048_L7_PA	fp07_04	7	4.201
RB_2860536_L7_PA	fp07_04	7	4.201
RB_2869226_L7_PA	fp07_04	7	4.201
RB_1819499_L7_PA	fp07_04	7	4.201
FB_0688824_L7_PA	fp07_04	7	4.201
RB_2851448_L7_PA	fp07_04	7	4.201
GD_00802_L7_PA	fp07_04	7	4.201
RB_2244430_L7_PA	fp07_04	7	4.201
FB_0688821_L7_PA	fp07_04	7	4.201
FB_0688810_L7_PA	fp07_04	7	4.201
FB_0688420_L7_PA	fp07_04	7	4.201
FB_0688078_L7_PA	fp07_04	7	4.201
RB_2872650_L7_PA	fp07_04	7	4.201
FB_0769828_L9_PA	fp07_04	7	4.201
FB_0689287_L7_PA	fp07_04	7	4.201
FB_0690038_L7_PA	fp07_05	7	5.604
FB_0769830_L9_PA	fp07_05	7	5.604

FB_0691778_L7_PA	fp07_05	7	5.604
FB_0690055_L7_PA	fp07_05	7	5.604
RB_3558655_L7_PA	fp07_06	7	6.852
FB_0690677_L7_PA	fp07_06	7	6.852
FB_0690054_L7_PA	fp07_06	7	6.852
FB_0690680_L7_PA	fp07_06	7	6.852
FB_0690906_L7_PA	fp07_06	7	6.852
FB_0690702_L7_PA	fp07_06	7	6.852
FB_0690913_L7_PA	fp07_07	7	7.411
FB_0691955_L7_PA	fp07_07	7	7.411
FB_0690918_L7_PA	fp07_07	7	7.411
FB_0690673_L7_PA	fp07_07	7	7.411
FB_0691039_L7_PA	fp07_07	7	7.411
FB_0690694_L7_PA	fp07_07	7	7.411
GD_01717_L7_PA	fp07_07	7	7.411
FB_0691049_L7_PA	fp07_08	7	8.722
FB_0690914_L7_PA	fp07_08	7	8.722
FB_0691041_L7_PA	fp07_08	7	8.722
FB_0691051_L7_PA	fp07_09	7	9.564
RB_3862604_L7_PA	fp07_10	7	10.375
RB_3864649_L7_PA	fp07_10	7	10.375
RB_5831303_L7_PA	fp07_10	7	10.375
RB_4120885_L7_PA	fp07_10	7	10.375
RB_4377413_L7_PA	fp07_10	7	10.375
RB_4155923_L7_PA	fp07_11	7	11.546
FB_0691770_L7_PA	fp07_11	7	11.546
RB_4372277_L7_PA	fp07_11	7	11.546
RB_4175763_L7_PA	fp07_11	7	11.546
FB_0691971_L7_PA	fp07_11	7	11.546
FB_0691784_L7_PA	fp07_11	7	11.546
FB_0691765_L7_PA	fp07_11	7	11.546
FB_0691766_L7_PA	fp07_11	7	11.546
FB_0691964_L7_PA	fp07_11	7	11.546
FB_0691946_L7_PA	fp07_11	7	11.546
FB_0691937_L7_PA	fp07_11	7	11.546
RB_4381600_L7_PA	fp07_11	7	11.546
FB_0691956_L7_PA	fp07_11	7	11.546
FB_0694688_L7_PA	fp07_12	7	12.281
GD_00658_L7_PA	fp07_12	7	12.281
FB_0693782_L7_PA	fp07_13	7	13.297
FB_0693784_L7_PA	fp07_13	7	13.297
FB_1013916_L7_28_1	fp07_14	7	14.334
FB_1013870_L7_28_1	fp07_14	7	14.334
FB_1013914_L7_28_1	fp07_14	7	14.334
FB_1013871_L7_28_1	fp07_14	7	14.334
RB_5842594_L7_PA	fp07_14	7	14.334

FB_0694687_L7_PA	fp07_15	7	15.787
RB_7097620_L7_PA	fp07_15	7	15.787
RB_6171256_L7_PA	fp07_16	7	16.72
FB_1014389_L7_28_1	fp07_16	7	16.72
GD_01872_L7_PA	fp07_17	7	17.705
FB_1014386_L7_28_1	fp07_17	7	17.705
FB_1014394_L7_28_1	fp07_17	7	17.705
RB_7265453_L7_PA	fp07_19	7	19.551
RB_7347591_L7_PA	fp07_19	7	19.551
FB_0696918_L7_PA	fp07_20	7	20.8
GD_02619_L7_PA	fp07_20	7	20.8
FB_0696573_L7_PA	fp07_20	7	20.8
FB_1116594_L6_66_3	fp07_20	7	20.8
FB_0696421_L7_PA	fp07_20	7	20.8
FB_0696565_L7_PA	fp07_20	7	20.8
GD_02853_L7_PA	fp07_20	7	20.8
RB_8158759_L7_PA	fp07_20	7	20.8
FB_0696567_L7_PA	fp07_20	7	20.8
FB_1116603_L6_66_3	fp07_20	7	20.8
FB_0696570_L7_PA	fp07_20	7	20.8
FB_0696431_L7_PA	fp07_20	7	20.8
FB_0696576_L7_PA	fp07_20	7	20.8
FB_0696557_L7_PA	fp07_20	7	20.8
RB_7992834_L7_PA	fp07_20	7	20.8
FB_1116599_L6_66_3	fp07_20	7	20.8
FB_1116597_L6_66_3	fp07_21	7	21.567
FB_0696921_L7_PA	fp07_21	7	21.567
FB_0696910_L7_PA	fp07_21	7	21.567
FB_0696422_L7_PA	fp07_21	7	21.567
FB_0696445_L7_PA	fp07_21	7	21.567
GD_01780_L7_PA	fp07_21	7	21.567
FB_1079748_L17_33_2	fp07_22	7	22.222
FB_1014922_L7_31_1	fp07_22	7	22.222
FB_0697476_L7_PA	fp07_22	7	22.222
FB_0697169_L7_PA	fp07_22	7	22.222
FB_1079752_L17_33_2	fp07_22	7	22.222
FB_1079740_L17_33_2	fp07_22	7	22.222
FB_0697477_L7_PA	fp07_22	7	22.222
FB_0697478_L7_PA	fp07_22	7	22.222
FB_0697187_L7_PA	fp07_22	7	22.222
FB_1079735_L17_33_2	fp07_22	7	22.222
RB_8490690_L7_PA	fp07_22	7	22.222
FB_0696912_L7_PA	fp07_22	7	22.222
RB_8295164_L7_PA	fp07_22	7	22.222
FB_0696914_L7_PA	fp07_22	7	22.222
FB_0696913_L7_PA	fp07_22	7	22.222

RB_9298084_L7_PA	fp07_22	7	22.222
FB_0698135_L7_PA	fp07_22	7	22.222
FB_0698141_L7_PA	fp07_22	7	22.222
FB_0698132_L7_PA	fp07_22	7	22.222
FB_0014364_L10_PA	fp07_23	7	23.505
FB_1014403_L7_28_1	fp07_23	7	23.505
FB_1015074_L7_31_1	fp07_24	7	24.31
FB_0699141_L7_PA	fp07_25	7	25.69
FB_0578713_L4_PA	fp07_25	7	25.69
RB_9956304_L7_PA	fp07_26	7	26.44
FB_0015267_L10_PA	fp07_26	7	26.44
FB_0699126_L7_PA	fp07_26	7	26.44
FB_1015413_L7_32_1	fp07_26	7	26.44
FB_0015266_L10_PA	fp07_26	7	26.44
FB_0829231_L10_35_1	fp07_26	7	26.44
FB_0699127_L7_PA	fp07_26	7	26.44
FB_0829097_L10_35_1	fp07_26	7	26.44
FB_0829100_L10_35_1	fp07_26	7	26.44
FB_0699139_L7_PA	fp07_26	7	26.44
FB_0485717_L2_PA	fp07_26	7	26.44
FB_0698914_L7_PA	fp07_26	7	26.44
FB_0578723_L4_PA	fp07_27	7	27.345
FB_0578731_L4_PA	fp07_27	7	27.345
FB_0446521_L1_PA	fp07_27	7	27.345
FB_0699147_L7_PA	fp07_27	7	27.345
FB_0578719_L4_PA	fp07_27	7	27.345
FB_1095794_L5_74_2	fp07_27	7	27.345
FB_0578705_L4_PA	fp07_27	7	27.345
FB_0578716_L4_PA	fp07_27	7	27.345
FB_0578707_L4_PA	fp07_27	7	27.345
FB_0578718_L4_PA	fp07_27	7	27.345
FB_0578715_L4_PA	fp07_27	7	27.345
FB_0578706_L4_PA	fp07_27	7	27.345
FB_0699339_L7_PA	fp07_27	7	27.345
FB_0377017_L17_PA	fp07_28	7	28.764
FB_0699351_L7_PA	fp07_28	7	28.764
FB_0699344_L7_PA	fp07_28	7	28.764
FB_0699343_L7_PA	fp07_28	7	28.764
RB_11050637_L7_PA	fp07_28	7	28.764
FB_1015565_L7_33_1	fp07_28	7	28.764
RB_11001915_L7_PA	fp07_28	7	28.764
FB_0959726_L2_35_1	fp07_28	7	28.764
FB_0959721_L2_35_1	fp07_28	7	28.764
FB_0699337_L7_PA	fp07_28	7	28.764
RB_11025706_L7_PA	fp07_28	7	28.764
GD_01882_L7_PA	fp07_29	7	29.587

FB_0700766_L7_PA	fp07_29	7	29.587
FB_0699897_L7_PA	fp07_29	7	29.587
FB_0699908_L7_PA	fp07_29	7	29.587
FB_0699905_L7_PA	fp07_29	7	29.587
FB_0700801_L7_PA	fp07_29	7	29.587
FB_0700773_L7_PA	fp07_29	7	29.587
GD_02293_L7_PA	fp07_29	7	29.587
FB_0699925_L7_PA	fp07_29	7	29.587
FB_0699892_L7_PA	fp07_29	7	29.587
FB_0929879_L17_13_1	fp07_29	7	29.587
FB_0699920_L7_PA	fp07_29	7	29.587
FB_0626003_L5_PA	fp07_29	7	29.587
FB_0625508_L5_PA	fp07_29	7	29.587
FB_0625502_L5_PA	fp07_29	7	29.587
FB_0626031_L5_PA	fp07_29	7	29.587
FB_1015621_L7_33_1	fp07_29	7	29.587
FB_1015626_L7_33_1	fp07_29	7	29.587
RB_11386714_L7_PA	fp07_29	7	29.587
FB_0700791_L7_PA	fp07_29	7	29.587
RB_11718356_L7_PA	fp07_29	7	29.587
FB_0377016_L17_PA	fp07_29	7	29.587
FB_0626011_L5_PA	fp07_30	7	30.365
FB_0626029_L5_PA	fp07_30	7	30.365
FB_0929880_L17_13_1	fp07_30	7	30.365
FB_0377010_L17_PA	fp07_30	7	30.365
FB_0703197_L7_PA	fp07_30	7	30.365
FB_0625513_L5_PA	fp07_30	7	30.365
FB_0625518_L5_PA	fp07_30	7	30.365
FB_0318108_L15_PA	fp07_31	7	31.815
FB_0913550_L15_72_1	fp07_31	7	31.815
FB_0318104_L15_PA	fp07_31	7	31.815
FB_0318109_L15_PA	fp07_31	7	31.815
FB_0318112_L15_PA	fp07_31	7	31.815
FB_1002149_L5_59_1	fp07_31	7	31.815
FB_1002134_L5_59_1	fp07_31	7	31.815
FB_0377009_L17_PA	fp07_31	7	31.815
FB_0703347_L7_PA	fp07_31	7	31.815
FB_0703196_L7_PA	fp07_32	7	32.391
FB_0703360_L7_PA	fp07_32	7	32.391
FB_0418734_L1_PA	fp07_32	7	32.391
FB_1016517_L7_38_1	fp07_32	7	32.391
FB_0703212_L7_PA	fp07_32	7	32.391
FB_0703190_L7_PA	fp07_32	7	32.391
FB_0703574_L7_PA	fp07_32	7	32.391
FB_0703594_L7_PA	fp07_32	7	32.391
FB_0702650_L7_PA	fp07_32	7	32.391

FB_1016521_L7_38_1	fp07_32	7	32.391
FB_0702646_L7_PA	fp07_32	7	32.391
FB_0703193_L7_PA	fp07_32	7	32.391
FB_0701846_L7_PA	fp07_32	7	32.391
FB_0701593_L7_PA	fp07_32	7	32.391
FB_0702648_L7_PA	fp07_32	7	32.391
FB_0703187_L7_PA	fp07_32	7	32.391
GD_02840_L7_PA	fp07_32	7	32.391
FB_0702647_L7_PA	fp07_32	7	32.391
FB_1016527_L7_38_1	fp07_33	7	33.096
GD_01305_L7_PA	fp07_33	7	33.096
FB_0704610_L7_PA	fp07_33	7	33.096
FB_1017167_L7_39_1	fp07_33	7	33.096
FB_1017184_L7_39_1	fp07_34	7	34.299
GD_02657_L7_PA	fp07_34	7	34.299
FB_0707354_L7_PA	fp07_34	7	34.299
FB_1017176_L7_39_1	fp07_34	7	34.299
FB_0705584_L7_PA	fp07_34	7	34.299
FB_0704608_L7_PA	fp07_34	7	34.299
FB_0318983_L15_PA	fp07_35	7	35.076
FB_0318978_L15_PA	fp07_35	7	35.076
FB_0318975_L15_PA	fp07_35	7	35.076
FB_0318997_L15_PA	fp07_35	7	35.076
FB_0913547_L15_72_1	fp07_35	7	35.076
FB_0318980_L15_PA	fp07_35	7	35.076
FB_0318977_L15_PA	fp07_35	7	35.076
FB_0913551_L15_72_1	fp07_35	7	35.076
FB_0318979_L15_PA	fp07_35	7	35.076
RB_18336417_L7_PA	fp07_35	7	35.076
FB_0318974_L15_PA	fp07_35	7	35.076
FB_0707360_L7_PA	fp07_36	7	36.407
FB_1017621_L7_40_1	fp07_36	7	36.407
FB_0707648_L7_PA	fp07_36	7	36.407
FB_0706807_L7_PA	fp07_36	7	36.407
FB_0909363_L15_59_1	fp07_36	7	36.407
GD_02875_L7_PA	fp07_36	7	36.407
FB_0706809_L7_PA	fp07_36	7	36.407
FB_0707645_L7_PA	fp07_36	7	36.407
FB_0707351_L7_PA	fp07_36	7	36.407
FB_0707641_L7_PA	fp07_36	7	36.407
FB_0706813_L7_PA	fp07_36	7	36.407
FB_0706822_L7_PA	fp07_36	7	36.407
FB_0706826_L7_PA	fp07_36	7	36.407
FB_0706828_L7_PA	fp07_36	7	36.407
FB_0706817_L7_PA	fp07_36	7	36.407
FB_0709120_L7_PA	fp07_36	7	36.407

FB_0708287_L7_PA	fp07_36	7	36.407
FB_1018052_L7_42_1	fp07_36	7	36.407
FB_0708291_L7_PA	fp07_36	7	36.407
FB_1018059_L7_42_1	fp07_36	7	36.407
GD_00871_L7_PA	fp07_36	7	36.407
FB_1018075_L7_42_1	fp07_36	7	36.407
FB_1018062_L7_42_1	fp07_36	7	36.407
FB_1018047_L7_42_1	fp07_36	7	36.407
FB_1017858_L7_41_1	fp07_36	7	36.407
FB_1017854_L7_41_1	fp07_36	7	36.407
FB_1017860_L7_41_1	fp07_36	7	36.407
FB_1017868_L7_41_1	fp07_36	7	36.407
FB_1018076_L7_42_1	fp07_36	7	36.407
RB_19500458_L7_PA	fp07_36	7	36.407
FB_1017624_L7_40_1	fp07_36	7	36.407
FB_0708282_L7_PA	fp07_36	7	36.407
FB_0709117_L7_PA	fp07_37	7	37.421
FB_0709116_L7_PA	fp07_37	7	37.421
FB_1017605_L7_40_1	fp07_37	7	37.421
FB_0709759_L7_PA	fp07_38	7	38.499
FB_0710390_L7_PA	fp07_38	7	38.499
FB_0709763_L7_PA	fp07_38	7	38.499
GD_02600_L7_PA	fp07_39	7	39.618
RB_21096889_L7_PA	fp07_39	7	39.618
RB_21096895_L7_PA	fp07_39	7	39.618
FB_0709115_L7_PA	fp07_40	7	40.118
FB_0711547_L7_PA	fp07_40	7	40.118
FB_1018189_L7_43_1	fp07_40	7	40.118
FB_0710373_L7_PA	fp07_40	7	40.118
FB_1080864_L1_76_2	fp07_42	7	42.907
FB_1080865_L1_76_2	fp07_42	7	42.907
FB_0710378_L7_PA	fp07_42	7	42.907
FB_0710852_L7_PA	fp07_42	7	42.907
FB_1018186_L7_43_1	fp07_42	7	42.907
FB_0710859_L7_PA	fp07_42	7	42.907
FB_0710858_L7_PA	fp07_42	7	42.907
FB_0710384_L7_PA	fp07_42	7	42.907
FB_0712384_L7_PA	fp07_43	7	43.637
FB_1018867_L7_46_1	fp07_43	7	43.637
FB_0712838_L7_PA	fp07_44	7	44.349
FB_0712832_L7_PA	fp07_44	7	44.349
FB_0731780_L8_PA	fp07_47	7	47.281
FB_0731799_L8_PA	fp07_47	7	47.281
FB_0942386_L1_51_1	fp07_47	7	47.281
FB_0942391_L1_51_1	fp07_47	7	47.281
FB_0942385_L1_51_1	fp07_47	7	47.281

FB_0713521_L7_PA	fp07_47	7	47.281
FB_0713524_L7_PA	fp07_47	7	47.281
FB_0713535_L7_PA	fp07_47	7	47.281
FB_1080867_L1_76_2	fp07_48	7	48.315
FB_1080866_L1_76_2	fp07_48	7	48.315
FB_1025495_L8_36_1	fp07_48	7	48.315
FB_1025503_L8_36_1	fp07_48	7	48.315
FB_1025479_L8_36_1	fp07_48	7	48.315
FB_1025660_L8_36_1	fp07_49	7	49.504
FB_1025658_L8_36_1	fp07_49	7	49.504
FB_0007625_L10_PA	fp07_49	7	49.504
FB_0732365_L8_PA	fp07_50	7	50.593
FB_0732403_L8_PA	fp07_50	7	50.593
FB_0732399_L8_PA	fp07_50	7	50.593
RB_25209129_L7_PA	fp07_50	7	50.593
FB_1025811_L8_36_1	fp07_50	7	50.593
RB_25190375_L7_PA	fp07_50	7	50.593
FB_1025808_L8_36_1	fp07_50	7	50.593
FB_1025815_L8_36_1	fp07_50	7	50.593
FB_1025804_L8_36_1	fp07_50	7	50.593
RB_25206470_L7_PA	fp07_50	7	50.593
GD_02291_L7_47_1	fp07_51	7	51.458
FB_0007637_L10_PA	fp07_51	7	51.458
FB_0713852_L7_PA	fp07_51	7	51.458
RB_25194017_L7_PA	fp07_51	7	51.458
FB_0713851_L7_PA	fp07_51	7	51.458
FB_0007623_L10_PA	fp07_51	7	51.458
RB_25223845_L7_PA	fp07_51	7	51.458
FB_0713830_L7_PA	fp07_51	7	51.458
FB_0007631_L10_PA	fp07_51	7	51.458
FB_1025669_L8_36_1	fp07_51	7	51.458
FB_0053410_L10_PA	fp07_52	7	52.718
FB_1093628_L5_60_2	fp07_53	7	53.542
FB_1093632_L5_60_2	fp07_53	7	53.542
FB_0486765_L2_PA	fp07_53	7	53.542
FB_0053401_L10_PA	fp07_53	7	53.542
FB_0432458_L1_PA	fp07_53	7	53.542
FB_0596791_L5_PA	fp07_53	7	53.542
FB_0432447_L1_PA	fp07_53	7	53.542
FB_0431636_L1_PA	fp07_53	7	53.542
FB_0486742_L2_PA	fp07_53	7	53.542
FB_0431637_L1_PA	fp07_53	7	53.542
FB_0596787_L5_PA	fp07_53	7	53.542
FB_0053381_L10_PA	fp07_55	7	55.418
FB_0894085_L14_46_1	fp07_56	7	56.664
FB_0560182_L4_PA	fp07_57	7	57.632

FB_0554490_L3_PA	fp07_58	7	58.628
FB_0978671_L4_15_1	fp07_58	7	58.628
FB_0978672_L4_15_1	fp07_58	7	58.628
RB_26215685_L7_PA	fp07_60	7	60.895
FB_0715485_L7_PA	fp07_60	7	60.895
FB_0714965_L7_PA	fp07_60	7	60.895
FB_0715472_L7_PA	fp07_60	7	60.895
FB_0714988_L7_PA	fp07_60	7	60.895
FB_0714957_L7_PA	fp07_60	7	60.895
FB_0714989_L7_PA	fp07_60	7	60.895
FB_0714971_L7_PA	fp07_60	7	60.895
FB_0714990_L7_PA	fp07_60	7	60.895
FB_0714996_L7_PA	fp07_60	7	60.895
FB_0714999_L7_PA	fp07_60	7	60.895
GD_01516_L7_PA	fp07_60	7	60.895
RB_26207129_L7_PA	fp07_60	7	60.895
FB_0715484_L7_PA	fp07_61	7	61.471
FB_0714993_L7_PA	fp07_61	7	61.471
FB_0716011_L7_PA	fp07_61	7	61.471
FB_0716013_L7_PA	fp07_62	7	62.362
FB_0716362_L7_PA	fp07_63	7	63.275
FB_0716365_L7_PA	fp07_63	7	63.275
FB_0716031_L7_PA	fp07_64	7	64.558
FB_0716018_L7_PA	fp07_64	7	64.558
FB_0716019_L7_PA	fp07_64	7	64.558
FB_1019936_L7_48_1	fp07_64	7	64.558
FB_1019948_L7_48_1	fp07_64	7	64.558
FB_0716015_L7_PA	fp07_64	7	64.558
FB_0716351_L7_PA	fp07_65	7	65.806
RB_27987747_L7_PA	fp07_66	7	66.461
RB_27352323_L7_PA	fp07_67	7	67.16
RB_28181089_L7_PA	fp07_67	7	67.16
FB_0717561_L7_PA	fp07_67	7	67.16
RB_27987569_L7_PA	fp07_67	7	67.16
FB_0716027_L7_PA	fp07_67	7	67.16
FB_1021029_L7_48_1	fp07_68	7	68.915
RB_29324859_L7_PA	fp07_69	7	69.424
FB_0718744_L7_PA	fp07_69	7	69.424
FB_0718334_L7_PA	fp07_69	7	69.424
FB_1020547_L7_48_1	fp07_69	7	69.424
FB_0718328_L7_PA	fp07_69	7	69.424
RB_29325151_L7_PA	fp07_69	7	69.424
FB_1021004_L7_48_1	fp07_70	7	70.655
FB_1020991_L7_48_1	fp07_70	7	70.655
FB_0720026_L7_PA	fp07_71	7	71.82
FB_0720027_L7_PA	fp07_71	7	71.82

FB_0720021_L7_PA	fp07_71	7	71.82
FB_0720015_L7_PA	fp07_71	7	71.82
RB_29604961_L7_PA	fp07_71	7	71.82
FB_1021031_L7_48_1	fp07_71	7	71.82
FB_0720018_L7_PA	fp07_71	7	71.82
FB_0720025_L7_PA	fp07_71	7	71.82
FB_1021027_L7_48_1	fp07_71	7	71.82
FB_0720647_L7_PA	fp07_74	7	74.299
FB_0720628_L7_PA	fp07_74	7	74.299
FB_1021295_L7_48_1	fp07_75	7	75.499
FB_0721792_L7_PA	fp07_75	7	75.499
FB_1021197_L7_48_1	fp07_75	7	75.499
FB_0721195_L7_PA	fp07_75	7	75.499
RB_30627770_L7_PA	fp07_75	7	75.499
FB_0721198_L7_PA	fp07_75	7	75.499
FB_0721803_L7_PA	fp07_75	7	75.499
FB_1021193_L7_48_1	fp07_75	7	75.499
FB_0721194_L7_PA	fp07_75	7	75.499
FB_0721780_L7_PA	fp07_75	7	75.499
FB_0721383_L7_PA	fp07_75	7	75.499
FB_0721788_L7_PA	fp07_75	7	75.499
FB_0721207_L7_PA	fp07_75	7	75.499
FB_1021282_L7_48_1	fp07_75	7	75.499
FB_0721793_L7_PA	fp07_75	7	75.499
FB_0721374_L7_PA	fp07_75	7	75.499
FB_0721368_L7_PA	fp07_75	7	75.499
FB_0721778_L7_PA	fp07_75	7	75.499
FB_0721802_L7_PA	fp07_76	7	76.517
FB_1021407_L7_48_1	fp07_76	7	76.517
FB_1021409_L7_48_1	fp07_76	7	76.517
RB_31135974_L7_PA	fp07_76	7	76.517
FB_1021417_L7_48_1	fp07_76	7	76.517
FB_0722034_L7_PA	fp07_76	7	76.517
FB_1021419_L7_48_1	fp07_76	7	76.517
FB_0723110_L8_PA	fp08_00	8	0.339
FB_0722481_L8_PA	fp08_00	8	0.339
FB_0723113_L8_PA	fp08_00	8	0.339
FB_1100217_L8_60_2	fp08_00	8	0.339
RB_250595_L8_PA	fp08_00	8	0.339
FB_0724330_L8_PA	fp08_00	8	0.339
RB_1323911_L8_PA	fp08_01	8	1.187
FB_1100200_L8_60_2	fp08_01	8	1.187
FB_1100193_L8_60_2	fp08_01	8	1.187
FB_1100215_L8_60_2	fp08_01	8	1.187
GD_00175_L8_31_1	fp08_01	8	1.187
FB_0723875_L8_PA	fp08_03	8	3.628

FB_0723882_L8_PA	fp08_03	8	3.628
RB_1316702_L8_PA	fp08_03	8	3.628
FB_1024523_L8_33_1	fp08_03	8	3.628
FB_0725059_L8_PA	fp08_03	8	3.628
FB_1100975_L8_62_2	fp08_03	8	3.628
FB_0729114_L8_PA	fp08_03	8	3.628
FB_0725030_L8_PA	fp08_03	8	3.628
FB_1024534_L8_33_1	fp08_03	8	3.628
FB_1100987_L8_62_2	fp08_03	8	3.628
FB_0729117_L8_PA	fp08_03	8	3.628
FB_1100974_L8_62_2	fp08_03	8	3.628
FB_0724349_L8_PA	fp08_03	8	3.628
FB_1024522_L8_33_1	fp08_03	8	3.628
FB_0724331_L8_PA	fp08_03	8	3.628
FB_0725037_L8_PA	fp08_03	8	3.628
FB_0725047_L8_PA	fp08_03	8	3.628
RB_1632544_L8_PA	fp08_03	8	3.628
GD_00487_L8_PA	fp08_03	8	3.628
RB_1622557_L8_PA	fp08_03	8	3.628
RB_637380_L8_PA	fp08_03	8	3.628
FB_1024518_L8_33_1	fp08_03	8	3.628
FB_0725039_L8_PA	fp08_03	8	3.628
FB_1024537_L8_33_1	fp08_03	8	3.628
FB_0725040_L8_PA	fp08_03	8	3.628
FB_1100970_L8_62_2	fp08_03	8	3.628
FB_0724319_L8_PA	fp08_03	8	3.628
FB_0725061_L8_PA	fp08_03	8	3.628
FB_1024525_L8_33_1	fp08_03	8	3.628
FB_0724335_L8_PA	fp08_03	8	3.628
RB_1286510_L8_PA	fp08_03	8	3.628
FB_1024530_L8_33_1	fp08_03	8	3.628
FB_0725048_L8_PA	fp08_03	8	3.628
FB_0729111_L8_PA	fp08_03	8	3.628
RB_1241072_L8_PA	fp08_03	8	3.628
FB_0723098_L8_PA	fp08_03	8	3.628
RB_626221_L8_PA	fp08_03	8	3.628
FB_1024515_L8_33_1	fp08_03	8	3.628
FB_0723123_L8_PA	fp08_03	8	3.628
FB_1100993_L8_62_2	fp08_04	8	4.48
FB_0725027_L8_PA	fp08_04	8	4.48
RB_1674505_L8_PA	fp08_04	8	4.48
FB_1024519_L8_33_1	fp08_05	8	5.499
FB_1101160_L8_62_2	fp08_05	8	5.499
FB_0729836_L8_PA	fp08_06	8	6.072
FB_1101159_L8_62_2	fp08_06	8	6.072
FB_0729832_L8_PA	fp08_06	8	6.072

FB_1101162_L8_62_2	fp08_06	8	6.072
FB_0729791_L8_PA	fp08_06	8	6.072
FB_1101170_L8_62_2	fp08_06	8	6.072
FB_1101153_L8_62_2	fp08_06	8	6.072
FB_0729828_L8_PA	fp08_06	8	6.072
FB_1101164_L8_62_2	fp08_06	8	6.072
FB_0729799_L8_PA	fp08_06	8	6.072
FB_0729818_L8_PA	fp08_06	8	6.072
FB_1101168_L8_62_2	fp08_06	8	6.072
FB_0729829_L8_PA	fp08_06	8	6.072
GD_01632_L8_32_1	fp08_06	8	6.072
FB_1101154_L8_62_2	fp08_06	8	6.072
FB_0725057_L8_PA	fp08_06	8	6.072
FB_0730634_L8_PA	fp08_09	8	9.663
FB_0730633_L8_PA	fp08_09	8	9.663
FB_1101166_L8_62_2	fp08_09	8	9.663
FB_0730652_L8_PA	fp08_09	8	9.663
FB_0730646_L8_PA	fp08_09	8	9.663
FB_0730638_L8_PA	fp08_09	8	9.663
FB_0730803_L8_PA	fp08_09	8	9.663
FB_1108623_L11_74_3	fp08_09	8	9.663
FB_1108624_L11_74_3	fp08_09	8	9.663
RB_6209800_L8_35_1	fp08_09	8	9.663
FB_1108618_L11_74_3	fp08_09	8	9.663
FB_0730644_L8_PA	fp08_09	8	9.663
FB_1108620_L11_74_3	fp08_09	8	9.663
FB_0730632_L8_PA	fp08_09	8	9.663
FB_0730813_L8_PA	fp08_09	8	9.663
FB_0730812_L8_PA	fp08_09	8	9.663
FB_1108619_L11_74_3	fp08_09	8	9.663
FB_1108638_L11_74_3	fp08_10	8	10.148
FB_0729110_L8_PA	fp08_10	8	10.148
RB_5120857_L8_PA	fp08_11	8	11.162
FB_0729907_L8_PA	fp08_11	8	11.162
FB_0729892_L8_PA	fp08_11	8	11.162
FB_0729911_L8_PA	fp08_11	8	11.162
FB_0734382_L8_PA	fp08_12	8	12.732
FB_0734384_L8_PA	fp08_13	8	13.101
RB_5166014_L8_PA	fp08_13	8	13.101
FB_0735251_L8_PA	fp08_13	8	13.101
RB_8450890_L8_36_1	fp08_14	8	14.07
FB_1026185_L8_36_1	fp08_14	8	14.07
FB_0734369_L8_PA	fp08_14	8	14.07
FB_0734367_L8_PA	fp08_14	8	14.07
FB_0735274_L8_PA	fp08_14	8	14.07
RB_8456044_L8_36_1	fp08_14	8	14.07

FB_0734368_L8_PA	fp08_14	8	14.07
FB_1026184_L8_36_1	fp08_14	8	14.07
FB_1026187_L8_36_1	fp08_14	8	14.07
FB_0735262_L8_PA	fp08_14	8	14.07
GD_01614_L8_PA	fp08_14	8	14.07
FB_0734392_L8_PA	fp08_14	8	14.07
RB_8440063_L8_PA	fp08_14	8	14.07
FB_0734378_L8_PA	fp08_14	8	14.07
FB_0734380_L8_PA	fp08_14	8	14.07
FB_0735264_L8_PA	fp08_14	8	14.07
FB_0735750_L8_PA	fp08_16	8	16.847
FB_1026753_L8_36_1	fp08_17	8	17.201
FB_1026740_L8_36_1	fp08_17	8	17.201
FB_0735727_L8_PA	fp08_17	8	17.201
FB_1026736_L8_36_1	fp08_17	8	17.201
RB_8877553_L8_PA	fp08_17	8	17.201
RB_8904555_L8_PA	fp08_17	8	17.201
FB_0735266_L8_PA	fp08_17	8	17.201
RB_8933286_L8_PA	fp08_17	8	17.201
FB_0735732_L8_PA	fp08_17	8	17.201
RB_8812926_L8_PA	fp08_17	8	17.201
RB_8898659_L8_PA	fp08_17	8	17.201
RB_8972436_L8_PA	fp08_17	8	17.201
FB_0735747_L8_PA	fp08_17	8	17.201
FB_0735731_L8_PA	fp08_17	8	17.201
FB_0874966_L13_34_1	fp08_17	8	17.201
FB_0735728_L8_PA	fp08_18	8	18.375
FB_0738126_L8_PA	fp08_18	8	18.375
FB_0736339_L8_PA	fp08_18	8	18.375
FB_0874952_L13_34_1	fp08_18	8	18.375
FB_0738134_L8_PA	fp08_18	8	18.375
FB_0736353_L8_PA	fp08_18	8	18.375
FB_0874945_L13_34_1	fp08_18	8	18.375
FB_0738156_L8_PA	fp08_18	8	18.375
FB_0736349_L8_PA	fp08_18	8	18.375
FB_0738125_L8_PA	fp08_18	8	18.375
CO_066276_L8_PA	fp08_18	8	18.375
FB_0874963_L13_34_1	fp08_18	8	18.375
GD_00215_L8_36_1	fp08_18	8	18.375
FB_0738137_L8_PA	fp08_18	8	18.375
FB_0874967_L13_34_1	fp08_18	8	18.375
FB_0740378_L8_PA	fp08_18	8	18.375
FB_0788500_L9_PA	fp08_19	8	19.479
RB_10528682_L8_PA	fp08_19	8	19.479
FB_0740377_L8_PA	fp08_19	8	19.479
GD_01132_L8_PA	fp08_20	8	20.771

FB_1105614_L9_41_2	fp08_20	8	20.771
FB_0740419_L8_PA	fp08_20	8	20.771
FB_0740827_L8_PA	fp08_20	8	20.771
FB_0740819_L8_PA	fp08_20	8	20.771
FB_0740404_L8_PA	fp08_20	8	20.771
FB_0740820_L8_PA	fp08_20	8	20.771
FB_0788498_L9_PA	fp08_20	8	20.771
FB_0788496_L9_PA	fp08_20	8	20.771
FB_0740826_L8_PA	fp08_20	8	20.771
RB_13348417_L8_PA	fp08_20	8	20.771
FB_1044100_L9_22_1	fp08_20	8	20.771
RB_12931783_L8_PA	fp08_20	8	20.771
FB_0740417_L8_PA	fp08_20	8	20.771
FB_0740383_L8_PA	fp08_20	8	20.771
FB_1105616_L9_41_2	fp08_20	8	20.771
FB_0740918_L8_PA	fp08_20	8	20.771
FB_0740382_L8_PA	fp08_20	8	20.771
FB_1044081_L9_22_1	fp08_20	8	20.771
FB_0740409_L8_PA	fp08_20	8	20.771
FB_0740906_L8_PA	fp08_20	8	20.771
FB_0740392_L8_PA	fp08_20	8	20.771
FB_0740916_L8_PA	fp08_20	8	20.771
FB_1044078_L9_22_1	fp08_21	8	21.566
FB_0788487_L9_PA	fp08_21	8	21.566
RB_12919113_L8_PA	fp08_21	8	21.566
RB_14278506_L8_PA	fp08_21	8	21.566
RB_14295970_L8_PA	fp08_21	8	21.566
FB_0740921_L8_PA	fp08_21	8	21.566
FB_1044079_L9_22_1	fp08_21	8	21.566
FB_0742415_L8_PA	fp08_21	8	21.566
RB_14298908_L8_PA	fp08_21	8	21.566
FB_0741257_L8_PA	fp08_21	8	21.566
GD_00584_L8_41_1	fp08_21	8	21.566
RB_14333819_L8_68_2	fp08_21	8	21.566
FB_0741258_L8_PA	fp08_21	8	21.566
FB_0741256_L8_PA	fp08_22	8	22.688
RB_14572969_L8_PA	fp08_22	8	22.688
GD_00639_L8_PA	fp08_22	8	22.688
FB_1044084_L9_22_1	fp08_22	8	22.688
RB_15006641_L8_PA	fp08_22	8	22.688
FB_0742401_L8_PA	fp08_22	8	22.688
FB_1028551_L8_41_1	fp08_22	8	22.688
FB_0742408_L8_PA	fp08_22	8	22.688
FB_1028553_L8_41_1	fp08_22	8	22.688
FB_0742418_L8_PA	fp08_23	8	23.175
FB_0742405_L8_PA	fp08_23	8	23.175

FB_0742409_L8_PA	fp08_23	8	23.175
RB_15251985_L8_PA	fp08_24	8	24.673
FB_1028552_L8_41_1	fp08_24	8	24.673
FB_0743093_L8_PA	fp08_25	8	25.546
FB_0743100_L8_PA	fp08_25	8	25.546
FB_0743112_L8_PA	fp08_25	8	25.546
FB_0743091_L8_PA	fp08_25	8	25.546
GD_00354_L8_PA	fp08_25	8	25.546
RB_15259509_L8_PA	fp08_25	8	25.546
FB_0743545_L8_PA	fp08_25	8	25.546
FB_0743546_L8_PA	fp08_25	8	25.546
FB_1029634_L8_41_1	fp08_27	8	27.45
FB_1102511_L8_68_2	fp08_27	8	27.45
FB_0741253_L8_PA	fp08_27	8	27.45
FB_0706561_L7_PA	fp08_27	8	27.45
GD_00086_L8_41_1	fp08_27	8	27.45
FB_0743572_L8_PA	fp08_27	8	27.45
FB_0264291_L15_PA	fp08_27	8	27.45
FB_1102514_L8_68_2	fp08_27	8	27.45
FB_0743551_L8_PA	fp08_27	8	27.45
FB_0706576_L7_PA	fp08_27	8	27.45
FB_1102515_L8_68_2	fp08_27	8	27.45
FB_0264285_L15_PA	fp08_27	8	27.45
FB_1029057_L8_41_1	fp08_27	8	27.45
FB_0743544_L8_PA	fp08_27	8	27.45
FB_1102492_L8_68_2	fp08_27	8	27.45
FB_0743552_L8_PA	fp08_27	8	27.45
FB_0264289_L15_PA	fp08_27	8	27.45
FB_0743564_L8_PA	fp08_27	8	27.45
FB_1029606_L8_41_1	fp08_27	8	27.45
FB_0706554_L7_PA	fp08_27	8	27.45
FB_0743543_L8_PA	fp08_27	8	27.45
FB_1029063_L8_41_1	fp08_27	8	27.45
FB_1029098_L8_41_1	fp08_27	8	27.45
FB_0743547_L8_PA	fp08_27	8	27.45
FB_0706569_L7_PA	fp08_27	8	27.45
FB_0706553_L7_PA	fp08_27	8	27.45
FB_1029100_L8_41_1	fp08_27	8	27.45
RB_16591000_L8_PA	fp08_27	8	27.45
RB_16577884_L8_PA	fp08_27	8	27.45
RB_16535826_L8_PA	fp08_27	8	27.45
FB_0744784_L8_PA	fp08_28	8	28.205
FB_0745842_L8_PA	fp08_28	8	28.205
FB_0744817_L8_PA	fp08_28	8	28.205
FB_0744815_L8_PA	fp08_28	8	28.205
FB_0745799_L8_PA	fp08_28	8	28.205

FB_0744821_L8_PA	fp08_28	8	28.205
FB_1117727_L8_88_3	fp08_28	8	28.205
FB_0744794_L8_PA	fp08_28	8	28.205
FB_1117728_L8_88_3	fp08_28	8	28.205
FB_0706579_L7_PA	fp08_28	8	28.205
FB_0744779_L8_PA	fp08_28	8	28.205
FB_0744781_L8_PA	fp08_28	8	28.205
FB_1117736_L8_88_3	fp08_28	8	28.205
FB_0264294_L15_PA	fp08_28	8	28.205
FB_0706570_L7_PA	fp08_28	8	28.205
FB_0746410_L8_PA	fp08_28	8	28.205
FB_0744785_L8_PA	fp08_28	8	28.205
FB_0745803_L8_PA	fp08_28	8	28.205
FB_0745335_L8_PA	fp08_28	8	28.205
FB_0745840_L8_PA	fp08_29	8	29.282
FB_0745823_L8_PA	fp08_29	8	29.282
FB_0745327_L8_PA	fp08_29	8	29.282
FB_1029897_L8_41_1	fp08_29	8	29.282
FB_0746385_L8_PA	fp08_29	8	29.282
FB_0745329_L8_PA	fp08_29	8	29.282
FB_0745330_L8_PA	fp08_29	8	29.282
FB_0745325_L8_PA	fp08_29	8	29.282
FB_0745334_L8_PA	fp08_29	8	29.282
FB_0746401_L8_PA	fp08_29	8	29.282
FB_0746380_L8_PA	fp08_29	8	29.282
RB_16583813_L8_PA	fp08_29	8	29.282
FB_1029875_L8_41_1	fp08_29	8	29.282
FB_0748467_L8_PA	fp08_31	8	31.648
RB_17361806_L8_PA	fp08_31	8	31.648
FB_1030858_L8_41_1	fp08_31	8	31.648
FB_1030993_L8_41_1	fp08_31	8	31.648
FB_1030862_L8_41_1	fp08_31	8	31.648
FB_0748453_L8_PA	fp08_31	8	31.648
RB_17343145_L8_PA	fp08_31	8	31.648
FB_0747614_L8_PA	fp08_31	8	31.648
FB_0748452_L8_PA	fp08_31	8	31.648
FB_0747616_L8_PA	fp08_31	8	31.648
FB_0748469_L8_PA	fp08_31	8	31.648
RB_17392486_L8_PA	fp08_31	8	31.648
FB_0748463_L8_PA	fp08_31	8	31.648
FB_0748462_L8_PA	fp08_31	8	31.648
FB_0748449_L8_PA	fp08_31	8	31.648
FB_0209687_L13_PA	fp08_31	8	31.648
FB_1030994_L8_41_1	fp08_31	8	31.648
FB_1030867_L8_41_1	fp08_31	8	31.648
FB_0747653_L8_PA	fp08_31	8	31.648

FB_0748455_L8_PA	fp08_31	8	31.648
FB_0747629_L8_PA	fp08_31	8	31.648
FB_0127286_L12_PA	fp08_33	8	33.454
FB_0750355_L8_PA	fp08_33	8	33.454
FB_0749502_L8_PA	fp08_33	8	33.454
FB_0749500_L8_PA	fp08_33	8	33.454
FB_0749518_L8_PA	fp08_33	8	33.454
FB_0750341_L8_PA	fp08_33	8	33.454
FB_0749655_L8_PA	fp08_33	8	33.454
FB_0749637_L8_PA	fp08_33	8	33.454
FB_0750349_L8_PA	fp08_33	8	33.454
FB_0750960_L8_PA	fp08_33	8	33.454
FB_0749498_L8_PA	fp08_33	8	33.454
FB_0749638_L8_PA	fp08_33	8	33.454
FB_0749496_L8_PA	fp08_33	8	33.454
FB_0749632_L8_PA	fp08_33	8	33.454
FB_0749652_L8_PA	fp08_33	8	33.454
FB_0749569_L8_PA	fp08_33	8	33.454
FB_0750344_L8_PA	fp08_33	8	33.454
FB_1031319_L8_42_1	fp08_33	8	33.454
FB_0749641_L8_PA	fp08_33	8	33.454
RB_18192194_L8_41_1	fp08_33	8	33.454
FB_0749506_L8_PA	fp08_33	8	33.454
FB_0750348_L8_PA	fp08_33	8	33.454
FB_0749634_L8_PA	fp08_33	8	33.454
FB_0749508_L8_PA	fp08_33	8	33.454
GD_01768_L8_PA	fp08_33	8	33.454
FB_0750329_L8_PA	fp08_33	8	33.454
FB_0747655_L8_PA	fp08_33	8	33.454
FB_0126727_L12_PA	fp08_34	8	34.455
FB_1032072_L8_47_1	fp08_34	8	34.455
FB_0753459_L8_PA	fp08_34	8	34.455
FB_0132996_L12_PA	fp08_34	8	34.455
GD_02685_L8_PA	fp08_35	8	35.863
FB_0754252_L8_PA	fp08_35	8	35.863
FB_0410059_L1_PA	fp08_35	8	35.863
FB_0751258_L8_PA	fp08_35	8	35.863
FB_0751257_L8_PA	fp08_35	8	35.863
FB_0750956_L8_PA	fp08_35	8	35.863
FB_0750959_L8_PA	fp08_35	8	35.863
FB_0750940_L8_PA	fp08_35	8	35.863
RB_19657174_L8_PA	fp08_35	8	35.863
FB_0750970_L8_PA	fp08_35	8	35.863
FB_0750951_L8_PA	fp08_35	8	35.863
GD_02689_L8_70_2	fp08_35	8	35.863
RB_19621603_L8_PA	fp08_35	8	35.863

FB_0750944_L8_PA	fp08_35	8	35.863
RB_18181838_L8_PA	fp08_36	8	36.657
GD_00862_L8_43_1	fp08_36	8	36.657
FB_0126741_L12_PA	fp08_36	8	36.657
FB_1069911_L15_102_2	fp08_36	8	36.657
FB_1069903_L15_102_2	fp08_36	8	36.657
FB_0309303_L15_PA	fp08_36	8	36.657
RB_23713008_L8_PA	fp08_38	8	38.376
FB_0127300_L12_PA	fp08_38	8	38.376
FB_0126740_L12_PA	fp08_38	8	38.376
FB_0751268_L8_PA	fp08_38	8	38.376
FB_0126721_L12_PA	fp08_38	8	38.376
FB_0126718_L12_PA	fp08_38	8	38.376
FB_0752322_L8_PA	fp08_38	8	38.376
FB_0126704_L12_PA	fp08_38	8	38.376
RB_20088635_L8_PA	fp08_38	8	38.376
FB_0127319_L12_PA	fp08_38	8	38.376
FB_0752321_L8_PA	fp08_38	8	38.376
GD_00311_L8_PA	fp08_38	8	38.376
FB_0752320_L8_PA	fp08_38	8	38.376
FB_0752328_L8_PA	fp08_38	8	38.376
FB_0127297_L12_PA	fp08_38	8	38.376
FB_0755026_L8_PA	fp08_40	8	40.219
FB_0755028_L8_PA	fp08_40	8	40.219
FB_0755020_L8_PA	fp08_40	8	40.219
GD_00157_L8_PA	fp08_41	8	41.408
FB_0753429_L8_PA	fp08_41	8	41.408
FB_0132990_L12_PA	fp08_41	8	41.408
FB_0932949_L17_16_1	fp08_41	8	41.408
FB_0755032_L8_PA	fp08_41	8	41.408
FB_0755095_L8_PA	fp08_41	8	41.408
FB_0754070_L8_PA	fp08_41	8	41.408
FB_0753471_L8_PA	fp08_41	8	41.408
FB_0755089_L8_PA	fp08_41	8	41.408
RB_21571716_L8_PA	fp08_41	8	41.408
RB_23464386_L8_PA	fp08_41	8	41.408
RB_22219984_L8_PA	fp08_41	8	41.408
GD_01479_L8_PA	fp08_41	8	41.408
FB_0756245_L8_PA	fp08_41	8	41.408
FB_1032282_L8_47_1	fp08_41	8	41.408
FB_0753427_L8_PA	fp08_41	8	41.408
RB_23752885_L8_PA	fp08_41	8	41.408
FB_0754072_L8_PA	fp08_41	8	41.408
FB_1032284_L8_47_1	fp08_41	8	41.408
RB_22201754_L8_PA	fp08_41	8	41.408
FB_0754071_L8_PA	fp08_41	8	41.408

FB_0753434_L8_PA	fp08_41	8	41.408
RB_25179171_L8_PA	fp08_41	8	41.408
RB_22558109_L8_PA	fp08_41	8	41.408
FB_0755022_L8_PA	fp08_41	8	41.408
FB_0132994_L12_PA	fp08_41	8	41.408
RB_21581037_L8_72_2	fp08_41	8	41.408
FB_0756228_L8_PA	fp08_41	8	41.408
RB_22527637_L8_PA	fp08_41	8	41.408
FB_1102997_L8_70_2	fp08_41	8	41.408
FB_0755090_L8_PA	fp08_41	8	41.408
FB_0754066_L8_PA	fp08_41	8	41.408
RB_23899578_L8_PA	fp08_41	8	41.408
FB_0754658_L8_PA	fp08_41	8	41.408
FB_0754661_L8_PA	fp08_41	8	41.408
GD_00542_L8_PA	fp08_41	8	41.408
FB_0754059_L8_PA	fp08_41	8	41.408
FB_0755823_L8_PA	fp08_42	8	42.322
FB_0410066_L1_PA	fp08_42	8	42.322
MdPI_L8_PA	fp08_42	8	42.322
FB_0409971_L1_PA	fp08_42	8	42.322
FB_0410049_L1_PA	fp08_42	8	42.322
FB_0938643_L1_36_1	fp08_42	8	42.322
FB_0409966_L1_PA	fp08_42	8	42.322
FB_0755822_L8_PA	fp08_42	8	42.322
FB_0755427_L8_PA	fp08_42	8	42.322
FB_0755412_L8_PA	fp08_42	8	42.322
FB_0756785_L8_PA	fp08_42	8	42.322
FB_0756772_L8_PA	fp08_42	8	42.322
FB_0756792_L8_PA	fp08_42	8	42.322
FB_1033129_L8_52_1	fp08_42	8	42.322
FB_0757617_L8_PA	fp08_42	8	42.322
FB_0142008_L12_PA	fp08_43	8	43.711
FB_0756774_L8_PA	fp08_43	8	43.711
FB_0755418_L8_PA	fp08_43	8	43.711
FB_0076254_L11_PA	fp08_43	8	43.711
FB_0142031_L12_PA	fp08_43	8	43.711
FB_0076260_L11_PA	fp08_43	8	43.711
FB_0142007_L12_PA	fp08_43	8	43.711
FB_0076279_L11_PA	fp08_43	8	43.711
FB_1018770_L7_44_1	fp08_45	8	45.031
FB_0712038_L7_PA	fp08_45	8	45.031
FB_0712034_L7_PA	fp08_45	8	45.031
FB_1033118_L8_52_1	fp08_45	8	45.031
FB_0712058_L7_PA	fp08_45	8	45.031
FB_1018471_L7_44_1	fp08_45	8	45.031
FB_0757609_L8_PA	fp08_45	8	45.031

FB_0758793_L8_PA	fp08_45	8	45.031
FB_1033131_L8_52_1	fp08_45	8	45.031
GD_01148_L8_52_1	fp08_45	8	45.031
FB_0151798_L12_PA	fp08_45	8	45.031
FB_1033087_L8_52_1	fp08_45	8	45.031
FB_1033086_L8_52_1	fp08_45	8	45.031
FB_0712047_L7_PA	fp08_45	8	45.031
FB_1033089_L8_52_1	fp08_45	8	45.031
FB_0758061_L8_PA	fp08_46	8	46.126
RB_27610478_L8_PA	fp08_46	8	46.126
FB_0891072_L14_41_1	fp08_46	8	46.126
FB_0758799_L8_PA	fp08_46	8	46.126
RB_27300439_L8_PA	fp08_47	8	47.491
FB_0758052_L8_PA	fp08_47	8	47.491
FB_0758071_L8_PA	fp08_47	8	47.491
FB_0758056_L8_PA	fp08_47	8	47.491
FB_0758049_L8_PA	fp08_47	8	47.491
RB_27300417_L8_PA	fp08_47	8	47.491
FB_0758058_L8_PA	fp08_47	8	47.491
FB_0758063_L8_PA	fp08_47	8	47.491
FB_0758068_L8_PA	fp08_47	8	47.491
FB_0192616_L13_PA	fp08_47	8	47.491
FB_0192587_L13_PA	fp08_48	8	48.515
RB_28160723_L8_PA	fp08_48	8	48.515
FB_0891086_L14_41_1	fp08_48	8	48.515
FB_0758858_L8_PA	fp08_48	8	48.515
FB_0891076_L14_41_1	fp08_48	8	48.515
FB_0891082_L14_41_1	fp08_48	8	48.515
RB_28172464_L8_PA	fp08_48	8	48.515
RB_28158121_L8_PA	fp08_48	8	48.515
RB_28185282_L8_PA	fp08_49	8	49.823
FB_0047635_L10_PA	fp08_50	8	50.372
FB_0877881_L13_40_1	fp08_50	8	50.372
RB_28263232_L8_PA	fp08_50	8	50.372
RB_28265359_L8_PA	fp08_50	8	50.372
FB_0192609_L13_PA	fp08_51	8	51.696
FB_0759167_L8_PA	fp08_52	8	52.908
FB_1096139_L5_79_2	fp08_52	8	52.908
FB_0092786_L11_PA	fp08_52	8	52.908
FB_0638996_L5_PA	fp08_52	8	52.908
FB_0759165_L8_PA	fp08_52	8	52.908
FB_1096133_L5_79_2	fp08_52	8	52.908
FB_0759174_L8_PA	fp08_52	8	52.908
FB_1033703_L8_55_1	fp08_52	8	52.908
FB_0092789_L11_PA	fp08_52	8	52.908
FB_0047657_L10_PA	fp08_52	8	52.908

FB_0047644_L10_PA	fp08_52	8	52.908
FB_0638992_L5_PA	fp08_52	8	52.908
FB_0759171_L8_PA	fp08_52	8	52.908
FB_0759435_L8_PA	fp08_52	8	52.908
FB_0047646_L10_PA	fp08_52	8	52.908
FB_0092800_L11_PA	fp08_52	8	52.908
FB_0971469_L3_48_1	fp08_52	8	52.908
FB_0093655_L11_PA	fp08_52	8	52.908
FB_0759176_L8_PA	fp08_52	8	52.908
FB_0759430_L8_PA	fp08_52	8	52.908
RB_28804875_L8_PA	fp08_52	8	52.908
FB_0759432_L8_PA	fp08_52	8	52.908
FB_0092788_L11_PA	fp08_52	8	52.908
FB_0093686_L11_PA	fp08_52	8	52.908
FB_0093670_L11_PA	fp08_53	8	53.513
FB_0971487_L3_48_1	fp08_53	8	53.513
FB_0971471_L3_48_1	fp08_53	8	53.513
FB_0760677_L8_PA	fp08_54	8	54.577
FB_0760678_L8_PA	fp08_54	8	54.577
RB_29562018_L8_PA	fp08_54	8	54.577
FB_0760676_L8_PA	fp08_54	8	54.577
FB_0760671_L8_PA	fp08_54	8	54.577
GD_02575_L8_PA	fp08_54	8	54.577
RB_30420390_L8_PA	fp08_54	8	54.577
RB_29503558_L8_55_1	fp08_54	8	54.577
RB_30479542_L8_55_1	fp08_54	8	54.577
FB_0760681_L8_PA	fp08_54	8	54.577
FB_0760679_L8_PA	fp08_54	8	54.577
FB_1103591_L8_79_2	fp08_54	8	54.577
FB_0762874_L8_PA	fp08_54	8	54.577
GD_01764_L8_PA	fp08_54	8	54.577
FB_0764491_L8_PA	fp08_54	8	54.577
FB_0762079_L8_PA	fp08_54	8	54.577
FB_0764495_L8_PA	fp08_54	8	54.577
FB_0762848_L8_PA	fp08_54	8	54.577
FB_0761422_L8_PA	fp08_54	8	54.577
GD_00342_L8_PA	fp08_54	8	54.577
FB_0763380_L8_PA	fp08_54	8	54.577
FB_0761401_L8_PA	fp08_54	8	54.577
FB_0761424_L8_PA	fp08_54	8	54.577
FB_0764494_L8_PA	fp08_54	8	54.577
FB_0763361_L8_PA	fp08_54	8	54.577
FB_0762849_L8_PA	fp08_54	8	54.577
FB_0761418_L8_PA	fp08_54	8	54.577
FB_1034615_L8_55_1	fp08_54	8	54.577
FB_0762852_L8_PA	fp08_54	8	54.577

FB_1034394_L8_55_1	fp08_54	8	54.577
FB_0763354_L8_PA	fp08_54	8	54.577
FB_0763381_L8_PA	fp08_54	8	54.577
FB_0762861_L8_PA	fp08_54	8	54.577
FB_0762087_L8_PA	fp08_54	8	54.577
FB_0762844_L8_PA	fp08_54	8	54.577
FB_0764489_L8_PA	fp08_54	8	54.577
RB_30403968_L8_PA	fp08_54	8	54.577
FB_1033691_L8_55_1	fp08_54	8	54.577
FB_1034396_L8_55_1	fp08_55	8	55.281
FB_1034389_L8_55_1	fp08_55	8	55.281
RB_31145617_L8_PA	fp08_55	8	55.281
FB_0761428_L8_PA	fp08_55	8	55.281
FB_1035415_L8_57_1	fp08_55	8	55.281
FB_0762865_L8_PA	fp08_55	8	55.281
FB_1103570_L8_79_2	fp08_55	8	55.281
FB_0763359_L8_PA	fp08_55	8	55.281
FB_0764505_L8_PA	fp08_55	8	55.281
GD_01576_L8_PA	fp08_55	8	55.281
FB_0764501_L8_PA	fp08_55	8	55.281
FB_1103569_L8_79_2	fp08_55	8	55.281
FB_0763357_L8_PA	fp08_55	8	55.281
FB_0764512_L8_PA	fp08_55	8	55.281
FB_0763370_L8_PA	fp08_55	8	55.281
FB_0763382_L8_PA	fp08_55	8	55.281
GD_00293_L8_PA	fp08_55	8	55.281
FB_0764492_L8_PA	fp08_55	8	55.281
FB_1103580_L8_79_2	fp08_55	8	55.281
GD_00975_L8_PA	fp08_55	8	55.281
FB_0763387_L8_PA	fp08_55	8	55.281
FB_0763150_L8_PA	fp08_55	8	55.281
FB_0764526_L8_PA	fp08_55	8	55.281
FB_0763372_L8_PA	fp08_55	8	55.281
RB_31137343_L8_PA	fp08_55	8	55.281
FB_0762850_L8_PA	fp08_56	8	56.979
FB_0766785_L8_PA	fp08_57	8	57.749
FB_1053497_L10_62_2	fp08_57	8	57.749
RB_32814468_L8_PA	fp08_57	8	57.749
FB_0765573_L8_PA	fp08_57	8	57.749
RB_32812193_L8_PA	fp08_57	8	57.749
FB_0765972_L8_PA	fp08_57	8	57.749
FB_0461094_L2_PA	fp08_57	8	57.749
FB_0765947_L8_PA	fp08_57	8	57.749
FB_0461096_L2_PA	fp08_57	8	57.749
FB_0765594_L8_PA	fp08_57	8	57.749
FB_0461119_L2_PA	fp08_57	8	57.749

FB_0461110_L2_PA	fp08_57	8	57.749
FB_0811039_L9_PA	fp08_57	8	57.749
FB_0811060_L9_PA	fp08_58	8	58.948
FB_0811059_L9_PA	fp08_58	8	58.948
FB_0766783_L8_PA	fp08_58	8	58.948
FB_0811040_L9_PA	fp08_58	8	58.948
FB_0811057_L9_PA	fp08_58	8	58.948
GD_01905_L8_PA	fp08_58	8	58.948
FB_0811056_L9_PA	fp08_58	8	58.948
FB_0461097_L2_PA	fp08_59	8	59.643
FB_1104008_L8_82_2	fp08_60	8	60.148
FB_1036769_L8_59_1	fp08_60	8	60.148
FB_0767171_L8_PA	fp08_60	8	60.148
FB_0767509_L8_PA	fp08_60	8	60.148
FB_1037272_L8_59_1	fp08_60	8	60.148
FB_0767502_L8_PA	fp08_60	8	60.148
FB_0767496_L8_PA	fp08_60	8	60.148
FB_0767507_L8_PA	fp08_60	8	60.148
FB_1104007_L8_82_2	fp08_60	8	60.148
FB_0767485_L8_PA	fp08_60	8	60.148
FB_1037262_L8_59_1	fp08_60	8	60.148
FB_0767500_L8_PA	fp08_60	8	60.148
FB_0767173_L8_PA	fp08_60	8	60.148
FB_1037246_L8_59_1	fp08_60	8	60.148
FB_0767501_L8_PA	fp08_60	8	60.148
GD_00246_L8_59_1	fp08_60	8	60.148
FB_0767172_L8_PA	fp08_60	8	60.148
FB_0461117_L2_PA	fp08_60	8	60.148
FB_1037247_L8_59_1	fp08_61	8	61.691
FB_0768978_L8_PA	fp08_61	8	61.691
FB_0768980_L8_PA	fp08_65	8	65.157
FB_0768985_L8_PA	fp08_65	8	65.157
FB_0769033_L8_PA	fp08_65	8	65.157
FB_0768984_L8_PA	fp08_65	8	65.157
FB_0768982_L8_PA	fp08_65	8	65.157
FB_0769005_L8_PA	fp08_65	8	65.157
FB_0769013_L8_PA	fp08_65	8	65.157
FB_0769030_L8_PA	fp08_65	8	65.157
FB_0768989_L8_PA	fp08_65	8	65.157
GD_00463_L9_PA	fp09_00	9	0.104
FB_0770567_L9_PA	fp09_00	9	0.104
FB_0770539_L9_PA	fp09_00	9	0.104
RB_506758_L9_PA	fp09_00	9	0.104
FB_0770538_L9_PA	fp09_00	9	0.104
RB_498570_L9_PA	fp09_00	9	0.104
RB_465054_L9_PA	fp09_00	9	0.104

FB_0770548_L9_PA	fp09_00	9	0.104
FB_0770560_L9_PA	fp09_00	9	0.104
FB_0770557_L9_PA	fp09_00	9	0.104
FB_0770554_L9_PA	fp09_00	9	0.104
FB_0770542_L9_PA	fp09_00	9	0.104
RB_443008_L9_PA	fp09_00	9	0.104
FB_0770537_L9_PA	fp09_00	9	0.104
RB_558868_L9_PA	fp09_00	9	0.104
RB_516728_L9_PA	fp09_00	9	0.104
FB_0771997_L9_PA	fp09_00	9	0.104
RB_561080_L9_PA	fp09_00	9	0.104
FB_0771341_L9_PA	fp09_01	9	1.467
RB_489635_L9_PA	fp09_01	9	1.467
FB_0771340_L9_PA	fp09_02	9	2.841
FB_0771987_L9_PA	fp09_03	9	3.645
FB_0772423_L9_PA	fp09_03	9	3.645
FB_0771342_L9_PA	fp09_03	9	3.645
RB_1433030_L9_20_1	fp09_03	9	3.645
RB_1276715_L9_PA	fp09_03	9	3.645
GD_01328_L9_39_2	fp09_03	9	3.645
RB_1733945_L9_39_2	fp09_03	9	3.645
FB_0771981_L9_PA	fp09_03	9	3.645
FB_1104594_L9_39_2	fp09_03	9	3.645
FB_0771982_L9_PA	fp09_03	9	3.645
RB_1273803_L9_PA	fp09_03	9	3.645
FB_0772424_L9_PA	fp09_03	9	3.645
FB_0772419_L9_PA	fp09_04	9	4.391
RB_995048_L9_PA	fp09_04	9	4.391
RB_1005241_L9_PA	fp09_04	9	4.391
GD_00169_L9_PA	fp09_05	9	5.52
FB_0773440_L9_PA	fp09_05	9	5.52
FB_0774498_L9_PA	fp09_05	9	5.52
FB_0771983_L9_PA	fp09_06	9	6.692
RB_1743002_L9_PA	fp09_06	9	6.692
FB_0773433_L9_PA	fp09_06	9	6.692
FB_1104599_L9_39_2	fp09_06	9	6.692
RB_1790459_L9_20_1	fp09_06	9	6.692
RB_1782044_L9_20_1	fp09_06	9	6.692
FB_0773415_L9_PA	fp09_06	9	6.692
FB_0773791_L9_PA	fp09_07	9	7.361
FB_0773441_L9_PA	fp09_07	9	7.361
FB_0773425_L9_PA	fp09_07	9	7.361
FB_0773418_L9_PA	fp09_07	9	7.361
FB_0773420_L9_PA	fp09_07	9	7.361
FB_0773445_L9_PA	fp09_07	9	7.361
FB_0773424_L9_PA	fp09_07	9	7.361

RB_1312317_L9_PA	fp09_07	9	7.361
RB_1502793_L9_PA	fp09_07	9	7.361
RB_1287577_L9_PA	fp09_07	9	7.361
RB_1765225_L9_PA	fp09_07	9	7.361
FB_1104657_L9_39_2	fp09_07	9	7.361
FB_1104663_L9_39_2	fp09_08	9	8.462
FB_0421231_L1_PA	fp09_08	9	8.462
FB_0773783_L9_PA	fp09_08	9	8.462
FB_0773779_L9_PA	fp09_08	9	8.462
GD_01573_L9_PA	fp09_08	9	8.462
FB_0773770_L9_PA	fp09_08	9	8.462
RB_1969453_L9_20_1	fp09_08	9	8.462
FB_0773781_L9_PA	fp09_08	9	8.462
FB_1104642_L9_39_2	fp09_08	9	8.462
FB_0774500_L9_PA	fp09_09	9	9.295
RB_2249956_L9_PA	fp09_10	9	10.799
FB_1104651_L9_39_2	fp09_10	9	10.799
RB_1993488_L9_PA	fp09_10	9	10.799
RB_2386872_L9_PA	fp09_10	9	10.799
FB_0775868_L9_PA	fp09_10	9	10.799
FB_0775855_L9_PA	fp09_10	9	10.799
FB_0774491_L9_PA	fp09_10	9	10.799
FB_0775850_L9_PA	fp09_10	9	10.799
FB_0775866_L9_PA	fp09_10	9	10.799
GD_00679_L9_PA	fp09_10	9	10.799
FB_0775854_L9_PA	fp09_10	9	10.799
FB_0774492_L9_PA	fp09_10	9	10.799
FB_1104650_L9_39_2	fp09_10	9	10.799
FB_0775869_L9_PA	fp09_10	9	10.799
GD_02134_L9_PA	fp09_10	9	10.799
RB_2397565_L9_PA	fp09_11	9	11.77
RB_3023374_L9_PA	fp09_11	9	11.77
RB_3028463_L9_PA	fp09_11	9	11.77
RB_3751782_L9_39_2	fp09_11	9	11.77
FB_1104730_L9_39_2	fp09_11	9	11.77
RB_3543417_L9_PA	fp09_11	9	11.77
FB_0775874_L9_PA	fp09_11	9	11.77
FB_1104735_L9_39_2	fp09_11	9	11.77
RB_3064284_L9_PA	fp09_11	9	11.77
FB_0776760_L9_PA	fp09_11	9	11.77
FB_0776380_L9_PA	fp09_11	9	11.77
FB_1104729_L9_39_2	fp09_12	9	12.422
FB_0776373_L9_PA	fp09_12	9	12.422
FB_0776378_L9_PA	fp09_12	9	12.422
FB_0776382_L9_PA	fp09_12	9	12.422
FB_1104725_L9_39_2	fp09_12	9	12.422

FB_0776767_L9_PA	fp09_12	9	12.422
FB_0776771_L9_PA	fp09_12	9	12.422
FB_0776761_L9_PA	fp09_12	9	12.422
FB_0776744_L9_PA	fp09_12	9	12.422
FB_1104736_L9_39_2	fp09_12	9	12.422
FB_0776750_L9_PA	fp09_12	9	12.422
FB_0776387_L9_PA	fp09_12	9	12.422
RB_3771454_L9_PA	fp09_12	9	12.422
FB_0777203_L9_PA	fp09_12	9	12.422
FB_0777173_L9_PA	fp09_12	9	12.422
FB_0777176_L9_PA	fp09_12	9	12.422
GD_01524_L9_PA	fp09_12	9	12.422
RB_3797355_L9_PA	fp09_12	9	12.422
RB_3788429_L9_PA	fp09_12	9	12.422
FB_1104803_L9_39_2	fp09_12	9	12.422
RB_3783110_L9_PA	fp09_13	9	13.573
FB_0775847_L9_PA	fp09_13	9	13.573
FB_0776766_L9_PA	fp09_13	9	13.573
FB_0778946_L9_PA	fp09_14	9	14.706
FB_0778924_L9_PA	fp09_14	9	14.706
FB_0778934_L9_PA	fp09_14	9	14.706
RB_5387010_L9_PA	fp09_14	9	14.706
FB_0778927_L9_PA	fp09_14	9	14.706
RB_4461663_L9_PA	fp09_14	9	14.706
RB_4685551_L9_PA	fp09_14	9	14.706
FB_0778216_L9_PA	fp09_15	9	15.293
FB_0778218_L9_PA	fp09_15	9	15.293
FB_1104962_L9_39_2	fp09_15	9	15.293
RB_4699317_L9_PA	fp09_16	9	16.433
RB_5399131_L9_PA	fp09_16	9	16.433
FB_0780721_L9_PA	fp09_16	9	16.433
FB_1104961_L9_39_2	fp09_17	9	17.604
FB_0905872_L15_49_1	fp09_17	9	17.604
FB_0778926_L9_PA	fp09_17	9	17.604
FB_0780096_L9_PA	fp09_17	9	17.604
FB_0992822_L5_38_1	fp09_17	9	17.604
GD_00514_L9_PA	fp09_19	9	19.764
FB_1105048_L9_39_2	fp09_19	9	19.764
FB_0780730_L9_PA	fp09_19	9	19.764
FB_0992839_L5_38_1	fp09_19	9	19.764
FB_1105039_L9_39_2	fp09_20	9	20.297
FB_1105049_L9_39_2	fp09_20	9	20.297
FB_1105046_L9_39_2	fp09_20	9	20.297
RB_5973848_L9_PA	fp09_20	9	20.297
FB_0781283_L9_PA	fp09_20	9	20.297
RB_5964010_L9_PA	fp09_20	9	20.297

FB_0067160_L11_PA	fp09_20	9	20.297
FB_0780098_L9_PA	fp09_20	9	20.297
RB_4435852_L9_PA	fp09_20	9	20.297
FB_0781287_L9_PA	fp09_21	9	21.811
FB_0781834_L9_PA	fp09_21	9	21.811
FB_0781827_L9_PA	fp09_21	9	21.811
FB_0781818_L9_PA	fp09_21	9	21.811
FB_0781808_L9_PA	fp09_21	9	21.811
FB_0992834_L5_38_1	fp09_21	9	21.811
FB_0781810_L9_PA	fp09_21	9	21.811
FB_0781806_L9_PA	fp09_21	9	21.811
FB_0992840_L5_38_1	fp09_21	9	21.811
RB_6402288_L9_PA	fp09_22	9	22.348
FB_0992843_L5_38_1	fp09_22	9	22.348
RB_5981805_L9_PA	fp09_22	9	22.348
RB_6288877_L9_PA	fp09_22	9	22.348
FB_0782159_L9_PA	fp09_22	9	22.348
FB_0782149_L9_PA	fp09_23	9	23.679
FB_0782143_L9_PA	fp09_24	9	24.503
FB_0782146_L9_PA	fp09_24	9	24.503
FB_0782168_L9_PA	fp09_24	9	24.503
FB_0782162_L9_PA	fp09_24	9	24.503
FB_0782383_L9_PA	fp09_24	9	24.503
FB_0782169_L9_PA	fp09_24	9	24.503
FB_1105115_L9_39_2	fp09_24	9	24.503
RB_6761696_L9_PA	fp09_25	9	25.321
FB_0783062_L9_PA	fp09_25	9	25.321
GD_01706_L9_PA	fp09_25	9	25.321
FB_0782812_L9_PA	fp09_25	9	25.321
FB_0782803_L9_PA	fp09_25	9	25.321
RB_7228622_L9_PA	fp09_25	9	25.321
FB_1105107_L9_39_2	fp09_25	9	25.321
FB_0783047_L9_PA	fp09_25	9	25.321
RB_6782368_L9_PA	fp09_25	9	25.321
FB_0783895_L9_PA	fp09_25	9	25.321
FB_0783050_L9_PA	fp09_25	9	25.321
FB_0783061_L9_PA	fp09_25	9	25.321
RB_7211083_L9_PA	fp09_25	9	25.321
RB_7705858_L9_PA	fp09_25	9	25.321
FB_0783048_L9_PA	fp09_25	9	25.321
RB_7727491_L9_PA	fp09_25	9	25.321
RB_7216327_L9_PA	fp09_25	9	25.321
FB_0783908_L9_PA	fp09_25	9	25.321
RB_7255390_L9_PA	fp09_25	9	25.321
FB_1105154_L9_39_2	fp09_25	9	25.321
FB_1105099_L9_39_2	fp09_25	9	25.321

RB_7220033_L9_PA	fp09_25	9	25.321
FB_1105162_L9_39_2	fp09_25	9	25.321
GD_00893_L9_PA	fp09_25	9	25.321
FB_0783899_L9_PA	fp09_25	9	25.321
FB_1105156_L9_39_2	fp09_25	9	25.321
FB_0783905_L9_PA	fp09_25	9	25.321
FB_0783912_L9_PA	fp09_25	9	25.321
FB_0783900_L9_PA	fp09_25	9	25.321
FB_0002378_L10_PA	fp09_26	9	26.201
FB_1105245_L9_39_2	fp09_26	9	26.201
FB_1105230_L9_39_2	fp09_26	9	26.201
FB_1105231_L9_39_2	fp09_26	9	26.201
FB_0785025_L9_PA	fp09_28	9	28.662
FB_1043371_L9_21_1	fp09_28	9	28.662
FB_1043369_L9_21_1	fp09_28	9	28.662
RB_8229716_L9_20_1	fp09_28	9	28.662
RB_10476886_L9_PA	fp09_29	9	29.566
FB_1105237_L9_39_2	fp09_30	9	30.51
FB_0787430_L9_PA	fp09_30	9	30.51
FB_0787090_L9_PA	fp09_30	9	30.51
FB_0787106_L9_PA	fp09_30	9	30.51
FB_0787424_L9_PA	fp09_30	9	30.51
FB_0787092_L9_PA	fp09_30	9	30.51
FB_0787418_L9_PA	fp09_30	9	30.51
FB_0787086_L9_PA	fp09_30	9	30.51
FB_0787420_L9_PA	fp09_30	9	30.51
FB_0787089_L9_PA	fp09_30	9	30.51
FB_1043360_L9_21_1	fp09_30	9	30.51
GD_00337_L9_PA	fp09_30	9	30.51
FB_1044384_L9_23_1	fp09_31	9	31.692
FB_1044388_L9_23_1	fp09_31	9	31.692
FB_1044389_L9_23_1	fp09_31	9	31.692
FB_0789986_L9_PA	fp09_31	9	31.692
FB_0789999_L9_PA	fp09_31	9	31.692
RB_11731639_L9_23_1	fp09_31	9	31.692
RB_11729185_L9_23_1	fp09_31	9	31.692
FB_0790000_L9_PA	fp09_31	9	31.692
RB_11719235_L9_23_1	fp09_33	9	33.492
RB_11711622_L9_PA	fp09_33	9	33.492
FB_0789989_L9_PA	fp09_33	9	33.492
FB_0791066_L9_PA	fp09_33	9	33.492
FB_0790672_L9_PA	fp09_34	9	34.191
FB_0787778_L9_PA	fp09_34	9	34.191
GD_02031_L9_PA	fp09_34	9	34.191
FB_0787786_L9_PA	fp09_34	9	34.191
FB_0787772_L9_PA	fp09_34	9	34.191

FB_0790677_L9_PA	fp09_34	9	34.191
FB_0790664_L9_PA	fp09_34	9	34.191
FB_0787782_L9_PA	fp09_34	9	34.191
FB_0791606_L9_PA	fp09_34	9	34.191
FB_0791612_L9_PA	fp09_34	9	34.191
FB_0787093_L9_PA	fp09_34	9	34.191
FB_1105799_L9_42_2	fp09_34	9	34.191
FB_0790679_L9_PA	fp09_35	9	35.814
FB_0790661_L9_PA	fp09_35	9	35.814
RB_7699964_L9_PA	fp09_36	9	36.153
FB_0791077_L9_PA	fp09_39	9	39.775
FB_0792090_L9_PA	fp09_39	9	39.775
FB_0791063_L9_PA	fp09_39	9	39.775
GD_00947_L9_PA	fp09_39	9	39.775
FB_1105804_L9_42_2	fp09_39	9	39.775
FB_0791078_L9_PA	fp09_39	9	39.775
FB_0791064_L9_PA	fp09_39	9	39.775
FB_0791057_L9_PA	fp09_39	9	39.775
FB_0791592_L9_PA	fp09_39	9	39.775
FB_0792110_L9_PA	fp09_40	9	40.377
RB_12880004_L9_PA	fp09_40	9	40.377
RB_12886110_L9_PA	fp09_40	9	40.377
GD_00331_L9_PA	fp09_40	9	40.377
FB_0792092_L9_PA	fp09_40	9	40.377
FB_1105236_L9_39_2	fp09_41	9	41.188
FB_0787084_L9_PA	fp09_41	9	41.188
RB_8291226_L9_PA	fp09_41	9	41.188
FB_1105224_L9_39_2	fp09_41	9	41.188
RB_8236405_L9_PA	fp09_41	9	41.188
RB_8269944_L9_PA	fp09_41	9	41.188
FB_1105228_L9_39_2	fp09_41	9	41.188
FB_0785012_L9_PA	fp09_41	9	41.188
FB_0785023_L9_PA	fp09_41	9	41.188
GD_01755_L9_23_1	fp09_41	9	41.188
FB_1044374_L9_23_1	fp09_41	9	41.188
FB_0790667_L9_PA	fp09_41	9	41.188
FB_0793636_L9_PA	fp09_41	9	41.188
RB_12849756_L9_PA	fp09_42	9	42.752
FB_0791596_L9_PA	fp09_42	9	42.752
FB_0792098_L9_PA	fp09_42	9	42.752
RB_14350830_L9_PA	fp09_42	9	42.752
FB_0792713_L9_PA	fp09_42	9	42.752
RB_14241533_L9_PA	fp09_42	9	42.752
FB_0793632_L9_PA	fp09_42	9	42.752
FB_0794276_L9_PA	fp09_42	9	42.752
FB_0792673_L9_PA	fp09_42	9	42.752

FB_0794263_L9_PA	fp09_42	9	42.752
FB_0794257_L9_PA	fp09_42	9	42.752
FB_0794261_L9_PA	fp09_42	9	42.752
FB_0227481_L14_PA	fp09_42	9	42.752
FB_0792679_L9_PA	fp09_42	9	42.752
FB_0792096_L9_PA	fp09_43	9	43.405
RB_12845175_L9_PA	fp09_43	9	43.405
FB_0793624_L9_PA	fp09_43	9	43.405
FB_0227483_L14_PA	fp09_43	9	43.405
FB_0793631_L9_PA	fp09_43	9	43.405
FB_0794278_L9_PA	fp09_43	9	43.405
FB_0227492_L14_PA	fp09_43	9	43.405
RB_14233188_L9_PA	fp09_43	9	43.405
FB_0793639_L9_PA	fp09_43	9	43.405
FB_0227487_L14_PA	fp09_43	9	43.405
FB_0227479_L14_PA	fp09_43	9	43.405
FB_0794247_L9_PA	fp09_43	9	43.405
FB_0794243_L9_PA	fp09_43	9	43.405
FB_0227518_L14_PA	fp09_43	9	43.405
GD_00958_L9_PA	fp09_43	9	43.405
FB_0798584_L9_PA	fp09_44	9	44.356
FB_0793621_L9_PA	fp09_44	9	44.356
RB_12825468_L9_PA	fp09_44	9	44.356
RB_18224270_L9_PA	fp09_45	9	45.844
FB_0799264_L9_PA	fp09_45	9	45.844
FB_0798596_L9_PA	fp09_45	9	45.844
FB_0799256_L9_PA	fp09_45	9	45.844
FB_0799250_L9_PA	fp09_45	9	45.844
FB_0799260_L9_PA	fp09_45	9	45.844
FB_0799259_L9_PA	fp09_45	9	45.844
FB_0794757_L9_PA	fp09_45	9	45.844
GD_02046_L9_PA	fp09_45	9	45.844
FB_0799262_L9_PA	fp09_45	9	45.844
FB_0798583_L9_PA	fp09_45	9	45.844
FB_0799244_L9_PA	fp09_45	9	45.844
RB_18289707_L9_PA	fp09_46	9	46.446
RB_18272664_L9_PA	fp09_46	9	46.446
RB_18243159_L9_PA	fp09_46	9	46.446
FB_0799274_L9_PA	fp09_46	9	46.446
RB_18226904_L9_PA	fp09_46	9	46.446
FB_0204654_L13_PA	fp09_48	9	48.157
FB_0204649_L13_PA	fp09_48	9	48.157
FB_0203803_L13_PA	fp09_50	9	50.677
FB_0201197_L13_PA	fp09_50	9	50.677
FB_0202592_L13_PA	fp09_50	9	50.677
FB_0201634_L13_PA	fp09_50	9	50.677

FB_0202588_L13_PA	fp09_50	9	50.677
FB_0201642_L13_PA	fp09_50	9	50.677
FB_0202084_L13_PA	fp09_50	9	50.677
FB_0880168_L13_46_1	fp09_50	9	50.677
FB_0201188_L13_PA	fp09_50	9	50.677
FB_0202275_L13_PA	fp09_50	9	50.677
FB_0800273_L9_PA	fp09_50	9	50.677
FB_0800269_L9_PA	fp09_50	9	50.677
FB_0800272_L9_PA	fp09_50	9	50.677
FB_0800289_L9_PA	fp09_50	9	50.677
FB_0202077_L13_PA	fp09_51	9	51.408
FB_0202087_L13_PA	fp09_51	9	51.408
FB_0202090_L13_PA	fp09_51	9	51.408
FB_0202076_L13_PA	fp09_51	9	51.408
FB_0880352_L13_46_1	fp09_51	9	51.408
FB_0880343_L13_46_1	fp09_51	9	51.408
GD_02100_L9_PA	fp09_52	9	52.311
FB_0202071_L13_PA	fp09_52	9	52.311
FB_0802850_L9_PA	fp09_53	9	53.186
GD_02451_L9_28_1	fp09_53	9	53.186
FB_0802846_L9_PA	fp09_53	9	53.186
FB_0802848_L9_PA	fp09_53	9	53.186
FB_0805660_L9_PA	fp09_54	9	54.728
FB_0197968_L13_PA	fp09_54	9	54.728
FB_0803510_L9_PA	fp09_54	9	54.728
GD_01648_L9_27_1	fp09_54	9	54.728
RB_20642843_L9_PA	fp09_54	9	54.728
FB_0804877_L9_PA	fp09_54	9	54.728
FB_0804880_L9_PA	fp09_54	9	54.728
FB_1047870_L9_28_1	fp09_54	9	54.728
FB_0197969_L13_PA	fp09_54	9	54.728
FB_0803520_L9_PA	fp09_54	9	54.728
FB_0803243_L9_PA	fp09_54	9	54.728
FB_1047223_L9_27_1	fp09_54	9	54.728
RB_20592637_L9_27_1	fp09_54	9	54.728
RB_21597933_L9_28_1	fp09_54	9	54.728
FB_1047472_L9_27_1	fp09_54	9	54.728
FB_0803517_L9_PA	fp09_54	9	54.728
RB_21623475_L9_PA	fp09_54	9	54.728
FB_0134182_L12_PA	fp09_54	9	54.728
FB_0134179_L12_PA	fp09_54	9	54.728
FB_0134191_L12_PA	fp09_54	9	54.728
GD_02230_L9_PA	fp09_54	9	54.728
FB_0134173_L12_PA	fp09_54	9	54.728
RB_21618606_L9_PA	fp09_54	9	54.728
FB_0134169_L12_PA	fp09_54	9	54.728

FB_0802851_L9_PA	fp09_54	9	54.728
FB_0802844_L9_PA	fp09_55	9	55.513
FB_0804485_L9_PA	fp09_55	9	55.513
FB_0805169_L9_PA	fp09_55	9	55.513
FB_0805042_L9_PA	fp09_56	9	56.7
FB_0960401_L2_36_1	fp09_56	9	56.7
FB_0944191_L1_56_1	fp09_56	9	56.7
GD_00053_L9_PA	fp09_56	9	56.7
FB_0944210_L1_56_1	fp09_56	9	56.7
FB_1047887_L9_28_1	fp09_56	9	56.7
FB_0812495_L9_PA	fp09_56	9	56.7
FB_1049022_L9_34_1	fp09_56	9	56.7
FB_0812507_L9_PA	fp09_56	9	56.7
FB_1049021_L9_34_1	fp09_56	9	56.7
FB_0806823_L9_PA	fp09_56	9	56.7
FB_0534784_L3_PA	fp09_56	9	56.7
FB_0812478_L9_PA	fp09_56	9	56.7
FB_0810587_L9_PA	fp09_56	9	56.7
FB_0804878_L9_PA	fp09_56	9	56.7
FB_0802843_L9_PA	fp09_56	9	56.7
FB_0201203_L13_PA	fp09_56	9	56.7
FB_0201630_L13_PA	fp09_56	9	56.7
FB_1047224_L9_27_1	fp09_56	9	56.7
FB_0201184_L13_PA	fp09_56	9	56.7
FB_0201629_L13_PA	fp09_56	9	56.7
FB_0201213_L13_PA	fp09_56	9	56.7
RB_20564976_L9_PA	fp09_56	9	56.7
FB_0805650_L9_PA	fp09_56	9	56.7
FB_0534756_L3_PA	fp09_57	9	57.738
FB_1047894_L9_28_1	fp09_57	9	57.738
FB_0497867_L2_PA	fp09_57	9	57.738
FB_0960398_L2_36_1	fp09_57	9	57.738
FB_0197962_L13_PA	fp09_57	9	57.738
FB_0497856_L2_PA	fp09_57	9	57.738
FB_0813263_L9_PA	fp09_57	9	57.738
FB_0813119_L9_PA	fp09_57	9	57.738
RB_29824342_L9_PA	fp09_57	9	57.738
FB_0813115_L9_PA	fp09_57	9	57.738
RB_29726331_L9_PA	fp09_57	9	57.738
FB_0813108_L9_PA	fp09_57	9	57.738
FB_0813107_L9_PA	fp09_57	9	57.738
GD_02437_L9_PA	fp09_57	9	57.738
FB_0813109_L9_PA	fp09_57	9	57.738
RB_29766970_L9_PA	fp09_60	9	60.347
FB_0805155_L9_PA	fp09_60	9	60.347
FB_0812477_L9_PA	fp09_61	9	61.242

FB_0813123_L9_PA	fp09_62	9	62.546
RB_29730777_L9_PA	fp09_62	9	62.546
FB_0814184_L9_PA	fp09_62	9	62.546
FB_0813223_L9_PA	fp09_62	9	62.546
FB_0822798_L9_PA	fp09_62	9	62.546
RB_24219741_L9_PA	fp09_62	9	62.546
FB_0822800_L9_PA	fp09_62	9	62.546
FB_1051896_L9_37_1	fp09_62	9	62.546
FB_1048942_L9_34_1	fp09_62	9	62.546
FB_0810580_L9_PA	fp09_62	9	62.546
FB_0814176_L9_PA	fp09_62	9	62.546
FB_0810589_L9_PA	fp09_62	9	62.546
FB_0822823_L9_PA	fp09_62	9	62.546
FB_1051895_L9_37_1	fp09_62	9	62.546
GD_00860_L9_PA	fp09_62	9	62.546
FB_0822803_L9_PA	fp09_62	9	62.546
RB_35913773_L9_PA	fp09_62	9	62.546
FB_0534771_L3_PA	fp09_62	9	62.546
FB_0507142_L3_PA	fp09_62	9	62.546
FB_1048937_L9_34_1	fp09_62	9	62.546
FB_0805044_L9_PA	fp09_62	9	62.546
FB_0813256_L9_PA	fp09_62	9	62.546
FB_0813129_L9_PA	fp09_63	9	63.698
FB_0815024_L9_PA	fp09_63	9	63.698
FB_0815027_L9_PA	fp09_63	9	63.698
FB_0815023_L9_PA	fp09_63	9	63.698
FB_0815086_L9_PA	fp09_63	9	63.698
GD_02482_L9_PA	fp09_63	9	63.698
FB_0904386_L15_48_1	fp09_63	9	63.698
FB_0815025_L9_PA	fp09_63	9	63.698
FB_0281021_L15_PA	fp09_64	9	64.578
FB_0281019_L15_PA	fp09_64	9	64.578
FB_0822825_L9_PA	fp09_64	9	64.578
FB_0904389_L15_48_1	fp09_64	9	64.578
FB_0281006_L15_PA	fp09_64	9	64.578
FB_0817523_L9_PA	fp09_64	9	64.578
FB_0280505_L15_PA	fp09_64	9	64.578
RB_33227831_L9_PA	fp09_64	9	64.578
FB_0904394_L15_48_1	fp09_64	9	64.578
FB_0281008_L15_PA	fp09_64	9	64.578
FB_0819956_L9_PA	fp09_64	9	64.578
RB_31984731_L9_PA	fp09_64	9	64.578
RB_32711352_L9_PA	fp09_64	9	64.578
FB_1051643_L9_36_1	fp09_64	9	64.578
RB_31701102_L9_PA	fp09_64	9	64.578
RB_31964859_L9_PA	fp09_64	9	64.578

FB_0280501_L15_PA	fp09_64	9	64.578
FB_0824611_L9_PA	fp09_64	9	64.578
FB_0280533_L15_PA	fp09_64	9	64.578
FB_0824598_L9_PA	fp09_64	9	64.578
FB_0280995_L15_PA	fp09_64	9	64.578
FB_0904385_L15_48_1	fp09_64	9	64.578
RB_31685318_L9_PA	fp09_64	9	64.578
RB_32540237_L9_PA	fp09_64	9	64.578
FB_1050859_L9_36_1	fp09_64	9	64.578
FB_0280992_L15_PA	fp09_64	9	64.578
RB_32297817_L9_PA	fp09_64	9	64.578
FB_0280524_L15_PA	fp09_64	9	64.578
GD_00467_L9_PA	fp09_64	9	64.578
FB_0824002_L9_PA	fp09_64	9	64.578
GD_01293_L9_PA	fp09_64	9	64.578
FB_0824001_L9_PA	fp09_64	9	64.578
RB_33180045_L9_PA	fp09_64	9	64.578
FB_0280549_L15_PA	fp09_64	9	64.578
FB_0816029_L9_PA	fp09_64	9	64.578
FB_0904411_L15_48_1	fp09_64	9	64.578
FB_1050865_L9_36_1	fp09_64	9	64.578
FB_0824584_L9_PA	fp09_64	9	64.578
FB_1106925_L9_51_2	fp09_64	9	64.578
FB_0816037_L9_PA	fp09_64	9	64.578
FB_0816035_L9_PA	fp09_64	9	64.578
GD_02059_L9_36_1	fp09_64	9	64.578
FB_0816821_L9_PA	fp09_64	9	64.578
FB_0816027_L9_PA	fp09_64	9	64.578
FB_0820946_L9_PA	fp09_64	9	64.578
RB_32531545_L9_PA	fp09_64	9	64.578
FB_1106928_L9_51_2	fp09_64	9	64.578
FB_1106933_L9_51_2	fp09_64	9	64.578
RB_32329275_L9_PA	fp09_64	9	64.578
RB_31694289_L9_PA	fp09_64	9	64.578
FB_0816028_L9_PA	fp09_64	9	64.578
RB_31860120_L9_PA	fp09_64	9	64.578
FB_0816809_L9_PA	fp09_64	9	64.578
FB_0817757_L9_PA	fp09_64	9	64.578
RB_33065704_L9_PA	fp09_64	9	64.578
FB_1106937_L9_51_2	fp09_64	9	64.578
GD_00659_L9_PA	fp09_64	9	64.578
FB_1107066_L9_51_2	fp09_64	9	64.578
FB_0816820_L9_PA	fp09_64	9	64.578
FB_0816014_L9_PA	fp09_64	9	64.578
FB_0819937_L9_PA	fp09_64	9	64.578
FB_0816808_L9_PA	fp09_64	9	64.578

FB_0816839_L9_PA	fp09_64	9	64.578
FB_0817519_L9_PA	fp09_65	9	65.581
RB_32698252_L9_PA	fp09_65	9	65.581
FB_0817506_L9_PA	fp09_65	9	65.581
FB_0817501_L9_PA	fp09_65	9	65.581
FB_0817515_L9_PA	fp09_65	9	65.581
FB_0817531_L9_PA	fp09_65	9	65.581
FB_0817504_L9_PA	fp09_65	9	65.581
FB_0817512_L9_PA	fp09_65	9	65.581
FB_0817516_L9_PA	fp09_65	9	65.581
FB_0817513_L9_PA	fp09_65	9	65.581
FB_1107085_L9_51_2	fp09_65	9	65.581
RB_33439813_L9_PA	fp09_66	9	66.279
FB_1050852_L9_36_1	fp09_66	9	66.279
RB_33185982_L9_PA	fp09_66	9	66.279
FB_1050862_L9_36_1	fp09_66	9	66.279
RB_33077622_L9_PA	fp09_66	9	66.279
FB_0817780_L9_PA	fp09_66	9	66.279
FB_0818945_L9_PA	fp09_66	9	66.279
FB_1050856_L9_36_1	fp09_66	9	66.279
FB_0818944_L9_PA	fp09_66	9	66.279
FB_1107065_L9_51_2	fp09_66	9	66.279
FB_0817758_L9_PA	fp09_66	9	66.279
FB_0817767_L9_PA	fp09_66	9	66.279
GD_00206_L9_PA	fp09_66	9	66.279
FB_1107078_L9_51_2	fp09_66	9	66.279
GD_00452_L9_36_1	fp09_66	9	66.279
FB_0819314_L9_PA	fp09_66	9	66.279
FB_0819941_L9_PA	fp09_67	9	67.382
FB_0821669_L9_PA	fp09_67	9	67.382
FB_0820945_L9_PA	fp09_67	9	67.382
FB_0819949_L9_PA	fp09_67	9	67.382
FB_0819942_L9_PA	fp09_67	9	67.382
FB_0820931_L9_PA	fp09_67	9	67.382
GD_01200_L9_PA	fp09_67	9	67.382
FB_0819954_L9_PA	fp09_67	9	67.382
FB_0821667_L9_PA	fp09_67	9	67.382
FB_1050833_L9_36_1	fp09_67	9	67.382
GD_00332_L9_PA	fp09_68	9	68.564
FB_0820932_L9_PA	fp09_69	9	69.128
FB_0820935_L9_PA	fp09_69	9	69.128
FB_0000878_L10_PA	fp10_00	10	0.116
FB_0000517_L10_PA	fp10_00	10	0.116
FB_0216378_L14_PA	fp10_00	10	0.116
FB_0000870_L10_PA	fp10_00	10	0.116
FB_0000011_L10_PA	fp10_00	10	0.116

FB_0000880_L10_PA	fp10_00	10	0.116
GD_00106_L10_PA	fp10_00	10	0.116
FB_0824887_L10_27_1	fp10_00	10	0.116
FB_0000529_L10_PA	fp10_00	10	0.116
FB_0824881_L10_27_1	fp10_00	10	0.116
FB_0216486_L14_PA	fp10_00	10	0.116
FB_0000521_L10_PA	fp10_00	10	0.116
FB_0216376_L14_PA	fp10_00	10	0.116
Vd_DCA1_Gluca	fp10_00	10	0.116
FB_0000582_L10_PA	fp10_01	10	1.81
FB_0000981_L10_PA	fp10_01	10	1.81
FB_0825056_L10_28_1	fp10_01	10	1.81
FB_0000978_L10_PA	fp10_01	10	1.81
FB_0000986_L10_PA	fp10_01	10	1.81
FB_0000996_L10_PA	fp10_01	10	1.81
RB_869972_L10_PA	fp10_01	10	1.81
FB_0001467_L10_PA	fp10_01	10	1.81
FB_0001475_L10_PA	fp10_01	10	1.81
FB_0001462_L10_PA	fp10_01	10	1.81
FB_0000993_L10_PA	fp10_01	10	1.81
FB_0000984_L10_PA	fp10_01	10	1.81
FB_0000989_L10_PA	fp10_01	10	1.81
FB_0825194_L10_28_1	fp10_01	10	1.81
FB_0000988_L10_PA	fp10_02	10	2.323
FB_0001468_L10_PA	fp10_02	10	2.323
FB_0825061_L10_28_1	fp10_02	10	2.323
FB_0001465_L10_PA	fp10_02	10	2.323
FB_0825201_L10_28_1	fp10_02	10	2.323
FB_0825199_L10_28_1	fp10_02	10	2.323
FB_0825197_L10_28_1	fp10_02	10	2.323
FB_1106438_L9_46_2	fp10_02	10	2.323
FB_0003101_L10_PA	fp10_03	10	3.629
FB_0825447_L10_30_1	fp10_04	10	4.275
FB_0003107_L10_PA	fp10_04	10	4.275
FB_0002649_L10_PA	fp10_04	10	4.275
FB_0002636_L10_PA	fp10_04	10	4.275
FB_0003491_L10_PA	fp10_04	10	4.275
FB_0003106_L10_PA	fp10_04	10	4.275
FB_0003485_L10_PA	fp10_04	10	4.275
FB_0003079_L10_PA	fp10_04	10	4.275
FB_0004037_L10_PA	fp10_04	10	4.275
FB_0002653_L10_PA	fp10_04	10	4.275
FB_0825437_L10_30_1	fp10_04	10	4.275
FB_0004016_L10_PA	fp10_04	10	4.275
FB_0002632_L10_PA	fp10_04	10	4.275
GD_01909_L10_PA	fp10_04	10	4.275

FB_0825439_L10_30_1	fp10_04	10	4.275
RB_2484105_L10_PA	fp10_04	10	4.275
FB_0002639_L10_PA	fp10_04	10	4.275
FB_0002637_L10_PA	fp10_04	10	4.275
FB_0003104_L10_PA	fp10_04	10	4.275
FB_0003096_L10_PA	fp10_04	10	4.275
FB_0003118_L10_PA	fp10_04	10	4.275
FB_0004019_L10_PA	fp10_04	10	4.275
FB_0003489_L10_PA	fp10_04	10	4.275
FB_0003490_L10_PA	fp10_04	10	4.275
FB_0004038_L10_PA	fp10_04	10	4.275
FB_0825590_L10_30_1	fp10_04	10	4.275
FB_0004046_L10_PA	fp10_04	10	4.275
FB_0003114_L10_PA	fp10_04	10	4.275
FB_0004044_L10_PA	fp10_04	10	4.275
RB_3294683_L10_PA	fp10_04	10	4.275
RB_3303962_L10_PA	fp10_05	10	5.287
RB_2563548_L10_PA	fp10_05	10	5.287
GD_00839_L10_PA	fp10_05	10	5.287
FB_0003482_L10_PA	fp10_05	10	5.287
FB_0004014_L10_PA	fp10_05	10	5.287
RB_3285289_L10_PA	fp10_06	10	6.774
RB_4034839_L10_PA	fp10_07	10	7.94
FB_0275982_L15_PA	fp10_07	10	7.94
FB_0275984_L15_PA	fp10_07	10	7.94
FB_0005008_L10_PA	fp10_07	10	7.94
FB_0005006_L10_PA	fp10_07	10	7.94
FB_0004993_L10_PA	fp10_08	10	8.596
RB_4041727_L10_PA	fp10_08	10	8.596
RB_4045102_L10_PA	fp10_08	10	8.596
GD_01810_L10_PA	fp10_08	10	8.596
FB_0055223_L11_PA	fp10_08	10	8.596
FB_0055224_L11_PA	fp10_08	10	8.596
FB_0055188_L11_PA	fp10_08	10	8.596
FB_0826633_L10_33_1	fp10_08	10	8.596
FB_0055205_L11_PA	fp10_08	10	8.596
FB_0839802_L11_26_1	fp10_08	10	8.596
FB_0010475_L10_PA	fp10_08	10	8.596
FB_0055202_L11_PA	fp10_08	10	8.596
FB_0008635_L10_PA	fp10_09	10	9.622
FB_0008637_L10_PA	fp10_09	10	9.622
FB_0826641_L10_33_1	fp10_09	10	9.622
FB_0839808_L11_26_1	fp10_09	10	9.622
FB_0009209_L10_PA	fp10_09	10	9.622
FB_0009237_L10_PA	fp10_09	10	9.622
FB_0009214_L10_PA	fp10_09	10	9.622

FB_0009745_L10_PA	fp10_09	10	9.622
GD_02051_L10_PA	fp10_09	10	9.622
FB_0009751_L10_PA	fp10_09	10	9.622
FB_0009773_L10_PA	fp10_09	10	9.622
FB_0009753_L10_PA	fp10_09	10	9.622
RB_7135490_L10_PA	fp10_09	10	9.622
RB_7167616_L10_33_1	fp10_09	10	9.622
FB_0009256_L10_PA	fp10_09	10	9.622
FB_0009203_L10_PA	fp10_09	10	9.622
FB_0009220_L10_PA	fp10_09	10	9.622
FB_0826637_L10_33_1	fp10_09	10	9.622
FB_0008612_L10_PA	fp10_10	10	10.313
FB_0009218_L10_PA	fp10_10	10	10.313
FB_0009219_L10_PA	fp10_10	10	10.313
FB_0010446_L10_PA	fp10_10	10	10.313
FB_0716938_L7_PA	fp10_10	10	10.313
GD_00875_L10_PA	fp10_10	10	10.313
FB_0010458_L10_PA	fp10_10	10	10.313
FB_0716924_L7_PA	fp10_10	10	10.313
FB_0716927_L7_PA	fp10_10	10	10.313
RB_9480406_L10_PA	fp10_11	10	11.437
RB_9629310_L10_PA	fp10_11	10	11.437
RB_9629001_L10_PA	fp10_11	10	11.437
RB_7121587_L10_PA	fp10_11	10	11.437
FB_0016736_L10_PA	fp10_11	10	11.437
FB_0122289_L12_PA	fp10_11	10	11.437
FB_0551208_L3_PA	fp10_11	10	11.437
FB_0296212_L15_PA	fp10_11	10	11.437
FB_0296210_L15_PA	fp10_11	10	11.437
FB_0014725_L10_PA	fp10_11	10	11.437
RB_7179858_L10_PA	fp10_12	10	12.751
FB_0015682_L10_PA	fp10_12	10	12.751
RB_11942818_L10_PA	fp10_12	10	12.751
FB_1023006_L8_33_1	fp10_12	10	12.751
FB_1100846_L8_62_2	fp10_12	10	12.751
FB_0726166_L8_PA	fp10_12	10	12.751
FB_0014738_L10_PA	fp10_12	10	12.751
FB_0726979_L8_PA	fp10_12	10	12.751
FB_0014529_L10_PA	fp10_12	10	12.751
RB_12048247_L10_PA	fp10_12	10	12.751
FB_0015893_L10_PA	fp10_12	10	12.751
FB_0017226_L10_PA	fp10_12	10	12.751
FB_0014551_L10_PA	fp10_12	10	12.751
FB_0014722_L10_PA	fp10_12	10	12.751
FB_0016721_L10_PA	fp10_12	10	12.751
FB_0829833_L10_37_1	fp10_12	10	12.751

FB_0016473_L10_PA	fp10_12	10	12.751
FB_0016741_L10_PA	fp10_12	10	12.751
FB_0015919_L10_PA	fp10_12	10	12.751
RB_13071892_L10_PA	fp10_12	10	12.751
FB_0014567_L10_PA	fp10_12	10	12.751
FB_0014558_L10_PA	fp10_12	10	12.751
FB_0014724_L10_PA	fp10_12	10	12.751
RB_13016569_L10_PA	fp10_12	10	12.751
FB_0015921_L10_PA	fp10_12	10	12.751
FB_0014555_L10_PA	fp10_12	10	12.751
RB_12053580_L10_PA	fp10_12	10	12.751
FB_0016482_L10_PA	fp10_12	10	12.751
FB_0017215_L10_PA	fp10_12	10	12.751
GD_01867_L10_PA	fp10_12	10	12.751
RB_10765940_L10_PA	fp10_12	10	12.751
RB_13033111_L10_PA	fp10_12	10	12.751
FB_0017224_L10_PA	fp10_12	10	12.751
RB_10712688_L10_PA	fp10_12	10	12.751
FB_0016486_L10_PA	fp10_12	10	12.751
FB_0014746_L10_PA	fp10_12	10	12.751
FB_0017217_L10_PA	fp10_12	10	12.751
RB_12433407_L10_PA	fp10_12	10	12.751
FB_0014728_L10_PA	fp10_12	10	12.751
FB_0016712_L10_PA	fp10_12	10	12.751
FB_0014721_L10_PA	fp10_12	10	12.751
FB_0016692_L10_PA	fp10_12	10	12.751
FB_0015895_L10_PA	fp10_12	10	12.751
FB_0829841_L10_37_1	fp10_12	10	12.751
FB_0726977_L8_PA	fp10_12	10	12.751
FB_0727275_L8_PA	fp10_12	10	12.751
FB_0727268_L8_PA	fp10_12	10	12.751
FB_1023458_L8_33_1	fp10_12	10	12.751
FB_0727271_L8_PA	fp10_12	10	12.751
FB_0017214_L10_PA	fp10_12	10	12.751
FB_1022997_L8_33_1	fp10_12	10	12.751
FB_0726173_L8_PA	fp10_12	10	12.751
FB_1100858_L8_62_2	fp10_12	10	12.751
FB_0726172_L8_PA	fp10_13	10	13.288
FB_0726189_L8_PA	fp10_13	10	13.288
FB_0230393_L14_PA	fp10_13	10	13.288
FB_0263752_L15_PA	fp10_13	10	13.288
FB_1086040_L2_51_2	fp10_13	10	13.288
FB_0727256_L8_PA	fp10_13	10	13.288
RB_12422488_L10_PA	fp10_13	10	13.288
RB_11992593_L10_PA	fp10_13	10	13.288
RB_11952578_L10_PA	fp10_13	10	13.288

FB_0015691_L10_PA	fp10_13	10	13.288
FB_0015689_L10_PA	fp10_13	10	13.288
FB_1100845_L8_62_2	fp10_14	10	14.517
FB_0230400_L14_PA	fp10_14	10	14.517
FB_0964217_L3_33_1	fp10_14	10	14.517
FB_1086054_L2_51_2	fp10_14	10	14.517
FB_0014146_L10_PA	fp10_14	10	14.517
FB_0014148_L10_PA	fp10_14	10	14.517
FB_1086041_L2_51_2	fp10_14	10	14.517
FB_0727010_L8_PA	fp10_14	10	14.517
FB_1023455_L8_33_1	fp10_14	10	14.517
FB_1023008_L8_33_1	fp10_14	10	14.517
FB_0726982_L8_PA	fp10_14	10	14.517
FB_1023005_L8_33_1	fp10_14	10	14.517
FB_0726987_L8_PA	fp10_14	10	14.517
FB_1022996_L8_33_1	fp10_14	10	14.517
FB_1100840_L8_62_2	fp10_14	10	14.517
FB_1023456_L8_33_1	fp10_14	10	14.517
FB_1100851_L8_62_2	fp10_14	10	14.517
FB_0727267_L8_PA	fp10_14	10	14.517
FB_0726185_L8_PA	fp10_14	10	14.517
FB_1100884_L8_62_2	fp10_14	10	14.517
FB_0726164_L8_PA	fp10_14	10	14.517
FB_1115484_L4_40_3	fp10_14	10	14.517
FB_0263746_L15_PA	fp10_14	10	14.517
FB_0296218_L15_PA	fp10_14	10	14.517
FB_0296215_L15_PA	fp10_14	10	14.517
FB_0551257_L3_PA	fp10_14	10	14.517
FB_0551574_L3_PA	fp10_14	10	14.517
FB_1086046_L2_51_2	fp10_14	10	14.517
FB_0122479_L12_PA	fp10_15	10	15.62
FB_0014150_L10_PA	fp10_15	10	15.62
FB_0121827_L12_PA	fp10_15	10	15.62
FB_0122283_L12_PA	fp10_15	10	15.62
FB_1086045_L2_51_2	fp10_15	10	15.62
FB_1086057_L2_51_2	fp10_15	10	15.62
RB_8552134_L10_PA	fp10_15	10	15.62
FB_0964205_L3_33_1	fp10_15	10	15.62
FB_0122473_L12_PA	fp10_15	10	15.62
FB_1086048_L2_51_2	fp10_15	10	15.62
FB_0704833_L7_PA	fp10_15	10	15.62
FB_0017894_L10_PA	fp10_16	10	16.486
RB_13706234_L10_PA	fp10_16	10	16.486
FB_0017897_L10_PA	fp10_16	10	16.486
FB_0551559_L3_PA	fp10_16	10	16.486
FB_0704823_L7_PA	fp10_16	10	16.486

FB_0551236_L3_PA	fp10_16	10	16.486
FB_0704812_L7_PA	fp10_17	10	17.3
FB_1115483_L4_40_3	fp10_17	10	17.3
FB_0230392_L14_PA	fp10_17	10	17.3
FB_0964239_L3_33_1	fp10_17	10	17.3
FB_0263745_L15_PA	fp10_17	10	17.3
FB_0296224_L15_PA	fp10_17	10	17.3
FB_0964224_L3_33_1	fp10_17	10	17.3
FB_0766229_L8_PA	fp10_17	10	17.3
FB_0120452_L12_PA	fp10_17	10	17.3
FB_1115486_L4_40_3	fp10_17	10	17.3
FB_1070778_L15_112_2	fp10_17	10	17.3
FB_0230420_L14_PA	fp10_17	10	17.3
FB_1115489_L4_40_3	fp10_17	10	17.3
FB_0120463_L12_PA	fp10_17	10	17.3
FB_1115487_L4_40_3	fp10_17	10	17.3
FB_0188546_L13_PA	fp10_17	10	17.3
FB_0795112_L9_PA	fp10_17	10	17.3
FB_0551217_L3_PA	fp10_17	10	17.3
FB_0795113_L9_PA	fp10_18	10	18.241
FB_0188587_L13_PA	fp10_18	10	18.241
FB_0188550_L13_PA	fp10_18	10	18.241
FB_0188560_L13_PA	fp10_18	10	18.241
FB_0014155_L10_PA	fp10_18	10	18.241
FB_0296801_L15_PA	fp10_18	10	18.241
FB_0188543_L13_PA	fp10_18	10	18.241
FB_1089575_L3_81_2	fp10_18	10	18.241
FB_0188585_L13_PA	fp10_18	10	18.241
FB_0551223_L3_PA	fp10_18	10	18.241
FB_0551216_L3_PA	fp10_18	10	18.241
FB_0551553_L3_PA	fp10_18	10	18.241
FB_0017889_L10_PA	fp10_18	10	18.241
FB_0011655_L10_PA	fp10_19	10	19.576
RB_8522809_L10_33_1	fp10_19	10	19.576
FB_0890039_L14_39_1	fp10_19	10	19.576
FB_0890027_L14_39_1	fp10_19	10	19.576
FB_0685114_L6_PA	fp10_19	10	19.576
FB_0685099_L6_PA	fp10_19	10	19.576
FB_1073519_L15_94_2	fp10_19	10	19.576
FB_0013172_L10_PA	fp10_19	10	19.576
FB_0685112_L6_PA	fp10_20	10	20.688
RB_13708618_L10_PA	fp10_20	10	20.688
FB_1052855_L10_57_2	fp10_20	10	20.688
FB_1052863_L10_57_2	fp10_20	10	20.688
FB_0013175_L10_PA	fp10_20	10	20.688
FB_1107370_L10_73_3	fp10_20	10	20.688

FB_1107365_L10_73_3	fp10_20	10	20.688
FB_1052860_L10_57_2	fp10_20	10	20.688
FB_0013167_L10_PA	fp10_20	10	20.688
FB_0685103_L6_PA	fp10_20	10	20.688
FB_1107373_L10_73_3	fp10_20	10	20.688
FB_1052858_L10_57_2	fp10_20	10	20.688
FB_1052854_L10_57_2	fp10_20	10	20.688
FB_0013159_L10_PA	fp10_20	10	20.688
FB_0685122_L6_PA	fp10_20	10	20.688
FB_0685113_L6_PA	fp10_20	10	20.688
FB_0013184_L10_PA	fp10_20	10	20.688
FB_1107376_L10_73_3	fp10_20	10	20.688
FB_0012374_L10_PA	fp10_21	10	21.687
RB_8557916_L10_PA	fp10_21	10	21.687
FB_1052872_L10_57_2	fp10_21	10	21.687
FB_1052864_L10_57_2	fp10_21	10	21.687
FB_1107364_L10_73_3	fp10_21	10	21.687
GD_00260_L10_PA	fp10_21	10	21.687
FB_0013192_L10_PA	fp10_22	10	22.719
FB_0011683_L10_PA	fp10_22	10	22.719
FB_0011650_L10_PA	fp10_22	10	22.719
FB_0011662_L10_PA	fp10_22	10	22.719
FB_0011697_L10_PA	fp10_22	10	22.719
FB_0011658_L10_PA	fp10_22	10	22.719
FB_0011645_L10_PA	fp10_22	10	22.719
FB_1048041_L9_30_1	fp10_23	10	23.755
FB_1048040_L9_30_1	fp10_23	10	23.755
FB_0018994_L10_PA	fp10_23	10	23.755
RB_8508757_L10_33_1	fp10_23	10	23.755
FB_0805745_L9_PA	fp10_23	10	23.755
FB_0805710_L9_PA	fp10_23	10	23.755
FB_0805743_L9_PA	fp10_23	10	23.755
FB_1067928_L14_60_2	fp10_23	10	23.755
FB_1067940_L14_60_2	fp10_23	10	23.755
FB_0805702_L9_PA	fp10_23	10	23.755
FB_0012368_L10_PA	fp10_23	10	23.755
FB_1048060_L9_30_1	fp10_23	10	23.755
FB_1048043_L9_30_1	fp10_23	10	23.755
FB_0235546_L14_PA	fp10_23	10	23.755
FB_0235556_L14_PA	fp10_23	10	23.755
FB_1048063_L9_30_1	fp10_23	10	23.755
FB_0235541_L14_PA	fp10_23	10	23.755
FB_0805708_L9_PA	fp10_23	10	23.755
FB_0805750_L9_PA	fp10_23	10	23.755
FB_1067946_L14_60_2	fp10_23	10	23.755
FB_1048078_L9_30_1	fp10_23	10	23.755

FB_0805709_L9_PA	fp10_23	10	23.755
FB_0012361_L10_PA	fp10_23	10	23.755
FB_0235585_L14_PA	fp10_23	10	23.755
FB_0018992_L10_PA	fp10_24	10	24.476
GD_01267_L10_PA	fp10_24	10	24.476
FB_0018991_L10_PA	fp10_24	10	24.476
RB_14712598_L10_PA	fp10_24	10	24.476
RB_14662823_L10_PA	fp10_25	10	25.479
RB_14704244_L10_PA	fp10_25	10	25.479
FB_0890031_L14_39_1	fp10_25	10	25.479
RB_14673668_L10_PA	fp10_25	10	25.479
FB_0018986_L10_PA	fp10_25	10	25.479
RB_16552378_L10_PA	fp10_25	10	25.479
FB_0021357_L10_PA	fp10_25	10	25.479
RB_16680919_L10_62_2	fp10_25	10	25.479
RB_16666886_L10_40_1	fp10_25	10	25.479
FB_0021350_L10_PA	fp10_25	10	25.479
GD_00015_L10_PA	fp10_25	10	25.479
FB_1106430_L9_46_2	fp10_25	10	25.479
FB_0021345_L10_PA	fp10_25	10	25.479
FB_0021361_L10_PA	fp10_25	10	25.479
FB_0021360_L10_PA	fp10_25	10	25.479
FB_0021362_L10_PA	fp10_25	10	25.479
FB_0022007_L10_PA	fp10_25	10	25.479
FB_0021367_L10_PA	fp10_25	10	25.479
FB_0011664_L10_PA	fp10_25	10	25.479
RB_8533446_L10_PA	fp10_25	10	25.479
FB_0021657_L10_PA	fp10_26	10	26.486
FB_0021654_L10_PA	fp10_26	10	26.486
FB_0021346_L10_PA	fp10_26	10	26.486
FB_0021659_L10_PA	fp10_26	10	26.486
RB_16729529_L10_PA	fp10_26	10	26.486
FB_0296219_L15_PA	fp10_26	10	26.486
FB_0022025_L10_PA	fp10_26	10	26.486
RB_16718103_L10_PA	fp10_26	10	26.486
FB_0021652_L10_PA	fp10_26	10	26.486
FB_0023488_L10_PA	fp10_27	10	27.568
RB_16720641_L10_PA	fp10_27	10	27.568
RB_16997826_L10_PA	fp10_27	10	27.568
FB_0022019_L10_PA	fp10_27	10	27.568
RB_16726719_L10_PA	fp10_28	10	28.51
FB_0022001_L10_PA	fp10_28	10	28.51
FB_0021664_L10_PA	fp10_28	10	28.51
FB_0021653_L10_PA	fp10_28	10	28.51
FB_0022305_L10_PA	fp10_28	10	28.51
FB_0023045_L10_PA	fp10_30	10	30.417

FB_0024082_L10_PA	fp10_30	10	30.417
RB_16552703_L10_PA	fp10_30	10	30.417
RB_16552411_L10_PA	fp10_30	10	30.417
FB_0022313_L10_PA	fp10_30	10	30.417
FB_0022308_L10_PA	fp10_30	10	30.417
GD_02009_L10_PA	fp10_30	10	30.417
FB_0023486_L10_PA	fp10_31	10	31.146
FB_0021665_L10_PA	fp10_32	10	32.395
FB_0022314_L10_PA	fp10_32	10	32.395
FB_0023495_L10_PA	fp10_32	10	32.395
RB_17592310_L10_PA	fp10_32	10	32.395
RB_17032808_L10_PA	fp10_32	10	32.395
FB_0022301_L10_PA	fp10_32	10	32.395
FB_0022307_L10_PA	fp10_32	10	32.395
FB_0023034_L10_PA	fp10_33	10	33.465
GD_01987_L10_PA	fp10_33	10	33.465
FB_0023498_L10_PA	fp10_33	10	33.465
FB_0023491_L10_PA	fp10_33	10	33.465
FB_1087355_L3_63_2	fp10_33	10	33.465
RB_17046031_L10_PA	fp10_33	10	33.465
FB_0024057_L10_PA	fp10_33	10	33.465
RB_14684226_L10_PA	fp10_33	10	33.465
RB_17043353_L10_PA	fp10_33	10	33.465
FB_0023485_L10_PA	fp10_33	10	33.465
RB_17914122_L10_PA	fp10_33	10	33.465
RB_17911896_L10_PA	fp10_34	10	34.693
FB_0023484_L10_PA	fp10_34	10	34.693
FB_0296220_L15_PA	fp10_34	10	34.693
RB_18475950_L10_PA	fp10_34	10	34.693
RB_17894779_L10_PA	fp10_34	10	34.693
FB_0024056_L10_PA	fp10_34	10	34.693
FB_0024079_L10_PA	fp10_34	10	34.693
FB_0024620_L10_PA	fp10_34	10	34.693
FB_0024602_L10_PA	fp10_34	10	34.693
FB_0024628_L10_PA	fp10_34	10	34.693
FB_0024599_L10_PA	fp10_35	10	35.233
RB_17892578_L10_PA	fp10_35	10	35.233
FB_0027053_L10_PA	fp10_36	10	36.24
FB_0027504_L10_PA	fp10_36	10	36.24
FB_0023032_L10_PA	fp10_36	10	36.24
FB_0025080_L10_PA	fp10_36	10	36.24
FB_0025075_L10_PA	fp10_36	10	36.24
FB_0025095_L10_PA	fp10_36	10	36.24
FB_0026048_L10_PA	fp10_36	10	36.24
FB_0024622_L10_PA	fp10_36	10	36.24
FB_0831299_L10_40_1	fp10_36	10	36.24

FB_0026022_L10_PA	fp10_36	10	36.24
FB_0831640_L10_40_1	fp10_36	10	36.24
FB_0026051_L10_PA	fp10_36	10	36.24
FB_0026052_L10_PA	fp10_36	10	36.24
FB_0026055_L10_PA	fp10_37	10	37.727
FB_0025089_L10_PA	fp10_37	10	37.727
FB_0831641_L10_40_1	fp10_37	10	37.727
FB_0831298_L10_40_1	fp10_37	10	37.727
FB_0831297_L10_40_1	fp10_37	10	37.727
FB_0831296_L10_40_1	fp10_37	10	37.727
FB_0831315_L10_40_1	fp10_37	10	37.727
GD_01832_L10_40_1	fp10_37	10	37.727
FB_0831317_L10_40_1	fp10_37	10	37.727
FB_0831634_L10_40_1	fp10_39	10	39.786
FB_0026026_L10_PA	fp10_39	10	39.786
GD_02183_L10_PA	fp10_39	10	39.786
FB_0026038_L10_PA	fp10_39	10	39.786
FB_0026024_L10_PA	fp10_39	10	39.786
FB_0027479_L10_PA	fp10_39	10	39.786
FB_0027480_L10_PA	fp10_39	10	39.786
FB_0027507_L10_PA	fp10_39	10	39.786
FB_0026018_L10_PA	fp10_39	10	39.786
FB_0027058_L10_PA	fp10_40	10	40.372
FB_1107553_L10_76_3	fp10_40	10	40.372
FB_0028123_L10_PA	fp10_40	10	40.372
FB_1107552_L10_76_3	fp10_40	10	40.372
FB_1107554_L10_76_3	fp10_40	10	40.372
RB_19497678_L10_PA	fp10_41	10	41.314
FB_0027472_L10_PA	fp10_41	10	41.314
FB_0832349_L10_40_1	fp10_41	10	41.314
FB_0028109_L10_PA	fp10_41	10	41.314
RB_19503429_L10_PA	fp10_41	10	41.314
FB_0028116_L10_PA	fp10_41	10	41.314
FB_0028768_L10_PA	fp10_42	10	42.572
GD_00869_L10_PA	fp10_42	10	42.572
FB_0028790_L10_PA	fp10_42	10	42.572
RB_20058117_L10_PA	fp10_42	10	42.572
FEM_cg_19.bis	fp10_42	10	42.572
RB_20013910_L10_40_1	fp10_42	10	42.572
FB_0832819_L10_41_1	fp10_42	10	42.572
FB_0028767_L10_PA	fp10_42	10	42.572
FB_0029596_L10_PA	fp10_42	10	42.572
FB_0028786_L10_PA	fp10_42	10	42.572
FB_0029595_L10_PA	fp10_42	10	42.572
FB_0028766_L10_PA	fp10_42	10	42.572
FB_0028781_L10_PA	fp10_42	10	42.572

FEM_cg_19	fp10_42	10	42.572
FB_0028782_L10_PA	fp10_42	10	42.572
RB_20833228_L10_PA	fp10_42	10	42.572
RB_20813363_L10_PA	fp10_42	10	42.572
FB_0832820_L10_41_1	fp10_42	10	42.572
FEM_cg_9	fp10_42	10	42.572
FEM_cg_8	fp10_42	10	42.572
FB_0029600_L10_PA	fp10_42	10	42.572
FB_0029607_L10_PA	fp10_42	10	42.572
FB_0029606_L10_PA	fp10_42	10	42.572
FB_0832811_L10_41_1	fp10_42	10	42.572
FB_0029622_L10_PA	fp10_42	10	42.572
FB_0029621_L10_PA	fp10_42	10	42.572
FEM_cg_10	fp10_42	10	42.572
FEM_cg_11	fp10_42	10	42.572
FEM_cg_14	fp10_42	10	42.572
FEM_cg_18	fp10_42	10	42.572
FEM_cg_17	fp10_42	10	42.572
RB_20810437_L10_PA	fp10_42	10	42.572
FB_0832361_L10_40_1	fp10_42	10	42.572
FB_0029601_L10_PA	fp10_43	10	43.385
FB_0030341_L10_PA	fp10_43	10	43.385
RB_22521117_L10_PA	fp10_45	10	45.306
FB_0032577_L10_PA	fp10_45	10	45.306
FB_0036761_L10_PA	fp10_45	10	45.306
GD_01264_L10_44_1	fp10_45	10	45.306
GD_00812_L10_PA	fp10_45	10	45.306
RB_26641840_L10_44_1	fp10_45	10	45.306
FB_0036156_L10_PA	fp10_45	10	45.306
FB_0036161_L10_PA	fp10_45	10	45.306
FB_0036757_L10_PA	fp10_45	10	45.306
RB_26552010_L10_PA	fp10_45	10	45.306
FB_0833947_L10_43_1	fp10_45	10	45.306
RB_25256330_L10_PA	fp10_45	10	45.306
FB_0035181_L10_PA	fp10_45	10	45.306
FB_0036762_L10_PA	fp10_46	10	46.544
FB_0036756_L10_PA	fp10_46	10	46.544
RB_24245886_L10_PA	fp10_46	10	46.544
FB_0033468_L10_PA	fp10_46	10	46.544
FB_0034809_L10_PA	fp10_46	10	46.544
RB_26612165_L10_PA	fp10_46	10	46.544
RB_26616826_L10_PA	fp10_46	10	46.544
FB_0037831_L10_PA	fp10_46	10	46.544
FB_0037836_L10_PA	fp10_46	10	46.544
RB_27656951_L10_PA	fp10_46	10	46.544
FB_0036751_L10_PA	fp10_46	10	46.544

FB_0037837_L10_PA	fp10_46	10	46.544
RB_26614519_L10_PA	fp10_46	10	46.544
FB_0007370_L10_PA	fp10_46	10	46.544
FB_0007375_L10_PA	fp10_46	10	46.544
RB_22517441_L10_PA	fp10_46	10	46.544
FB_0031578_L10_PA	fp10_46	10	46.544
FB_0031585_L10_PA	fp10_46	10	46.544
FB_0832999_L10_41_1	fp10_46	10	46.544
FB_0007388_L10_PA	fp10_46	10	46.544
RB_22513868_L10_PA	fp10_46	10	46.544
FB_0036166_L10_PA	fp10_46	10	46.544
RB_26518569_L10_PA	fp10_46	10	46.544
FB_0036154_L10_PA	fp10_46	10	46.544
RB_26516335_L10_PA	fp10_46	10	46.544
FB_0035179_L10_PA	fp10_47	10	47.63
FB_0833943_L10_43_1	fp10_47	10	47.63
FB_0833948_L10_43_1	fp10_47	10	47.63
RB_23270798_L10_PA	fp10_47	10	47.63
FB_0037843_L10_PA	fp10_47	10	47.63
FB_0034098_L10_PA	fp10_47	10	47.63
FB_0834908_L10_45_1	fp10_47	10	47.63
FB_0037856_L10_PA	fp10_47	10	47.63
FB_0034085_L10_PA	fp10_47	10	47.63
GD_00971_L10_PA	fp10_47	10	47.63
FB_0032556_L10_PA	fp10_47	10	47.63
RB_25259266_L10_PA	fp10_47	10	47.63
RB_23285470_L10_PA	fp10_47	10	47.63
FB_0032582_L10_PA	fp10_47	10	47.63
FB_0034089_L10_PA	fp10_47	10	47.63
RB_28354787_L10_PA	fp10_47	10	47.63
RB_24273907_L10_PA	fp10_47	10	47.63
RB_23908228_L10_PA	fp10_47	10	47.63
RB_24294786_L10_PA	fp10_47	10	47.63
FB_0033008_L10_PA	fp10_47	10	47.63
FB_0034805_L10_PA	fp10_47	10	47.63
FB_0033445_L10_PA	fp10_47	10	47.63
FB_0033011_L10_PA	fp10_47	10	47.63
FB_0033462_L10_PA	fp10_47	10	47.63
FB_0032554_L10_PA	fp10_48	10	48.425
FB_0032300_L10_PA	fp10_48	10	48.425
RB_28610777_L10_45_1	fp10_48	10	48.425
RB_28354774_L10_PA	fp10_48	10	48.425
RB_28632998_L10_PA	fp10_48	10	48.425
RB_36033314_L10_49_1	fp10_49	10	49.682
RB_29031109_L10_PA	fp10_49	10	49.682
FB_0037842_L10_PA	fp10_49	10	49.682

RB_29213640_L10_PA	fp10_49	10	49.682
FB_0039713_L10_PA	fp10_49	10	49.682
RB_29044684_L10_PA	fp10_49	10	49.682
FB_0039071_L10_PA	fp10_49	10	49.682
FB_0040453_L10_PA	fp10_50	10	50.077
FB_0605153_L5_PA	fp10_50	10	50.077
FB_0039685_L10_PA	fp10_50	10	50.077
RB_29917745_L10_PA	fp10_50	10	50.077
RB_30206374_L10_PA	fp10_50	10	50.077
GD_00257_L10_PA	fp10_50	10	50.077
FB_0039053_L10_PA	fp10_50	10	50.077
FB_0040411_L10_PA	fp10_50	10	50.077
FB_0039680_L10_PA	fp10_50	10	50.077
FB_0605152_L5_PA	fp10_50	10	50.077
CONS61_L10_PA	fp10_50	10	50.077
FB_0040440_L10_PA	fp10_50	10	50.077
FB_0040406_L10_PA	fp10_50	10	50.077
GD_01570_L10_PA	fp10_50	10	50.077
FB_0039054_L10_PA	fp10_50	10	50.077
FB_0040425_L10_PA	fp10_50	10	50.077
FB_0040442_L10_PA	fp10_50	10	50.077
FB_0041076_L10_PA	fp10_50	10	50.077
FB_0040423_L10_PA	fp10_50	10	50.077
GD_00734_L10_PA	fp10_50	10	50.077
FB_0040410_L10_PA	fp10_50	10	50.077
FB_0835177_L10_45_1	fp10_50	10	50.077
FB_0040125_L10_PA	fp10_51	10	51.851
FB_0040123_L10_PA	fp10_51	10	51.851
RB_29020445_L10_PA	fp10_51	10	51.851
FB_0835532_L10_45_1	fp10_52	10	52.574
RB_29213692_L10_PA	fp10_52	10	52.574
RB_30223751_L10_PA	fp10_52	10	52.574
FB_0605151_L5_PA	fp10_52	10	52.574
FB_0040463_L10_PA	fp10_52	10	52.574
RB_31275344_L10_PA	fp10_52	10	52.574
RB_31320811_L10_PA	fp10_52	10	52.574
RB_30595771_L10_PA	fp10_52	10	52.574
FB_0042083_L10_PA	fp10_52	10	52.574
FB_0042078_L10_PA	fp10_52	10	52.574
FB_0605829_L5_PA	fp10_53	10	53.603
FB_0605818_L5_PA	fp10_53	10	53.603
FB_0605824_L5_PA	fp10_53	10	53.603
FB_0041084_L10_PA	fp10_53	10	53.603
FB_0041082_L10_PA	fp10_53	10	53.603
RB_31345666_L10_PA	fp10_53	10	53.603
FB_0041574_L10_PA	fp10_53	10	53.603

FB_0042076_L10_PA	fp10_53	10	53.603
FB_0042563_L10_PA	fp10_53	10	53.603
GD_01908_L10_PA	fp10_53	10	53.603
FB_0042077_L10_PA	fp10_53	10	53.603
FB_0042597_L10_PA	fp10_53	10	53.603
FB_0042567_L10_PA	fp10_53	10	53.603
FB_0042578_L10_PA	fp10_53	10	53.603
FB_0041573_L10_PA	fp10_53	10	53.603
FB_0041596_L10_PA	fp10_53	10	53.603
FB_0042584_L10_PA	fp10_53	10	53.603
FB_0041568_L10_PA	fp10_53	10	53.603
FB_0041587_L10_PA	fp10_53	10	53.603
FB_0041570_L10_PA	fp10_53	10	53.603
GD_01299_L10_PA	fp10_53	10	53.603
RB_31295437_L10_PA	fp10_56	10	56.386
RB_31279293_L10_PA	fp10_56	10	56.386
RB_31306024_L10_PA	fp10_57	10	57.484
RB_31331375_L10_PA	fp10_57	10	57.484
RB_30396941_L10_PA	fp10_57	10	57.484
GD_01961_L10_PA	fp10_58	10	58.561
FB_1054784_L10_69_2	fp10_59	10	59.752
FB_0043320_L10_PA	fp10_59	10	59.752
FB_0043322_L10_PA	fp10_59	10	59.752
FB_1054785_L10_69_2	fp10_59	10	59.752
FB_1054790_L10_69_2	fp10_59	10	59.752
RB_31676157_L10_PA	fp10_59	10	59.752
FB_0043319_L10_PA	fp10_59	10	59.752
RB_31707563_L10_PA	fp10_59	10	59.752
FB_1054768_L10_69_2	fp10_59	10	59.752
FB_1054781_L10_69_2	fp10_59	10	59.752
FB_0043325_L10_PA	fp10_59	10	59.752
FB_0043324_L10_PA	fp10_59	10	59.752
FB_1054774_L10_69_2	fp10_59	10	59.752
GD_00099_L10_PA	fp10_59	10	59.752
FB_0043317_L10_PA	fp10_59	10	59.752
FB_1054772_L10_69_2	fp10_59	10	59.752
FB_0046460_L10_PA	fp10_61	10	61.72
FB_0046458_L10_PA	fp10_61	10	61.72
FB_0044822_L10_PA	fp10_61	10	61.72
FB_0044418_L10_PA	fp10_62	10	62.469
FB_0044414_L10_PA	fp10_62	10	62.469
RB_32372997_L10_47_1	fp10_62	10	62.469
RB_32751652_L10_PA	fp10_62	10	62.469
RB_32402084_L10_47_1	fp10_62	10	62.469
FB_0044425_L10_PA	fp10_62	10	62.469
FB_0052480_L10_PA	fp10_62	10	62.469

FB_0045153_L10_PA	fp10_62	10	62.469
FB_0048115_L10_PA	fp10_63	10	63.416
FB_0837587_L10_48_1	fp10_63	10	63.416
RB_36071826_L10_PA	fp10_63	10	63.416
RB_33520271_L10_PA	fp10_63	10	63.416
FB_0049924_L10_PA	fp10_63	10	63.416
FB_0837574_L10_48_1	fp10_63	10	63.416
FB_0049857_L10_PA	fp10_63	10	63.416
FB_0837564_L10_48_1	fp10_63	10	63.416
FB_0045917_L10_PA	fp10_63	10	63.416
FB_0049856_L10_PA	fp10_63	10	63.416
FB_0049859_L10_PA	fp10_63	10	63.416
RB_36091067_L10_PA	fp10_63	10	63.416
RB_34936228_L10_48_1	fp10_63	10	63.416
RB_36066362_L10_PA	fp10_63	10	63.416
FB_0049741_L10_PA	fp10_63	10	63.416
FB_0048450_L10_PA	fp10_63	10	63.416
FB_0048123_L10_PA	fp10_63	10	63.416
FB_0049926_L10_PA	fp10_63	10	63.416
FB_0048104_L10_PA	fp10_63	10	63.416
RB_33506050_L10_PA	fp10_63	10	63.416
FB_0837576_L10_48_1	fp10_63	10	63.416
RB_33483733_L10_PA	fp10_63	10	63.416
FB_0049850_L10_PA	fp10_63	10	63.416
RB_36052300_L10_PA	fp10_63	10	63.416
FB_0837602_L10_48_1	fp10_63	10	63.416
FB_0049854_L10_PA	fp10_63	10	63.416
FB_0049851_L10_PA	fp10_63	10	63.416
FB_0837578_L10_48_1	fp10_63	10	63.416
FB_0048108_L10_PA	fp10_63	10	63.416
FB_0045158_L10_PA	fp10_64	10	64.846
FB_0048100_L10_PA	fp10_64	10	64.846
RB_36100460_L10_PA	fp10_64	10	64.846
FB_0045154_L10_PA	fp10_64	10	64.846
FB_0045914_L10_PA	fp10_64	10	64.846
RB_33501094_L10_PA	fp10_64	10	64.846
FB_0044803_L10_PA	fp10_64	10	64.846
RB_32730568_L10_PA	fp10_64	10	64.846
FB_0045926_L10_PA	fp10_64	10	64.846
FB_0049852_L10_PA	fp10_64	10	64.846
FB_0050205_L10_PA	fp10_65	10	65.78
RB_36985238_L10_PA	fp10_66	10	66.469
FB_0838432_L10_49_1	fp10_66	10	66.469
FB_0050201_L10_PA	fp10_67	10	67.619
FB_0050233_L10_PA	fp10_67	10	67.619
FB_0050215_L10_PA	fp10_67	10	67.619

FB_0051942_L10_PA	fp10_67	10	67.619
RB_36898184_L10_PA	fp10_67	10	67.619
RB_36883478_L10_PA	fp10_67	10	67.619
FB_0051414_L10_PA	fp10_67	10	67.619
FB_0050933_L10_PA	fp10_67	10	67.619
FB_0051422_L10_PA	fp10_67	10	67.619
FB_0050932_L10_PA	fp10_67	10	67.619
FB_0838443_L10_49_1	fp10_67	10	67.619
RB_36885663_L10_PA	fp10_67	10	67.619
FB_0838444_L10_49_1	fp10_67	10	67.619
FB_0050202_L10_PA	fp10_67	10	67.619
FB_0838423_L10_49_1	fp10_67	10	67.619
RB_36887888_L10_PA	fp10_67	10	67.619
RB_36853428_L10_PA	fp10_67	10	67.619
FEM_cg_4	fp10_67	10	67.619
FB_0050923_L10_PA	fp10_67	10	67.619
FB_0051759_L10_PA	fp10_68	10	68.667
RB_37031349_L10_PA	fp10_69	10	69.458
FB_0838244_L10_49_1	fp10_69	10	69.458
GD_01761_L10_PA	fp10_71	10	71.997
FB_0050921_L10_PA	fp10_71	10	71.997
FB_0050930_L10_PA	fp10_71	10	71.997
FB_0052472_L10_PA	fp10_73	10	73.404
FB_0051428_L10_PA	fp10_74	10	74.436
GD_00405_L10_PA	fp10_74	10	74.436
FEM_cg_1	fp10_74	10	74.436
FB_0051435_L10_PA	fp10_74	10	74.436
RB_36862797_L10_PA	fp10_74	10	74.436
FB_0051420_L10_PA	fp10_74	10	74.436
FB_0051751_L10_PA	fp10_74	10	74.436
FB_0051740_L10_PA	fp10_74	10	74.436
FB_0051416_L10_PA	fp10_74	10	74.436
RB_37072737_L10_PA	fp10_75	10	75.641
GD_00604_L10_PA	fp10_75	10	75.641
FB_0051784_L10_PA	fp10_75	10	75.641
FB_0051764_L10_PA	fp10_75	10	75.641
RB_37052690_L10_PA	fp10_75	10	75.641
FB_0052515_L10_PA	fp10_76	10	76.846
FB_0052503_L10_PA	fp10_76	10	76.846
FB_0052470_L10_PA	fp10_76	10	76.846
FB_0053638_L11_PA	fp11_00	11	0.11
FB_0054195_L11_PA	fp11_00	11	0.11
FB_0053704_L11_PA	fp11_00	11	0.11
FB_0053629_L11_PA	fp11_00	11	0.11
FB_0839403_L11_26_1	fp11_00	11	0.11
FB_0854043_L12_26_1	fp11_00	11	0.11

FB_0053694_L11_PA	fp11_00	11	0.11
FB_0054468_L11_PA	fp11_00	11	0.11
FB_0053634_L11_PA	fp11_00	11	0.11
FB_0054473_L11_PA	fp11_00	11	0.11
FB_0839408_L11_26_1	fp11_00	11	0.11
FB_0053621_L11_PA	fp11_00	11	0.11
FB_1055658_L11_48_2	fp11_00	11	0.11
FB_0054478_L11_PA	fp11_00	11	0.11
FB_0054474_L11_PA	fp11_00	11	0.11
FB_0054183_L11_PA	fp11_00	11	0.11
FB_0839399_L11_26_1	fp11_00	11	0.11
FB_0053698_L11_PA	fp11_00	11	0.11
FB_0839410_L11_26_1	fp11_00	11	0.11
FB_0854015_L12_26_1	fp11_00	11	0.11
FB_0054471_L11_PA	fp11_00	11	0.11
FB_0053679_L11_PA	fp11_00	11	0.11
FB_0839413_L11_26_1	fp11_00	11	0.11
FB_0305355_L15_PA	fp11_00	11	0.11
FB_0053695_L11_PA	fp11_00	11	0.11
FB_0305361_L15_PA	fp11_01	11	1.42
RB_557285_L11_PA	fp11_01	11	1.42
FB_1055746_L11_48_2	fp11_01	11	1.42
FB_1055755_L11_48_2	fp11_01	11	1.42
FB_0305354_L15_PA	fp11_01	11	1.42
FB_1055739_L11_48_2	fp11_01	11	1.42
FB_1055749_L11_48_2	fp11_01	11	1.42
FB_1055740_L11_48_2	fp11_01	11	1.42
FB_1107933_L11_64_3	fp11_01	11	1.42
FB_0054185_L11_PA	fp11_01	11	1.42
FB_0056178_L11_PA	fp11_01	11	1.42
FB_0054472_L11_PA	fp11_01	11	1.42
FB_0055541_L11_PA	fp11_01	11	1.42
FB_0055755_L11_PA	fp11_01	11	1.42
FB_0055550_L11_PA	fp11_01	11	1.42
FB_0055544_L11_PA	fp11_01	11	1.42
FB_1107931_L11_64_3	fp11_02	11	2.461
FB_1055750_L11_48_2	fp11_02	11	2.461
FB_0304732_L15_PA	fp11_02	11	2.461
FB_0248620_L14_PA	fp11_02	11	2.461
FB_0304721_L15_PA	fp11_02	11	2.461
FB_0304729_L15_PA	fp11_02	11	2.461
FB_0248621_L14_PA	fp11_02	11	2.461
FB_0910724_L15_62_1	fp11_02	11	2.461
FB_0056166_L11_PA	fp11_02	11	2.461
FB_0304737_L15_PA	fp11_02	11	2.461
FB_0056161_L11_PA	fp11_02	11	2.461

FB_0840090_L11_27_1	fp11_02	11	2.461
FB_0248609_L14_PA	fp11_02	11	2.461
FB_0840091_L11_27_1	fp11_02	11	2.461
FB_0840085_L11_27_1	fp11_02	11	2.461
FB_0055750_L11_PA	fp11_02	11	2.461
FB_0055549_L11_PA	fp11_02	11	2.461
FB_0055761_L11_PA	fp11_02	11	2.461
FB_0055753_L11_PA	fp11_03	11	3.418
FB_0055748_L11_PA	fp11_03	11	3.418
FB_0055546_L11_PA	fp11_03	11	3.418
FB_0840952_L11_29_1	fp11_03	11	3.418
FB_0059424_L11_PA	fp11_03	11	3.418
FB_0248626_L14_PA	fp11_03	11	3.418
FB_0248606_L14_PA	fp11_03	11	3.418
RB_3469064_L11_PA	fp11_03	11	3.418
FB_0057462_L11_PA	fp11_03	11	3.418
FB_0055543_L11_PA	fp11_03	11	3.418
FB_0057478_L11_PA	fp11_03	11	3.418
FB_0057451_L11_PA	fp11_03	11	3.418
FB_0057814_L11_PA	fp11_03	11	3.418
GD_02626_L11_PA	fp11_03	11	3.418
GD_01167_L11_PA	fp11_03	11	3.418
FB_0057795_L11_PA	fp11_03	11	3.418
FB_0057479_L11_PA	fp11_03	11	3.418
FB_0057797_L11_PA	fp11_03	11	3.418
RB_5021535_L11_PA	fp11_04	11	4.46
FB_0056171_L11_PA	fp11_04	11	4.46
FB_0058240_L11_PA	fp11_04	11	4.46
FB_0058227_L11_PA	fp11_04	11	4.46
FB_0058243_L11_PA	fp11_04	11	4.46
FB_0058248_L11_PA	fp11_04	11	4.46
FB_0058555_L11_PA	fp11_04	11	4.46
RB_4936280_L11_PA	fp11_04	11	4.46
RB_4936897_L11_PA	fp11_04	11	4.46
RB_5173582_L11_PA	fp11_04	11	4.46
RB_5054497_L11_PA	fp11_04	11	4.46
RB_5075367_L11_PA	fp11_04	11	4.46
FB_0060042_L11_PA	fp11_04	11	4.46
FB_0055745_L11_PA	fp11_04	11	4.46
FB_0058552_L11_PA	fp11_04	11	4.46
FB_0058557_L11_PA	fp11_04	11	4.46
FB_0058955_L11_PA	fp11_04	11	4.46
FB_0060047_L11_PA	fp11_05	11	5.099
FB_0059412_L11_PA	fp11_05	11	5.099
FB_0059426_L11_PA	fp11_05	11	5.099
RB_5159764_L11_PA	fp11_06	11	6.251

FB_0060049_L11_PA	fp11_06	11	6.251
FB_0059435_L11_PA	fp11_06	11	6.251
GD_01713_L11_PA	fp11_06	11	6.251
FB_0060045_L11_PA	fp11_06	11	6.251
GD_00418_L11_PA	fp11_06	11	6.251
FB_0060059_L11_PA	fp11_06	11	6.251
FB_0059434_L11_PA	fp11_06	11	6.251
RB_5208182_L11_PA	fp11_06	11	6.251
FB_0059438_L11_PA	fp11_06	11	6.251
FB_0059429_L11_PA	fp11_06	11	6.251
RB_5197434_L11_PA	fp11_06	11	6.251
RB_5944780_L11_29_1	fp11_06	11	6.251
FB_0063329_L11_PA	fp11_06	11	6.251
FB_0061193_L11_PA	fp11_06	11	6.251
RB_6496114_L11_29_1	fp11_06	11	6.251
FB_1056194_L11_51_2	fp11_06	11	6.251
FB_0061653_L11_PA	fp11_06	11	6.251
RB_6372814_L11_PA	fp11_06	11	6.251
RB_6372895_L11_PA	fp11_06	11	6.251
FB_0061189_L11_PA	fp11_07	11	7.534
RB_5482687_L11_PA	fp11_07	11	7.534
RB_1697921_L11_PA	fp11_07	11	7.534
FB_0840959_L11_29_1	fp11_07	11	7.534
FB_0059427_L11_PA	fp11_07	11	7.534
FB_0055551_L11_PA	fp11_07	11	7.534
FB_0057471_L11_PA	fp11_07	11	7.534
FB_0061949_L11_PA	fp11_07	11	7.534
GD_00621_L11_PA	fp11_07	11	7.534
FB_0061915_L11_PA	fp11_07	11	7.534
FB_0061962_L11_PA	fp11_07	11	7.534
FB_0061913_L11_PA	fp11_07	11	7.534
RB_7189032_L11_PA	fp11_09	11	9.081
FB_0062330_L11_PA	fp11_09	11	9.081
FB_0061923_L11_PA	fp11_09	11	9.081
FB_0645521_L6_PA	fp11_09	11	9.081
FB_0061658_L11_PA	fp11_09	11	9.081
RB_7143110_L11_PA	fp11_09	11	9.081
FB_0063317_L11_PA	fp11_09	11	9.081
FB_0062809_L11_PA	fp11_09	11	9.081
FB_0061654_L11_PA	fp11_09	11	9.081
FB_0062824_L11_PA	fp11_09	11	9.081
FB_0063314_L11_PA	fp11_09	11	9.081
FB_0062303_L11_PA	fp11_09	11	9.081
FB_0062333_L11_PA	fp11_09	11	9.081
FB_0061199_L11_PA	fp11_09	11	9.081
FB_0062307_L11_PA	fp11_09	11	9.081

FB_0061195_L11_PA	fp11_09	11	9.081
FB_0061661_L11_PA	fp11_09	11	9.081
FB_0063320_L11_PA	fp11_09	11	9.081
RB_6709034_L11_PA	fp11_09	11	9.081
FB_0841915_L11_29_1	fp11_09	11	9.081
FB_0062815_L11_PA	fp11_09	11	9.081
FB_0063321_L11_PA	fp11_10	11	10.367
FB_0063313_L11_PA	fp11_10	11	10.367
RB_8682607_L11_PA	fp11_10	11	10.367
FB_1056410_L11_51_2	fp11_10	11	10.367
FB_0064308_L11_PA	fp11_10	11	10.367
FB_0064020_L11_PA	fp11_10	11	10.367
RB_8707503_L11_PA	fp11_10	11	10.367
FB_0065004_L11_PA	fp11_10	11	10.367
FB_0842249_L11_29_1	fp11_10	11	10.367
GD_00735_L11_PA	fp11_10	11	10.367
FB_0065634_L11_PA	fp11_10	11	10.367
RB_8702100_L11_PA	fp11_10	11	10.367
FB_0064051_L11_PA	fp11_10	11	10.367
FB_0065019_L11_PA	fp11_10	11	10.367
FB_0065016_L11_PA	fp11_10	11	10.367
FB_0065876_L11_PA	fp11_10	11	10.367
FB_0065851_L11_PA	fp11_10	11	10.367
FB_0064300_L11_PA	fp11_10	11	10.367
FB_0065849_L11_PA	fp11_10	11	10.367
FB_0064987_L11_PA	fp11_10	11	10.367
FB_0064056_L11_PA	fp11_10	11	10.367
FB_0065872_L11_PA	fp11_10	11	10.367
FB_0064302_L11_PA	fp11_10	11	10.367
FB_0064309_L11_PA	fp11_10	11	10.367
FB_0064998_L11_PA	fp11_10	11	10.367
FB_0064023_L11_PA	fp11_10	11	10.367
FB_0065847_L11_PA	fp11_10	11	10.367
FB_0065857_L11_PA	fp11_10	11	10.367
FB_0065631_L11_PA	fp11_10	11	10.367
FB_0064032_L11_PA	fp11_10	11	10.367
FB_0062335_L11_PA	fp11_10	11	10.367
FB_0065015_L11_PA	fp11_10	11	10.367
RB_6704249_L11_PA	fp11_10	11	10.367
FB_0061914_L11_PA	fp11_10	11	10.367
RB_9976495_L11_PA	fp11_11	11	11.555
FB_0065614_L11_PA	fp11_11	11	11.555
FB_0065622_L11_PA	fp11_11	11	11.555
FB_1056519_L11_51_2	fp11_11	11	11.555
FB_0065620_L11_PA	fp11_11	11	11.555
RB_6697510_L11_PA	fp11_11	11	11.555

FB_0841914_L11_29_1	fp11_11	11	11.555
RB_12380727_L11_PA	fp11_12	11	12.376
FB_0063327_L11_PA	fp11_12	11	12.376
RB_8694610_L11_PA	fp11_12	11	12.376
FB_0065012_L11_PA	fp11_12	11	12.376
RB_10597805_L11_PA	fp11_13	11	13.563
FB_0067274_L11_PA	fp11_13	11	13.563
FB_0067276_L11_PA	fp11_13	11	13.563
FB_0842254_L11_29_1	fp11_13	11	13.563
FB_1096673_L6_48_2	fp11_13	11	13.563
FB_0063325_L11_PA	fp11_13	11	13.563
FB_0066493_L11_PA	fp11_15	11	15.409
RB_9965260_L11_PA	fp11_15	11	15.409
FB_0066483_L11_PA	fp11_15	11	15.409
FB_1096674_L6_48_2	fp11_15	11	15.409
FB_0066494_L11_PA	fp11_15	11	15.409
FB_1096672_L6_48_2	fp11_15	11	15.409
FB_0066491_L11_PA	fp11_15	11	15.409
FB_0034812_L10_PA	fp11_15	11	15.409
FB_1056723_L11_53_2	fp11_15	11	15.409
FB_0067281_L11_PA	fp11_15	11	15.409
RB_11306023_L11_PA	fp11_16	11	16.484
RB_11309307_L11_PA	fp11_16	11	16.484
FB_0067279_L11_PA	fp11_16	11	16.484
RB_11608827_L11_PA	fp11_16	11	16.484
FB_0067989_L11_PA	fp11_17	11	17.353
GD_00254_L11_PA	fp11_17	11	17.353
RB_10708802_L11_PA	fp11_17	11	17.353
FB_0067825_L11_PA	fp11_17	11	17.353
FB_0067824_L11_PA	fp11_17	11	17.353
FB_0067812_L11_PA	fp11_17	11	17.353
FB_0067823_L11_PA	fp11_17	11	17.353
RB_10565787_L11_PA	fp11_17	11	17.353
FB_0068414_L11_PA	fp11_17	11	17.353
RB_12148665_L11_PA	fp11_17	11	17.353
FB_1056718_L11_53_2	fp11_17	11	17.353
FB_0430670_L1_PA	fp11_17	11	17.353
FB_0067993_L11_PA	fp11_17	11	17.353
FB_0069360_L11_PA	fp11_17	11	17.353
RB_11831569_L11_PA	fp11_17	11	17.353
FB_0067998_L11_PA	fp11_18	11	18.314
FB_0068398_L11_PA	fp11_18	11	18.314
FB_0069361_L11_PA	fp11_18	11	18.314
FB_0068007_L11_PA	fp11_18	11	18.314
RB_11573565_L11_PA	fp11_18	11	18.314
RB_11595996_L11_PA	fp11_18	11	18.314

GD_00377_L11_PA	fp11_18	11	18.314
FB_0068004_L11_PA	fp11_18	11	18.314
RB_12169230_L11_PA	fp11_18	11	18.314
FB_0068415_L11_PA	fp11_18	11	18.314
FB_0067999_L11_PA	fp11_18	11	18.314
RB_11824111_L11_PA	fp11_18	11	18.314
FB_1056905_L11_53_2	fp11_19	11	19.092
RB_12142492_L11_PA	fp11_19	11	19.092
GD_00240_L11_PA	fp11_20	11	20.592
FB_0069370_L11_PA	fp11_20	11	20.592
FB_0069352_L11_PA	fp11_20	11	20.592
FB_1056907_L11_53_2	fp11_20	11	20.592
FB_0069376_L11_PA	fp11_20	11	20.592
FB_1056909_L11_53_2	fp11_20	11	20.592
RB_12150935_L11_PA	fp11_20	11	20.592
FB_0844561_L11_31_1	fp11_20	11	20.592
FB_0070710_L11_PA	fp11_20	11	20.592
GD_00037_L11_PA	fp11_20	11	20.592
FB_1057011_L11_53_2	fp11_20	11	20.592
FB_1057003_L11_53_2	fp11_20	11	20.592
FB_0070724_L11_PA	fp11_20	11	20.592
RB_12352575_L11_PA	fp11_20	11	20.592
FB_0070718_L11_PA	fp11_20	11	20.592
FB_1056990_L11_53_2	fp11_20	11	20.592
FB_1057021_L11_53_2	fp11_20	11	20.592
FB_0069369_L11_PA	fp11_20	11	20.592
RB_12349819_L11_PA	fp11_20	11	20.592
FB_1056992_L11_53_2	fp11_20	11	20.592
GD_02465_L11_PA	fp11_20	11	20.592
GD_00441_L11_PA	fp11_20	11	20.592
RB_12934511_L11_PA	fp11_20	11	20.592
FB_0071391_L11_PA	fp11_20	11	20.592
FB_0071392_L11_PA	fp11_20	11	20.592
RB_12328377_L11_PA	fp11_20	11	20.592
FB_0075139_L11_PA	fp11_21	11	21.585
FB_0069354_L11_PA	fp11_21	11	21.585
FB_0071387_L11_PA	fp11_21	11	21.585
FB_0074720_L11_PA	fp11_21	11	21.585
RB_12911499_L11_31_1	fp11_21	11	21.585
FB_0072017_L11_PA	fp11_21	11	21.585
FB_0075421_L11_PA	fp11_21	11	21.585
RB_15738487_L11_PA	fp11_21	11	21.585
FB_0071995_L11_PA	fp11_21	11	21.585
FB_0075140_L11_PA	fp11_21	11	21.585
FB_0075427_L11_PA	fp11_21	11	21.585
FB_0074221_L11_PA	fp11_22	11	22.483

FB_0073626_L11_PA	fp11_22	11	22.483
FB_1057083_L11_54_2	fp11_22	11	22.483
RB_14850427_L11_PA	fp11_22	11	22.483
RB_12309737_L11_PA	fp11_22	11	22.483
FB_0877968_L13_40_1	fp11_22	11	22.483
FB_0193334_L13_PA	fp11_23	11	23.2
FB_0071388_L11_PA	fp11_25	11	25.64
FB_0877955_L13_40_1	fp11_26	11	26.648
FB_0940200_L1_42_1	fp11_26	11	26.648
FB_0072297_L11_PA	fp11_26	11	26.648
RB_13655380_L11_PA	fp11_26	11	26.648
FB_0072302_L11_PA	fp11_26	11	26.648
FB_0939858_L1_42_1	fp11_26	11	26.648
FB_0072298_L11_PA	fp11_26	11	26.648
FB_0940194_L1_42_1	fp11_26	11	26.648
FB_0072604_L11_PA	fp11_26	11	26.648
FB_0072603_L11_PA	fp11_26	11	26.648
FB_0845056_L11_32_1	fp11_26	11	26.648
FB_0072612_L11_PA	fp11_26	11	26.648
FB_0194114_L13_PA	fp11_27	11	27.457
FB_1098476_L7_49_2	fp11_28	11	28.6
FB_0654293_L6_PA	fp11_28	11	28.6
FB_0489514_L2_PA	fp11_28	11	28.6
FB_0111923_L12_PA	fp11_28	11	28.6
RB_22723616_L11_PA	fp11_28	11	28.6
FB_0855587_L12_27_1	fp11_28	11	28.6
RB_22729723_L11_PA	fp11_28	11	28.6
FB_0855601_L12_27_1	fp11_28	11	28.6
FB_0081375_L11_PA	fp11_28	11	28.6
FB_0077297_L11_PA	fp11_28	11	28.6
FB_0077308_L11_PA	fp11_28	11	28.6
FB_0193353_L13_PA	fp11_28	11	28.6
FB_0193335_L13_PA	fp11_28	11	28.6
FB_0193332_L13_PA	fp11_28	11	28.6
FB_0078678_L11_PA	fp11_29	11	29.95
FB_0855598_L12_27_1	fp11_29	11	29.95
FB_0077309_L11_PA	fp11_29	11	29.95
FB_0078000_L11_PA	fp11_29	11	29.95
FB_0845784_L11_33_1	fp11_29	11	29.95
FB_0077776_L11_PA	fp11_29	11	29.95
FB_0077786_L11_PA	fp11_29	11	29.95
FB_0939149_L1_42_1	fp11_29	11	29.95
FB_0308692_L15_PA	fp11_29	11	29.95
FB_0078313_L11_PA	fp11_29	11	29.95
GD_01187_L11_PA	fp11_29	11	29.95
FB_0996783_L5_44_1	fp11_29	11	29.95

FB_0077774_L11_PA	fp11_29	11	29.95
FB_0076552_L11_PA	fp11_29	11	29.95
FB_0078695_L11_PA	fp11_29	11	29.95
FB_0996803_L5_44_1	fp11_29	11	29.95
FB_0077291_L11_PA	fp11_29	11	29.95
FB_0078309_L11_PA	fp11_29	11	29.95
FB_0078328_L11_PA	fp11_29	11	29.95
FB_0077287_L11_PA	fp11_29	11	29.95
FB_0077315_L11_PA	fp11_29	11	29.95
FB_0996804_L5_44_1	fp11_29	11	29.95
FB_0078014_L11_PA	fp11_29	11	29.95
FB_0081115_L11_PA	fp11_29	11	29.95
FB_1098471_L7_49_2	fp11_29	11	29.95
FB_0078684_L11_PA	fp11_29	11	29.95
FB_0078311_L11_PA	fp11_29	11	29.95
FB_0078002_L11_PA	fp11_29	11	29.95
RB_16672448_L11_PA	fp11_29	11	29.95
FB_0078026_L11_PA	fp11_29	11	29.95
FB_0076567_L11_PA	fp11_29	11	29.95
FB_0077779_L11_PA	fp11_29	11	29.95
FB_0077314_L11_PA	fp11_29	11	29.95
FB_1098464_L7_49_2	fp11_29	11	29.95
FB_0077294_L11_PA	fp11_29	11	29.95
FB_0076551_L11_PA	fp11_29	11	29.95
FB_0077770_L11_PA	fp11_29	11	29.95
FB_0078003_L11_PA	fp11_29	11	29.95
FB_0076561_L11_PA	fp11_29	11	29.95
FB_0077750_L11_PA	fp11_29	11	29.95
FB_0078778_L11_PA	fp11_29	11	29.95
FB_0078327_L11_PA	fp11_29	11	29.95
FB_1104685_L9_39_2	fp11_29	11	29.95
FB_0077303_L11_PA	fp11_29	11	29.95
FB_0083299_L11_PA	fp11_30	11	30.6
RB_19384185_L11_PA	fp11_30	11	30.6
FB_0077916_L11_PA	fp11_30	11	30.6
FB_0078768_L11_PA	fp11_30	11	30.6
FB_0078779_L11_PA	fp11_30	11	30.6
FB_0855591_L12_27_1	fp11_30	11	30.6
FB_0078776_L11_PA	fp11_30	11	30.6
FB_0111922_L12_PA	fp11_30	11	30.6
FB_1104688_L9_39_2	fp11_30	11	30.6
FB_0855586_L12_27_1	fp11_30	11	30.6
RB_19378044_L11_PA	fp11_30	11	30.6
GD_01878_L11_PA	fp11_30	11	30.6
FB_0111924_L12_PA	fp11_30	11	30.6
RB_23881186_L11_PA	fp11_31	11	31.361

FB_0077928_L11_PA	fp11_31	11	31.361
FB_0077938_L11_PA	fp11_31	11	31.361
FB_0111925_L12_PA	fp11_31	11	31.361
FB_0077909_L11_PA	fp11_31	11	31.361
FB_0083301_L11_PA	fp11_31	11	31.361
GD_10010_L11_PA	fp11_31	11	31.361
FB_0077930_L11_PA	fp11_31	11	31.361
FB_0654291_L6_PA	fp11_31	11	31.361
FB_0077913_L11_PA	fp11_31	11	31.361
FB_0081952_L11_PA	fp11_31	11	31.361
FB_0489251_L2_PA	fp11_31	11	31.361
FB_0078690_L11_PA	fp11_31	11	31.361
FB_0847269_L11_40_1	fp11_32	11	32.602
FB_0083456_L11_PA	fp11_32	11	32.602
FB_0855597_L12_27_1	fp11_32	11	32.602
FB_0489250_L2_PA	fp11_33	11	33.216
FB_0081348_L11_PA	fp11_33	11	33.216
FB_0489517_L2_PA	fp11_33	11	33.216
FB_0081954_L11_PA	fp11_33	11	33.216
FB_0489282_L2_PA	fp11_33	11	33.216
FB_0489530_L2_PA	fp11_33	11	33.216
FB_0489260_L2_PA	fp11_33	11	33.216
FB_0081979_L11_PA	fp11_33	11	33.216
GD_00743_L11_PA	fp11_33	11	33.216
FB_0081965_L11_PA	fp11_33	11	33.216
FB_0081364_L11_PA	fp11_33	11	33.216
FB_0489265_L2_PA	fp11_33	11	33.216
FB_0489266_L2_PA	fp11_33	11	33.216
RB_22759608_L11_PA	fp11_33	11	33.216
FB_0081976_L11_PA	fp11_33	11	33.216
FB_0081967_L11_PA	fp11_34	11	34.264
FB_0488798_L2_PA	fp11_34	11	34.264
FB_0725507_L8_PA	fp11_34	11	34.264
FB_1085936_L2_50_2	fp11_34	11	34.264
FB_0725494_L8_PA	fp11_34	11	34.264
FB_1085938_L2_50_2	fp11_34	11	34.264
FB_1085951_L2_50_2	fp11_34	11	34.264
FB_0488795_L2_PA	fp11_34	11	34.264
FB_0488794_L2_PA	fp11_34	11	34.264
FB_1085947_L2_50_2	fp11_34	11	34.264
FB_0083833_L11_PA	fp11_34	11	34.264
FB_0847958_L11_41_1	fp11_35	11	35.451
FB_0083829_L11_PA	fp11_35	11	35.451
FB_0084534_L11_PA	fp11_35	11	35.451
FB_0083448_L11_PA	fp11_35	11	35.451
FB_0084571_L11_PA	fp11_35	11	35.451

FB_0083429_L11_PA	fp11_35	11	35.451
FB_0288672_L15_PA	fp11_35	11	35.451
FB_0083444_L11_PA	fp11_35	11	35.451
FB_0083452_L11_PA	fp11_35	11	35.451
FB_0083455_L11_PA	fp11_35	11	35.451
FB_0083822_L11_PA	fp11_35	11	35.451
FB_0083830_L11_PA	fp11_35	11	35.451
FB_0085106_L11_PA	fp11_35	11	35.451
RB_24586716_L11_PA	fp11_36	11	36.376
FB_0848298_L11_41_1	fp11_37	11	37.357
FB_0083820_L11_PA	fp11_37	11	37.357
FB_0848270_L11_41_1	fp11_37	11	37.357
FB_0847944_L11_41_1	fp11_37	11	37.357
FB_0085102_L11_PA	fp11_37	11	37.357
FB_0848258_L11_41_1	fp11_37	11	37.357
FB_0083436_L11_PA	fp11_37	11	37.357
FB_0083839_L11_PA	fp11_37	11	37.357
FB_1072883_L15_87_2	fp11_37	11	37.357
FB_0288676_L15_PA	fp11_37	11	37.357
FB_0848306_L11_41_1	fp11_37	11	37.357
FB_0083819_L11_PA	fp11_37	11	37.357
FB_0083446_L11_PA	fp11_37	11	37.357
FB_1072865_L15_87_2	fp11_37	11	37.357
FB_0848307_L11_41_1	fp11_37	11	37.357
GD_01797_L11_PA	fp11_37	11	37.357
FB_0085446_L11_PA	fp11_37	11	37.357
FB_0086582_L11_PA	fp11_37	11	37.357
FB_0085429_L11_PA	fp11_37	11	37.357
MDP000017222	fp11_38	11	38.482
FB_0085428_L11_PA	fp11_38	11	38.482
FB_0086581_L11_PA	fp11_38	11	38.482
FB_0085444_L11_PA	fp11_38	11	38.482
FB_0848252_L11_41_1	fp11_38	11	38.482
FB_0086598_L11_PA	fp11_38	11	38.482
FB_0085425_L11_PA	fp11_38	11	38.482
RB_26026377_L11_PA	fp11_38	11	38.482
GD_01857_L11_41_1	fp11_38	11	38.482
FB_0087006_L11_PA	fp11_39	11	39.518
FB_0087003_L11_PA	fp11_39	11	39.518
FB_0085424_L11_PA	fp11_39	11	39.518
FB_0086989_L11_PA	fp11_39	11	39.518
RB_26099970_L11_41_1	fp11_39	11	39.518
FB_0086996_L11_PA	fp11_39	11	39.518
FB_0087944_L11_PA	fp11_39	11	39.518
RB_26829691_L11_PA	fp11_39	11	39.518
FB_0087723_L11_PA	fp11_39	11	39.518

FB_0087720_L11_PA	fp11_39	11	39.518
FB_0087920_L11_PA	fp11_39	11	39.518
FB_0087917_L11_PA	fp11_39	11	39.518
GD_01361_L11_PA	fp11_39	11	39.518
FB_0087919_L11_PA	fp11_39	11	39.518
RB_26810795_L11_PA	fp11_39	11	39.518
FB_0087922_L11_PA	fp11_39	11	39.518
FB_0849347_L11_41_1	fp11_39	11	39.518
FB_0087914_L11_PA	fp11_39	11	39.518
FB_0087928_L11_PA	fp11_39	11	39.518
FB_0089958_L11_PA	fp11_40	11	40.286
FB_0089267_L11_PA	fp11_40	11	40.286
FB_0849822_L11_42_1	fp11_40	11	40.286
FB_0092266_L11_PA	fp11_40	11	40.286
FB_0227164_L14_PA	fp11_40	11	40.286
FB_0849835_L11_42_1	fp11_40	11	40.286
FB_1017320_L7_39_1	fp11_40	11	40.286
FB_0089188_L11_PA	fp11_40	11	40.286
FB_0089920_L11_PA	fp11_40	11	40.286
FB_0087722_L11_PA	fp11_40	11	40.286
FB_0089263_L11_PA	fp11_40	11	40.286
FB_1017315_L7_39_1	fp11_40	11	40.286
FB_0089190_L11_PA	fp11_40	11	40.286
FB_0086583_L11_PA	fp11_40	11	40.286
FB_1108422_L11_72_3	fp11_40	11	40.286
FB_0706064_L7_PA	fp11_40	11	40.286
FB_1108431_L11_72_3	fp11_40	11	40.286
FB_0706061_L7_PA	fp11_40	11	40.286
FB_1108428_L11_72_3	fp11_40	11	40.286
GD_01705_L11_PA	fp11_40	11	40.286
FB_0850158_L11_42_1	fp11_40	11	40.286
FB_0849825_L11_42_1	fp11_40	11	40.286
FB_0089936_L11_PA	fp11_40	11	40.286
FB_0090678_L11_PA	fp11_40	11	40.286
FB_0089928_L11_PA	fp11_40	11	40.286
FB_1108418_L11_72_3	fp11_40	11	40.286
FB_1108415_L11_72_3	fp11_40	11	40.286
FB_1017340_L7_39_1	fp11_40	11	40.286
FB_0849828_L11_42_1	fp11_40	11	40.286
FB_1108435_L11_72_3	fp11_40	11	40.286
FB_0089940_L11_PA	fp11_40	11	40.286
FB_0089947_L11_PA	fp11_40	11	40.286
FB_0086596_L11_PA	fp11_40	11	40.286
FB_1017327_L7_39_1	fp11_40	11	40.286
RB_28699805_L11_PA	fp11_40	11	40.286
FB_0089272_L11_PA	fp11_40	11	40.286

FB_0706050_L7_PA	fp11_40	11	40.286
FB_0849815_L11_42_1	fp11_40	11	40.286
FB_0089189_L11_PA	fp11_40	11	40.286
FB_1017322_L7_39_1	fp11_40	11	40.286
FB_0089250_L11_PA	fp11_40	11	40.286
FB_0849813_L11_42_1	fp11_40	11	40.286
FB_0850200_L11_42_1	fp11_40	11	40.286
FB_0850166_L11_42_1	fp11_40	11	40.286
FB_0850156_L11_42_1	fp11_40	11	40.286
FB_0090683_L11_PA	fp11_40	11	40.286
FB_1017317_L7_39_1	fp11_40	11	40.286
RB_28752026_L11_PA	fp11_40	11	40.286
GD_00662_L11_PA	fp11_41	11	41.534
FB_0850192_L11_42_1	fp11_41	11	41.534
FB_0089923_L11_PA	fp11_41	11	41.534
FB_1108425_L11_72_3	fp11_41	11	41.534
FB_0227161_L14_PA	fp11_41	11	41.534
FB_0227162_L14_PA	fp11_41	11	41.534
FB_0227148_L14_PA	fp11_41	11	41.534
FB_0091446_L11_PA	fp11_41	11	41.534
FB_0094426_L11_PA	fp11_41	11	41.534
RB_29463163_L11_PA	fp11_42	11	42.127
FB_0226687_L14_PA	fp11_42	11	42.127
FB_0226677_L14_PA	fp11_42	11	42.127
FB_0090677_L11_PA	fp11_42	11	42.127
FB_0090657_L11_PA	fp11_42	11	42.127
FB_0092278_L11_PA	fp11_43	11	43.986
FB_0094479_L11_PA	fp11_43	11	43.986
FB_0226663_L14_PA	fp11_43	11	43.986
RB_30589564_L11_PA	fp11_43	11	43.986
FB_0091430_L11_PA	fp11_43	11	43.986
RB_29832087_L11_PA	fp11_43	11	43.986
FB_0091425_L11_PA	fp11_43	11	43.986
FB_0227149_L14_PA	fp11_43	11	43.986
FB_0092079_L11_PA	fp11_43	11	43.986
FB_0226670_L14_PA	fp11_43	11	43.986
FB_0092280_L11_PA	fp11_43	11	43.986
FB_0094434_L11_PA	fp11_45	11	45.838
FB_0094463_L11_PA	fp11_45	11	45.838
FB_1058256_L11_59_2	fp11_46	11	46.448
FB_0092083_L11_PA	fp11_46	11	46.448
FB_0095139_L11_PA	fp11_47	11	47.058
RB_32934800_L11_PA	fp11_48	11	48.688
FB_0095759_L11_PA	fp11_48	11	48.688
GD_00647_L11_PA	fp11_48	11	48.688
FB_0095750_L11_PA	fp11_48	11	48.688

FB_0096420_L11_PA	fp11_48	11	48.688
FB_0096419_L11_PA	fp11_48	11	48.688
FB_0096437_L11_PA	fp11_48	11	48.688
FB_0095755_L11_PA	fp11_48	11	48.688
GD_00566_L11_PA	fp11_48	11	48.688
FB_1108494_L11_72_3	fp11_48	11	48.688
FB_1108506_L11_72_3	fp11_48	11	48.688
FB_0096440_L11_PA	fp11_48	11	48.688
FB_0096426_L11_PA	fp11_48	11	48.688
RB_32553307_L11_PA	fp11_48	11	48.688
FB_1108488_L11_72_3	fp11_48	11	48.688
FB_1108493_L11_72_3	fp11_48	11	48.688
FB_1108495_L11_72_3	fp11_49	11	49.616
FB_0851592_L11_44_1	fp11_50	11	50.679
FB_0097090_L11_PA	fp11_50	11	50.679
FB_0097116_L11_PA	fp11_50	11	50.679
FB_0851591_L11_44_1	fp11_50	11	50.679
FB_0096742_L11_PA	fp11_50	11	50.679
FB_0851595_L11_44_1	fp11_50	11	50.679
RB_33467558_L11_PA	fp11_50	11	50.679
FB_0097096_L11_PA	fp11_50	11	50.679
FB_0851593_L11_44_1	fp11_50	11	50.679
FB_0097092_L11_PA	fp11_50	11	50.679
RB_33482482_L11_PA	fp11_50	11	50.679
GD_02576_L11_PA	fp11_50	11	50.679
FB_0851658_L11_44_1	fp11_55	11	55.1
RB_34213532_L11_PA	fp11_55	11	55.1
RB_34210655_L11_PA	fp11_55	11	55.1
FB_0851669_L11_44_1	fp11_55	11	55.1
FB_0098389_L11_PA	fp11_55	11	55.1
RB_35954489_L11_PA	fp11_55	11	55.1
FB_0098934_L11_PA	fp11_55	11	55.1
FB_0216941_L14_PA	fp11_55	11	55.1
FB_0216939_L14_PA	fp11_55	11	55.1
RB_35958135_L11_PA	fp11_55	11	55.1
RB_35850734_L11_PA	fp11_55	11	55.1
RB_35967392_L11_PA	fp11_55	11	55.1
FB_0098378_L11_PA	fp11_55	11	55.1
FB_0098361_L11_PA	fp11_55	11	55.1
FB_0100849_L11_PA	fp11_56	11	56.889
FB_0216925_L14_PA	fp11_56	11	56.889
FB_0098382_L11_PA	fp11_56	11	56.889
FB_0216929_L14_PA	fp11_56	11	56.889
FB_0099345_L11_PA	fp11_56	11	56.889
FB_0216949_L14_PA	fp11_56	11	56.889
FB_0099335_L11_PA	fp11_56	11	56.889

FB_0099339_L11_PA	fp11_57	11	57.478
RB_35803279_L11_PA	fp11_57	11	57.478
RB_36008454_L11_PA	fp11_59	11	59.377
FB_0852584_L11_45_1	fp11_59	11	59.377
FB_0101355_L11_PA	fp11_59	11	59.377
FB_0852596_L11_45_1	fp11_59	11	59.377
FB_0101336_L11_PA	fp11_59	11	59.377
RB_35853497_L11_PA	fp11_59	11	59.377
FB_0103149_L11_PA	fp11_60	11	60.86
FB_0852912_L11_46_1	fp11_61	11	61.922
FB_0102214_L11_PA	fp11_61	11	61.922
FB_0102221_L11_PA	fp11_61	11	61.922
RB_36927967_L11_PA	fp11_61	11	61.922
RB_36931755_L11_PA	fp11_62	11	62.382
FB_0104745_L11_PA	fp11_62	11	62.382
FB_0105096_L11_PA	fp11_63	11	63.813
FB_0853706_L11_47_1	fp11_63	11	63.813
FB_0105098_L11_PA	fp11_63	11	63.813
FB_0105100_L11_PA	fp11_63	11	63.813
FB_0105104_L11_PA	fp11_63	11	63.813
FB_0105088_L11_PA	fp11_63	11	63.813
FB_0853712_L11_47_1	fp11_63	11	63.813
FB_0105116_L11_PA	fp11_63	11	63.813
FB_0853797_L11_47_1	fp11_63	11	63.813
FB_0104764_L11_PA	fp11_64	11	64.473
FB_0104746_L11_PA	fp11_64	11	64.473
FB_0104743_L11_PA	fp11_64	11	64.473
FB_0137132_L12_PA	fp11_64	11	64.473
FB_0853704_L11_47_1	fp11_64	11	64.473
FB_0105095_L11_PA	fp11_64	11	64.473
FB_0106092_L11_PA	fp11_65	11	65.343
GD_00182_L11_PA	fp11_65	11	65.343
FB_0710937_L7_PA	fp11_65	11	65.343
FB_0106675_L11_PA	fp11_65	11	65.343
FB_0710915_L7_PA	fp11_65	11	65.343
FB_0106108_L11_PA	fp11_65	11	65.343
FB_0106668_L11_PA	fp11_65	11	65.343
FB_0106088_L11_PA	fp11_65	11	65.343
FB_0105552_L11_PA	fp11_65	11	65.343
FB_0104753_L11_PA	fp11_65	11	65.343
FB_0106090_L11_PA	fp11_65	11	65.343
FB_0853956_L11_47_1	fp11_65	11	65.343
FB_0853955_L11_47_1	fp11_65	11	65.343
FB_0853820_L11_47_1	fp11_65	11	65.343
FB_0853819_L11_47_1	fp11_65	11	65.343
FB_0106666_L11_PA	fp11_66	11	66.153

FB_0105555_L11_PA	fp11_66	11	66.153
FB_0105550_L11_PA	fp11_66	11	66.153
FB_0852908_L11_46_1	fp11_66	11	66.153
RB_36934343_L11_PA	fp11_66	11	66.153
FB_0105122_L11_PA	fp11_66	11	66.153
FB_0853709_L11_47_1	fp11_66	11	66.153
FB_0106095_L11_PA	fp11_66	11	66.153
FB_0852930_L11_46_1	fp11_66	11	66.153
FB_0103141_L11_PA	fp11_66	11	66.153
FB_0103138_L11_PA	fp11_66	11	66.153
RB_39190659_L11_PA	fp11_66	11	66.153
FB_0852911_L11_46_1	fp11_66	11	66.153
RB_38742272_L11_PA	fp11_66	11	66.153
FB_0852923_L11_46_1	fp11_66	11	66.153
FB_0104752_L11_PA	fp11_66	11	66.153
FB_0105560_L11_PA	fp11_66	11	66.153
RB_39149329_L11_PA	fp11_66	11	66.153
FB_0105553_L11_PA	fp11_66	11	66.153
FB_0852913_L11_46_1	fp11_66	11	66.153
RB_38347479_L11_PA	fp11_66	11	66.153
FB_0105556_L11_PA	fp11_66	11	66.153
FB_0106133_L11_PA	fp11_66	11	66.153
FB_0105101_L11_PA	fp11_66	11	66.153
FB_0106671_L11_PA	fp11_66	11	66.153
FB_0853817_L11_47_1	fp11_66	11	66.153
RB_38732054_L11_PA	fp11_66	11	66.153
FB_0106115_L11_PA	fp11_66	11	66.153
RB_38702850_L11_PA	fp11_66	11	66.153
RB_38694306_L11_PA	fp11_66	11	66.153
FB_0102218_L11_PA	fp11_66	11	66.153
FB_0106112_L11_PA	fp11_66	11	66.153
FB_0106099_L11_PA	fp11_66	11	66.153
RB_40016890_L11_PA	fp11_66	11	66.153
RB_39786717_L11_PA	fp11_66	11	66.153
RB_38697189_L11_PA	fp11_66	11	66.153
FB_0103991_L11_PA	fp11_66	11	66.153
FB_0104755_L11_PA	fp11_66	11	66.153
FB_0103160_L11_PA	fp11_67	11	67.355
FB_0104751_L11_PA	fp11_67	11	67.355
RB_40025534_L11_PA	fp11_67	11	67.355
RB_40056015_L11_PA	fp11_67	11	67.355
FB_0853804_L11_47_1	fp11_67	11	67.355
RB_39914390_L11_PA	fp11_68	11	68.461
RB_39914254_L11_PA	fp11_68	11	68.461
FB_0710911_L7_PA	fp11_69	11	69.666
RB_40045235_L11_PA	fp11_69	11	69.666

FB_0710908_L7_PA	fp11_69	11	69.666
FB_0107064_L11_PA	fp11_69	11	69.666
RB_494465_L12_PA	fp12_00	12	0.612
RB_500084_L12_PA	fp12_00	12	0.612
RB_31831_L12_PA	fp12_00	12	0.612
RB_22208_L12_PA	fp12_00	12	0.612
RB_473947_L12_PA	fp12_00	12	0.612
FB_0107522_L12_PA	fp12_00	12	0.612
FB_0107532_L12_PA	fp12_00	12	0.612
FB_0107523_L12_PA	fp12_00	12	0.612
FB_0107535_L12_PA	fp12_00	12	0.612
FB_0109509_L12_PA	fp12_00	12	0.612
FB_0107999_L12_PA	fp12_00	12	0.612
FB_0107529_L12_PA	fp12_00	12	0.612
FB_0110360_L12_PA	fp12_00	12	0.612
RB_2956505_L12_PA	fp12_00	12	0.612
FB_0110377_L12_PA	fp12_00	12	0.612
FB_0855312_L12_27_1	fp12_00	12	0.612
FB_0111163_L12_PA	fp12_00	12	0.612
RB_1240623_L12_PA	fp12_00	12	0.612
RB_1475357_L12_PA	fp12_00	12	0.612
FB_0109517_L12_PA	fp12_00	12	0.612
RB_1193551_L12_PA	fp12_00	12	0.612
RB_1215786_L12_PA	fp12_00	12	0.612
RB_1816057_L12_PA	fp12_00	12	0.612
FB_0110379_L12_PA	fp12_00	12	0.612
RB_1818084_L12_PA	fp12_00	12	0.612
FB_0108825_L12_PA	fp12_00	12	0.612
FB_0110376_L12_PA	fp12_00	12	0.612
FB_0108826_L12_PA	fp12_00	12	0.612
RB_1195935_L12_PA	fp12_00	12	0.612
FB_0110362_L12_PA	fp12_00	12	0.612
RB_2343296_L12_PA	fp12_00	12	0.612
FB_0110890_L12_PA	fp12_00	12	0.612
FB_0108793_L12_PA	fp12_01	12	1.635
FB_0110887_L12_PA	fp12_01	12	1.635
FB_0108815_L12_PA	fp12_01	12	1.635
RB_1205264_L12_PA	fp12_01	12	1.635
FB_0108809_L12_PA	fp12_01	12	1.635
FB_0109515_L12_PA	fp12_01	12	1.635
FB_0111366_L12_PA	fp12_01	12	1.635
FB_0855318_L12_27_1	fp12_01	12	1.635
FB_0111348_L12_PA	fp12_01	12	1.635
GD_00116_L12_PA	fp12_01	12	1.635
FB_0855293_L12_27_1	fp12_01	12	1.635
FB_0110363_L12_PA	fp12_01	12	1.635

FB_0110371_L12_PA	fp12_01	12	1.635
FB_0418365_L1_PA	fp12_02	12	2.677
RB_3446082_L12_PA	fp12_02	12	2.677
FB_0111352_L12_PA	fp12_02	12	2.677
FB_0112256_L12_PA	fp12_02	12	2.677
FB_0111355_L12_PA	fp12_02	12	2.677
FB_0111904_L12_PA	fp12_02	12	2.677
RB_3506169_L12_PA	fp12_03	12	3.517
FB_0855319_L12_27_1	fp12_03	12	3.517
GD_01615_L12_PA	fp12_03	12	3.517
FB_0112239_L12_PA	fp12_03	12	3.517
FB_0112260_L12_PA	fp12_03	12	3.517
GD_01391_L12_PA	fp12_03	12	3.517
FB_0112245_L12_PA	fp12_03	12	3.517
FB_0110366_L12_PA	fp12_03	12	3.517
RB_3025133_L12_PA	fp12_04	12	4.202
FB_0112238_L12_PA	fp12_04	12	4.202
RB_3725831_L12_PA	fp12_05	12	5.451
GD_00430_L12_PA	fp12_05	12	5.451
FB_0855922_L12_27_1	fp12_06	12	6.268
FB_0855803_L12_27_1	fp12_06	12	6.268
FB_0112839_L12_PA	fp12_06	12	6.268
FB_0855931_L12_27_1	fp12_06	12	6.268
FB_0113077_L12_PA	fp12_06	12	6.268
FB_1045136_L9_23_1	fp12_06	12	6.268
FB_0855932_L12_27_1	fp12_06	12	6.268
FB_0855929_L12_27_1	fp12_06	12	6.268
FB_0113071_L12_PA	fp12_06	12	6.268
FB_0113072_L12_PA	fp12_06	12	6.268
FB_0855807_L12_27_1	fp12_06	12	6.268
FB_0113090_L12_PA	fp12_06	12	6.268
FB_1045122_L9_23_1	fp12_06	12	6.268
FB_1045123_L9_23_1	fp12_06	12	6.268
FB_0855815_L12_27_1	fp12_06	12	6.268
FB_0113348_L12_PA	fp12_06	12	6.268
FB_0113360_L12_PA	fp12_06	12	6.268
RB_4336568_L12_PA	fp12_06	12	6.268
FB_0113377_L12_PA	fp12_07	12	7.876
FB_0113340_L12_PA	fp12_07	12	7.876
RB_4304933_L12_PA	fp12_07	12	7.876
FB_0113378_L12_PA	fp12_07	12	7.876
FB_0113339_L12_PA	fp12_07	12	7.876
FB_0114383_L12_PA	fp12_08	12	8.011
FB_0114369_L12_PA	fp12_09	12	9.139
FB_0114362_L12_PA	fp12_09	12	9.139
FB_0114368_L12_PA	fp12_09	12	9.139

FB_0114363_L12_PA	fp12_09	12	9.139
GD_01907_L12_PA	fp12_09	12	9.139
FB_0114799_L12_PA	fp12_09	12	9.139
FB_0116521_L12_PA	fp12_10	12	10.903
FB_0114795_L12_PA	fp12_10	12	10.903
FB_0114822_L12_PA	fp12_10	12	10.903
FB_0114805_L12_PA	fp12_10	12	10.903
FB_0116519_L12_PA	fp12_10	12	10.903
FB_1059530_L12_49_2	fp12_10	12	10.903
FB_0856387_L12_28_1	fp12_10	12	10.903
FB_0114819_L12_PA	fp12_10	12	10.903
FB_0116527_L12_PA	fp12_10	12	10.903
FB_0114826_L12_PA	fp12_10	12	10.903
FB_0856389_L12_28_1	fp12_10	12	10.903
RB_5173778_L12_PA	fp12_10	12	10.903
FB_1059526_L12_49_2	fp12_10	12	10.903
FB_0114810_L12_PA	fp12_10	12	10.903
RB_7275449_L12_PA	fp12_11	12	11.689
FB_0250746_L14_PA	fp12_11	12	11.689
FB_0789370_L9_PA	fp12_12	12	12.381
FB_0789350_L9_PA	fp12_12	12	12.381
FB_0118326_L12_PA	fp12_12	12	12.381
FB_0134978_L12_PA	fp12_12	12	12.381
FB_0789349_L9_PA	fp12_12	12	12.381
FB_0789346_L9_PA	fp12_12	12	12.381
FB_1044212_L9_23_1	fp12_12	12	12.381
FB_0134946_L12_PA	fp12_12	12	12.381
RB_5173113_L12_PA	fp12_12	12	12.381
RB_5411076_L12_PA	fp12_12	12	12.381
FB_0134949_L12_PA	fp12_12	12	12.381
FB_0223654_L14_PA	fp12_12	12	12.381
FB_0117243_L12_PA	fp12_12	12	12.381
FB_0117757_L12_PA	fp12_12	12	12.381
RB_7044670_L12_PA	fp12_12	12	12.381
FB_0118080_L12_PA	fp12_12	12	12.381
FB_0119351_L12_PA	fp12_12	12	12.381
FB_1044215_L9_23_1	fp12_12	12	12.381
FB_0118091_L12_PA	fp12_12	12	12.381
FB_0134961_L12_PA	fp12_12	12	12.381
FB_0118081_L12_PA	fp12_12	12	12.381
FB_0117245_L12_PA	fp12_12	12	12.381
FB_0119339_L12_PA	fp12_12	12	12.381
FB_0117239_L12_PA	fp12_12	12	12.381
FB_0225931_L14_PA	fp12_12	12	12.381
FB_0223616_L14_PA	fp12_12	12	12.381
FB_0250756_L14_PA	fp12_12	12	12.381

FB_0134948_L12_PA	fp12_12	12	12.381
FB_0223625_L14_PA	fp12_12	12	12.381
FB_0789387_L9_PA	fp12_12	12	12.381
FB_0223619_L14_PA	fp12_12	12	12.381
FB_0117748_L12_PA	fp12_12	12	12.381
FB_0119342_L12_PA	fp12_12	12	12.381
FB_0117741_L12_PA	fp12_12	12	12.381
FB_0225936_L14_PA	fp12_12	12	12.381
FB_0223640_L14_PA	fp12_12	12	12.381
FB_0118976_L12_PA	fp12_13	12	13.43
FB_0225586_L14_PA	fp12_13	12	13.43
FB_0118970_L12_PA	fp12_13	12	13.43
FB_0118966_L12_PA	fp12_13	12	13.43
FB_0857162_L12_32_1	fp12_13	12	13.43
FB_0118958_L12_PA	fp12_13	12	13.43
FB_0225603_L14_PA	fp12_13	12	13.43
FB_0857164_L12_32_1	fp12_13	12	13.43
GD_01703_L12_PA	fp12_13	12	13.43
FB_0857167_L12_32_1	fp12_13	12	13.43
FB_0225605_L14_PA	fp12_13	12	13.43
FB_0857165_L12_32_1	fp12_13	12	13.43
RB_8805420_L12_PA	fp12_13	12	13.43
FB_0119357_L12_PA	fp12_13	12	13.43
RB_8821550_L12_PA	fp12_13	12	13.43
FB_0225610_L14_PA	fp12_14	12	14.432
FB_0225932_L14_PA	fp12_14	12	14.432
FB_0225596_L14_PA	fp12_14	12	14.432
FB_0225587_L14_PA	fp12_14	12	14.432
FB_0118965_L12_PA	fp12_14	12	14.432
FB_0225595_L14_PA	fp12_14	12	14.432
FB_0119565_L12_PA	fp12_14	12	14.432
FB_0119569_L12_PA	fp12_14	12	14.432
RB_8810680_L12_PA	fp12_14	12	14.432
FB_0119366_L12_PA	fp12_14	12	14.432
RB_8802561_L12_PA	fp12_14	12	14.432
FB_0119337_L12_PA	fp12_14	12	14.432
FB_0118488_L12_PA	fp12_14	12	14.432
FB_0119353_L12_PA	fp12_14	12	14.432
FB_0120081_L12_PA	fp12_15	12	15.548
RB_9274031_L12_PA	fp12_15	12	15.548
FB_0857653_L12_33_1	fp12_15	12	15.548
FB_0857668_L12_33_1	fp12_15	12	15.548
FB_0120098_L12_PA	fp12_15	12	15.548
FB_0857647_L12_33_1	fp12_15	12	15.548
FB_0634977_L5_PA	fp12_15	12	15.548
RB_9330075_L12_PA	fp12_15	12	15.548

FB_0999931_L5_51_1	fp12_15	12	15.548
FB_0120077_L12_PA	fp12_15	12	15.548
FB_0120075_L12_PA	fp12_15	12	15.548
FB_0999945_L5_51_1	fp12_15	12	15.548
FB_0635022_L5_PA	fp12_15	12	15.548
FB_0120082_L12_PA	fp12_15	12	15.548
GD_02053_L12_PA	fp12_15	12	15.548
FB_0999957_L5_51_1	fp12_15	12	15.548
FB_0120095_L12_PA	fp12_15	12	15.548
FB_0635020_L5_PA	fp12_15	12	15.548
FB_0120078_L12_PA	fp12_15	12	15.548
FB_0999953_L5_51_1	fp12_15	12	15.548
FB_0124929_L12_PA	fp12_16	12	16.805
FB_0125522_L12_PA	fp12_16	12	16.805
FB_0126187_L12_PA	fp12_16	12	16.805
FB_0126141_L12_PA	fp12_16	12	16.805
FB_0126134_L12_PA	fp12_16	12	16.805
FB_0122736_L12_PA	fp12_16	12	16.805
FB_0124942_L12_PA	fp12_16	12	16.805
FB_0082448_L11_PA	fp12_16	12	16.805
FB_0123463_L12_PA	fp12_16	12	16.805
FB_0853221_L11_47_1	fp12_16	12	16.805
FB_0125386_L12_PA	fp12_16	12	16.805
FB_0082462_L11_PA	fp12_16	12	16.805
FB_0121073_L12_PA	fp12_16	12	16.805
FB_0124940_L12_PA	fp12_16	12	16.805
FB_0125389_L12_PA	fp12_16	12	16.805
FB_0124936_L12_PA	fp12_16	12	16.805
FB_0121090_L12_PA	fp12_16	12	16.805
FB_0124088_L12_PA	fp12_16	12	16.805
FB_0124090_L12_PA	fp12_16	12	16.805
FB_0124095_L12_PA	fp12_16	12	16.805
FB_0124934_L12_PA	fp12_16	12	16.805
FB_0082477_L11_PA	fp12_16	12	16.805
FB_0124944_L12_PA	fp12_16	12	16.805
FB_0858095_L12_35_1	fp12_16	12	16.805
FB_0125525_L12_PA	fp12_16	12	16.805
FB_0121053_L12_PA	fp12_17	12	17.253
FB_0121064_L12_PA	fp12_17	12	17.253
FB_0124093_L12_PA	fp12_17	12	17.253
FB_0121061_L12_PA	fp12_17	12	17.253
FB_0126179_L12_PA	fp12_17	12	17.253
FB_1057895_L11_58_2	fp12_17	12	17.253
FB_0121089_L12_PA	fp12_17	12	17.253
GD_02814_L12_PA	fp12_17	12	17.253
FB_0124091_L12_PA	fp12_17	12	17.253

FB_0126161_L12_PA	fp12_17	12	17.253
FB_0121074_L12_PA	fp12_17	12	17.253
FB_0858092_L12_35_1	fp12_17	12	17.253
FB_0103299_L11_PA	fp12_17	12	17.253
RB_16343119_L12_PA	fp12_18	12	18.739
RB_16355366_L12_PA	fp12_18	12	18.739
FB_0635661_L5_PA	fp12_19	12	19.568
FB_0635633_L5_PA	fp12_19	12	19.568
RB_16317377_L12_PA	fp12_19	12	19.568
FB_0127620_L12_PA	fp12_19	12	19.568
FB_0859499_L12_38_1	fp12_19	12	19.568
GD_01501_L12_PA	fp12_19	12	19.568
FB_0128307_L12_PA	fp12_19	12	19.568
FB_0128358_L12_PA	fp12_19	12	19.568
FB_0859505_L12_38_1	fp12_20	12	20.447
FB_0128330_L12_PA	fp12_20	12	20.447
RB_16369231_L12_PA	fp12_20	12	20.447
FB_0128309_L12_PA	fp12_20	12	20.447
FB_0128315_L12_PA	fp12_20	12	20.447
FB_0129384_L12_PA	fp12_20	12	20.447
FB_0129369_L12_PA	fp12_20	12	20.447
FB_0543517_L3_PA	fp12_20	12	20.447
FB_0130131_L12_PA	fp12_20	12	20.447
FB_0128823_L12_PA	fp12_20	12	20.447
FB_0129370_L12_PA	fp12_20	12	20.447
FB_0129371_L12_PA	fp12_20	12	20.447
FB_0543523_L3_PA	fp12_20	12	20.447
GD_02115_L12_39_1	fp12_20	12	20.447
FB_0128851_L12_PA	fp12_20	12	20.447
FB_0130114_L12_PA	fp12_20	12	20.447
FB_0128827_L12_PA	fp12_20	12	20.447
FB_0129399_L12_PA	fp12_20	12	20.447
RB_17312775_L12_PA	fp12_21	12	21.627
FB_0129398_L12_PA	fp12_21	12	21.627
FB_0543540_L3_PA	fp12_21	12	21.627
FB_0131938_L12_PA	fp12_21	12	21.627
FB_0860464_L12_39_1	fp12_21	12	21.627
FB_0125521_L12_PA	fp12_21	12	21.627
FB_0082467_L11_PA	fp12_21	12	21.627
FB_0131440_L12_PA	fp12_21	12	21.627
FB_0133548_L12_PA	fp12_21	12	21.627
FB_0133575_L12_PA	fp12_21	12	21.627
FB_0860436_L12_39_1	fp12_21	12	21.627
RB_17368072_L12_PA	fp12_21	12	21.627
FB_0133559_L12_PA	fp12_21	12	21.627
FB_0860466_L12_39_1	fp12_21	12	21.627

FB_0131004_L12_PA	fp12_21	12	21.627
RB_20525005_L12_PA	fp12_21	12	21.627
GD_00599_L12_PA	fp12_21	12	21.627
FB_0133552_L12_PA	fp12_21	12	21.627
FB_1060566_L12_56_2	fp12_21	12	21.627
FB_0130997_L12_PA	fp12_21	12	21.627
FB_0860462_L12_39_1	fp12_21	12	21.627
FB_0131936_L12_PA	fp12_22	12	22.277
RB_21195004_L12_PA	fp12_22	12	22.277
FB_0133817_L12_PA	fp12_22	12	22.277
FB_0130137_L12_PA	fp12_22	12	22.277
RB_22652840_L12_PA	fp12_22	12	22.277
GD_01555_L12_PA	fp12_22	12	22.277
FB_0131929_L12_PA	fp12_22	12	22.277
RB_19224299_L12_PA	fp12_22	12	22.277
RB_19244913_L12_PA	fp12_22	12	22.277
FB_0131933_L12_PA	fp12_22	12	22.277
GD_01357_L12_PA	fp12_23	12	23.941
FB_0133829_L12_PA	fp12_24	12	24.591
RB_21326884_L12_PA	fp12_24	12	24.591
FB_0134629_L12_PA	fp12_24	12	24.591
RB_21248954_L12_PA	fp12_25	12	25.52
FB_0133816_L12_PA	fp12_25	12	25.52
FB_0133815_L12_PA	fp12_25	12	25.52
RB_21385102_L12_PA	fp12_25	12	25.52
FB_0860971_L12_39_1	fp12_25	12	25.52
RB_22654900_L12_PA	fp12_25	12	25.52
GD_00314_L12_PA	fp12_26	12	26.329
RB_21401673_L12_PA	fp12_26	12	26.329
FB_0133818_L12_PA	fp12_26	12	26.329
FB_0864452_L12_46_1	fp12_26	12	26.329
FB_0135471_L12_PA	fp12_26	12	26.329
FB_0135442_L12_PA	fp12_26	12	26.329
FB_0861622_L12_42_1	fp12_26	12	26.329
FB_0135158_L12_PA	fp12_26	12	26.329
FB_0134628_L12_PA	fp12_26	12	26.329
FB_1060723_L12_58_2	fp12_26	12	26.329
FB_0861177_L12_41_1	fp12_26	12	26.329
FB_0135161_L12_PA	fp12_26	12	26.329
FB_0861603_L12_42_1	fp12_26	12	26.329
FB_0134646_L12_PA	fp12_26	12	26.329
GD_01150_L12_PA	fp12_26	12	26.329
FB_1060729_L12_58_2	fp12_26	12	26.329
FB_0135460_L12_PA	fp12_26	12	26.329
FB_0861180_L12_41_1	fp12_26	12	26.329
RB_22722118_L12_42_1	fp12_26	12	26.329

FB_0861607_L12_42_1	fp12_26	12	26.329
FB_0134639_L12_PA	fp12_26	12	26.329
FB_0955785_L2_26_1	fp12_26	12	26.329
FB_0135452_L12_PA	fp12_26	12	26.329
FB_0133819_L12_PA	fp12_26	12	26.329
RB_21317748_L12_PA	fp12_26	12	26.329
FB_0134656_L12_PA	fp12_26	12	26.329
FB_0861619_L12_42_1	fp12_26	12	26.329
FB_0133826_L12_PA	fp12_26	12	26.329
RB_24006092_L12_PA	fp12_26	12	26.329
FB_0135153_L12_PA	fp12_26	12	26.329
RB_23385635_L12_PA	fp12_27	12	27.255
FB_1061765_L12_61_2	fp12_27	12	27.255
FB_0135603_L12_PA	fp12_27	12	27.255
FB_0135594_L12_PA	fp12_27	12	27.255
FB_0135585_L12_PA	fp12_27	12	27.255
FB_0135600_L12_PA	fp12_27	12	27.255
FB_1061772_L12_61_2	fp12_27	12	27.255
FB_0135596_L12_PA	fp12_27	12	27.255
RB_23280662_L12_PA	fp12_27	12	27.255
FB_0135604_L12_PA	fp12_27	12	27.255
RB_21257957_L12_PA	fp12_27	12	27.255
FB_0133814_L12_PA	fp12_27	12	27.255
RB_23400893_L12_PA	fp12_27	12	27.255
RB_23391266_L12_PA	fp12_28	12	28.516
FB_0136411_L12_PA	fp12_28	12	28.516
FB_0862766_L12_44_1	fp12_29	12	29.272
FB_0139780_L12_PA	fp12_29	12	29.272
FB_0983199_L4_22_1	fp12_29	12	29.272
FB_0742392_L8_PA	fp12_29	12	29.272
GD_01798_L12_PA	fp12_29	12	29.272
FB_0137091_L12_PA	fp12_29	12	29.272
FB_0862765_L12_44_1	fp12_29	12	29.272
FB_0137116_L12_PA	fp12_29	12	29.272
FB_0136418_L12_PA	fp12_29	12	29.272
FB_0136419_L12_PA	fp12_29	12	29.272
FB_0139788_L12_PA	fp12_29	12	29.272
FB_0137102_L12_PA	fp12_29	12	29.272
FB_0742396_L8_PA	fp12_29	12	29.272
FB_0139763_L12_PA	fp12_29	12	29.272
FB_0137094_L12_PA	fp12_29	12	29.272
FB_0136421_L12_PA	fp12_29	12	29.272
FB_0137109_L12_PA	fp12_29	12	29.272
FB_0141444_L12_PA	fp12_29	12	29.272
FB_0140970_L12_PA	fp12_29	12	29.272
FB_0140788_L12_PA	fp12_30	12	30.794

FB_1091489_L4_28_2	fp12_30	12	30.794
FB_0136416_L12_PA	fp12_30	12	30.794
FB_0140326_L12_PA	fp12_31	12	31.453
FB_0862187_L12_43_1	fp12_31	12	31.453
RB_29037245_L12_PA	fp12_32	12	32.475
RB_29023351_L12_PA	fp12_32	12	32.475
RB_29040874_L12_PA	fp12_32	12	32.475
FB_0141454_L12_PA	fp12_33	12	33.452
FB_0140991_L12_PA	fp12_33	12	33.452
FB_0140979_L12_PA	fp12_33	12	33.452
FB_0140790_L12_PA	fp12_33	12	33.452
FB_0863154_L12_45_1	fp12_33	12	33.452
FB_0181906_L13_PA	fp12_33	12	33.452
FB_1109632_L13_73_3	fp12_33	12	33.452
FB_0141486_L12_PA	fp12_33	12	33.452
FB_0140805_L12_PA	fp12_33	12	33.452
FB_0141244_L12_PA	fp12_33	12	33.452
RB_27689885_L12_PA	fp12_33	12	33.452
FB_1091498_L4_28_2	fp12_33	12	33.452
FB_0140968_L12_PA	fp12_33	12	33.452
FB_0181929_L13_PA	fp12_33	12	33.452
FB_0140796_L12_PA	fp12_33	12	33.452
FB_0141468_L12_PA	fp12_33	12	33.452
FB_0141245_L12_PA	fp12_33	12	33.452
FB_1108951_L12_71_3	fp12_33	12	33.452
FB_0140808_L12_PA	fp12_33	12	33.452
RB_27155494_L12_PA	fp12_33	12	33.452
FB_0141443_L12_PA	fp12_33	12	33.452
FB_0863120_L12_44_1	fp12_33	12	33.452
FB_1091502_L4_28_2	fp12_33	12	33.452
RB_27168371_L12_PA	fp12_33	12	33.452
FB_0863675_L12_46_1	fp12_33	12	33.452
FB_0863678_L12_46_1	fp12_33	12	33.452
GD_01769_L12_PA	fp12_33	12	33.452
FB_0142661_L12_PA	fp12_33	12	33.452
FB_0142655_L12_PA	fp12_33	12	33.452
FB_0142631_L12_PA	fp12_34	12	34.477
FB_0143071_L12_PA	fp12_34	12	34.477
FB_0617866_L5_PA	fp12_34	12	34.477
FB_0143061_L12_PA	fp12_34	12	34.477
FB_0142648_L12_PA	fp12_35	12	35.364
RB_29098150_L12_PA	fp12_35	12	35.364
FB_0143043_L12_PA	fp12_35	12	35.364
FB_1091503_L4_28_2	fp12_35	12	35.364
RB_29723749_L12_PA	fp12_35	12	35.364
FB_0145071_L12_PA	fp12_35	12	35.364

FB_0181919_L13_PA	fp12_35	12	35.364
FB_0140971_L12_PA	fp12_35	12	35.364
FB_0146185_L12_PA	fp12_36	12	36.384
FB_0864896_L12_46_1	fp12_36	12	36.384
FB_1061424_L12_61_2	fp12_36	12	36.384
GD_01420_L12_PA	fp12_36	12	36.384
FB_0146181_L12_PA	fp12_36	12	36.384
FB_0864902_L12_46_1	fp12_36	12	36.384
FB_0864904_L12_46_1	fp12_36	12	36.384
FB_1061427_L12_61_2	fp12_36	12	36.384
FB_1061426_L12_61_2	fp12_36	12	36.384
FB_0864895_L12_46_1	fp12_36	12	36.384
FB_0145074_L12_PA	fp12_36	12	36.384
RB_30684803_L12_PA	fp12_36	12	36.384
FB_0146178_L12_PA	fp12_36	12	36.384
FB_0146183_L12_PA	fp12_36	12	36.384
FB_0145023_L12_PA	fp12_36	12	36.384
FB_0145054_L12_PA	fp12_36	12	36.384
FB_0865132_L12_46_1	fp12_37	12	37.753
FB_0865133_L12_46_1	fp12_37	12	37.753
FB_0865128_L12_46_1	fp12_37	12	37.753
FB_0865134_L12_46_1	fp12_37	12	37.753
FB_0121066_L12_PA	fp12_37	12	37.753
FB_0146889_L12_PA	fp12_37	12	37.753
FB_0146875_L12_PA	fp12_37	12	37.753
FB_0146192_L12_PA	fp12_37	12	37.753
FB_0146191_L12_PA	fp12_37	12	37.753
FB_0865877_L12_46_1	fp12_38	12	38.575
FB_0865143_L12_46_1	fp12_38	12	38.575
FB_1115961_L5_91_3	fp12_38	12	38.575
FB_0146888_L12_PA	fp12_38	12	38.575
FB_1115969_L5_91_3	fp12_38	12	38.575
FB_1115970_L5_91_3	fp12_38	12	38.575
FB_0865940_L12_46_1	fp12_39	12	39.247
FB_0147970_L12_PA	fp12_39	12	39.247
FB_0148938_L12_PA	fp12_39	12	39.247
FB_0147935_L12_PA	fp12_39	12	39.247
GD_01426_L12_PA	fp12_40	12	40.006
FB_0147250_L12_PA	fp12_41	12	41.277
FB_0147252_L12_PA	fp12_41	12	41.277
RB_31180930_L12_PA	fp12_41	12	41.277
FB_0147267_L12_PA	fp12_41	12	41.277
FB_0147255_L12_PA	fp12_41	12	41.277
FB_0147260_L12_PA	fp12_41	12	41.277
FB_0147256_L12_PA	fp12_41	12	41.277
FB_0147268_L12_PA	fp12_41	12	41.277

RB_30691702_L12_46_1	fp12_41	12	41.277
FB_0147965_L12_PA	fp12_42	12	42.48
FB_0147955_L12_PA	fp12_42	12	42.48
FB_0147893_L12_PA	fp12_42	12	42.48
FB_0865863_L12_46_1	fp12_42	12	42.48
GD_00641_L12_PA	fp12_42	12	42.48
FB_0147963_L12_PA	fp12_42	12	42.48
FB_0865865_L12_46_1	fp12_42	12	42.48
FB_0147940_L12_PA	fp12_42	12	42.48
FB_0865893_L12_46_1	fp12_42	12	42.48
FB_0147949_L12_PA	fp12_42	12	42.48
FB_0147881_L12_PA	fp12_42	12	42.48
FB_0147901_L12_PA	fp12_42	12	42.48
FB_0865864_L12_46_1	fp12_42	12	42.48
FB_0147249_L12_PA	fp12_42	12	42.48
FB_0147890_L12_PA	fp12_42	12	42.48
FB_0147896_L12_PA	fp12_42	12	42.48
FB_0865868_L12_46_1	fp12_42	12	42.48
FB_1062032_L12_61_2	fp12_42	12	42.48
FB_0865859_L12_46_1	fp12_42	12	42.48
FB_0865945_L12_46_1	fp12_42	12	42.48
FB_0865932_L12_46_1	fp12_42	12	42.48
RB_31638697_L12_PA	fp12_43	12	43.658
FB_0148274_L12_PA	fp12_43	12	43.658
FB_0148294_L12_PA	fp12_43	12	43.658
RB_32489536_L12_PA	fp12_44	12	44.676
FB_0866253_L12_46_1	fp12_44	12	44.676
FB_0149403_L12_PA	fp12_44	12	44.676
GD_00762_L12_PA	fp12_44	12	44.676
FB_0148933_L12_PA	fp12_44	12	44.676
FB_0148935_L12_PA	fp12_44	12	44.676
FB_0148922_L12_PA	fp12_44	12	44.676
RB_32469819_L12_PA	fp12_45	12	45.807
RB_32114913_L12_PA	fp12_45	12	45.807
FB_0866260_L12_46_1	fp12_45	12	45.807
RB_32711249_L12_PA	fp12_45	12	45.807
RB_32472396_L12_PA	fp12_45	12	45.807
FB_0149411_L12_PA	fp12_45	12	45.807
FB_0149412_L12_PA	fp12_45	12	45.807
FB_0866760_L12_46_1	fp12_46	12	46.258
FB_0866635_L12_46_1	fp12_46	12	46.258
FB_0150011_L12_PA	fp12_46	12	46.258
GD_00362_L12_PA	fp12_46	12	46.258
FB_0149992_L12_PA	fp12_46	12	46.258
FB_0149997_L12_PA	fp12_46	12	46.258
FB_0149417_L12_PA	fp12_46	12	46.258

FB_0149419_L12_PA	fp12_46	12	46.258
FB_0866257_L12_46_1	fp12_46	12	46.258
FB_0149401_L12_PA	fp12_46	12	46.258
FB_0866258_L12_46_1	fp12_46	12	46.258
FB_0150001_L12_PA	fp12_46	12	46.258
FB_0149400_L12_PA	fp12_46	12	46.258
GD_00840_L12_PA	fp12_46	12	46.258
FB_0866256_L12_46_1	fp12_46	12	46.258
FB_0866661_L12_46_1	fp12_46	12	46.258
RB_32726730_L12_PA	fp12_46	12	46.258
FB_0866753_L12_46_1	fp12_46	12	46.258
FB_0150553_L12_PA	fp12_46	12	46.258
RB_33183417_L12_PA	fp12_46	12	46.258
RB_32759123_L12_PA	fp12_46	12	46.258
FB_0866759_L12_46_1	fp12_46	12	46.258
FB_0866743_L12_46_1	fp12_46	12	46.258
FB_0151258_L12_PA	fp12_47	12	47.374
FB_0150544_L12_PA	fp12_47	12	47.374
FB_0150541_L12_PA	fp12_47	12	47.374
FB_1017431_L7_39_1	fp12_47	12	47.374
FB_0866768_L12_46_1	fp12_47	12	47.374
FB_0866902_L12_46_1	fp12_47	12	47.374
FB_0151264_L12_PA	fp12_47	12	47.374
RB_33692967_L12_PA	fp12_47	12	47.374
FB_0866895_L12_46_1	fp12_47	12	47.374
RB_33709896_L12_PA	fp12_47	12	47.374
GD_02008_L12_PA	fp12_47	12	47.374
FB_0866910_L12_46_1	fp12_47	12	47.374
FB_1017441_L7_39_1	fp12_47	12	47.374
FB_0866745_L12_46_1	fp12_47	12	47.374
FB_0866897_L12_46_1	fp12_47	12	47.374
FB_1017440_L7_39_1	fp12_47	12	47.374
RB_33687206_L12_PA	fp12_47	12	47.374
FB_0866893_L12_46_1	fp12_47	12	47.374
FB_0150538_L12_PA	fp12_47	12	47.374
FB_1017455_L7_39_1	fp12_47	12	47.374
FB_0866920_L12_46_1	fp12_47	12	47.374
FB_0151248_L12_PA	fp12_47	12	47.374
FB_0151256_L12_PA	fp12_47	12	47.374
RB_33563721_L12_PA	fp12_47	12	47.374
FB_0866653_L12_46_1	fp12_47	12	47.374
FB_0866649_L12_46_1	fp12_47	12	47.374
FB_0150547_L12_PA	fp12_47	12	47.374
RB_33183873_L12_PA	fp12_47	12	47.374
RB_33388245_L12_PA	fp12_47	12	47.374
RB_33390300_L12_PA	fp12_47	12	47.374

FB_0867411_L12_46_1	fp12_47	12	47.374
FB_0867409_L12_46_1	fp12_47	12	47.374
RB_33117888_L12_61_2	fp12_48	12	48.508
RB_33383602_L12_PA	fp12_48	12	48.508
FB_0866896_L12_46_1	fp12_48	12	48.508
RB_33385879_L12_PA	fp12_48	12	48.508
FB_0866918_L12_46_1	fp12_48	12	48.508
FB_0151267_L12_PA	fp12_48	12	48.508
FB_0152374_L12_PA	fp12_48	12	48.508
RB_33397489_L12_PA	fp12_48	12	48.508
FB_0867538_L12_46_1	fp12_48	12	48.508
FB_0867539_L12_46_1	fp12_48	12	48.508
FB_0153857_L12_PA	fp12_48	12	48.508
FB_0153826_L12_PA	fp12_48	12	48.508
FB_0867544_L12_46_1	fp12_49	12	49.284
FB_0867549_L12_46_1	fp12_49	12	49.284
FB_0867407_L12_46_1	fp12_49	12	49.284
FB_1062420_L12_61_2	fp12_49	12	49.284
FB_0153828_L12_PA	fp12_50	12	50.551
FB_0867854_L12_46_1	fp12_50	12	50.551
GD_01874_L12_PA	fp12_50	12	50.551
GD_02228_L12_61_2	fp12_50	12	50.551
FB_1062419_L12_61_2	fp12_50	12	50.551
FB_0153240_L12_PA	fp12_50	12	50.551
FB_1062422_L12_61_2	fp12_50	12	50.551
FB_1062411_L12_61_2	fp12_50	12	50.551
FB_0153255_L12_PA	fp12_50	12	50.551
FB_0153863_L12_PA	fp12_50	12	50.551
RB_34815269_L12_PA	fp12_50	12	50.551
RB_34953707_L12_PA	fp12_50	12	50.551
FB_0867856_L12_46_1	fp12_50	12	50.551
RB_34822031_L12_PA	fp12_50	12	50.551
FB_0867878_L12_46_1	fp12_50	12	50.551
FB_0153836_L12_PA	fp12_50	12	50.551
RB_34804790_L12_PA	fp12_50	12	50.551
RB_35230462_L12_PA	fp12_50	12	50.551
RB_34960014_L12_PA	fp12_50	12	50.551
RB_35618016_L12_PA	fp12_50	12	50.551
FB_0153841_L12_PA	fp12_50	12	50.551
FB_0153244_L12_PA	fp12_50	12	50.551
FB_0867884_L12_46_1	fp12_50	12	50.551
FB_0153264_L12_PA	fp12_50	12	50.551
FB_1062410_L12_61_2	fp12_50	12	50.551
RB_34800048_L12_PA	fp12_50	12	50.551
RB_34809522_L12_PA	fp12_50	12	50.551
RB_34921188_L12_PA	fp12_50	12	50.551

FB_0867885_L12_46_1	fp12_50	12	50.551
FB_0153850_L12_PA	fp12_50	12	50.551
FB_0867888_L12_46_1	fp12_50	12	50.551
FB_0153833_L12_PA	fp12_50	12	50.551
MDP0000951479	fp12_51	12	51.584
FB_0154728_L12_PA	fp12_51	12	51.584
FB_0867882_L12_46_1	fp12_51	12	51.584
RB_35134191_L12_PA	fp12_51	12	51.584
RB_35103565_L12_PA	fp12_51	12	51.584
FB_1062574_L12_61_2	fp12_51	12	51.584
RB_35615663_L12_PA	fp12_51	12	51.584
FB_0154300_L12_PA	fp12_52	12	52.503
FB_0154283_L12_PA	fp12_52	12	52.503
RB_35171763_L12_PA	fp12_52	12	52.503
FB_0154293_L12_PA	fp12_52	12	52.503
FB_1062561_L12_61_2	fp12_52	12	52.503
FB_0154726_L12_PA	fp12_52	12	52.503
FB_0867855_L12_46_1	fp12_52	12	52.503
GD_01793_L12_PA	fp12_52	12	52.503
EV_Eve1_R422	fp12_52	12	52.503
FB_0154733_L12_PA	fp12_52	12	52.503
FB_0154743_L12_PA	fp12_52	12	52.503
GD_00338_L12_PA	fp12_52	12	52.503
FB_0154747_L12_PA	fp12_52	12	52.503
FB_0154749_L12_PA	fp12_52	12	52.503
FB_0868356_L12_46_1	fp12_53	12	53.217
FB_0868172_L12_46_1	fp12_53	12	53.217
FB_0156085_L12_PA	fp12_53	12	53.217
GD_02848_L12_PA	fp12_53	12	53.217
FB_0868360_L12_46_1	fp12_53	12	53.217
FB_0868156_L12_46_1	fp12_53	12	53.217
FB_0868150_L12_46_1	fp12_53	12	53.217
GD_01855_L12_PA	fp12_53	12	53.217
FB_0868362_L12_46_1	fp12_53	12	53.217
FB_0155367_L12_PA	fp12_53	12	53.217
RB_35649702_L12_PA	fp12_53	12	53.217
RB_35907115_L12_PA	fp12_53	12	53.217
RB_35612232_L12_PA	fp12_53	12	53.217
FB_1062550_L12_61_2	fp12_53	12	53.217
FB_1062573_L12_61_2	fp12_53	12	53.217
RB_35835624_L12_PA	fp12_53	12	53.217
FB_1062559_L12_61_2	fp12_53	12	53.217
FB_1062575_L12_61_2	fp12_53	12	53.217
FB_1062568_L12_61_2	fp12_53	12	53.217
RB_35724634_L12_PA	fp12_53	12	53.217
RB_35721635_L12_PA	fp12_53	12	53.217

FB_0868163_L12_46_1	fp12_53	12	53.217
FB_0868354_L12_46_1	fp12_53	12	53.217
FB_0868178_L12_46_1	fp12_53	12	53.217
FB_1062571_L12_61_2	fp12_53	12	53.217
FB_0156873_L13_PA	fp13_00	13	0.217
RB_256845_L13_PA	fp13_00	13	0.217
FB_0156323_L13_PA	fp13_00	13	0.217
RB_339509_L13_PA	fp13_00	13	0.217
FB_0156877_L13_PA	fp13_00	13	0.217
FB_0156321_L13_PA	fp13_00	13	0.217
FB_0156349_L13_PA	fp13_00	13	0.217
FB_0156346_L13_PA	fp13_00	13	0.217
RB_357151_L13_PA	fp13_00	13	0.217
FB_0904050_L15_46_1	fp13_00	13	0.217
RB_329179_L13_PA	fp13_00	13	0.217
FB_0156884_L13_PA	fp13_00	13	0.217
FB_0156340_L13_PA	fp13_00	13	0.217
FB_0156858_L13_PA	fp13_00	13	0.217
FB_0156324_L13_PA	fp13_00	13	0.217
GD_00368_L13_PA	fp13_00	13	0.217
RB_570740_L13_PA	fp13_00	13	0.217
RB_41497_L13_PA	fp13_00	13	0.217
RB_371869_L13_PA	fp13_00	13	0.217
FB_1062750_L13_50_2	fp13_00	13	0.217
FB_0157703_L13_PA	fp13_00	13	0.217
FB_0157718_L13_PA	fp13_00	13	0.217
FB_0157725_L13_PA	fp13_00	13	0.217
FB_0157730_L13_PA	fp13_00	13	0.217
RB_852820_L13_PA	fp13_00	13	0.217
FB_1062744_L13_50_2	fp13_00	13	0.217
RB_875526_L13_PA	fp13_00	13	0.217
FB_0157706_L13_PA	fp13_00	13	0.217
RB_895608_L13_26_1	fp13_01	13	1.659
FB_1062851_L13_50_2	fp13_01	13	1.659
FB_1062847_L13_50_2	fp13_01	13	1.659
FB_1062848_L13_50_2	fp13_01	13	1.659
GD_02018_L13_PA	fp13_01	13	1.659
FB_0904066_L15_46_1	fp13_01	13	1.659
FB_0904073_L15_46_1	fp13_01	13	1.659
FB_0157710_L13_PA	fp13_01	13	1.659
FB_0158484_L13_PA	fp13_01	13	1.659
FB_1062846_L13_50_2	fp13_01	13	1.659
FB_0158497_L13_PA	fp13_01	13	1.659
FB_0158492_L13_PA	fp13_01	13	1.659
RB_1140009_L13_PA	fp13_01	13	1.659
FB_0158498_L13_PA	fp13_01	13	1.659

GD_00279_L13_PA	fp13_01	13	1.659
FB_0158480_L13_PA	fp13_01	13	1.659
FB_0158500_L13_PA	fp13_01	13	1.659
RB_1112866_L13_PA	fp13_02	13	2.546
FB_0904054_L15_46_1	fp13_02	13	2.546
FB_1109354_L13_70_3	fp13_02	13	2.546
FB_1109360_L13_70_3	fp13_02	13	2.546
FB_1109367_L13_70_3	fp13_02	13	2.546
FB_1109370_L13_70_3	fp13_02	13	2.546
FB_1109352_L13_70_3	fp13_02	13	2.546
FB_0158505_L13_PA	fp13_02	13	2.546
RB_602461_L13_PA	fp13_02	13	2.546
RB_317637_L13_PA	fp13_03	13	3.362
FB_0156878_L13_PA	fp13_03	13	3.362
FB_1109362_L13_70_3	fp13_03	13	3.362
RB_1108141_L13_PA	fp13_03	13	3.362
FB_0156338_L13_PA	fp13_04	13	4.504
FB_0159295_L13_PA	fp13_04	13	4.504
FB_0159271_L13_PA	fp13_04	13	4.504
FB_0159290_L13_PA	fp13_04	13	4.504
GD_01579_L13_PA	fp13_05	13	5.333
FB_0158523_L13_PA	fp13_06	13	6.601
FB_0156883_L13_PA	fp13_06	13	6.601
FB_0160147_L13_PA	fp13_06	13	6.601
FB_0160149_L13_PA	fp13_06	13	6.601
FB_0160788_L13_PA	fp13_06	13	6.601
FB_0160790_L13_PA	fp13_06	13	6.601
FB_0160150_L13_PA	fp13_06	13	6.601
GD_00098_L13_PA	fp13_06	13	6.601
RB_2852846_L13_PA	fp13_06	13	6.601
FB_1063135_L13_50_2	fp13_07	13	7.734
FB_1063148_L13_50_2	fp13_07	13	7.734
FB_0160781_L13_PA	fp13_07	13	7.734
RB_2811025_L13_PA	fp13_07	13	7.734
RB_880764_L13_26_1	fp13_07	13	7.734
FB_0161448_L13_PA	fp13_07	13	7.734
RB_2813296_L13_PA	fp13_07	13	7.734
FB_0161442_L13_PA	fp13_07	13	7.734
FB_0161441_L13_PA	fp13_07	13	7.734
FB_0160792_L13_PA	fp13_07	13	7.734
FB_0161454_L13_PA	fp13_08	13	8.583
FB_0161453_L13_PA	fp13_08	13	8.583
FB_0163092_L13_PA	fp13_08	13	8.583
FB_0162288_L13_PA	fp13_08	13	8.583
FB_0163090_L13_PA	fp13_08	13	8.583
FB_1063443_L13_50_2	fp13_08	13	8.583

RB_9219470_L13_PA	fp13_09	13	9.719
GD_01742L13_PA	fp13_09	13	9.719
RB_2217552_L13_PA	fp13_09	13	9.719
RB_2180685_L13_PA	fp13_09	13	9.719
FB_0161449_L13_PA	fp13_10	13	10.413
RB_2823627_L13_PA	fp13_10	13	10.413
FB_0161444_L13_PA	fp13_10	13	10.413
FB_1063445_L13_50_2	fp13_12	13	12.585
RB_2826288_L13_PA	fp13_12	13	12.585
FB_0162295_L13_PA	fp13_12	13	12.585
FB_0162291_L13_PA	fp13_12	13	12.585
FB_0162290_L13_PA	fp13_12	13	12.585
FB_0162262_L13_PA	fp13_12	13	12.585
FB_0163076_L13_PA	fp13_12	13	12.585
FB_1063440_L13_50_2	fp13_12	13	12.585
FB_1109507_L13_70_3	fp13_12	13	12.585
FB_1109486_L13_70_3	fp13_12	13	12.585
FB_1109494_L13_70_3	fp13_12	13	12.585
FB_1109505_L13_70_3	fp13_12	13	12.585
FB_0162293_L13_PA	fp13_12	13	12.585
FB_1109489_L13_70_3	fp13_12	13	12.585
FB_1063447_L13_50_2	fp13_12	13	12.585
RB_3832821_L13_PA	fp13_13	13	13.42
FB_0163084_L13_PA	fp13_13	13	13.42
RB_3856174_L13_PA	fp13_13	13	13.42
FB_0163066_L13_PA	fp13_13	13	13.42
RB_3776960_L13_26_1	fp13_13	13	13.42
GD_00980_L13_PA	fp13_13	13	13.42
GD_00565_L13_PA	fp13_13	13	13.42
FB_0163086_L13_PA	fp13_13	13	13.42
FB_1063469_L13_50_2	fp13_13	13	13.42
FB_0163099_L13_PA	fp13_13	13	13.42
RB_3770859_L13_PA	fp13_13	13	13.42
FB_1063477_L13_50_2	fp13_13	13	13.42
FB_0163068_L13_PA	fp13_13	13	13.42
FB_1063470_L13_50_2	fp13_13	13	13.42
FB_0163853_L13_PA	fp13_13	13	13.42
FB_0163861_L13_PA	fp13_14	13	14.498
FB_0163862_L13_PA	fp13_14	13	14.498
FB_0163866_L13_PA	fp13_14	13	14.498
FB_0164064_L13_PA	fp13_14	13	14.498
RB_4574491_L13_PA	fp13_14	13	14.498
RB_4566253_L13_PA	fp13_14	13	14.498
FB_0163863_L13_PA	fp13_14	13	14.498
FB_0164079_L13_PA	fp13_14	13	14.498
FB_0164072_L13_PA	fp13_14	13	14.498

FB_0164087_L13_PA	fp13_14	13	14.498
FB_0164464_L13_PA	fp13_14	13	14.498
FB_0164461_L13_PA	fp13_15	13	15.487
FB_0164466_L13_PA	fp13_15	13	15.487
FB_0164457_L13_PA	fp13_15	13	15.487
FB_0164463_L13_PA	fp13_15	13	15.487
RB_4580429_L13_PA	fp13_16	13	16.806
FB_0165004_L13_PA	fp13_16	13	16.806
RB_6017316_L13_PA	fp13_16	13	16.806
FB_0166446_L13_PA	fp13_16	13	16.806
RB_4948282_L13_PA	fp13_16	13	16.806
RB_6005626_L13_PA	fp13_16	13	16.806
RB_5662695_L13_PA	fp13_16	13	16.806
FB_0165634_L13_PA	fp13_16	13	16.806
FB_0166438_L13_PA	fp13_16	13	16.806
RB_5611802_L13_PA	fp13_16	13	16.806
RB_5621134_L13_PA	fp13_16	13	16.806
FB_0165639_L13_PA	fp13_16	13	16.806
FB_0166008_L13_PA	fp13_16	13	16.806
FB_0166004_L13_PA	fp13_16	13	16.806
FB_0166015_L13_PA	fp13_16	13	16.806
RB_5624353_L13_PA	fp13_16	13	16.806
FB_0166006_L13_PA	fp13_16	13	16.806
FB_0166011_L13_PA	fp13_16	13	16.806
FB_0165636_L13_PA	fp13_16	13	16.806
RB_5659750_L13_PA	fp13_16	13	16.806
FB_0166422_L13_PA	fp13_16	13	16.806
RB_6014979_L13_PA	fp13_16	13	16.806
FB_0166956_L13_PA	fp13_17	13	17.722
FB_0165003_L13_PA	fp13_17	13	17.722
RB_4926284_L13_PA	fp13_17	13	17.722
RB_5637395_L13_PA	fp13_17	13	17.722
FB_0167427_L13_PA	fp13_18	13	18.271
RB_7138956_L13_PA	fp13_18	13	18.271
FB_0168225_L13_PA	fp13_18	13	18.271
FB_1063546_L13_50_2	fp13_18	13	18.271
FB_1063536_L13_50_2	fp13_18	13	18.271
FB_0168217_L13_PA	fp13_18	13	18.271
FB_0168765_L13_PA	fp13_18	13	18.271
FB_0168191_L13_PA	fp13_18	13	18.271
FB_1063545_L13_50_2	fp13_18	13	18.271
FB_0168210_L13_PA	fp13_18	13	18.271
RB_7165677_L13_PA	fp13_18	13	18.271
FB_0168780_L13_PA	fp13_18	13	18.271
FB_0168208_L13_PA	fp13_18	13	18.271
FB_0168230_L13_PA	fp13_18	13	18.271

RB_6451182_L13_PA	fp13_18	13	18.271
RB_7172710_L13_PA	fp13_18	13	18.271
RB_6451261_L13_PA	fp13_18	13	18.271
FB_0168770_L13_PA	fp13_19	13	19.27
FB_0166933_L13_PA	fp13_19	13	19.27
RB_6306433_L13_PA	fp13_19	13	19.27
FB_0166944_L13_PA	fp13_19	13	19.27
FB_0166930_L13_PA	fp13_19	13	19.27
RB_6361691_L13_PA	fp13_19	13	19.27
FB_1063547_L13_50_2	fp13_19	13	19.27
FB_0168774_L13_PA	fp13_19	13	19.27
FB_0168766_L13_PA	fp13_19	13	19.27
FB_0168749_L13_PA	fp13_19	13	19.27
FB_0168228_L13_PA	fp13_19	13	19.27
FB_0168841_L13_PA	fp13_20	13	20.557
GD_02124_L13_PA	fp13_20	13	20.557
FB_0168840_L13_PA	fp13_20	13	20.557
FB_0169770_L13_PA	fp13_20	13	20.557
FB_0656556_L6_PA	fp13_20	13	20.557
FB_0656547_L6_PA	fp13_20	13	20.557
RB_7144285_L13_PA	fp13_20	13	20.557
RB_7141897_L13_PA	fp13_20	13	20.557
FB_0168763_L13_PA	fp13_20	13	20.557
FB_0169291_L13_PA	fp13_20	13	20.557
FB_0169771_L13_PA	fp13_21	13	21.767
FB_0656546_L6_PA	fp13_21	13	21.767
FB_0656544_L6_PA	fp13_21	13	21.767
FB_1063556_L13_50_2	fp13_21	13	21.767
FB_0170335_L13_PA	fp13_21	13	21.767
FB_0170394_L13_PA	fp13_21	13	21.767
FB_0170332_L13_PA	fp13_21	13	21.767
FB_0170322_L13_PA	fp13_21	13	21.767
FB_0170390_L13_PA	fp13_21	13	21.767
RB_8619897_L13_PA	fp13_21	13	21.767
FB_0656555_L6_PA	fp13_22	13	22.499
FB_0170802_L13_PA	fp13_22	13	22.499
FB_0170803_L13_PA	fp13_23	13	23.535
FB_0170808_L13_PA	fp13_23	13	23.535
FB_0170822_L13_PA	fp13_23	13	23.535
FB_0171459_L13_PA	fp13_23	13	23.535
FB_0171576_L13_PA	fp13_23	13	23.535
FB_0168858_L13_PA	fp13_23	13	23.535
FB_0171446_L13_PA	fp13_23	13	23.535
FB_0168845_L13_PA	fp13_23	13	23.535
FB_0168843_L13_PA	fp13_23	13	23.535
FB_0172117_L13_PA	fp13_24	13	24.675

FB_0172120_L13_PA	fp13_24	13	24.675
FB_0172568_L13_PA	fp13_24	13	24.675
FB_0175134_L13_PA	fp13_24	13	24.675
GD_01776_L13_PA	fp13_24	13	24.675
FB_0172119_L13_PA	fp13_24	13	24.675
FB_0171445_L13_PA	fp13_24	13	24.675
FB_0755328_L8_PA	fp13_24	13	24.675
FB_0755309_L8_PA	fp13_24	13	24.675
FB_0173073_L13_PA	fp13_24	13	24.675
FB_0172146_L13_PA	fp13_24	13	24.675
FB_0174609_L13_PA	fp13_25	13	25.432
FB_0173072_L13_PA	fp13_25	13	25.432
FB_0174620_L13_PA	fp13_25	13	25.432
FB_0173071_L13_PA	fp13_25	13	25.432
FB_0872904_L13_28_1	fp13_25	13	25.432
FB_0175653_L13_PA	fp13_25	13	25.432
GD_01704_L13_PA	fp13_25	13	25.432
FB_0175124_L13_PA	fp13_25	13	25.432
FB_0178007_L13_PA	fp13_25	13	25.432
FB_0172145_L13_PA	fp13_25	13	25.432
GD_01507_L13_28_1	fp13_25	13	25.432
FB_0173098_L13_PA	fp13_25	13	25.432
FB_0172143_L13_PA	fp13_25	13	25.432
FB_0174614_L13_PA	fp13_26	13	26.253
FB_0174625_L13_PA	fp13_26	13	26.253
FB_0174619_L13_PA	fp13_26	13	26.253
RB_12139342_L13_PA	fp13_26	13	26.253
RB_10714013_L13_PA	fp13_26	13	26.253
FB_0175657_L13_PA	fp13_26	13	26.253
FB_0175147_L13_PA	fp13_26	13	26.253
FB_0175661_L13_PA	fp13_27	13	27.472
FB_0178008_L13_PA	fp13_27	13	27.472
FB_0175654_L13_PA	fp13_27	13	27.472
FB_0175652_L13_PA	fp13_27	13	27.472
RB_13057490_L13_PA	fp13_27	13	27.472
FB_0176316_L13_PA	fp13_28	13	28.406
FB_0176300_L13_PA	fp13_28	13	28.406
FB_0178601_L13_PA	fp13_29	13	29.248
FB_0176250_L13_PA	fp13_29	13	29.248
FB_0176246_L13_PA	fp13_29	13	29.248
FB_0179384_L13_PA	fp13_29	13	29.248
GD_00943_L13_PA	fp13_29	13	29.248
FB_0178598_L13_PA	fp13_29	13	29.248
FB_0179490_L13_PA	fp13_29	13	29.248
FB_0178588_L13_PA	fp13_29	13	29.248
FB_0179385_L13_PA	fp13_29	13	29.248

FB_0178074_L13_PA	fp13_29	13	29.248
FB_0176305_L13_PA	fp13_29	13	29.248
FB_0179496_L13_PA	fp13_29	13	29.248
FB_0179410_L13_PA	fp13_29	13	29.248
FB_0176281_L13_PA	fp13_29	13	29.248
FB_0179399_L13_PA	fp13_29	13	29.248
FB_0178080_L13_PA	fp13_29	13	29.248
FB_0176248_L13_PA	fp13_29	13	29.248
FB_0178089_L13_PA	fp13_29	13	29.248
FB_0873957_L13_32_1	fp13_29	13	29.248
FB_0178084_L13_PA	fp13_29	13	29.248
FB_0873958_L13_32_1	fp13_29	13	29.248
FB_0176288_L13_PA	fp13_29	13	29.248
RB_15707000_L13_PA	fp13_29	13	29.248
FB_0178611_L13_PA	fp13_29	13	29.248
FB_0178613_L13_PA	fp13_29	13	29.248
RB_15693091_L13_33_1	fp13_29	13	29.248
FB_0873946_L13_32_1	fp13_29	13	29.248
FB_0175634_L13_PA	fp13_29	13	29.248
FB_0180706_L13_PA	fp13_30	13	30.587
FB_0180668_L13_PA	fp13_30	13	30.587
FB_0874521_L13_33_1	fp13_30	13	30.587
FB_0874522_L13_33_1	fp13_30	13	30.587
FB_1063856_L13_57_2	fp13_30	13	30.587
GD_00390_L13_PA	fp13_30	13	30.587
FB_0875076_L13_35_1	fp13_30	13	30.587
Mald1_L13_PA	fp13_31	13	31.596
GD_00890_L13_PA	fp13_31	13	31.596
FB_0180701_L13_PA	fp13_31	13	31.596
FB_0180676_L13_PA	fp13_31	13	31.596
FB_0181277_L13_PA	fp13_31	13	31.596
RB_16737064_L13_34_1	fp13_31	13	31.596
FB_0984682_L4_24_1	fp13_31	13	31.596
FB_0180683_L13_PA	fp13_31	13	31.596
FB_0181274_L13_PA	fp13_31	13	31.596
FB_0874607_L13_34_1	fp13_31	13	31.596
FB_1063871_L13_57_2	fp13_31	13	31.596
FB_0180698_L13_PA	fp13_31	13	31.596
FB_0180266_L13_PA	fp13_32	13	32.451
FB_0875102_L13_35_1	fp13_32	13	32.451
FB_0181260_L13_PA	fp13_32	13	32.451
FB_0182727_L13_PA	fp13_32	13	32.451
FB_0181265_L13_PA	fp13_32	13	32.451
FB_0181270_L13_PA	fp13_32	13	32.451
FB_1063850_L13_57_2	fp13_32	13	32.451
FB_0874515_L13_33_1	fp13_32	13	32.451

FB_0874514_L13_33_1	fp13_32	13	32.451
GD_01844_L13_PA	fp13_32	13	32.451
FB_0984687_L4_24_1	fp13_32	13	32.451
FB_0180677_L13_PA	fp13_32	13	32.451
FB_0182710_L13_PA	fp13_32	13	32.451
FB_0181259_L13_PA	fp13_32	13	32.451
FB_0182725_L13_PA	fp13_32	13	32.451
FB_0181290_L13_PA	fp13_32	13	32.451
FB_0184869_L13_PA	fp13_32	13	32.451
FB_0183443_L13_PA	fp13_32	13	32.451
FB_0183421_L13_PA	fp13_32	13	32.451
GD_00902_L13_PA	fp13_32	13	32.451
FB_0183964_L13_PA	fp13_32	13	32.451
FB_0183947_L13_PA	fp13_32	13	32.451
FB_0183439_L13_PA	fp13_32	13	32.451
FB_0184071_L13_PA	fp13_32	13	32.451
FB_0585451_L4_PA	fp13_32	13	32.451
FB_0183966_L13_PA	fp13_32	13	32.451
FB_0183948_L13_PA	fp13_32	13	32.451
FB_0184075_L13_PA	fp13_32	13	32.451
FB_0184073_L13_PA	fp13_32	13	32.451
FB_0184834_L13_PA	fp13_33	13	33.993
FB_0184845_L13_PA	fp13_33	13	33.993
RB_19786931_L13_PA	fp13_33	13	33.993
GD_01334_L13_PA	fp13_33	13	33.993
FB_0184851_L13_PA	fp13_33	13	33.993
FB_0875074_L13_35_1	fp13_33	13	33.993
FB_0184835_L13_PA	fp13_33	13	33.993
FB_0186188_L13_PA	fp13_34	13	34.09
RB_19805064_L13_PA	fp13_35	13	35.473
FB_0184855_L13_PA	fp13_35	13	35.473
FB_1072538_L15_86_2	fp13_35	13	35.473
FB_0759967_L8_PA	fp13_36	13	36.881
GD_00532_L13_PA	fp13_37	13	37.715
RB_20181857_L13_PA	fp13_37	13	37.715
RB_20353369_L13_PA	fp13_38	13	38.892
RB_20377010_L13_PA	fp13_39	13	39.537
FB_0186190_L13_PA	fp13_39	13	39.537
FB_0186215_L13_PA	fp13_39	13	39.537
FB_0186206_L13_PA	fp13_39	13	39.537
FB_0186189_L13_PA	fp13_39	13	39.537
GD_02063_L13_PA	fp13_39	13	39.537
FB_0186196_L13_PA	fp13_39	13	39.537
FB_0186191_L13_PA	fp13_39	13	39.537
FB_0186209_L13_PA	fp13_39	13	39.537
FB_0187718_L13_PA	fp13_43	13	43.903

FB_0187723_L13_PA	fp13_43	13	43.903
FB_0195485_L13_PA	fp13_43	13	43.903
FB_0759954_L8_PA	fp13_43	13	43.903
FB_0188006_L13_PA	fp13_43	13	43.903
FB_0189856_L13_PA	fp13_43	13	43.903
FB_0189848_L13_PA	fp13_43	13	43.903
FB_0188000_L13_PA	fp13_43	13	43.903
FB_0188003_L13_PA	fp13_43	13	43.903
FB_0759960_L8_PA	fp13_43	13	43.903
FB_0759958_L8_PA	fp13_43	13	43.903
RB_23186788_L13_PA	fp13_43	13	43.903
RB_21675582_L13_PA	fp13_43	13	43.903
FB_0188005_L13_PA	fp13_44	13	44.42
FB_0188007_L13_PA	fp13_44	13	44.42
FB_0188001_L13_PA	fp13_44	13	44.42
FB_0188008_L13_PA	fp13_44	13	44.42
FB_0759955_L8_PA	fp13_44	13	44.42
RB_27872911_L13_PA	fp13_44	13	44.42
FB_0877273_L13_38_1	fp13_44	13	44.42
FB_1109827_L13_74_3	fp13_44	13	44.42
RB_21716536_L13_PA	fp13_44	13	44.42
FB_1109828_L13_74_3	fp13_44	13	44.42
FB_0189854_L13_PA	fp13_44	13	44.42
FB_0195481_L13_PA	fp13_44	13	44.42
FB_0191442_L13_PA	fp13_44	13	44.42
FB_0759961_L8_PA	fp13_45	13	45.57
FB_0188002_L13_PA	fp13_45	13	45.57
FB_0191446_L13_PA	fp13_45	13	45.57
FB_0759952_L8_PA	fp13_45	13	45.57
FB_0187733_L13_PA	fp13_45	13	45.57
FB_0187717_L13_PA	fp13_45	13	45.57
FB_0193100_L13_PA	fp13_45	13	45.57
RB_23962473_L13_PA	fp13_45	13	45.57
FB_0190892_L13_PA	fp13_45	13	45.57
FB_0195764_L13_PA	fp13_45	13	45.57
FB_0195752_L13_PA	fp13_45	13	45.57
FB_0191443_L13_PA	fp13_45	13	45.57
FB_0195744_L13_PA	fp13_45	13	45.57
FB_0190893_L13_PA	fp13_45	13	45.57
FB_0193083_L13_PA	fp13_45	13	45.57
FB_0195477_L13_PA	fp13_45	13	45.57
FB_0193084_L13_PA	fp13_45	13	45.57
FB_0195736_L13_PA	fp13_45	13	45.57
FB_0193094_L13_PA	fp13_45	13	45.57
FB_0193093_L13_PA	fp13_45	13	45.57
FB_0195480_L13_PA	fp13_45	13	45.57

FB_0191439_L13_PA	fp13_45	13	45.57
GD_00164_L13_PA	fp13_45	13	45.57
FB_0195762_L13_PA	fp13_45	13	45.57
FB_0195497_L13_PA	fp13_45	13	45.57
FB_0195478_L13_PA	fp13_45	13	45.57
FB_0190901_L13_PA	fp13_45	13	45.57
FB_0195506_L13_PA	fp13_45	13	45.57
FB_0195519_L13_PA	fp13_45	13	45.57
FB_0195737_L13_PA	fp13_45	13	45.57
GD_02333_L13_PA	fp13_45	13	45.57
FB_0196606_L13_PA	fp13_45	13	45.57
FB_0877280_L13_38_1	fp13_45	13	45.57
FB_0877272_L13_38_1	fp13_46	13	46.176
FB_0195738_L13_PA	fp13_46	13	46.176
FB_0196596_L13_PA	fp13_47	13	47.781
FB_0196607_L13_PA	fp13_47	13	47.781
FB_0196568_L13_PA	fp13_47	13	47.781
FB_0196601_L13_PA	fp13_47	13	47.781
FB_0199605_L13_PA	fp13_47	13	47.781
FB_0005460_L10_PA	fp13_48	13	48.812
FB_0199612_L13_PA	fp13_48	13	48.812
FB_0199601_L13_PA	fp13_48	13	48.812
FB_0005457_L10_PA	fp13_48	13	48.812
FB_0933510_L17_16_1	fp13_48	13	48.812
FB_0902736_L15_45_1	fp13_48	13	48.812
FB_0199599_L13_PA	fp13_48	13	48.812
FB_0366875_L16_PA	fp13_48	13	48.812
FB_0199161_L13_PA	fp13_48	13	48.812
FB_0902718_L15_45_1	fp13_48	13	48.812
FB_0902720_L15_45_1	fp13_48	13	48.812
FB_0199078_L13_PA	fp13_48	13	48.812
FB_0199079_L13_PA	fp13_48	13	48.812
FB_0199149_L13_PA	fp13_49	13	49.463
GD_01316_L13_PA	fp13_49	13	49.463
FB_0199164_L13_PA	fp13_49	13	49.463
FB_0933540_L17_16_1	fp13_49	13	49.463
FB_0933527_L17_16_1	fp13_49	13	49.463
FB_0199852_L13_PA	fp13_49	13	49.463
FB_0917911_L16_16_1	fp13_49	13	49.463
RB_31089065_L13_PA	fp13_49	13	49.463
FB_0199158_L13_PA	fp13_49	13	49.463
RB_31050547_L13_PA	fp13_49	13	49.463
FB_0933532_L17_16_1	fp13_49	13	49.463
FB_0879784_L13_46_1	fp13_49	13	49.463
FB_0933501_L17_16_1	fp13_49	13	49.463
FB_0271915_L15_PA	fp13_49	13	49.463

FB_0879783_L13_46_1	fp13_49	13	49.463
RB_31059344_L13_PA	fp13_49	13	49.463
RB_31085738_L13_PA	fp13_49	13	49.463
FB_0199163_L13_PA	fp13_49	13	49.463
FB_0917937_L16_16_1	fp13_49	13	49.463
FB_0933528_L17_16_1	fp13_49	13	49.463
FB_0199606_L13_PA	fp13_49	13	49.463
FB_0204991_L13_PA	fp13_49	13	49.463
FB_0881379_L13_46_1	fp13_49	13	49.463
FB_0570977_L4_PA	fp13_49	13	49.463
FB_0199144_L13_PA	fp13_49	13	49.463
FB_0199147_L13_PA	fp13_49	13	49.463
FB_0933502_L17_16_1	fp13_50	13	50.621
FB_0917941_L16_16_1	fp13_50	13	50.621
RB_31057207_L13_PA	fp13_50	13	50.621
FB_0198556_L13_PA	fp13_50	13	50.621
FB_0198553_L13_PA	fp13_50	13	50.621
FB_0571000_L4_PA	fp13_50	13	50.621
FB_0571003_L4_PA	fp13_50	13	50.621
FB_0197233_L13_PA	fp13_50	13	50.621
FB_0366865_L16_PA	fp13_50	13	50.621
GD_01786_L13_PA	fp13_50	13	50.621
FB_0902721_L15_45_1	fp13_50	13	50.621
FB_0571001_L4_PA	fp13_50	13	50.621
FB_0806594_L9_PA	fp13_50	13	50.621
FB_1065729_L13_67_2	fp13_50	13	50.621
FB_0205536_L13_PA	fp13_50	13	50.621
FB_0933525_L17_16_1	fp13_50	13	50.621
FB_0005458_L10_PA	fp13_50	13	50.621
FB_0806586_L9_PA	fp13_50	13	50.621
GD_02452_L13_43_1	fp13_50	13	50.621
FB_0570998_L4_PA	fp13_50	13	50.621
FB_0197195_L13_PA	fp13_50	13	50.621
FB_0933530_L17_16_1	fp13_50	13	50.621
FB_0199082_L13_PA	fp13_50	13	50.621
FB_0366862_L16_PA	fp13_50	13	50.621
FB_0205534_L13_PA	fp13_50	13	50.621
FB_0198572_L13_PA	fp13_50	13	50.621
FB_0366863_L16_PA	fp13_50	13	50.621
FB_0198554_L13_PA	fp13_50	13	50.621
FB_0366890_L16_PA	fp13_50	13	50.621
FB_1065730_L13_67_2	fp13_50	13	50.621
FB_0197217_L13_PA	fp13_50	13	50.621
FB_0208516_L13_PA	fp13_50	13	50.621
FB_0208051_L13_PA	fp13_50	13	50.621
FB_0205545_L13_PA	fp13_50	13	50.621

RB_34977942_L13_PA	fp13_51	13	51.549
FB_0881390_L13_46_1	fp13_51	13	51.549
FB_0917935_L16_16_1	fp13_51	13	51.549
FB_0879786_L13_46_1	fp13_51	13	51.549
FB_1065735_L13_67_2	fp13_51	13	51.549
FB_0879807_L13_46_1	fp13_51	13	51.549
FB_0881259_L13_46_1	fp13_51	13	51.549
GD_01899_L13_PA	fp13_51	13	51.549
FB_1065416_L13_67_2	fp13_51	13	51.549
FB_1109943_L13_75_3	fp13_51	13	51.549
FB_0204997_L13_PA	fp13_51	13	51.549
FB_0205018_L13_PA	fp13_51	13	51.549
FB_1065411_L13_67_2	fp13_51	13	51.549
FB_0881270_L13_46_1	fp13_51	13	51.549
FB_1065410_L13_67_2	fp13_51	13	51.549
GD_02890_L13_67_2	fp13_51	13	51.549
FB_0204990_L13_PA	fp13_51	13	51.549
FB_0881268_L13_46_1	fp13_51	13	51.549
FB_1075679_L16_33_2	fp13_51	13	51.549
FB_0209689_L13_PA	fp13_51	13	51.549
FB_0208534_L13_PA	fp13_51	13	51.549
FB_0908445_L15_59_1	fp13_51	13	51.549
FB_0208080_L13_PA	fp13_51	13	51.549
FB_0835207_L10_45_1	fp13_51	13	51.549
FB_0208528_L13_PA	fp13_51	13	51.549
FB_0835223_L10_45_1	fp13_51	13	51.549
FB_0208508_L13_PA	fp13_51	13	51.549
FB_1065919_L13_68_2	fp13_51	13	51.549
FB_0208520_L13_PA	fp13_51	13	51.549
FB_0208065_L13_PA	fp13_51	13	51.549
FB_0209711_L13_PA	fp13_51	13	51.549
FB_0908450_L15_59_1	fp13_51	13	51.549
FB_0835217_L10_45_1	fp13_51	13	51.549
FB_0208054_L13_PA	fp13_51	13	51.549
FB_0908449_L15_59_1	fp13_51	13	51.549
FB_0208507_L13_PA	fp13_51	13	51.549
FB_1065933_L13_68_2	fp13_51	13	51.549
RB_38238865_L13_PA	fp13_51	13	51.549
FB_0208076_L13_PA	fp13_51	13	51.549
FB_0208042_L13_PA	fp13_51	13	51.549
FB_0208524_L13_PA	fp13_52	13	52.508
FB_0902912_L15_45_1	fp13_52	13	52.508
FB_0482830_L2_PA	fp13_52	13	52.508
FB_0188842_L13_PA	fp13_52	13	52.508
FB_1045533_L9_24_1	fp13_52	13	52.508
FB_1075065_L16_31_2	fp13_52	13	52.508

FB_0124503_L12_PA	fp13_52	13	52.508
FB_0188856_L13_PA	fp13_52	13	52.508
FB_0208519_L13_PA	fp13_52	13	52.508
FB_0208522_L13_PA	fp13_52	13	52.508
FB_0806468_L9_PA	fp13_52	13	52.508
FB_0482859_L2_PA	fp13_52	13	52.508
FB_0822194_L9_PA	fp13_52	13	52.508
FB_0822195_L9_PA	fp13_52	13	52.508
FB_0482849_L2_PA	fp13_52	13	52.508
FB_0210859_L13_PA	fp13_52	13	52.508
FB_0877051_L13_37_1	fp13_53	13	53.224
FB_0210831_L13_PA	fp13_53	13	53.224
FB_0877061_L13_37_1	fp13_53	13	53.224
FB_0877071_L13_37_1	fp13_53	13	53.224
FB_0210865_L13_PA	fp13_53	13	53.224
FB_0212297_L13_PA	fp13_53	13	53.224
RB_39662008_L13_PA	fp13_53	13	53.224
FB_1058711_L11_63_2	fp13_53	13	53.224
FB_0410956_L1_PA	fp13_53	13	53.224
FB_0367674_L16_PA	fp13_53	13	53.224
FB_1083811_L2_38_2	fp13_53	13	53.224
FB_0806473_L9_PA	fp13_53	13	53.224
FB_0367331_L16_PA	fp13_53	13	53.224
FB_0367318_L16_PA	fp13_53	13	53.224
FB_0801346_L9_PA	fp13_53	13	53.224
FB_0208087_L13_PA	fp13_53	13	53.224
FB_0211719_L13_PA	fp13_53	13	53.224
FB_0055387_L11_PA	fp13_54	13	54.621
FB_0211766_L13_PA	fp13_55	13	55.116
FB_0789055_L9_PA	fp13_55	13	55.116
FB_0210830_L13_PA	fp13_55	13	55.116
FB_0209712_L13_PA	fp13_55	13	55.116
FB_0481017_L2_PA	fp13_55	13	55.116
FB_0883713_L14_27_1	fp14_00	14	0.213
FB_0212425_L14_PA	fp14_00	14	0.213
FB_0212417_L14_PA	fp14_00	14	0.213
FB_0212356_L14_PA	fp14_00	14	0.213
FB_0212348_L14_PA	fp14_00	14	0.213
FB_0212342_L14_PA	fp14_00	14	0.213
FB_0212429_L14_PA	fp14_00	14	0.213
FB_0213419_L14_PA	fp14_01	14	1.46
RB_738185_L14_PA	fp14_01	14	1.46
RB_586914_L14_PA	fp14_01	14	1.46
RB_612490_L14_PA	fp14_01	14	1.46
FB_0212691_L14_PA	fp14_01	14	1.46
FB_0212712_L14_PA	fp14_01	14	1.46

GD_01888_L14_PA	fp14_01	14	1.46
GD_01902_L14_PA	fp14_01	14	1.46
RB_741541_L14_PA	fp14_01	14	1.46
FB_0213683_L14_PA	fp14_01	14	1.46
FB_0213689_L14_PA	fp14_01	14	1.46
RB_735528_L14_PA	fp14_01	14	1.46
FB_0213692_L14_PA	fp14_01	14	1.46
RB_699123_L14_PA	fp14_01	14	1.46
FB_1066383_L14_50_2	fp14_01	14	1.46
FB_0212347_L14_PA	fp14_01	14	1.46
FB_0212427_L14_PA	fp14_01	14	1.46
FB_0883668_L14_27_1	fp14_01	14	1.46
FB_0212338_L14_PA	fp14_01	14	1.46
RB_197462_L14_PA	fp14_01	14	1.46
RB_1184953_L14_PA	fp14_02	14	2.537
FB_1066384_L14_50_2	fp14_02	14	2.537
RB_226288_L14_PA	fp14_02	14	2.537
GD_00025_L14_27_1	fp14_03	14	3.435
RB_246638_L14_PA	fp14_03	14	3.435
FB_0212690_L14_PA	fp14_03	14	3.435
FB_1066406_L14_50_2	fp14_03	14	3.435
FB_0214486_L14_PA	fp14_03	14	3.435
FB_0883727_L14_27_1	fp14_03	14	3.435
FB_0214691_L14_PA	fp14_03	14	3.435
FB_0214492_L14_PA	fp14_03	14	3.435
FB_0214504_L14_PA	fp14_03	14	3.435
FB_0214705_L14_PA	fp14_03	14	3.435
FB_0214693_L14_PA	fp14_03	14	3.435
FB_0214704_L14_PA	fp14_03	14	3.435
FB_0214700_L14_PA	fp14_03	14	3.435
RB_2130966_L14_PA	fp14_03	14	3.435
RB_3598110_L14_PA	fp14_03	14	3.435
RB_2138918_L14_PA	fp14_03	14	3.435
FB_0571554_L4_PA	fp14_04	14	4.778
FB_0571576_L4_PA	fp14_04	14	4.778
FB_0571562_L4_PA	fp14_04	14	4.778
FB_0571569_L4_PA	fp14_04	14	4.778
FB_0571593_L4_PA	fp14_04	14	4.778
FB_0571560_L4_PA	fp14_04	14	4.778
GD_01853_L14_PA	fp14_04	14	4.778
FB_0219163_L14_PA	fp14_05	14	5.405
FB_0219149_L14_PA	fp14_05	14	5.405
RB_4987187_L14_PA	fp14_05	14	5.405
FB_0218297_L14_PA	fp14_05	14	5.405
RB_3601270_L14_PA	fp14_05	14	5.405
FB_0215393_L14_PA	fp14_05	14	5.405

GD_01846_L14_PA	fp14_05	14	5.405
FB_0215400_L14_PA	fp14_05	14	5.405
FB_0571558_L4_PA	fp14_05	14	5.405
FB_0215402_L14_PA	fp14_05	14	5.405
FB_0215392_L14_PA	fp14_05	14	5.405
FB_0215428_L14_PA	fp14_05	14	5.405
FB_0215417_L14_PA	fp14_05	14	5.405
FB_0220497_L14_PA	fp14_06	14	6.682
GD_01767_L14_PA	fp14_06	14	6.682
FB_0215710_L14_PA	fp14_06	14	6.682
FB_0216731_L14_PA	fp14_06	14	6.682
FB_0215701_L14_PA	fp14_06	14	6.682
FB_0215688_L14_PA	fp14_06	14	6.682
FB_0215720_L14_PA	fp14_06	14	6.682
FB_0216722_L14_PA	fp14_06	14	6.682
FB_0216744_L14_PA	fp14_06	14	6.682
FB_0216728_L14_PA	fp14_06	14	6.682
FB_0215969_L14_PA	fp14_06	14	6.682
FB_0218781_L14_PA	fp14_06	14	6.682
FB_0216751_L14_PA	fp14_06	14	6.682
FB_0218775_L14_PA	fp14_06	14	6.682
FB_0215698_L14_PA	fp14_06	14	6.682
FB_0216732_L14_PA	fp14_06	14	6.682
FB_0884877_L14_30_1	fp14_06	14	6.682
FB_0885492_L14_31_1	fp14_06	14	6.682
RB_6133421_L14_PA	fp14_07	14	7.442
GD_00248_L14_PA	fp14_07	14	7.442
GD_01401_L14_PA	fp14_07	14	7.442
RB_4973511_L14_PA	fp14_07	14	7.442
FB_0885497_L14_31_1	fp14_07	14	7.442
FB_0885507_L14_31_1	fp14_07	14	7.442
FB_0218294_L14_PA	fp14_07	14	7.442
FB_0885509_L14_31_1	fp14_07	14	7.442
FB_0885523_L14_31_1	fp14_07	14	7.442
RB_6153056_L14_PA	fp14_07	14	7.442
FB_0885521_L14_31_1	fp14_07	14	7.442
FB_0885494_L14_31_1	fp14_07	14	7.442
GD_00010_L14_PA	fp14_07	14	7.442
FB_0218311_L14_PA	fp14_07	14	7.442
FB_0885516_L14_31_1	fp14_07	14	7.442
FB_0206812_L13_PA	fp14_07	14	7.442
FB_0220496_L14_PA	fp14_07	14	7.442
FB_0207486_L13_PA	fp14_07	14	7.442
FB_0206806_L13_PA	fp14_07	14	7.442
FB_0594524_L5_PA	fp14_07	14	7.442
FB_0594515_L5_PA	fp14_07	14	7.442

FB_0221140_L14_PA	fp14_07	14	7.442
RB_2076283_L14_PA	fp14_07	14	7.442
RB_6130026_L14_PA	fp14_07	14	7.442
FB_0206805_L13_PA	fp14_07	14	7.442
RB_6168713_L14_PA	fp14_07	14	7.442
FB_0220498_L14_PA	fp14_07	14	7.442
RB_6465741_L14_PA	fp14_07	14	7.442
FB_0886144_L14_31_1	fp14_08	14	8.518
FB_0221135_L14_PA	fp14_08	14	8.518
FB_0886186_L14_31_1	fp14_08	14	8.518
FB_0206810_L13_PA	fp14_08	14	8.518
FB_0221139_L14_PA	fp14_08	14	8.518
FB_0221151_L14_PA	fp14_08	14	8.518
FB_0886152_L14_31_1	fp14_08	14	8.518
FB_0190582_L13_PA	fp14_08	14	8.518
FB_0206807_L13_PA	fp14_08	14	8.518
FB_0221133_L14_PA	fp14_08	14	8.518
FB_0886185_L14_31_1	fp14_09	14	9.523
FB_1019917_L7_48_1	fp14_09	14	9.523
FB_1019925_L7_48_1	fp14_09	14	9.523
FB_0221145_L14_PA	fp14_09	14	9.523
RB_8280462_L14_32_1	fp14_10	14	10.424
FB_0222992_L14_PA	fp14_11	14	11.551
FB_1066937_L14_55_2	fp14_11	14	11.551
FB_1066954_L14_55_2	fp14_11	14	11.551
FB_1066933_L14_55_2	fp14_11	14	11.551
RB_9067765_L14_PA	fp14_11	14	11.551
FB_1066936_L14_55_2	fp14_11	14	11.551
FB_0224648_L14_PA	fp14_12	14	12.715
FB_0224646_L14_PA	fp14_12	14	12.715
FB_0226601_L14_PA	fp14_12	14	12.715
RB_9077395_L14_PA	fp14_12	14	12.715
FB_1073522_L15_94_2	fp14_12	14	12.715
FB_0226596_L14_PA	fp14_12	14	12.715
FB_0224659_L14_PA	fp14_12	14	12.715
GD_01650_L14_32_1	fp14_12	14	12.715
FB_0226603_L14_PA	fp14_12	14	12.715
RB_9119037_L14_32_1	fp14_12	14	12.715
RB_9112020_L14_32_1	fp14_12	14	12.715
RB_9141829_L14_32_1	fp14_12	14	12.715
FB_0226599_L14_PA	fp14_12	14	12.715
FB_0224649_L14_PA	fp14_13	14	13.67
RB_11386591_L14_PA	fp14_13	14	13.67
FB_0230746_L14_PA	fp14_13	14	13.67
FB_0232243_L14_PA	fp14_14	14	14.579
RB_12582508_L14_PA	fp14_14	14	14.579

RB_12456572_L14_PA	fp14_14	14	14.579
FB_0888218_L14_36_1	fp14_14	14	14.579
FB_0233257_L14_PA	fp14_14	14	14.579
FB_0228550_L14_PA	fp14_14	14	14.579
FB_0228566_L14_PA	fp14_15	14	15.463
RB_12578927_L14_PA	fp14_15	14	15.463
FB_0232570_L14_PA	fp14_15	14	15.463
FB_0228549_L14_PA	fp14_15	14	15.463
FB_1067584_L14_58_2	fp14_15	14	15.463
FB_0228731_L14_PA	fp14_15	14	15.463
RB_12558583_L14_PA	fp14_15	14	15.463
GD_00170_L14_PA	fp14_15	14	15.463
FB_0228733_L14_PA	fp14_15	14	15.463
FB_0228559_L14_PA	fp14_15	14	15.463
FB_0228728_L14_PA	fp14_15	14	15.463
FB_0232201_L14_PA	fp14_15	14	15.463
FB_0228551_L14_PA	fp14_15	14	15.463
FB_0230780_L14_PA	fp14_15	14	15.463
FB_0231494_L14_PA	fp14_15	14	15.463
FB_0228555_L14_PA	fp14_15	14	15.463
FB_0230748_L14_PA	fp14_15	14	15.463
FB_0231503_L14_PA	fp14_16	14	16.585
FB_0232578_L14_PA	fp14_16	14	16.585
FB_0232565_L14_PA	fp14_16	14	16.585
FB_0231535_L14_PA	fp14_16	14	16.585
FB_0232191_L14_PA	fp14_16	14	16.585
FB_0764349_L8_PA	fp14_16	14	16.585
FB_0233260_L14_PA	fp14_16	14	16.585
FB_0233281_L14_PA	fp14_16	14	16.585
FB_0233865_L14_PA	fp14_16	14	16.585
FB_0233286_L14_PA	fp14_16	14	16.585
FB_0233283_L14_PA	fp14_16	14	16.585
FB_0233294_L14_PA	fp14_16	14	16.585
GD_00259L14_PA	fp14_16	14	16.585
FB_0233852_L14_PA	fp14_16	14	16.585
FB_0228749_L14_PA	fp14_16	14	16.585
FB_0233288_L14_PA	fp14_16	14	16.585
FB_0233245_L14_PA	fp14_17	14	17.321
RB_16037281_L14_PA	fp14_17	14	17.321
FB_0443119_L1_PA	fp14_17	14	17.321
FB_0233849_L14_PA	fp14_17	14	17.321
FB_1030188_L8_41_1	fp14_17	14	17.321
FB_1062992_L13_50_2	fp14_18	14	18.841
FB_1062997_L13_50_2	fp14_18	14	18.841
FB_1063003_L13_50_2	fp14_18	14	18.841
FB_1030166_L8_41_1	fp14_19	14	19.931

FB_1063022_L13_50_2	fp14_20	14	20.645
FB_1030164_L8_41_1	fp14_20	14	20.645
FB_0247243_L14_PA	fp14_20	14	20.645
FB_1030165_L8_41_1	fp14_20	14	20.645
FB_1030171_L8_41_1	fp14_20	14	20.645
FB_1030185_L8_41_1	fp14_20	14	20.645
FB_0233860_L14_PA	fp14_20	14	20.645
FB_1030192_L8_41_1	fp14_20	14	20.645
FB_0232571_L14_PA	fp14_21	14	21.285
FB_0411694_L1_PA	fp14_21	14	21.285
FB_0238391_L14_PA	fp14_21	14	21.285
FB_0231507_L14_PA	fp14_21	14	21.285
FB_0238394_L14_PA	fp14_21	14	21.285
FB_0443141_L1_PA	fp14_21	14	21.285
FB_0443127_L1_PA	fp14_21	14	21.285
FB_0242018_L14_PA	fp14_21	14	21.285
FB_0237657_L14_PA	fp14_21	14	21.285
FB_0643911_L5_PA	fp14_21	14	21.285
FB_0240859_L14_PA	fp14_22	14	22.458
FB_0228563_L14_PA	fp14_22	14	22.458
RB_21013914_L14_PA	fp14_22	14	22.458
FB_0240847_L14_PA	fp14_22	14	22.458
FB_0238390_L14_PA	fp14_22	14	22.458
FB_0238382_L14_PA	fp14_22	14	22.458
FB_0443117_L1_PA	fp14_22	14	22.458
FB_0240837_L14_PA	fp14_22	14	22.458
RB_20380789_L14_41_1	fp14_22	14	22.458
RB_18386236_L14_PA	fp14_22	14	22.458
FB_0240850_L14_PA	fp14_22	14	22.458
FB_0239447_L14_PA	fp14_22	14	22.458
RB_18388411_L14_PA	fp14_22	14	22.458
FB_0238398_L14_PA	fp14_22	14	22.458
FB_0236531_L14_PA	fp14_22	14	22.458
FB_0238832_L14_PA	fp14_22	14	22.458
FB_0236522_L14_PA	fp14_22	14	22.458
FB_0236541_L14_PA	fp14_22	14	22.458
FB_0236535_L14_PA	fp14_22	14	22.458
FB_0236520_L14_PA	fp14_22	14	22.458
GD_02426_L14_PA	fp14_22	14	22.458
FB_0236526_L14_PA	fp14_22	14	22.458
FB_0241198_L14_PA	fp14_22	14	22.458
FB_0239459_L14_PA	fp14_22	14	22.458
RB_21190967_L14_PA	fp14_22	14	22.458
FB_0241200_L14_PA	fp14_22	14	22.458
RB_20371627_L14_PA	fp14_23	14	23.373
RB_20343075_L14_PA	fp14_23	14	23.373

FB_0240846_L14_PA	fp14_23	14	23.373
FB_0242008_L14_PA	fp14_23	14	23.373
FB_0240205_L14_PA	fp14_23	14	23.373
FB_0242661_L14_PA	fp14_23	14	23.373
FB_0240202_L14_PA	fp14_23	14	23.373
GD_02638_L14_41_1	fp14_23	14	23.373
FB_0240854_L14_PA	fp14_24	14	24.471
FB_0239463_L14_PA	fp14_24	14	24.471
FB_1068038_L14_61_2	fp14_24	14	24.471
FB_0239450_L14_PA	fp14_24	14	24.471
FB_0242021_L14_PA	fp14_24	14	24.471
FB_0240838_L14_PA	fp14_24	14	24.471
FB_0240834_L14_PA	fp14_24	14	24.471
GD_02363_L14_PA	fp14_24	14	24.471
FB_0242012_L14_PA	fp14_24	14	24.471
FB_0242013_L14_PA	fp14_24	14	24.471
FB_0954098_L2_24_1	fp14_24	14	24.471
FB_0242029_L14_PA	fp14_24	14	24.471
FB_0954101_L2_24_1	fp14_24	14	24.471
FB_1068112_L14_61_2	fp14_24	14	24.471
RB_21895761_L14_PA	fp14_25	14	25.795
FB_0643870_L5_PA	fp14_25	14	25.795
FB_0242643_L14_PA	fp14_25	14	25.795
FB_0643854_L5_PA	fp14_25	14	25.795
RB_21880406_L14_PA	fp14_25	14	25.795
RB_21893245_L14_PA	fp14_25	14	25.795
FB_0242659_L14_PA	fp14_26	14	26.85
FB_0643901_L5_PA	fp14_27	14	27.412
FB_0210028_L13_PA	fp14_27	14	27.412
FB_0891144_L14_41_1	fp14_27	14	27.412
FB_0242657_L14_PA	fp14_27	14	27.412
FB_0242656_L14_PA	fp14_27	14	27.412
FB_0478225_L2_PA	fp14_27	14	27.412
FB_0643855_L5_PA	fp14_27	14	27.412
FB_0242660_L14_PA	fp14_27	14	27.412
FB_0892357_L14_42_1	fp14_27	14	27.412
FB_0643861_L5_PA	fp14_27	14	27.412
FB_0242003_L14_PA	fp14_27	14	27.412
FB_0210009_L13_PA	fp14_27	14	27.412
FB_1068069_L14_61_2	fp14_27	14	27.412
FB_0643859_L5_PA	fp14_27	14	27.412
FB_0891145_L14_41_1	fp14_27	14	27.412
FB_0242651_L14_PA	fp14_27	14	27.412
FB_0241199_L14_PA	fp14_27	14	27.412
FB_1068049_L14_61_2	fp14_27	14	27.412
FB_0240849_L14_PA	fp14_27	14	27.412

FB_0478236_L2_PA	fp14_27	14	27.412
FB_0242658_L14_PA	fp14_27	14	27.412
FB_1068063_L14_61_2	fp14_27	14	27.412
FB_0500932_L2_PA	fp14_27	14	27.412
FB_0500921_L2_PA	fp14_28	14	28.631
FB_0873047_L13_29_1	fp14_28	14	28.631
FB_0174163_L13_PA	fp14_28	14	28.631
FB_0500911_L2_PA	fp14_28	14	28.631
FB_0479731_L2_PA	fp14_28	14	28.631
FB_0245115_L14_PA	fp14_28	14	28.631
FB_0065715_L11_PA	fp14_28	14	28.631
FB_0873073_L13_29_1	fp14_28	14	28.631
FB_0873065_L13_29_1	fp14_28	14	28.631
FB_0873052_L13_29_1	fp14_28	14	28.631
FB_0873060_L13_29_1	fp14_28	14	28.631
RB_23373351_L14_PA	fp14_28	14	28.631
RB_23403516_L14_62_2	fp14_28	14	28.631
RB_23443053_L14_PA	fp14_28	14	28.631
FB_0245109_L14_PA	fp14_28	14	28.631
RB_23384607_L14_PA	fp14_28	14	28.631
FB_0892354_L14_42_1	fp14_28	14	28.631
FB_0867269_L12_46_1	fp14_29	14	29.524
RB_23859882_L14_PA	fp14_29	14	29.524
FB_0245793_L14_PA	fp14_29	14	29.524
RB_23860486_L14_PA	fp14_29	14	29.524
AFL2_L14_42_1	fp14_29	14	29.524
RB_23859847_L14_PA	fp14_29	14	29.524
FB_0873056_L13_29_1	fp14_29	14	29.524
FB_0873029_L13_29_1	fp14_29	14	29.524
FB_0173726_L13_PA	fp14_29	14	29.524
FB_0478224_L2_PA	fp14_29	14	29.524
FB_0479737_L2_PA	fp14_29	14	29.524
FB_0479716_L2_PA	fp14_29	14	29.524
FB_0479723_L2_PA	fp14_29	14	29.524
FB_0479724_L2_PA	fp14_29	14	29.524
FB_0479161_L2_PA	fp14_29	14	29.524
FB_0479732_L2_PA	fp14_29	14	29.524
FB_0479162_L2_PA	fp14_29	14	29.524
RB_22410872_L14_PA	fp14_29	14	29.524
FB_0481537_L2_PA	fp14_29	14	29.524
FB_0173301_L13_PA	fp14_29	14	29.524
FB_0242950_L14_PA	fp14_29	14	29.524
FB_0243294_L14_PA	fp14_29	14	29.524
FB_0481538_L2_PA	fp14_29	14	29.524
RB_22376050_L14_PA	fp14_29	14	29.524
FB_0243307_L14_PA	fp14_29	14	29.524

RB_22379821_L14_PA	fp14_29	14	29.524
FB_0479183_L2_PA	fp14_30	14	30.356
FB_0478223_L2_PA	fp14_30	14	30.356
FB_0243300_L14_PA	fp14_30	14	30.356
FB_0151807_L12_PA	fp14_30	14	30.356
FB_0479727_L2_PA	fp14_30	14	30.356
FB_0243902_L14_PA	fp14_30	14	30.356
FB_0892346_L14_42_1	fp14_30	14	30.356
FB_0243906_L14_PA	fp14_30	14	30.356
FB_0173730_L13_PA	fp14_30	14	30.356
FB_0243305_L14_PA	fp14_30	14	30.356
FB_0481505_L2_PA	fp14_30	14	30.356
FB_0173295_L13_PA	fp14_30	14	30.356
FB_0173715_L13_PA	fp14_30	14	30.356
FB_0242970_L14_PA	fp14_30	14	30.356
RB_23343351_L14_PA	fp14_30	14	30.356
FB_0173299_L13_PA	fp14_30	14	30.356
FB_0243929_L14_PA	fp14_30	14	30.356
RB_23446212_L14_PA	fp14_30	14	30.356
FB_0892351_L14_42_1	fp14_30	14	30.356
RB_22384593_L14_PA	fp14_30	14	30.356
FB_0242963_L14_PA	fp14_30	14	30.356
FB_0173696_L13_PA	fp14_30	14	30.356
FB_0867284_L12_46_1	fp14_30	14	30.356
FB_0481522_L2_PA	fp14_30	14	30.356
FB_0173317_L13_PA	fp14_30	14	30.356
FB_0173329_L13_PA	fp14_30	14	30.356
FB_0151805_L12_PA	fp14_30	14	30.356
FB_0242958_L14_PA	fp14_30	14	30.356
FB_0243916_L14_PA	fp14_31	14	31.798
FB_0245792_L14_PA	fp14_31	14	31.798
FB_0245107_L14_PA	fp14_31	14	31.798
FB_1068591_L14_62_2	fp14_31	14	31.798
FB_0245791_L14_PA	fp14_31	14	31.798
FB_0247544_L14_PA	fp14_31	14	31.798
FB_0247159_L14_PA	fp14_31	14	31.798
GD_00453_L14_PA	fp14_31	14	31.798
FB_0247522_L14_PA	fp14_31	14	31.798
FB_0893025_L14_45_1	fp14_31	14	31.798
FB_0246020_L14_PA	fp14_31	14	31.798
RB_24054772_L14_PA	fp14_31	14	31.798
FB_0247179_L14_PA	fp14_31	14	31.798
FB_0246609_L14_PA	fp14_31	14	31.798
FB_0245800_L14_PA	fp14_31	14	31.798
FB_0246614_L14_PA	fp14_31	14	31.798
FB_0247142_L14_PA	fp14_31	14	31.798

FB_0245783_L14_PA	fp14_31	14	31.798
FB_0247530_L14_PA	fp14_31	14	31.798
FB_1008282_L6_41_1	fp14_31	14	31.798
FB_0245797_L14_PA	fp14_31	14	31.798
GD_01531_L14_42_1	fp14_31	14	31.798
FB_0893029_L14_45_1	fp14_31	14	31.798
FB_0247138_L14_PA	fp14_31	14	31.798
FB_0893026_L14_45_1	fp14_31	14	31.798
RB_24800426_L14_PA	fp14_32	14	32.27
RB_24796798_L14_PA	fp14_32	14	32.27
FB_0246018_L14_PA	fp14_32	14	32.27
FB_0246461_L14_PA	fp14_32	14	32.27
RB_24046950_L14_PA	fp14_32	14	32.27
RB_24822463_L14_PA	fp14_32	14	32.27
FB_0246009_L14_PA	fp14_32	14	32.27
FB_0247181_L14_PA	fp14_32	14	32.27
RB_26127140_L14_PA	fp14_32	14	32.27
FB_0245796_L14_PA	fp14_32	14	32.27
GD_02673_L14_PA	fp14_32	14	32.27
FB_0246031_L14_PA	fp14_32	14	32.27
FB_0246452_L14_PA	fp14_32	14	32.27
FB_0246026_L14_PA	fp14_32	14	32.27
FB_1008286_L6_41_1	fp14_32	14	32.27
FB_0246590_L14_PA	fp14_32	14	32.27
FB_0893020_L14_45_1	fp14_32	14	32.27
FB_1008287_L6_41_1	fp14_32	14	32.27
FB_0247861_L14_PA	fp14_33	14	33.533
FB_0246601_L14_PA	fp14_33	14	33.533
FB_0246599_L14_PA	fp14_33	14	33.533
FB_0246592_L14_PA	fp14_33	14	33.533
FB_0246594_L14_PA	fp14_33	14	33.533
FB_0247832_L14_PA	fp14_33	14	33.533
FB_0928132_L17_13_1	fp14_34	14	34.708
FB_0247842_L14_PA	fp14_34	14	34.708
FB_0928127_L17_13_1	fp14_34	14	34.708
FB_0247867_L14_PA	fp14_34	14	34.708
FB_0247837_L14_PA	fp14_34	14	34.708
FB_0247873_L14_PA	fp14_34	14	34.708
FB_0247841_L14_PA	fp14_34	14	34.708
FB_0247848_L14_PA	fp14_34	14	34.708
FB_0247833_L14_PA	fp14_34	14	34.708
FB_1068708_L14_65_2	fp14_34	14	34.708
FB_0249091_L14_PA	fp14_35	14	35.428
FB_0249079_L14_PA	fp14_35	14	35.428
FB_0674168_L6_PA	fp14_35	14	35.428
RB_26872853_L14_46_1	fp14_35	14	35.428

RB_26884307_L14_PA	fp14_35	14	35.428
FB_0251453_L14_PA	fp14_35	14	35.428
FB_0674147_L6_PA	fp14_36	14	36.4
FB_0674140_L6_PA	fp14_36	14	36.4
FB_0249111_L14_PA	fp14_37	14	37.411
FB_0249098_L14_PA	fp14_37	14	37.411
FB_0249063_L14_PA	fp14_37	14	37.411
FB_0249076_L14_PA	fp14_37	14	37.411
FB_0249112_L14_PA	fp14_37	14	37.411
FB_0249704_L14_PA	fp14_37	14	37.411
FB_0249069_L14_PA	fp14_37	14	37.411
FB_0893615_L14_46_1	fp14_38	14	38.269
FB_0893588_L14_46_1	fp14_38	14	38.269
FB_0893621_L14_46_1	fp14_38	14	38.269
FB_0893589_L14_46_1	fp14_38	14	38.269
FB_0893599_L14_46_1	fp14_38	14	38.269
FB_0893591_L14_46_1	fp14_38	14	38.269
FB_0893604_L14_46_1	fp14_38	14	38.269
FB_0893593_L14_46_1	fp14_38	14	38.269
FB_1068707_L14_65_2	fp14_38	14	38.269
FB_0252933_L14_PA	fp14_38	14	38.269
FB_0894766_L14_47_1	fp14_38	14	38.269
FB_0251413_L14_PA	fp14_38	14	38.269
FB_0249691_L14_PA	fp14_39	14	39.805
FB_0249681_L14_PA	fp14_39	14	39.805
RB_27605568_L14_65_2	fp14_39	14	39.805
RB_27608850_L14_65_2	fp14_39	14	39.805
FB_0894772_L14_47_1	fp14_41	14	41.542
RB_29465265_L14_PA	fp14_41	14	41.542
FB_0251436_L14_PA	fp14_41	14	41.542
FB_0251441_L14_PA	fp14_41	14	41.542
FB_0251432_L14_PA	fp14_41	14	41.542
FB_0251414_L14_PA	fp14_41	14	41.542
GD_02039_L14_47_1	fp14_41	14	41.542
FB_0251437_L14_PA	fp14_41	14	41.542
FB_0894760_L14_47_1	fp14_41	14	41.542
FB_0251435_L14_PA	fp14_41	14	41.542
FB_0251410_L14_PA	fp14_41	14	41.542
FB_0252941_L14_PA	fp14_41	14	41.542
FB_0253399_L14_PA	fp14_41	14	41.542
FB_0255209_L14_PA	fp14_42	14	42.393
RB_29481112_L14_PA	fp14_42	14	42.393
FB_0895022_L14_47_1	fp14_42	14	42.393
FB_0252229_L14_PA	fp14_42	14	42.393
FB_0252201_L14_PA	fp14_42	14	42.393
FB_0252239_L14_PA	fp14_42	14	42.393

FB_0895019_L14_47_1	fp14_42	14	42.393
FB_1069379_L14_66_2	fp14_42	14	42.393
FB_1069375_L14_66_2	fp14_42	14	42.393
FB_0895028_L14_47_1	fp14_43	14	43.465
FB_0252956_L14_PA	fp14_43	14	43.465
RB_29621172_L14_PA	fp14_43	14	43.465
FB_0253401_L14_PA	fp14_43	14	43.465
FB_0252953_L14_PA	fp14_43	14	43.465
FB_0252945_L14_PA	fp14_43	14	43.465
FB_0895641_L14_47_1	fp14_43	14	43.465
GD_01811_L14_PA	fp14_43	14	43.465
RB_29623528_L14_PA	fp14_43	14	43.465
FB_0252951_L14_PA	fp14_43	14	43.465
FB_1069380_L14_66_2	fp14_43	14	43.465
FB_0253620_L14_PA	fp14_43	14	43.465
FB_0253392_L14_PA	fp14_43	14	43.465
FB_0253389_L14_PA	fp14_43	14	43.465
FB_0253385_L14_PA	fp14_43	14	43.465
FB_1069377_L14_66_2	fp14_43	14	43.465
GD_00171_L14_PA	fp14_43	14	43.465
FB_0253407_L14_PA	fp14_43	14	43.465
RB_29528073_L14_PA	fp14_43	14	43.465
FB_0254716_L14_PA	fp14_44	14	44.293
FB_0253631_L14_PA	fp14_45	14	45.157
FB_0254158_L14_PA	fp14_45	14	45.157
FB_0253624_L14_PA	fp14_45	14	45.157
RB_30481224_L14_48_1	fp14_45	14	45.157
FB_0254161_L14_PA	fp14_45	14	45.157
FB_0253621_L14_PA	fp14_45	14	45.157
FB_0254156_L14_PA	fp14_45	14	45.157
FB_0253637_L14_PA	fp14_45	14	45.157
FB_0253627_L14_PA	fp14_45	14	45.157
FB_0253665_L14_PA	fp14_45	14	45.157
FB_0254155_L14_PA	fp14_45	14	45.157
FB_0254154_L14_PA	fp14_45	14	45.157
RB_29619098_L14_PA	fp14_45	14	45.157
RB_29660783_L14_PA	fp14_45	14	45.157
FB_0254722_L14_PA	fp14_45	14	45.157
FB_0275155_L15_PA	fp14_46	14	46.156
FB_0254699_L14_PA	fp14_47	14	47.438
FB_0254731_L14_PA	fp14_47	14	47.438
FB_0254729_L14_PA	fp14_47	14	47.438
FB_0254702_L14_PA	fp14_47	14	47.438
FB_0254713_L14_PA	fp14_49	14	49.863
FB_0255229_L14_PA	fp14_49	14	49.863
FB_0255232_L14_PA	fp14_49	14	49.863

FB_0255208_L14_PA	fp14_49	14	49.863
FB_0255205_L14_PA	fp14_49	14	49.863
FB_0255213_L14_PA	fp14_49	14	49.863
FB_0255936_L14_PA	fp14_49	14	49.863
FB_0896416_L14_48_1	fp14_51	14	51.578
FB_0256330_L14_PA	fp14_51	14	51.578
FB_0255923_L14_PA	fp14_51	14	51.578
FB_0255933_L14_PA	fp14_51	14	51.578
FB_0255922_L14_PA	fp14_51	14	51.578
FB_0255952_L14_PA	fp14_51	14	51.578
RB_31543180_L14_PA	fp14_51	14	51.578
FB_0256314_L14_PA	fp14_52	14	52.284
RB_31708508_L14_PA	fp14_52	14	52.284
RB_31661119_L14_PA	fp14_52	14	52.284
RB_31976560_L14_PA	fp14_52	14	52.284
RB_31889669_L14_PA	fp14_52	14	52.284
RB_31737586_L14_PA	fp14_52	14	52.284
GD_02079_L14_PA	fp14_52	14	52.284
GD_00213_L14_PA	fp14_52	14	52.284
FB_0256872_L14_PA	fp14_52	14	52.284
RB_31504922_L14_PA	fp14_52	14	52.284
FB_0256657_L14_PA	fp14_52	14	52.284
RB_31667231_L14_PA	fp14_52	14	52.284
RB_31516805_L14_PA	fp14_52	14	52.284
FB_0256312_L14_PA	fp14_52	14	52.284
FB_0256327_L14_PA	fp14_52	14	52.284
FB_0256329_L14_PA	fp14_52	14	52.284
FB_0256325_L14_PA	fp14_52	14	52.284
FB_0256862_L14_PA	fp14_52	14	52.284
GD_02021_L14_67_2	fp14_52	14	52.284
FB_0256675_L14_PA	fp14_52	14	52.284
FB_0256887_L14_PA	fp14_53	14	53.685
RB_33103877_L14_PA	fp14_53	14	53.685
FB_0257192_L14_PA	fp14_53	14	53.685
FB_0257620_L14_PA	fp14_54	14	54.789
GD_00023_L14_PA	fp14_54	14	54.789
GD_01639_L14_PA	fp14_54	14	54.789
FB_0257612_L14_PA	fp14_54	14	54.789
RB_33259280_L14_PA	fp14_55	14	55.528
RB_33250295_L14_PA	fp14_55	14	55.528
RB_33044188_L14_PA	fp14_55	14	55.528
GD_01548_L14_PA	fp14_55	14	55.528
FB_0257985_L14_PA	fp14_55	14	55.528
RB_33279650_L14_PA	fp14_55	14	55.528
RB_33144152_L14_PA	fp14_55	14	55.528
FB_0257988_L14_PA	fp14_55	14	55.528

RB_32977051_L14_PA	fp14_55	14	55.528
GD_00052_L14_49_1	fp14_55	14	55.528
FB_0257998_L14_PA	fp14_55	14	55.528
FB_0896841_L14_49_1	fp14_56	14	56.311
FB_0259322_L14_PA	fp14_56	14	56.311
FB_0259319_L14_PA	fp14_56	14	56.311
FB_0862419_L12_44_1	fp15_00	15	0.08
FB_0137544_L12_PA	fp15_00	15	0.08
FB_0862434_L12_44_1	fp15_00	15	0.08
FB_0137529_L12_PA	fp15_00	15	0.08
FB_0137524_L12_PA	fp15_00	15	0.08
FB_0137546_L12_PA	fp15_00	15	0.08
FB_0137538_L12_PA	fp15_00	15	0.08
FB_0137542_L12_PA	fp15_00	15	0.08
FB_0443777_L1_PA	fp15_01	15	1.197
FB_0443789_L1_PA	fp15_01	15	1.197
FB_0137790_L12_PA	fp15_01	15	1.197
FB_1061094_L12_60_2	fp15_01	15	1.197
FB_1061085_L12_60_2	fp15_01	15	1.197
FB_1061098_L12_60_2	fp15_01	15	1.197
FB_0137528_L12_PA	fp15_01	15	1.197
FB_0137548_L12_PA	fp15_01	15	1.197
FB_0259548_L15_PA	fp15_02	15	2.351
FB_0443798_L1_PA	fp15_02	15	2.351
FB_0728421_L8_PA	fp15_02	15	2.351
FB_0138059_L12_PA	fp15_02	15	2.351
FB_0138067_L12_PA	fp15_02	15	2.351
FB_0138075_L12_PA	fp15_02	15	2.351
FB_0138055_L12_PA	fp15_02	15	2.351
FB_0138077_L12_PA	fp15_02	15	2.351
FB_0138071_L12_PA	fp15_02	15	2.351
FB_0138062_L12_PA	fp15_02	15	2.351
FB_0138762_L12_PA	fp15_03	15	3.557
FB_0443799_L1_PA	fp15_04	15	4.742
FB_0728420_L8_PA	fp15_04	15	4.742
GD_00349_L15_84_2	fp15_04	15	4.742
FB_0728424_L8_PA	fp15_04	15	4.742
FB_0139307_L12_PA	fp15_04	15	4.742
FB_0260105_L15_PA	fp15_04	15	4.742
FB_0260108_L15_PA	fp15_04	15	4.742
FB_0260798_L15_PA	fp15_06	15	6.054
FB_0260942_L15_PA	fp15_06	15	6.054
RB_976750_L15_84_2	fp15_06	15	6.054
RB_1065683_L15_PA	fp15_06	15	6.054
GD_01009_L15_PA	fp15_06	15	6.054
FB_0260802_L15_PA	fp15_06	15	6.054

FB_0260805_L15_PA	fp15_06	15	6.054
FB_0260949_L15_PA	fp15_06	15	6.054
RB_1098793_L15_PA	fp15_06	15	6.054
FB_0260793_L15_PA	fp15_06	15	6.054
GD_00694_L15_44_1	fp15_06	15	6.054
RB_966107_L15_PA	fp15_06	15	6.054
FB_0260796_L15_PA	fp15_06	15	6.054
FB_0260784_L15_PA	fp15_06	15	6.054
FB_0260791_L15_PA	fp15_06	15	6.054
RB_1451208_L15_PA	fp15_07	15	7.733
FB_0261723_L15_PA	fp15_07	15	7.733
RB_1451232_L15_PA	fp15_07	15	7.733
RB_1539502_L15_PA	fp15_07	15	7.733
FB_0261743_L15_PA	fp15_07	15	7.733
FB_0261726_L15_PA	fp15_07	15	7.733
GD_00717_L15_PA	fp15_07	15	7.733
FB_0261757_L15_PA	fp15_10	15	10.707
FB_0261728_L15_PA	fp15_10	15	10.707
RB_1883712_L15_44_1	fp15_10	15	10.707
FB_0261730_L15_PA	fp15_10	15	10.707
RB_1516126_L15_PA	fp15_10	15	10.707
RB_2538403_L15_44_1	fp15_10	15	10.707
RB_1508786_L15_PA	fp15_11	15	11.351
FB_0065692_L11_PA	fp15_12	15	12.874
FB_0264674_L15_PA	fp15_12	15	12.874
FB_0264669_L15_PA	fp15_12	15	12.874
FB_0264668_L15_PA	fp15_12	15	12.874
FB_0262909_L15_PA	fp15_12	15	12.874
FB_0262942_L15_PA	fp15_12	15	12.874
RB_2558984_L15_117_3	fp15_12	15	12.874
RB_2337559_L15_PA	fp15_12	15	12.874
GD_00842_L15_44_1	fp15_12	15	12.874
FB_0262922_L15_PA	fp15_12	15	12.874
FB_0262929_L15_PA	fp15_12	15	12.874
FB_0065699_L11_PA	fp15_12	15	12.874
RB_2548564_L15_117_3	fp15_12	15	12.874
FB_0065714_L11_PA	fp15_12	15	12.874
FB_0264671_L15_PA	fp15_12	15	12.874
FB_0065707_L11_PA	fp15_12	15	12.874
FB_0065736_L11_PA	fp15_12	15	12.874
FB_0262904_L15_PA	fp15_12	15	12.874
FB_0262913_L15_PA	fp15_12	15	12.874
RB_3034625_L15_PA	fp15_14	15	14.568
FB_0264670_L15_PA	fp15_14	15	14.568
RB_3031530_L15_PA	fp15_14	15	14.568
RB_3588459_L15_PA	fp15_14	15	14.568

RB_3034504_L15_PA	fp15_14	15	14.568
RB_3055741_L15_PA	fp15_15	15	15.322
GD_01814_L15_44_1	fp15_15	15	15.322
GD_00134_L15_PA	fp15_16	15	16.945
FB_0265759_L15_PA	fp15_16	15	16.945
RB_3536720_L15_PA	fp15_16	15	16.945
FB_0266368_L15_PA	fp15_17	15	17.899
RB_4061886_L15_PA	fp15_17	15	17.899
FB_0266200_L15_PA	fp15_17	15	17.899
FB_0266602_L15_PA	fp15_17	15	17.899
FB_0266376_L15_PA	fp15_17	15	17.899
FB_0266374_L15_PA	fp15_17	15	17.899
GD_01047_L15_PA	fp15_17	15	17.899
FB_0266603_L15_PA	fp15_18	15	18.321
RB_4223857_L15_PA	fp15_18	15	18.321
FB_0266638_L15_PA	fp15_18	15	18.321
RB_4208391_L15_PA	fp15_18	15	18.321
FB_0266599_L15_PA	fp15_18	15	18.321
FB_0266632_L15_PA	fp15_18	15	18.321
RB_4212845_L15_PA	fp15_18	15	18.321
FB_0267066_L15_PA	fp15_19	15	19.546
FB_0267067_L15_PA	fp15_19	15	19.546
GD_00330_L15_PA	fp15_20	15	20.607
RB_3542415_L15_PA	fp15_20	15	20.607
RB_3458220_L15_PA	fp15_20	15	20.607
FB_0268132_L15_PA	fp15_20	15	20.607
FB_0268130_L15_PA	fp15_20	15	20.607
FB_0267854_L15_PA	fp15_20	15	20.607
RB_4957230_L15_PA	fp15_22	15	22.251
FB_0269152_L15_PA	fp15_23	15	23.32
GD_01185_L15_PA	fp15_23	15	23.32
FB_0268660_L15_PA	fp15_24	15	24.609
FB_0270357_L15_PA	fp15_24	15	24.609
FB_0269350_L15_PA	fp15_24	15	24.609
RB_5937629_L15_PA	fp15_24	15	24.609
FB_0269154_L15_PA	fp15_24	15	24.609
FB_0269150_L15_PA	fp15_24	15	24.609
RB_5934756_L15_PA	fp15_24	15	24.609
FB_0269153_L15_PA	fp15_24	15	24.609
FB_0269149_L15_PA	fp15_24	15	24.609
RB_5972769_L15_PA	fp15_24	15	24.609
FB_0269343_L15_PA	fp15_24	15	24.609
RB_5724203_L15_PA	fp15_25	15	25.239
RB_5967251_L15_PA	fp15_25	15	25.239
FB_0269355_L15_PA	fp15_25	15	25.239
FB_0269160_L15_PA	fp15_25	15	25.239

RB_5944490_L15_PA	fp15_25	15	25.239
FB_1072044_L15_84_2	fp15_26	15	26.915
FB_0269941_L15_PA	fp15_26	15	26.915
RB_6558174_L15_PA	fp15_26	15	26.915
FB_0270355_L15_PA	fp15_26	15	26.915
RB_6300357_L15_PA	fp15_27	15	27.36
RB_6636944_L15_PA	fp15_27	15	27.36
RB_6893289_L15_PA	fp15_27	15	27.36
FB_0270928_L15_PA	fp15_27	15	27.36
GD_00076_L15_44_1	fp15_27	15	27.36
FB_0270925_L15_PA	fp15_27	15	27.36
RB_6548475_L15_PA	fp15_27	15	27.36
FB_0270356_L15_PA	fp15_27	15	27.36
FB_0270352_L15_PA	fp15_27	15	27.36
FB_0270349_L15_PA	fp15_27	15	27.36
FB_1072053_L15_84_2	fp15_28	15	28.266
FB_1072052_L15_84_2	fp15_28	15	28.266
RB_6579785_L15_PA	fp15_28	15	28.266
FB_1072046_L15_84_2	fp15_28	15	28.266
FB_0269936_L15_PA	fp15_28	15	28.266
RB_6637447_L15_PA	fp15_28	15	28.266
RB_7261398_L15_84_2	fp15_28	15	28.266
RB_6589830_L15_PA	fp15_29	15	29.659
GD_00274_L15_PA	fp15_30	15	30.668
FB_1072206_L15_84_2	fp15_31	15	31.528
FB_1072204_L15_84_2	fp15_31	15	31.528
FB_0271892_L15_PA	fp15_31	15	31.528
FB_0271894_L15_PA	fp15_31	15	31.528
FB_0271890_L15_PA	fp15_31	15	31.528
FB_0271888_L15_PA	fp15_31	15	31.528
RB_7749743_L15_PA	fp15_32	15	32.398
FB_0271909_L15_PA	fp15_32	15	32.398
FB_0272639_L15_PA	fp15_33	15	33.383
FB_0272642_L15_PA	fp15_33	15	33.383
GD_01488_L15_PA	fp15_33	15	33.383
RB_7760764_L15_PA	fp15_33	15	33.383
FB_0272664_L15_PA	fp15_33	15	33.383
FB_0272637_L15_PA	fp15_33	15	33.383
RB_8129810_L15_PA	fp15_33	15	33.383
FB_0273311_L15_PA	fp15_33	15	33.383
FB_0273300_L15_PA	fp15_38	15	38.77
RB_8248084_L15_PA	fp15_38	15	38.77
FB_0273317_L15_PA	fp15_38	15	38.77
FB_0273322_L15_PA	fp15_38	15	38.77
RB_8101776_L15_PA	fp15_38	15	38.77
FB_0273303_L15_PA	fp15_38	15	38.77

FB_0273327_L15_PA	fp15_38	15	38.77
FB_0275556_L15_PA	fp15_39	15	39.607
RB_8352817_L15_PA	fp15_39	15	39.607
FB_0274558_L15_PA	fp15_39	15	39.607
GD_00396_L15_PA	fp15_39	15	39.607
GD_00796_L15_PA	fp15_39	15	39.607
FB_0273786_L15_PA	fp15_39	15	39.607
FB_1072335_L15_84_2	fp15_39	15	39.607
FB_0273784_L15_PA	fp15_39	15	39.607
FB_0274563_L15_PA	fp15_39	15	39.607
FB_0273798_L15_PA	fp15_39	15	39.607
FB_0274557_L15_PA	fp15_39	15	39.607
FB_0273810_L15_PA	fp15_39	15	39.607
RB_8718910_L15_PA	fp15_39	15	39.607
FB_0273783_L15_PA	fp15_39	15	39.607
FB_1072285_L15_84_2	fp15_39	15	39.607
FB_1072289_L15_84_2	fp15_39	15	39.607
FB_1072304_L15_84_2	fp15_39	15	39.607
FB_0274556_L15_PA	fp15_39	15	39.607
FB_0274564_L15_PA	fp15_39	15	39.607
FB_0273789_L15_PA	fp15_39	15	39.607
FB_0273811_L15_PA	fp15_39	15	39.607
FB_1072287_L15_84_2	fp15_39	15	39.607
FB_0273800_L15_PA	fp15_39	15	39.607
GD_00894_L15_PA	fp15_39	15	39.607
FB_0273819_L15_PA	fp15_39	15	39.607
FB_1072308_L15_84_2	fp15_39	15	39.607
FB_0275986_L15_PA	fp15_40	15	40.452
FB_0276002_L15_PA	fp15_40	15	40.452
FB_0727918_L8_PA	fp15_40	15	40.452
FB_0276003_L15_PA	fp15_40	15	40.452
FB_0727900_L8_PA	fp15_40	15	40.452
FB_0275561_L15_PA	fp15_40	15	40.452
FB_0727898_L8_PA	fp15_40	15	40.452
FB_0277085_L15_PA	fp15_40	15	40.452
FB_0277113_L15_PA	fp15_40	15	40.452
FB_0275558_L15_PA	fp15_40	15	40.452
FB_0277091_L15_PA	fp15_40	15	40.452
FB_0276005_L15_PA	fp15_40	15	40.452
FB_0727892_L8_PA	fp15_40	15	40.452
FB_0727903_L8_PA	fp15_40	15	40.452
RB_9838626_L15_PA	fp15_40	15	40.452
FB_0727924_L8_PA	fp15_40	15	40.452
FB_0727928_L8_PA	fp15_40	15	40.452
FB_0275147_L15_PA	fp15_40	15	40.452
FB_0275139_L15_PA	fp15_40	15	40.452

FB_0277107_L15_PA	fp15_40	15	40.452
FB_0276012_L15_PA	fp15_40	15	40.452
FB_0277093_L15_PA	fp15_40	15	40.452
FB_0275136_L15_PA	fp15_40	15	40.452
FB_0275156_L15_PA	fp15_40	15	40.452
FB_0275552_L15_PA	fp15_40	15	40.452
GD_00543_L15_PA	fp15_40	15	40.452
FB_0277138_L15_PA	fp15_40	15	40.452
FB_0277090_L15_PA	fp15_40	15	40.452
FB_0274562_L15_PA	fp15_40	15	40.452
RB_8730844_L15_PA	fp15_40	15	40.452
FB_0277111_L15_PA	fp15_40	15	40.452
FB_0903357_L15_46_1	fp15_41	15	41.695
FB_0277882_L15_PA	fp15_41	15	41.695
FB_0277871_L15_PA	fp15_41	15	41.695
RB_11070621_L15_PA	fp15_41	15	41.695
RB_11074629_L15_PA	fp15_42	15	42.588
FB_0277864_L15_PA	fp15_42	15	42.588
FB_0277885_L15_PA	fp15_42	15	42.588
RB_11082663_L15_PA	fp15_42	15	42.588
FB_0279013_L15_PA	fp15_45	15	45.402
RB_11858982_L15_PA	fp15_45	15	45.402
FB_0281963_L15_PA	fp15_45	15	45.402
GD_01146_L15_PA	fp15_45	15	45.402
FB_0279019_L15_PA	fp15_45	15	45.402
RB_11859098_L15_PA	fp15_45	15	45.402
FB_0903830_L15_46_1	fp15_45	15	45.402
FB_0279012_L15_PA	fp15_45	15	45.402
FB_0281408_L15_PA	fp15_45	15	45.402
GD_01587_L15_PA	fp15_45	15	45.402
FB_0280082_L15_PA	fp15_45	15	45.402
RB_12572371_L15_PA	fp15_45	15	45.402
FB_0282008_L15_PA	fp15_45	15	45.402
RB_12159735_L15_PA	fp15_45	15	45.402
GD_00397_L15_47_1	fp15_45	15	45.402
RB_14680263_L15_PA	fp15_46	15	46.136
FB_0281409_L15_PA	fp15_46	15	46.136
FB_0283084_L15_PA	fp15_46	15	46.136
FB_1011101_L6_43_1	fp15_46	15	46.136
FB_1011103_L6_43_1	fp15_46	15	46.136
FB_0281958_L15_PA	fp15_46	15	46.136
FB_1011104_L6_43_1	fp15_46	15	46.136
FB_0903826_L15_46_1	fp15_46	15	46.136
FB_0281446_L15_PA	fp15_46	15	46.136
GD_00429_L15_PA	fp15_46	15	46.136
FB_0281404_L15_PA	fp15_46	15	46.136

FB_1011096_L6_43_1	fp15_46	15	46.136
FB_0283075_L15_PA	fp15_46	15	46.136
FB_0903835_L15_46_1	fp15_46	15	46.136
FB_0283095_L15_PA	fp15_46	15	46.136
GD_02003_L15_PA	fp15_46	15	46.136
RB_14726709_L15_49_1	fp15_46	15	46.136
RB_11858781_L15_PA	fp15_46	15	46.136
FB_0281418_L15_PA	fp15_46	15	46.136
FB_0283069_L15_PA	fp15_46	15	46.136
FB_0283091_L15_PA	fp15_46	15	46.136
FB_0283067_L15_PA	fp15_46	15	46.136
RB_12099465_L15_PA	fp15_46	15	46.136
FB_0281967_L15_PA	fp15_46	15	46.136
FB_0281962_L15_PA	fp15_46	15	46.136
FB_0280070_L15_PA	fp15_46	15	46.136
FB_0280058_L15_PA	fp15_46	15	46.136
GD_00816_L15_PA	fp15_47	15	47.274
FB_0281970_L15_PA	fp15_47	15	47.274
FB_0284921_L15_PA	fp15_47	15	47.274
FB_0206522_L13_PA	fp15_47	15	47.274
FB_0284922_L15_PA	fp15_47	15	47.274
GD_00273_L15_PA	fp15_47	15	47.274
FB_0284925_L15_PA	fp15_47	15	47.274
FB_0284928_L15_PA	fp15_47	15	47.274
RB_15046874_L15_PA	fp15_47	15	47.274
FB_0284918_L15_PA	fp15_47	15	47.274
RB_15755494_L15_PA	fp15_47	15	47.274
FB_0281399_L15_PA	fp15_47	15	47.274
FB_1011112_L6_43_1	fp15_47	15	47.274
FB_0118846_L12_PA	fp15_47	15	47.274
FB_0281413_L15_PA	fp15_47	15	47.274
FB_0283098_L15_PA	fp15_47	15	47.274
FB_0284942_L15_PA	fp15_48	15	48.733
FB_0285536_L15_PA	fp15_49	15	49.126
FB_0285871_L15_PA	fp15_49	15	49.126
FB_0285869_L15_PA	fp15_49	15	49.126
FB_0285528_L15_PA	fp15_49	15	49.126
FB_0285541_L15_PA	fp15_49	15	49.126
RB_16440305_L15_PA	fp15_49	15	49.126
FB_0285527_L15_PA	fp15_49	15	49.126
RB_16491350_L15_PA	fp15_50	15	50.493
FB_0287797_L15_PA	fp15_51	15	51.215
FB_0287796_L15_PA	fp15_51	15	51.215
FB_0287791_L15_PA	fp15_51	15	51.215
FB_0287926_L15_PA	fp15_52	15	52.723
FB_0287933_L15_PA	fp15_52	15	52.723

FB_0287920_L15_PA	fp15_52	15	52.723
FB_0286363_L15_PA	fp15_52	15	52.723
FB_0288402_L15_PA	fp15_53	15	53.838
FB_0286279_L15_PA	fp15_53	15	53.838
FB_0286992_L15_PA	fp15_53	15	53.838
FB_0286285_L15_PA	fp15_53	15	53.838
FB_0286377_L15_PA	fp15_53	15	53.838
FB_0287539_L15_PA	fp15_53	15	53.838
FB_0286280_L15_PA	fp15_53	15	53.838
FB_0286354_L15_PA	fp15_53	15	53.838
RB_17583563_L15_PA	fp15_53	15	53.838
RB_17549468_L15_PA	fp15_53	15	53.838
FB_0287533_L15_PA	fp15_53	15	53.838
RB_17596372_L15_PA	fp15_53	15	53.838
FB_0286997_L15_PA	fp15_53	15	53.838
FB_0286275_L15_PA	fp15_53	15	53.838
FB_0290290_L15_PA	fp15_54	15	54.514
RB_18322083_L15_PA	fp15_54	15	54.514
RB_18324149_L15_PA	fp15_54	15	54.514
FB_0288411_L15_PA	fp15_54	15	54.514
FB_0288409_L15_PA	fp15_54	15	54.514
FB_0288403_L15_PA	fp15_54	15	54.514
RB_19064661_L15_PA	fp15_54	15	54.514
RB_19062612_L15_PA	fp15_54	15	54.514
FB_0289029_L15_PA	fp15_54	15	54.514
FB_0289002_L15_PA	fp15_54	15	54.514
RB_19049191_L15_PA	fp15_54	15	54.514
FB_0287930_L15_PA	fp15_54	15	54.514
FB_0784461_L9_PA	fp15_54	15	54.514
FB_0288397_L15_PA	fp15_54	15	54.514
FB_0906790_L15_53_1	fp15_55	15	55.724
FB_0289890_L15_PA	fp15_55	15	55.724
FB_0906912_L15_53_1	fp15_55	15	55.724
FB_0289388_L15_PA	fp15_55	15	55.724
FB_0289376_L15_PA	fp15_55	15	55.724
FB_0289385_L15_PA	fp15_55	15	55.724
FB_0289914_L15_PA	fp15_56	15	56.879
FB_0466896_L2_PA	fp15_56	15	56.879
FB_0290279_L15_PA	fp15_56	15	56.879
FB_0466901_L2_PA	fp15_56	15	56.879
FB_0627889_L5_PA	fp15_56	15	56.879
FB_0997970_L5_44_1	fp15_56	15	56.879
FB_0290261_L15_PA	fp15_56	15	56.879
FB_0997976_L5_44_1	fp15_56	15	56.879
FB_0997972_L5_44_1	fp15_56	15	56.879
GD_00615_L15_53_1	fp15_56	15	56.879

RB_20348625_L15_PA	fp15_56	15	56.879
FB_0906917_L15_53_1	fp15_57	15	57.509
FB_0289917_L15_PA	fp15_57	15	57.509
FB_0466917_L2_PA	fp15_57	15	57.509
FB_0290637_L15_PA	fp15_57	15	57.509
GD_02080_L15_PA	fp15_57	15	57.509
FB_0627892_L5_PA	fp15_57	15	57.509
RB_20367259_L15_PA	fp15_57	15	57.509
FB_0906915_L15_53_1	fp15_58	15	58.122
FB_0289913_L15_PA	fp15_58	15	58.122
FB_0291049_L15_PA	fp15_58	15	58.122
FB_0290842_L15_PA	fp15_58	15	58.122
FB_0291047_L15_PA	fp15_58	15	58.122
FB_0291077_L15_PA	fp15_58	15	58.122
FB_0907019_L15_54_1	fp15_58	15	58.122
RB_21316696_L15_PA	fp15_58	15	58.122
RB_20420907_L15_PA	fp15_58	15	58.122
FB_0291045_L15_PA	fp15_58	15	58.122
FB_0290845_L15_PA	fp15_58	15	58.122
FB_0907021_L15_54_1	fp15_58	15	58.122
FB_0424215_L1_PA	fp15_58	15	58.122
FB_0627900_L5_PA	fp15_58	15	58.122
FB_0291638_L15_PA	fp15_59	15	59.622
FB_0291630_L15_PA	fp15_59	15	59.622
FB_0291654_L15_PA	fp15_59	15	59.622
GD_02313_L15_PA	fp15_59	15	59.622
FB_0291644_L15_PA	fp15_59	15	59.622
RB_21453078_L15_PA	fp15_59	15	59.622
FB_0907127_L15_55_1	fp15_59	15	59.622
FB_0907132_L15_55_1	fp15_59	15	59.622
FB_0907128_L15_55_1	fp15_59	15	59.622
FB_1111282_L15_122_3	fp15_59	15	59.622
RB_21481700_L15_PA	fp15_60	15	60.501
RB_21936514_L15_PA	fp15_60	15	60.501
FB_0292277_L15_PA	fp15_60	15	60.501
FB_0292264_L15_PA	fp15_60	15	60.501
FB_0292265_L15_PA	fp15_60	15	60.501
FB_0292286_L15_PA	fp15_60	15	60.501
FB_0292260_L15_PA	fp15_60	15	60.501
FB_0292284_L15_PA	fp15_60	15	60.501
RB_23563572_L15_PA	fp15_60	15	60.501
FB_0292885_L15_PA	fp15_61	15	61.735
RB_24699387_L15_58_1	fp15_61	15	61.735
FB_0295067_L15_PA	fp15_61	15	61.735
RB_26306308_L15_PA	fp15_61	15	61.735
FB_0295109_L15_PA	fp15_61	15	61.735

RB_24710442_L15_PA	fp15_61	15	61.735
FB_0908882_L15_59_1	fp15_61	15	61.735
FB_0292279_L15_PA	fp15_62	15	62.866
FB_0292874_L15_PA	fp15_62	15	62.866
FB_0294089_L15_PA	fp15_62	15	62.866
FB_0292871_L15_PA	fp15_62	15	62.866
GD_00038_L15_PA	fp15_62	15	62.866
GD_01813_L15_PA	fp15_62	15	62.866
FB_0294085_L15_PA	fp15_62	15	62.866
FB_0294071_L15_PA	fp15_62	15	62.866
FB_0294088_L15_PA	fp15_62	15	62.866
FB_0294064_L15_PA	fp15_62	15	62.866
FB_0294070_L15_PA	fp15_62	15	62.866
FB_0295121_L15_PA	fp15_62	15	62.866
RB_22160069_L15_PA	fp15_62	15	62.866
FB_0908069_L15_58_1	fp15_63	15	63.447
FB_0294092_L15_PA	fp15_63	15	63.447
FB_0295112_L15_PA	fp15_64	15	64.2
RB_22107062_L15_PA	fp15_64	15	64.2
FB_0298177_L15_PA	fp15_64	15	64.2
FB_0295736_L15_PA	fp15_64	15	64.2
FB_0298879_L15_PA	fp15_64	15	64.2
FB_0297336_L15_PA	fp15_64	15	64.2
FB_0908070_L15_58_1	fp15_64	15	64.2
FB_0298880_L15_PA	fp15_64	15	64.2
FB_1073613_L15_95_2	fp15_64	15	64.2
RB_22129590_L15_PA	fp15_64	15	64.2
FB_0298860_L15_PA	fp15_64	15	64.2
RB_22000686_L15_PA	fp15_64	15	64.2
RB_23523902_L15_PA	fp15_64	15	64.2
GD_02859_L15_58_1	fp15_64	15	64.2
FB_0908091_L15_58_1	fp15_64	15	64.2
FB_0298169_L15_PA	fp15_64	15	64.2
FB_0298187_L15_PA	fp15_64	15	64.2
FB_0908054_L15_58_1	fp15_64	15	64.2
FB_0908079_L15_58_1	fp15_64	15	64.2
GD_01850_L15_PA	fp15_64	15	64.2
RB_26289434_L15_59_1	fp15_64	15	64.2
FB_0295737_L15_PA	fp15_64	15	64.2
FB_0908058_L15_58_1	fp15_64	15	64.2
FB_1073612_L15_95_2	fp15_64	15	64.2
FB_0298181_L15_PA	fp15_64	15	64.2
FB_0297343_L15_PA	fp15_64	15	64.2
RB_22126816_L15_PA	fp15_64	15	64.2
FB_0295734_L15_PA	fp15_64	15	64.2
FB_0298182_L15_PA	fp15_64	15	64.2

FB_0298862_L15_PA	fp15_65	15	65.486
FB_0299551_L15_PA	fp15_65	15	65.486
FB_0301374_L15_PA	fp15_65	15	65.486
RB_28678775_L15_96_2	fp15_65	15	65.486
FB_0299554_L15_PA	fp15_65	15	65.486
RB_27011620_L15_PA	fp15_65	15	65.486
FB_0299547_L15_PA	fp15_66	15	66.208
FB_0908996_L15_59_1	fp15_66	15	66.208
FB_0299545_L15_PA	fp15_66	15	66.208
FB_1073866_L15_95_2	fp15_67	15	67.678
FB_1073867_L15_95_2	fp15_67	15	67.678
RB_27052178_L15_PA	fp15_67	15	67.678
FB_1073874_L15_95_2	fp15_67	15	67.678
FB_1073890_L15_95_2	fp15_67	15	67.678
FB_1073882_L15_95_2	fp15_67	15	67.678
FB_0301346_L15_PA	fp15_67	15	67.678
FB_0302304_L15_PA	fp15_67	15	67.678
GD_00208_L15_PA	fp15_67	15	67.678
FB_0300630_L15_PA	fp15_67	15	67.678
RB_27079169_L15_PA	fp15_67	15	67.678
FB_1073889_L15_95_2	fp15_67	15	67.678
RB_28693156_L15_96_2	fp15_67	15	67.678
FB_0301372_L15_PA	fp15_67	15	67.678
FB_1073869_L15_95_2	fp15_67	15	67.678
FB_0300197_L15_PA	fp15_67	15	67.678
FB_0301373_L15_PA	fp15_67	15	67.678
FB_0301349_L15_PA	fp15_67	15	67.678
FB_0300641_L15_PA	fp15_67	15	67.678
RB_27352264_L15_PA	fp15_67	15	67.678
FB_0300634_L15_PA	fp15_67	15	67.678
FB_0303060_L15_PA	fp15_67	15	67.678
FB_0909163_L15_59_1	fp15_67	15	67.678
FB_0300198_L15_PA	fp15_67	15	67.678
RB_28687459_L15_96_2	fp15_67	15	67.678
GD_02592_L15_PA	fp15_67	15	67.678
FB_0301362_L15_PA	fp15_67	15	67.678
FB_0301359_L15_PA	fp15_67	15	67.678
FB_0301353_L15_PA	fp15_67	15	67.678
FB_0302305_L15_PA	fp15_67	15	67.678
RB_27882182_L15_PA	fp15_67	15	67.678
FB_0909518_L15_59_1	fp15_67	15	67.678
FB_0409129_L17_PA	fp15_69	15	69.224
FB_0305838_L15_PA	fp15_69	15	69.224
FB_0258781_L14_PA	fp15_69	15	69.224
FB_0305605_L15_PA	fp15_69	15	69.224
FB_0977419_L4_14_1	fp15_69	15	69.224

FB_0303416_L15_PA	fp15_69	15	69.224
FB_0303054_L15_PA	fp15_69	15	69.224
FB_0305614_L15_PA	fp15_69	15	69.224
FB_0303441_L15_PA	fp15_69	15	69.224
RB_28734396_L15_PA	fp15_69	15	69.224
FB_0305826_L15_PA	fp15_69	15	69.224
FB_0306553_L15_PA	fp15_69	15	69.224
FB_0409156_L17_PA	fp15_69	15	69.224
FB_0409132_L17_PA	fp15_69	15	69.224
FB_0305613_L15_PA	fp15_69	15	69.224
GD_02219_L15_PA	fp15_69	15	69.224
FB_0306552_L15_PA	fp15_69	15	69.224
FB_0409127_L17_PA	fp15_69	15	69.224
FB_0306562_L15_PA	fp15_69	15	69.224
FB_0409141_L17_PA	fp15_69	15	69.224
FB_0786557_L9_PA	fp15_69	15	69.224
FB_0786544_L9_PA	fp15_69	15	69.224
FB_0303610_L15_PA	fp15_69	15	69.224
FB_0944900_L1_56_1	fp15_69	15	69.224
FEM_cg_6.bis	fp15_69	15	69.224
FB_0842161_L11_29_1	fp15_69	15	69.224
FB_0944896_L1_56_1	fp15_69	15	69.224
FB_0304198_L15_PA	fp15_69	15	69.224
FB_0303417_L15_PA	fp15_69	15	69.224
FB_1069204_L14_66_2	fp15_69	15	69.224
FB_0842159_L11_29_1	fp15_69	15	69.224
FB_0258761_L14_PA	fp15_69	15	69.224
FEM_cg_6	fp15_69	15	69.224
FEM_cg_5	fp15_69	15	69.224
FB_0977412_L4_14_1	fp15_69	15	69.224
FB_1069220_L14_66_2	fp15_69	15	69.224
FB_0977423_L4_14_1	fp15_69	15	69.224
FB_0786569_L9_PA	fp15_69	15	69.224
FB_0303425_L15_PA	fp15_69	15	69.224
FB_0910614_L15_61_1	fp15_69	15	69.224
FB_1069215_L14_66_2	fp15_69	15	69.224
FB_0786541_L9_PA	fp15_69	15	69.224
FB_0786571_L9_PA	fp15_69	15	69.224
FB_0977426_L4_14_1	fp15_69	15	69.224
FB_0977413_L4_14_1	fp15_69	15	69.224
FB_0785569_L9_PA	fp15_69	15	69.224
FB_0304159_L15_PA	fp15_69	15	69.224
FB_0785557_L9_PA	fp15_69	15	69.224
FB_0303413_L15_PA	fp15_69	15	69.224
FB_0829536_L10_36_1	fp15_69	15	69.224
FB_0303590_L15_PA	fp15_69	15	69.224

FB_0008291_L10_PA	fp15_69	15	69.224
FB_0829531_L10_36_1	fp15_69	15	69.224
FB_0829528_L10_36_1	fp15_69	15	69.224
FB_0258767_L14_PA	fp15_69	15	69.224
FB_0303543_L15_PA	fp15_70	15	70.696
FB_0842164_L11_29_1	fp15_70	15	70.696
FB_0116099_L12_PA	fp15_70	15	70.696
FB_0303445_L15_PA	fp15_70	15	70.696
RB_29953390_L15_PA	fp15_70	15	70.696
FB_0859922_L12_39_1	fp15_70	15	70.696
FB_0303603_L15_PA	fp15_70	15	70.696
FB_0977421_L4_14_1	fp15_70	15	70.696
FB_0307351_L15_PA	fp15_70	15	70.696
RB_33667246_L15_PA	fp15_70	15	70.696
FB_0309112_L15_PA	fp15_70	15	70.696
FB_0008294_L10_PA	fp15_70	15	70.696
FB_0309126_L15_PA	fp15_70	15	70.696
FB_0303614_L15_PA	fp15_70	15	70.696
FB_0944897_L1_56_1	fp15_71	15	71.366
RB_2521511_L15_44_1	fp15_71	15	71.366
FB_0910615_L15_61_1	fp15_71	15	71.366
FB_0305632_L15_PA	fp15_71	15	71.366
FB_0309550_L15_PA	fp15_72	15	72.24
FB_0308928_L15_PA	fp15_72	15	72.24
FB_0308010_L15_PA	fp15_72	15	72.24
FB_0309562_L15_PA	fp15_72	15	72.24
FB_0309093_L15_PA	fp15_72	15	72.24
FB_0309086_L15_PA	fp15_72	15	72.24
RB_41031897_L15_PA	fp15_72	15	72.24
FB_0308934_L15_PA	fp15_72	15	72.24
FB_0306554_L15_PA	fp15_72	15	72.24
FB_0309117_L15_PA	fp15_73	15	73.328
RB_33631031_L15_PA	fp15_73	15	73.328
FB_0309563_L15_PA	fp15_73	15	73.328
FB_0008274_L10_PA	fp15_73	15	73.328
FB_0409147_L17_PA	fp15_73	15	73.328
FB_0409151_L17_PA	fp15_73	15	73.328
RB_33627719_L15_PA	fp15_73	15	73.328
FB_0935903_L17_21_1	fp15_73	15	73.328
FB_0310355_L15_PA	fp15_73	15	73.328
FB_0935899_L17_21_1	fp15_73	15	73.328
FB_0935929_L17_21_1	fp15_74	15	74.513
FB_0935905_L17_21_1	fp15_74	15	74.513
FB_0935906_L17_21_1	fp15_74	15	74.513
FB_0008287_L10_PA	fp15_75	15	75.74
FB_0309535_L15_PA	fp15_75	15	75.74

FB_0307331_L15_PA	fp15_75	15	75.74
FB_0313994_L15_PA	fp15_75	15	75.74
GD_00357_L15_PA	fp15_75	15	75.74
FB_0308006_L15_PA	fp15_75	15	75.74
FB_0310406_L15_PA	fp15_75	15	75.74
FB_0311596_L15_PA	fp15_75	15	75.74
FB_0309568_L15_PA	fp15_75	15	75.74
FB_0307994_L15_PA	fp15_75	15	75.74
FB_0310779_L15_PA	fp15_75	15	75.74
FB_0222015_L14_PA	fp15_75	15	75.74
FB_0314292_L15_PA	fp15_75	15	75.74
FB_0309536_L15_PA	fp15_75	15	75.74
FB_0307345_L15_PA	fp15_75	15	75.74
FB_0308922_L15_PA	fp15_75	15	75.74
FB_0311607_L15_PA	fp15_75	15	75.74
FB_0310377_L15_PA	fp15_75	15	75.74
FB_0309090_L15_PA	fp15_75	15	75.74
FB_0309551_L15_PA	fp15_75	15	75.74
FB_0935898_L17_21_1	fp15_75	15	75.74
FB_0308920_L15_PA	fp15_75	15	75.74
FB_0859919_L12_39_1	fp15_75	15	75.74
FB_0222417_L14_PA	fp15_75	15	75.74
FB_0314766_L15_PA	fp15_75	15	75.74
FB_0310405_L15_PA	fp15_75	15	75.74
GD_02275_L15_PA	fp15_75	15	75.74
FB_0310353_L15_PA	fp15_75	15	75.74
FB_0310350_L15_PA	fp15_75	15	75.74
FB_0307986_L15_PA	fp15_75	15	75.74
FB_0938607_L17_23_1	fp15_75	15	75.74
FB_0222462_L14_PA	fp15_75	15	75.74
FB_0400333_L17_PA	fp15_75	15	75.74
FB_0314227_L15_PA	fp15_75	15	75.74
FB_0076529_L11_PA	fp15_75	15	75.74
FB_0911807_L15_66_1	fp15_75	15	75.74
FB_0314769_L15_PA	fp15_75	15	75.74
FB_0308919_L15_PA	fp15_75	15	75.74
FB_0217370_L14_PA	fp15_75	15	75.74
RB_40449639_L15_PA	fp15_75	15	75.74
FB_0314302_L15_PA	fp15_75	15	75.74
FB_0222428_L14_PA	fp15_75	15	75.74
FB_0314768_L15_PA	fp15_75	15	75.74
FB_0019465_L10_PA	fp15_75	15	75.74
FB_0314779_L15_PA	fp15_75	15	75.74
FB_0938628_L17_23_1	fp15_75	15	75.74
FB_0313979_L15_PA	fp15_75	15	75.74
FB_0315925_L15_PA	fp15_75	15	75.74

FB_0221986_L14_PA	fp15_75	15	75.74
FB_0314237_L15_PA	fp15_75	15	75.74
FB_0310782_L15_PA	fp15_75	15	75.74
FB_0310665_L15_PA	fp15_75	15	75.74
FB_0311625_L15_PA	fp15_76	15	76.407
FB_0935931_L17_21_1	fp15_76	15	76.407
FB_0310681_L15_PA	fp15_76	15	76.407
FB_0912273_L15_67_1	fp15_76	15	76.407
FB_0310675_L15_PA	fp15_76	15	76.407
FB_0314297_L15_PA	fp15_76	15	76.407
FB_0314218_L15_PA	fp15_76	15	76.407
FB_0312303_L15_PA	fp15_76	15	76.407
FB_0310785_L15_PA	fp15_76	15	76.407
FB_0912250_L15_67_1	fp15_76	15	76.407
FB_0315908_L15_PA	fp15_76	15	76.407
FB_0019463_L10_PA	fp15_76	15	76.407
FB_0310684_L15_PA	fp15_76	15	76.407
FB_0313973_L15_PA	fp15_76	15	76.407
FB_0912269_L15_67_1	fp15_76	15	76.407
FB_0912259_L15_67_1	fp15_76	15	76.407
FB_0312336_L15_PA	fp15_76	15	76.407
FB_0314223_L15_PA	fp15_76	15	76.407
FB_0528789_L3_PA	fp15_76	15	76.407
FB_0310799_L15_PA	fp15_76	15	76.407
FB_0217361_L14_PA	fp15_76	15	76.407
FB_0311593_L15_PA	fp15_76	15	76.407
FB_0308960_L15_PA	fp15_76	15	76.407
FB_0312319_L15_PA	fp15_76	15	76.407
FB_0019472_L10_PA	fp15_76	15	76.407
FB_0310835_L15_PA	fp15_76	15	76.407
FB_0312338_L15_PA	fp15_76	15	76.407
FB_0311602_L15_PA	fp15_76	15	76.407
FB_0912270_L15_67_1	fp15_76	15	76.407
FB_0309095_L15_PA	fp15_76	15	76.407
FB_0019467_L10_PA	fp15_76	15	76.407
FB_0310784_L15_PA	fp15_76	15	76.407
FB_0222017_L14_PA	fp15_76	15	76.407
FB_0222440_L14_PA	fp15_76	15	76.407
FB_0912247_L15_67_1	fp15_76	15	76.407
FB_0310813_L15_PA	fp15_76	15	76.407
FB_0314284_L15_PA	fp15_76	15	76.407
FB_0310680_L15_PA	fp15_76	15	76.407
FB_0912135_L15_67_1	fp15_76	15	76.407
FB_0879682_L13_45_1	fp15_76	15	76.407
FB_0311600_L15_PA	fp15_76	15	76.407
FB_0315439_L15_PA	fp15_76	15	76.407

FB_0315443_L15_PA	fp15_76	15	76.407
FB_0217358_L14_PA	fp15_76	15	76.407
FB_0912122_L15_67_1	fp15_76	15	76.407
FB_0315450_L15_PA	fp15_76	15	76.407
FB_0314295_L15_PA	fp15_76	15	76.407
FB_0315438_L15_PA	fp15_76	15	76.407
FB_0310821_L15_PA	fp15_76	15	76.407
FB_0310777_L15_PA	fp15_76	15	76.407
FB_0217374_L14_PA	fp15_76	15	76.407
FB_0222033_L14_PA	fp15_76	15	76.407
FB_0528787_L3_PA	fp15_76	15	76.407
FB_0528794_L3_PA	fp15_76	15	76.407
FB_0528783_L3_PA	fp15_76	15	76.407
RB_39870632_L15_PA	fp15_76	15	76.407
FB_0885039_L14_30_1	fp15_76	15	76.407
FB_0528781_L3_PA	fp15_76	15	76.407
FB_0912126_L15_67_1	fp15_77	15	77.606
FB_0912973_L15_71_1	fp15_77	15	77.606
FB_0993021_L5_38_1	fp15_77	15	77.606
FB_0019470_L10_PA	fp15_78	15	78.675
FB_0912966_L15_71_1	fp15_78	15	78.675
FB_0316420_L15_PA	fp15_78	15	78.675
FB_0221987_L14_PA	fp15_78	15	78.675
FB_0912274_L15_67_1	fp15_78	15	78.675
FB_0312324_L15_PA	fp15_78	15	78.675
FB_0316403_L15_PA	fp15_78	15	78.675
FB_0316437_L15_PA	fp15_78	15	78.675
FB_0993063_L5_38_1	fp15_78	15	78.675
FB_0316442_L15_PA	fp15_78	15	78.675
RB_41915853_L15_PA	fp15_78	15	78.675
RB_41915787_L15_PA	fp15_78	15	78.675
FB_0316422_L15_PA	fp15_78	15	78.675
FB_0316412_L15_PA	fp15_78	15	78.675
FB_0316410_L15_PA	fp15_78	15	78.675
FB_0316414_L15_PA	fp15_79	15	79.558
FB_0913109_L15_72_1	fp15_79	15	79.558
FB_0414694_L1_PA	fp15_79	15	79.558
FB_0743973_L8_PA	fp15_79	15	79.558
FB_0913300_L15_72_1	fp15_79	15	79.558
FB_0683490_L6_PA	fp15_79	15	79.558
FB_0906605_L15_53_1	fp15_79	15	79.558
GD_01133_L15_PA	fp15_79	15	79.558
FB_0913107_L15_72_1	fp15_79	15	79.558
FB_0317373_L15_PA	fp15_79	15	79.558
FB_0317349_L15_PA	fp15_79	15	79.558
FB_0913114_L15_72_1	fp15_79	15	79.558

FB_0913119_L15_72_1	fp15_79	15	79.558
FB_0913329_L15_72_1	fp15_79	15	79.558
FB_0317365_L15_PA	fp15_79	15	79.558
FB_0913106_L15_72_1	fp15_79	15	79.558
FB_0913110_L15_72_1	fp15_79	15	79.558
FB_0317353_L15_PA	fp15_79	15	79.558
FB_0913108_L15_72_1	fp15_79	15	79.558
FB_0317362_L15_PA	fp15_79	15	79.558
FB_0317357_L15_PA	fp15_79	15	79.558
FB_0316434_L15_PA	fp15_79	15	79.558
FB_0414059_L1_PA	fp15_79	15	79.558
FB_0743933_L8_PA	fp15_79	15	79.558
FB_0414673_L1_PA	fp15_79	15	79.558
FB_0414099_L1_PA	fp15_79	15	79.558
FB_0743994_L8_PA	fp15_79	15	79.558
FB_0467734_L2_PA	fp15_79	15	79.558
FB_0467722_L2_PA	fp15_79	15	79.558
FB_0683487_L6_PA	fp15_79	15	79.558
FB_0743934_L8_PA	fp15_79	15	79.558
FB_0743931_L8_PA	fp15_79	15	79.558
FB_0683509_L6_PA	fp15_79	15	79.558
FB_0906594_L15_53_1	fp15_79	15	79.558
FB_0906593_L15_53_1	fp15_79	15	79.558
FB_0316606_L15_PA	fp15_79	15	79.558
FB_0743943_L8_PA	fp15_79	15	79.558
FB_0683466_L6_PA	fp15_79	15	79.558
FB_0683494_L6_PA	fp15_79	15	79.558
FB_0683497_L6_PA	fp15_79	15	79.558
FB_0414665_L1_PA	fp15_80	15	80.616
FB_0316611_L15_PA	fp15_80	15	80.616
FB_0435530_L1_PA	fp15_80	15	80.616
FB_0993052_L5_38_1	fp15_80	15	80.616
FB_0320325_L15_PA	fp15_80	15	80.616
FB_0319565_L15_PA	fp15_80	15	80.616
FB_0319552_L15_PA	fp15_80	15	80.616
RB_44682057_L15_PA	fp15_80	15	80.616
FB_0914369_L15_73_1	fp15_80	15	80.616
RB_44674746_L15_PA	fp15_80	15	80.616
FB_0414063_L1_PA	fp15_81	15	81.379
FB_0414109_L1_PA	fp15_81	15	81.379
FB_1071604_L15_84_2	fp15_81	15	81.379
FB_0939340_L1_42_1	fp15_82	15	82.424
RB_45049266_L15_PA	fp15_82	15	82.424
FB_0326214_L15_PA	fp15_82	15	82.424
FB_0916683_L15_79_1	fp15_83	15	83.535
RB_51283711_L15_PA	fp15_83	15	83.535

RB_51295332_L15_PA	fp15_83	15	83.535
FB_0321072_L15_PA	fp15_83	15	83.535
FB_0363982_L16_PA	fp15_84	15	84.321
FB_0363970_L16_PA	fp15_84	15	84.321
FB_1071606_L15_84_2	fp15_84	15	84.321
FB_0634865_L5_PA	fp15_84	15	84.321
GD_01805_L15_PA	fp15_84	15	84.321
FB_0413175_L1_PA	fp15_84	15	84.321
FB_0319566_L15_PA	fp15_84	15	84.321
FB_0634869_L5_PA	fp15_84	15	84.321
FB_0914377_L15_73_1	fp15_84	15	84.321
FB_0913791_L15_73_1	fp15_84	15	84.321
FB_0913965_L15_73_1	fp15_84	15	84.321
FB_0913979_L15_73_1	fp15_84	15	84.321
FB_0634403_L5_PA	fp15_84	15	84.321
FB_0320337_L15_PA	fp15_84	15	84.321
FB_0634410_L5_PA	fp15_84	15	84.321
FB_0320327_L15_PA	fp15_84	15	84.321
FB_0320326_L15_PA	fp15_84	15	84.321
FB_0363923_L16_PA	fp15_84	15	84.321
FB_0319139_L15_PA	fp15_84	15	84.321
FB_0413188_L1_PA	fp15_84	15	84.321
RB_44333089_L15_PA	fp15_84	15	84.321
FB_0412436_L1_PA	fp15_84	15	84.321
FB_0319560_L15_PA	fp15_84	15	84.321
FB_0939336_L1_42_1	fp15_84	15	84.321
FB_0319548_L15_PA	fp15_84	15	84.321
FB_0412425_L1_PA	fp15_84	15	84.321
FB_0319554_L15_PA	fp15_84	15	84.321
FB_0319579_L15_PA	fp15_84	15	84.321
FB_0413173_L1_PA	fp15_84	15	84.321
FB_0319137_L15_PA	fp15_84	15	84.321
FB_0363964_L16_PA	fp15_84	15	84.321
FB_0322196_L15_PA	fp15_84	15	84.321
FB_0321693_L15_PA	fp15_84	15	84.321
FB_1071599_L15_84_2	fp15_84	15	84.321
FB_0363980_L16_PA	fp15_84	15	84.321
FB_0913992_L15_73_1	fp15_84	15	84.321
FB_0913967_L15_73_1	fp15_84	15	84.321
FB_0319136_L15_PA	fp15_84	15	84.321
FB_0319555_L15_PA	fp15_84	15	84.321
FB_0321070_L15_PA	fp15_84	15	84.321
FB_0322188_L15_PA	fp15_84	15	84.321
FB_1071609_L15_84_2	fp15_84	15	84.321
FB_0363999_L16_PA	fp15_85	15	85.431
FB_0913787_L15_73_1	fp15_85	15	85.431

FB_0914634_L15_73_1	fp15_85	15	85.431
FB_0042282_L10_PA	fp15_85	15	85.431
FB_0914382_L15_73_1	fp15_85	15	85.431
FB_0321056_L15_PA	fp15_85	15	85.431
FB_0323485_L15_PA	fp15_85	15	85.431
FB_0323475_L15_PA	fp15_85	15	85.431
FB_0321692_L15_PA	fp15_85	15	85.431
FB_0323484_L15_PA	fp15_85	15	85.431
FB_0324449_L15_PA	fp15_85	15	85.431
FB_0913959_L15_73_1	fp15_85	15	85.431
FB_0324477_L15_PA	fp15_86	15	86.293
FB_0042272_L10_PA	fp15_86	15	86.293
FB_0322315_L15_PA	fp15_86	15	86.293
FB_0914650_L15_73_1	fp15_86	15	86.293
FB_0322639_L15_PA	fp15_86	15	86.293
FB_0322317_L15_PA	fp15_86	15	86.293
RB_45932116_L15_PA	fp15_86	15	86.293
FB_0323470_L15_PA	fp15_86	15	86.293
FB_0322908_L15_PA	fp15_86	15	86.293
FB_0323486_L15_PA	fp15_86	15	86.293
FB_0322318_L15_PA	fp15_86	15	86.293
FB_0324445_L15_PA	fp15_86	15	86.293
FB_0323483_L15_PA	fp15_86	15	86.293
FB_0322649_L15_PA	fp15_86	15	86.293
RB_45923143_L15_PA	fp15_86	15	86.293
FB_0322323_L15_PA	fp15_86	15	86.293
RB_45910901_L15_73_1	fp15_86	15	86.293
RB_46758413_L15_PA	fp15_86	15	86.293
RB_46711004_L15_PA	fp15_86	15	86.293
FB_0914637_L15_73_1	fp15_86	15	86.293
FB_0324474_L15_PA	fp15_86	15	86.293
FB_0322640_L15_PA	fp15_86	15	86.293
FB_0322311_L15_PA	fp15_86	15	86.293
FB_0323978_L15_PA	fp15_86	15	86.293
FB_0323478_L15_PA	fp15_86	15	86.293
FB_0323965_L15_PA	fp15_86	15	86.293
FB_0323983_L15_PA	fp15_86	15	86.293
FB_0323976_L15_PA	fp15_86	15	86.293
FB_0323964_L15_PA	fp15_86	15	86.293
FB_0323967_L15_PA	fp15_86	15	86.293
FB_0323994_L15_PA	fp15_86	15	86.293
FB_0324446_L15_PA	fp15_86	15	86.293
FB_0322313_L15_PA	fp15_86	15	86.293
FB_0324471_L15_PA	fp15_87	15	87.281
FB_0323993_L15_PA	fp15_87	15	87.281
RB_46681827_L15_PA	fp15_87	15	87.281

RB_46715163_L15_PA	fp15_87	15	87.281
FB_0915569_L15_76_1	fp15_87	15	87.281
RB_49031915_L15_PA	fp15_87	15	87.281
RB_49274977_L15_PA	fp15_88	15	88.585
FB_0915589_L15_76_1	fp15_88	15	88.585
FB_0042295_L10_PA	fp15_88	15	88.585
FB_0915567_L15_76_1	fp15_88	15	88.585
FB_0915601_L15_76_1	fp15_88	15	88.585
RB_50088799_L15_PA	fp15_88	15	88.585
FB_0323476_L15_PA	fp15_88	15	88.585
FB_0915790_L15_77_1	fp15_89	15	89.397
RB_51280174_L15_78_1	fp15_89	15	89.397
FB_0326225_L15_PA	fp15_89	15	89.397
FB_0326218_L15_PA	fp15_89	15	89.397
FB_1111763_L15_134_3	fp15_89	15	89.397
FB_1111765_L15_134_3	fp15_89	15	89.397
GD_00079_L15_PA	fp15_89	15	89.397
FB_0327411_L15_PA	fp15_89	15	89.397
FB_0916689_L15_79_1	fp15_89	15	89.397
FB_0916688_L15_79_1	fp15_89	15	89.397
FB_0916681_L15_79_1	fp15_89	15	89.397
FB_0330077_L15_PA	fp15_89	15	89.397
FB_0917579_L15_83_1	fp15_90	15	90.913
FB_0330338_L15_PA	fp15_90	15	90.913
FB_0916687_L15_79_1	fp15_90	15	90.913
FB_0328736_L15_PA	fp15_90	15	90.913
RB_49250482_L15_PA	fp15_90	15	90.913
FB_0916686_L15_79_1	fp15_90	15	90.913
FB_0916776_L15_82_1	fp15_90	15	90.913
FB_0937355_L17_22_1	fp15_90	15	90.913
FB_0915575_L15_76_1	fp15_90	15	90.913
FB_0327412_L15_PA	fp15_91	15	91.533
RB_50036156_L15_PA	fp15_91	15	91.533
FB_0326223_L15_PA	fp15_91	15	91.533
FB_0327408_L15_PA	fp15_91	15	91.533
RB_49286326_L15_PA	fp15_91	15	91.533
FB_0332803_L15_PA	fp15_91	15	91.533
FB_0332800_L15_PA	fp15_91	15	91.533
FB_0329812_L15_PA	fp15_91	15	91.533
FB_0917577_L15_83_1	fp15_91	15	91.533
FB_0333013_L15_PA	fp15_91	15	91.533
RB_55338420_L15_PA	fp15_91	15	91.533
FB_0332840_L15_PA	fp15_91	15	91.533
FB_0329836_L15_PA	fp15_91	15	91.533
FB_0333035_L15_PA	fp15_91	15	91.533
FB_1079820_L17_33_2	fp15_91	15	91.533

FB_0333022_L15_PA	fp15_91	15	91.533
FB_0329820_L15_PA	fp15_91	15	91.533
FB_0333016_L15_PA	fp15_91	15	91.533
FB_0333034_L15_PA	fp15_91	15	91.533
FB_1079822_L17_33_2	fp15_91	15	91.533
FB_0333043_L15_PA	fp15_91	15	91.533
FB_0330459_L15_PA	fp15_91	15	91.533
FB_0330457_L15_PA	fp15_91	15	91.533
FB_0330309_L15_PA	fp15_91	15	91.533
FB_0330308_L15_PA	fp15_91	15	91.533
FB_0332806_L15_PA	fp15_91	15	91.533
FB_0330328_L15_PA	fp15_91	15	91.533
FB_0330303_L15_PA	fp15_91	15	91.533
FB_0330072_L15_PA	fp15_91	15	91.533
RB_51276899_L15_PA	fp15_91	15	91.533
FB_0916770_L15_82_1	fp15_91	15	91.533
GD_01392_L15_PA	fp15_91	15	91.533
RB_55332967_L15_PA	fp15_92	15	92.472
GD_00525_L15_82_1	fp15_92	15	92.472
FB_0331114_L15_PA	fp15_92	15	92.472
FB_0331127_L15_PA	fp15_92	15	92.472
GD_01778_L15_PA	fp15_92	15	92.472
FB_0916782_L15_82_1	fp15_92	15	92.472
FB_0331133_L15_PA	fp15_92	15	92.472
FB_0331125_L15_PA	fp15_92	15	92.472
FB_0917058_L15_82_1	fp15_92	15	92.472
FB_0333037_L15_PA	fp15_92	15	92.472
FB_0333038_L15_PA	fp15_92	15	92.472
FB_0332820_L15_PA	fp15_92	15	92.472
FB_0917515_L15_82_1	fp15_92	15	92.472
FB_0917423_L15_82_1	fp15_92	15	92.472
FB_0328051_L15_PA	fp15_92	15	92.472
FB_0331934_L15_PA	fp15_92	15	92.472
FB_0917424_L15_82_1	fp15_92	15	92.472
RB_54463488_L15_PA	fp15_92	15	92.472
FB_0331926_L15_PA	fp15_92	15	92.472
FB_0917495_L15_82_1	fp15_92	15	92.472
FB_0945606_L1_58_1	fp15_92	15	92.472
FB_0331919_L15_PA	fp15_92	15	92.472
FB_0906051_L15_50_1	fp15_92	15	92.472
FB_0945607_L1_58_1	fp15_92	15	92.472
RB_54500502_L15_PA	fp15_92	15	92.472
FB_0328059_L15_PA	fp15_92	15	92.472
FB_0945583_L1_58_1	fp15_92	15	92.472
FB_0328048_L15_PA	fp15_92	15	92.472
FB_0328049_L15_PA	fp15_92	15	92.472

RB_54456004_L15_PA	fp15_92	15	92.472
FB_0945611_L1_58_1	fp15_92	15	92.472
FB_0346912_L16_PA	fp16_00	16	0.203
FB_0347192_L16_PA	fp16_00	16	0.203
FB_0346906_L16_PA	fp16_00	16	0.203
RB_7417624_L16_PA	fp16_00	16	0.203
FB_0346915_L16_PA	fp16_00	16	0.203
FB_0347189_L16_PA	fp16_00	16	0.203
FB_0347588_L16_PA	fp16_00	16	0.203
FB_0347184_L16_PA	fp16_00	16	0.203
FB_0347190_L16_PA	fp16_00	16	0.203
RB_7623675_L16_PA	fp16_00	16	0.203
FB_0347198_L16_PA	fp16_00	16	0.203
FB_1052355_L10_53_2	fp16_00	16	0.203
FB_0346901_L16_PA	fp16_00	16	0.203
RB_7883793_L16_PA	fp16_00	16	0.203
FB_0347205_L16_PA	fp16_00	16	0.203
FB_0347204_L16_PA	fp16_00	16	0.203
FB_0347990_L16_PA	fp16_01	16	1.279
FB_0348126_L16_PA	fp16_01	16	1.279
FB_0348001_L16_PA	fp16_01	16	1.279
FB_0347988_L16_PA	fp16_01	16	1.279
RB_7899151_L16_PA	fp16_01	16	1.279
FB_0348117_L16_PA	fp16_01	16	1.279
FB_0348004_L16_PA	fp16_01	16	1.279
FB_1052350_L10_53_2	fp16_01	16	1.279
FB_0333549_L16_PA	fp16_02	16	2.423
FB_0334329_L16_PA	fp16_02	16	2.423
FB_0334307_L16_PA	fp16_02	16	2.423
RB_1187517_L16_PA	fp16_02	16	2.423
FB_0333569_L16_PA	fp16_02	16	2.423
FB_0335525_L16_PA	fp16_03	16	3.784
FB_0918384_L16_16_1	fp16_03	16	3.784
FB_0335348_L16_PA	fp16_03	16	3.784
RB_1225178_L16_PA	fp16_03	16	3.784
FB_0335240_L16_PA	fp16_03	16	3.784
FB_0335238_L16_PA	fp16_03	16	3.784
GD_01588_L16_PA	fp16_03	16	3.784
FB_0335338_L16_PA	fp16_03	16	3.784
FB_0335349_L16_PA	fp16_03	16	3.784
FB_0918150_L16_16_1	fp16_03	16	3.784
FB_0334926_L16_PA	fp16_04	16	4.561
RB_1617869_L16_PA	fp16_04	16	4.561
FB_0335535_L16_PA	fp16_04	16	4.561
FB_1074682_L16_31_2	fp16_04	16	4.561
RB_1538074_L16_PA	fp16_04	16	4.561

GD_01734_L16_PA	fp16_04	16	4.561
FB_0336063_L16_PA	fp16_04	16	4.561
RB_1540624_L16_PA	fp16_04	16	4.561
RB_1312612_L16_PA	fp16_04	16	4.561
FB_0336036_L16_PA	fp16_04	16	4.561
FB_0335523_L16_PA	fp16_04	16	4.561
FB_1074678_L16_31_2	fp16_04	16	4.561
RB_1452699_L16_PA	fp16_04	16	4.561
RB_1669091_L16_PA	fp16_05	16	5.388
FB_1074691_L16_31_2	fp16_05	16	5.388
FB_1074699_L16_31_2	fp16_05	16	5.388
FB_0918389_L16_16_1	fp16_05	16	5.388
RB_1571002_L16_PA	fp16_05	16	5.388
FB_0336046_L16_PA	fp16_05	16	5.388
RB_1496083_L16_PA	fp16_05	16	5.388
FB_0337045_L16_PA	fp16_05	16	5.388
FB_0337051_L16_PA	fp16_05	16	5.388
RB_1972373_L16_PA	fp16_05	16	5.388
FB_0337044_L16_PA	fp16_05	16	5.388
FB_0337042_L16_PA	fp16_06	16	6.109
FB_0338092_L16_PA	fp16_07	16	7.629
FB_1074674_L16_31_2	fp16_07	16	7.629
FB_1074747_L16_31_2	fp16_07	16	7.629
FB_1074767_L16_31_2	fp16_07	16	7.629
FB_0338093_L16_PA	fp16_08	16	8.095
FB_1074783_L16_31_2	fp16_08	16	8.095
FB_1074762_L16_31_2	fp16_08	16	8.095
FB_1074768_L16_31_2	fp16_08	16	8.095
FB_0338060_L16_PA	fp16_09	16	9.377
FB_0339055_L16_PA	fp16_09	16	9.377
RB_2419416_L16_PA	fp16_11	16	11.219
FB_1074748_L16_31_2	fp16_11	16	11.219
GD_00719_L16_PA	fp16_11	16	11.219
FB_0338085_L16_PA	fp16_11	16	11.219
FB_0338061_L16_PA	fp16_11	16	11.219
FB_0338087_L16_PA	fp16_11	16	11.219
FB_0338082_L16_PA	fp16_11	16	11.219
FB_0337023_L16_PA	fp16_12	16	12.859
FB_0339053_L16_PA	fp16_13	16	13.709
FB_1074872_L16_31_2	fp16_13	16	13.709
FB_0339062_L16_PA	fp16_13	16	13.709
FB_0339072_L16_PA	fp16_13	16	13.709
FB_1074884_L16_31_2	fp16_13	16	13.709
FB_0339052_L16_PA	fp16_13	16	13.709
RB_2593090_L16_PA	fp16_13	16	13.709
FB_0339069_L16_PA	fp16_13	16	13.709

FB_0918876_L16_16_1	fp16_13	16	13.709
GD_01116_L16_PA	fp16_14	16	14.555
FB_0339068_L16_PA	fp16_14	16	14.555
RB_2745724_L16_PA	fp16_15	16	15.394
FB_1074870_L16_31_2	fp16_15	16	15.394
FB_0340499_L16_PA	fp16_15	16	15.394
FB_0340484_L16_PA	fp16_15	16	15.394
RB_3461705_L16_PA	fp16_15	16	15.394
RB_3437104_L16_PA	fp16_15	16	15.394
RB_3193686_L16_PA	fp16_16	16	16.849
FB_0340494_L16_PA	fp16_17	16	17.172
FB_0340909_L16_PA	fp16_18	16	18.599
GD_00353_L16_PA	fp16_18	16	18.599
RB_3299822_L16_PA	fp16_18	16	18.599
RB_3377332_L16_PA	fp16_18	16	18.599
RB_3303558_L16_PA	fp16_18	16	18.599
RB_3434038_L16_PA	fp16_18	16	18.599
FB_1074919_L16_31_2	fp16_18	16	18.599
RB_3334689_L16_PA	fp16_18	16	18.599
RB_3384428_L16_PA	fp16_18	16	18.599
FB_0340501_L16_PA	fp16_18	16	18.599
RB_3465402_L16_PA	fp16_18	16	18.599
FB_1074920_L16_31_2	fp16_18	16	18.599
RB_3297840_L16_PA	fp16_18	16	18.599
FB_0340917_L16_PA	fp16_18	16	18.599
FB_0340903_L16_PA	fp16_18	16	18.599
FB_0340495_L16_PA	fp16_18	16	18.599
FB_0340916_L16_PA	fp16_19	16	19.715
RB_3756948_L16_PA	fp16_19	16	19.715
RB_3810235_L16_PA	fp16_19	16	19.715
FB_0340919_L16_PA	fp16_19	16	19.715
RB_3844766_L16_PA	fp16_19	16	19.715
RB_3777151_L16_PA	fp16_19	16	19.715
FB_0340502_L16_PA	fp16_19	16	19.715
RB_3830418_L16_PA	fp16_19	16	19.715
RB_3847368_L16_PA	fp16_19	16	19.715
FB_0340906_L16_PA	fp16_19	16	19.715
GD_01627_L16_PA	fp16_19	16	19.715
RB_3779751_L16_PA	fp16_19	16	19.715
RB_3855345_L16_PA	fp16_19	16	19.715
RB_3832708_L16_PA	fp16_19	16	19.715
RB_3988516_L16_PA	fp16_20	16	20.581
RB_3978947_L16_31_2	fp16_20	16	20.581
RB_3760217_L16_PA	fp16_20	16	20.581
RB_3937877_L16_PA	fp16_20	16	20.581
RB_3986331_L16_PA	fp16_20	16	20.581

FB_1075011_L16_31_2	fp16_22	16	22.492
FB_0341669_L16_PA	fp16_22	16	22.492
GD_01186_L16_16_1	fp16_22	16	22.492
FB_0341683_L16_PA	fp16_22	16	22.492
FB_0341673_L16_PA	fp16_22	16	22.492
FB_1075007_L16_31_2	fp16_22	16	22.492
FB_0341684_L16_PA	fp16_22	16	22.492
FB_0382351_L17_PA	fp16_22	16	22.492
RB_4201996_L16_PA	fp16_22	16	22.492
RB_4199694_L16_PA	fp16_22	16	22.492
FB_0382348_L17_PA	fp16_22	16	22.492
FB_0382342_L17_PA	fp16_22	16	22.492
FB_0341668_L16_PA	fp16_22	16	22.492
FB_1075006_L16_31_2	fp16_22	16	22.492
FB_0341666_L16_PA	fp16_22	16	22.492
FB_0382345_L17_PA	fp16_23	16	23.172
FB_0382364_L17_PA	fp16_23	16	23.172
FB_1075040_L16_31_2	fp16_24	16	24.33
FB_1075043_L16_31_2	fp16_24	16	24.33
FB_1075047_L16_31_2	fp16_24	16	24.33
FB_0343481_L16_PA	fp16_24	16	24.33
FB_0382354_L17_PA	fp16_24	16	24.33
FB_0343479_L16_PA	fp16_24	16	24.33
FB_0342929_L16_PA	fp16_24	16	24.33
FB_0342932_L16_PA	fp16_24	16	24.33
FB_0342934_L16_PA	fp16_24	16	24.33
FB_0343465_L16_PA	fp16_25	16	25.626
RB_5423990_L16_PA	fp16_25	16	25.626
RB_5434935_L16_40_3	fp16_25	16	25.626
RB_5407802_L16_PA	fp16_26	16	26.617
GD_00030_L16_PA	fp16_27	16	27.629
FB_1075289_L16_31_2	fp16_28	16	28.264
RB_5665967_L16_31_2	fp16_28	16	28.264
FB_0921308_L16_16_1	fp16_28	16	28.264
RB_5603911_L16_16_1	fp16_28	16	28.264
RB_5601568_L16_16_1	fp16_28	16	28.264
FB_0921309_L16_16_1	fp16_28	16	28.264
FB_0921295_L16_16_1	fp16_28	16	28.264
RB_5652449_L16_31_2	fp16_28	16	28.264
FB_1075309_L16_31_2	fp16_28	16	28.264
FB_1075308_L16_31_2	fp16_28	16	28.264
FB_1075282_L16_31_2	fp16_28	16	28.264
FB_1075283_L16_31_2	fp16_28	16	28.264
FB_0344904_L16_PA	fp16_29	16	29.487
FB_0344930_L16_PA	fp16_29	16	29.487
RB_6007512_L16_PA	fp16_29	16	29.487

RB_6054018_L16_PA	fp16_29	16	29.487
FB_0344910_L16_PA	fp16_29	16	29.487
FB_0344907_L16_PA	fp16_29	16	29.487
FB_0345414_L16_PA	fp16_30	16	30.089
FB_0344903_L16_PA	fp16_30	16	30.089
FB_0345431_L16_PA	fp16_30	16	30.089
FB_0345418_L16_PA	fp16_30	16	30.089
FB_0345413_L16_PA	fp16_30	16	30.089
FB_0345412_L16_PA	fp16_30	16	30.089
FB_0345411_L16_PA	fp16_30	16	30.089
FB_0345423_L16_PA	fp16_30	16	30.089
GD_01003_L16_PA	fp16_30	16	30.089
FB_0344908_L16_PA	fp16_30	16	30.089
FB_0921813_L16_16_1	fp16_31	16	31.514
RB_6537387_L16_PA	fp16_31	16	31.514
RB_6705025_L16_PA	fp16_31	16	31.514
FB_0921781_L16_16_1	fp16_32	16	32.532
FB_1075618_L16_31_2	fp16_32	16	32.532
RB_6665064_L16_PA	fp16_32	16	32.532
FB_0346036_L16_PA	fp16_32	16	32.532
FB_1075569_L16_31_2	fp16_32	16	32.532
FB_0346028_L16_PA	fp16_32	16	32.532
GD_01173_L16_PA	fp16_32	16	32.532
FB_1075561_L16_31_2	fp16_32	16	32.532
GD_01866_L16_PA	fp16_32	16	32.532
FB_1075559_L16_31_2	fp16_32	16	32.532
FB_1075562_L16_31_2	fp16_32	16	32.532
FB_0921783_L16_16_1	fp16_32	16	32.532
FB_0346046_L16_PA	fp16_32	16	32.532
FB_1075563_L16_31_2	fp16_32	16	32.532
RB_6842969_L16_PA	fp16_33	16	33.41
RB_6845933_L16_PA	fp16_33	16	33.41
FB_0569192_L4_PA	fp16_34	16	34.277
FB_0568958_L4_PA	fp16_35	16	35.143
FB_0568952_L4_PA	fp16_36	16	36.608
RB_8342463_L16_PA	fp16_36	16	36.608
RB_8348973_L16_PA	fp16_36	16	36.608
FB_0177532_L13_PA	fp16_37	16	37.407
FB_0177536_L13_PA	fp16_37	16	37.407
RB_8380997_L16_PA	fp16_37	16	37.407
FB_0348676_L16_PA	fp16_38	16	38.621
FB_0349781_L16_PA	fp16_38	16	38.621
FB_0353756_L16_PA	fp16_38	16	38.621
FB_0352364_L16_PA	fp16_38	16	38.621
FB_0349776_L16_PA	fp16_38	16	38.621
FB_0352398_L16_PA	fp16_38	16	38.621

GD_01244_L16_PA	fp16_38	16	38.621
RB_12362737_L16_PA	fp16_38	16	38.621
FB_0353770_L16_PA	fp16_38	16	38.621
RB_12364910_L16_PA	fp16_38	16	38.621
FB_0349750_L16_PA	fp16_38	16	38.621
FB_0349752_L16_PA	fp16_38	16	38.621
FB_0353763_L16_PA	fp16_38	16	38.621
FB_0349753_L16_PA	fp16_38	16	38.621
RB_12334023_L16_PA	fp16_38	16	38.621
FB_0349785_L16_PA	fp16_38	16	38.621
FB_0352365_L16_PA	fp16_38	16	38.621
FB_0352361_L16_PA	fp16_38	16	38.621
FB_0353760_L16_PA	fp16_38	16	38.621
FB_0349767_L16_PA	fp16_38	16	38.621
FB_0349751_L16_PA	fp16_38	16	38.621
FB_0568953_L4_PA	fp16_38	16	38.621
FB_0354230_L16_PA	fp16_39	16	39.849
FB_0354210_L16_PA	fp16_39	16	39.849
FB_0354226_L16_PA	fp16_39	16	39.849
FB_0354239_L16_PA	fp16_39	16	39.849
FB_0354236_L16_PA	fp16_39	16	39.849
GD_02087_L16_PA	fp16_39	16	39.849
RB_8392816_L16_PA	fp16_40	16	40.708
FB_0355100_L16_PA	fp16_40	16	40.708
RB_12359661_L16_PA	fp16_40	16	40.708
FB_0354740_L16_PA	fp16_41	16	41.34
FB_0354735_L16_PA	fp16_41	16	41.34
FB_0354768_L16_PA	fp16_41	16	41.34
FB_0354750_L16_PA	fp16_41	16	41.34
FB_0354722_L16_PA	fp16_41	16	41.34
FB_0354753_L16_PA	fp16_41	16	41.34
FB_0354770_L16_PA	fp16_41	16	41.34
FB_0354732_L16_PA	fp16_41	16	41.34
FB_0354746_L16_PA	fp16_41	16	41.34
GD_01484_L16_PA	fp16_41	16	41.34
FB_0354728_L16_PA	fp16_41	16	41.34
RB_14143772_L16_PA	fp16_41	16	41.34
FB_0924002_L16_22_1	fp16_41	16	41.34
FB_0355078_L16_PA	fp16_41	16	41.34
FB_0355076_L16_PA	fp16_41	16	41.34
RB_12329007_L16_PA	fp16_41	16	41.34
FB_0355073_L16_PA	fp16_41	16	41.34
RB_9033706_L16_PA	fp16_41	16	41.34
FB_0356440_L16_PA	fp16_41	16	41.34
RB_12850088_L16_PA	fp16_41	16	41.34
FB_0354221_L16_PA	fp16_42	16	42.383

FB_0354228_L16_PA	fp16_42	16	42.383
FB_0349772_L16_PA	fp16_42	16	42.383
RB_9090256_L16_PA	fp16_42	16	42.383
FB_0348697_L16_PA	fp16_42	16	42.383
RB_9088223_L16_PA	fp16_42	16	42.383
FB_0349124_L16_PA	fp16_42	16	42.383
RB_9092272_L16_PA	fp16_42	16	42.383
FB_0348693_L16_PA	fp16_42	16	42.383
RB_9082266_L16_PA	fp16_42	16	42.383
FB_0349778_L16_PA	fp16_42	16	42.383
FB_0349126_L16_PA	fp16_42	16	42.383
RB_9085264_L16_PA	fp16_42	16	42.383
FB_0356453_L16_PA	fp16_42	16	42.383
GD_01715_L16_19_1	fp16_42	16	42.383
FB_0923999_L16_22_1	fp16_42	16	42.383
GD_01624_L16_PA	fp16_43	16	43.508
FB_0355396_L16_PA	fp16_43	16	43.508
FB_0355403_L16_PA	fp16_43	16	43.508
FB_0356162_L16_PA	fp16_43	16	43.508
FB_0354202_L16_PA	fp16_44	16	44.444
FB_0355446_L16_PA	fp16_44	16	44.444
FB_1076328_L16_35_2	fp16_44	16	44.444
FB_1076327_L16_35_2	fp16_44	16	44.444
FB_1076333_L16_35_2	fp16_44	16	44.444
FB_1076340_L16_35_2	fp16_44	16	44.444
FB_1076355_L16_35_2	fp16_44	16	44.444
FB_1076353_L16_35_2	fp16_44	16	44.444
FB_0355441_L16_PA	fp16_44	16	44.444
FB_1076336_L16_35_2	fp16_44	16	44.444
FB_1076369_L16_35_2	fp16_44	16	44.444
FB_0355449_L16_PA	fp16_44	16	44.444
FB_0357815_L16_PA	fp16_44	16	44.444
FB_0925093_L16_22_1	fp16_44	16	44.444
FB_0356157_L16_PA	fp16_45	16	45.416
FB_0924459_L16_22_1	fp16_45	16	45.416
RB_14504645_L16_PA	fp16_45	16	45.416
FB_0924469_L16_22_1	fp16_45	16	45.416
FB_0356435_L16_PA	fp16_45	16	45.416
FB_0356160_L16_PA	fp16_45	16	45.416
RB_14645161_L16_PA	fp16_45	16	45.416
FB_0356156_L16_PA	fp16_45	16	45.416
FB_0356161_L16_PA	fp16_45	16	45.416
FB_0925084_L16_22_1	fp16_45	16	45.416
GD_00356_L16_PA	fp16_45	16	45.416
FB_1112350_L16_42_3	fp16_46	16	46.501
GD_02783_L16_PA	fp16_46	16	46.501

FB_0357371_L16_PA	fp16_47	16	47.523
FB_0357369_L16_PA	fp16_47	16	47.523
FB_0357350_L16_PA	fp16_47	16	47.523
FB_1112355_L16_42_3	fp16_47	16	47.523
FB_0357368_L16_PA	fp16_47	16	47.523
FB_0357363_L16_PA	fp16_47	16	47.523
RB_15032763_L16_PA	fp16_47	16	47.523
FB_0357356_L16_PA	fp16_47	16	47.523
FB_0793222_L9_PA	fp16_47	16	47.523
RB_15527276_L16_PA	fp16_47	16	47.523
RB_15512541_L16_PA	fp16_47	16	47.523
FB_0925086_L16_22_1	fp16_47	16	47.523
RB_15467926_L16_22_1	fp16_47	16	47.523
FB_0035909_L10_PA	fp16_47	16	47.523
FB_0358337_L16_PA	fp16_47	16	47.523
FB_0358340_L16_PA	fp16_47	16	47.523
FB_0035911_L10_PA	fp16_47	16	47.523
FB_0358336_L16_PA	fp16_47	16	47.523
FB_0924833_L16_22_1	fp16_47	16	47.523
FB_0357798_L16_PA	fp16_47	16	47.523
GD_02130_L16_PA	fp16_47	16	47.523
FB_0035922_L10_PA	fp16_47	16	47.523
FB_0924847_L16_22_1	fp16_47	16	47.523
RB_15494702_L16_PA	fp16_47	16	47.523
FB_0035910_L10_PA	fp16_47	16	47.523
FB_0035904_L10_PA	fp16_47	16	47.523
FB_0924831_L16_22_1	fp16_47	16	47.523
FB_0358866_L16_PA	fp16_47	16	47.523
FB_0684179_L6_PA	fp16_49	16	49.116
FB_1070067_L15_103_2	fp16_49	16	49.116
FB_0684212_L6_PA	fp16_49	16	49.116
FB_0793220_L9_PA	fp16_49	16	49.116
FB_0358862_L16_PA	fp16_49	16	49.116
FB_0359098_L16_PA	fp16_49	16	49.116
FB_0358842_L16_PA	fp16_49	16	49.116
FB_0684186_L6_PA	fp16_49	16	49.116
FB_0358843_L16_PA	fp16_49	16	49.116
FB_0793212_L9_PA	fp16_49	16	49.116
FB_0684182_L6_PA	fp16_49	16	49.116
FB_0359060_L16_PA	fp16_49	16	49.116
FB_0358849_L16_PA	fp16_49	16	49.116
FB_0793204_L9_PA	fp16_49	16	49.116
FB_0793202_L9_PA	fp16_49	16	49.116
FB_0358851_L16_PA	fp16_49	16	49.116
FB_1070075_L15_103_2	fp16_49	16	49.116
FB_0793221_L9_PA	fp16_49	16	49.116

FB_1070069_L15_103_2	fp16_49	16	49.116
FB_0359078_L16_PA	fp16_49	16	49.116
FB_0358841_L16_PA	fp16_49	16	49.116
FB_0360622_L16_PA	fp16_49	16	49.116
FB_0925088_L16_22_1	fp16_49	16	49.116
FB_0977570_L4_14_1	fp16_50	16	50.443
FB_0293397_L15_PA	fp16_50	16	50.443
FB_1020738_L7_48_1	fp16_50	16	50.443
FB_0925859_L16_25_1	fp16_51	16	51.802
FB_0361417_L16_PA	fp16_51	16	51.802
FB_0322938_L15_PA	fp16_51	16	51.802
RB_20428802_L16_PA	fp16_51	16	51.802
FB_0361401_L16_PA	fp16_51	16	51.802
FB_0192206_L13_PA	fp16_51	16	51.802
FB_0433621_L1_PA	fp16_51	16	51.802
FB_0018667_L10_PA	fp16_51	16	51.802
FB_0362423_L16_PA	fp16_51	16	51.802
FB_0322951_L15_PA	fp16_51	16	51.802
FB_0433631_L1_PA	fp16_51	16	51.802
FB_0188904_L13_PA	fp16_51	16	51.802
FB_0788909_L9_PA	fp16_51	16	51.802
FB_0364483_L16_PA	fp16_51	16	51.802
FB_0284555_L15_PA	fp16_51	16	51.802
FB_0433626_L1_PA	fp16_51	16	51.802
FB_0926221_L16_25_1	fp16_51	16	51.802
FB_0360096_L16_PA	fp16_51	16	51.802
FB_0359650_L16_PA	fp16_51	16	51.802
FB_0434405_L1_PA	fp16_51	16	51.802
FB_0293376_L15_PA	fp16_51	16	51.802
FB_0364473_L16_PA	fp16_51	16	51.802
FB_0925847_L16_25_1	fp16_51	16	51.802
FB_0788925_L9_PA	fp16_51	16	51.802
FB_0925851_L16_25_1	fp16_51	16	51.802
FB_0925846_L16_25_1	fp16_51	16	51.802
FB_0322940_L15_PA	fp16_51	16	51.802
FB_0359648_L16_PA	fp16_51	16	51.802
FB_0434432_L1_PA	fp16_51	16	51.802
FB_0360031_L16_PA	fp16_51	16	51.802
FB_0284577_L15_PA	fp16_51	16	51.802
FB_0361410_L16_PA	fp16_51	16	51.802
FB_0293351_L15_PA	fp16_51	16	51.802
FB_0364470_L16_PA	fp16_51	16	51.802
FB_0360039_L16_PA	fp16_51	16	51.802
FB_0926224_L16_25_1	fp16_51	16	51.802
FB_0188893_L13_PA	fp16_51	16	51.802
FB_0188890_L13_PA	fp16_51	16	51.802

FB_0433627_L1_PA	fp16_51	16	51.802
FB_0788926_L9_PA	fp16_51	16	51.802
FB_0188885_L13_PA	fp16_51	16	51.802
FB_0365149_L16_PA	fp16_51	16	51.802
FB_0434444_L1_PA	fp16_51	16	51.802
FB_0284557_L15_PA	fp16_51	16	51.802
FB_0434464_L1_PA	fp16_51	16	51.802
GD_01237_L16_PA	fp16_51	16	51.802
FB_0360045_L16_PA	fp16_51	16	51.802
FB_0433622_L1_PA	fp16_51	16	51.802
FB_0998236_L5_44_1	fp16_51	16	51.802
FB_0359645_L16_PA	fp16_51	16	51.802
FB_0925849_L16_25_1	fp16_51	16	51.802
FB_0359651_L16_PA	fp16_51	16	51.802
FB_0360030_L16_PA	fp16_51	16	51.802
FB_0926202_L16_25_1	fp16_51	16	51.802
FB_0434409_L1_PA	fp16_51	16	51.802
FB_0998256_L5_44_1	fp16_51	16	51.802
FB_0284543_L15_PA	fp16_51	16	51.802
FB_0977558_L4_14_1	fp16_51	16	51.802
FB_0433654_L1_PA	fp16_51	16	51.802
FB_0926216_L16_25_1	fp16_51	16	51.802
RB_20224906_L16_PA	fp16_51	16	51.802
FB_0434424_L1_PA	fp16_51	16	51.802
FB_0433639_L1_PA	fp16_51	16	51.802
FB_0925850_L16_25_1	fp16_51	16	51.802
FB_0192231_L13_PA	fp16_51	16	51.802
FB_0188886_L13_PA	fp16_51	16	51.802
FB_0788914_L9_PA	fp16_51	16	51.802
FB_0788908_L9_PA	fp16_51	16	51.802
FB_0360037_L16_PA	fp16_51	16	51.802
FB_0293370_L15_PA	fp16_51	16	51.802
FB_0433649_L1_PA	fp16_51	16	51.802
FB_0977581_L4_14_1	fp16_51	16	51.802
GD_02207_L16_25_1	fp16_51	16	51.802
FB_0359684_L16_PA	fp16_51	16	51.802
FB_0365160_L16_PA	fp16_52	16	52.443
FB_0434449_L1_PA	fp16_53	16	53.254
GD_00879_L16_PA	fp16_53	16	53.254
FB_0352058_L16_PA	fp16_53	16	53.254
FB_0439991_L1_PA	fp16_53	16	53.254
FB_0352061_L16_PA	fp16_53	16	53.254
FB_0439970_L1_PA	fp16_53	16	53.254
FB_0439971_L1_PA	fp16_53	16	53.254
FB_0439985_L1_PA	fp16_53	16	53.254
FB_0439351_L1_PA	fp16_53	16	53.254

FB_0439972_L1_PA	fp16_53	16	53.254
FB_1082002_L1_82_2	fp16_53	16	53.254
FB_0439327_L1_PA	fp16_53	16	53.254
FB_0365226_L16_PA	fp16_53	16	53.254
FB_0365213_L16_PA	fp16_53	16	53.254
FB_0188884_L13_PA	fp16_54	16	54.555
FB_0351381_L16_PA	fp16_54	16	54.555
FB_0366025_L16_PA	fp16_54	16	54.555
FB_0366029_L16_PA	fp16_54	16	54.555
FB_0365542_L16_PA	fp16_54	16	54.555
FB_0392587_L17_PA	fp16_54	16	54.555
GD_00626_L16_28_1	fp16_54	16	54.555
FB_0366006_L16_PA	fp16_54	16	54.555
FB_1116422_L6_62_3	fp16_54	16	54.555
FB_0365536_L16_PA	fp16_54	16	54.555
FB_0365214_L16_PA	fp16_54	16	54.555
FB_0365538_L16_PA	fp16_54	16	54.555
FB_0352048_L16_PA	fp16_54	16	54.555
FB_0883406_L13_49_1	fp16_54	16	54.555
RB_9996257_L16_PA	fp16_54	16	54.555
FB_0393156_L17_PA	fp16_55	16	55.637
RB_9973499_L16_PA	fp16_55	16	55.637
RB_10009408_L16_PA	fp16_55	16	55.637
RB_9988815_L16_PA	fp16_55	16	55.637
FB_0934532_L17_18_1	fp16_55	16	55.637
FB_0351380_L16_PA	fp16_55	16	55.637
FB_0352059_L16_PA	fp16_55	16	55.637
FB_0392580_L17_PA	fp16_56	16	56.052
FB_0365540_L16_PA	fp16_57	16	57.254
FB_0351386_L16_PA	fp16_58	16	58.389
FB_1116822_L6_67_3	fp16_58	16	58.389
FB_0177170_L13_PA	fp16_58	16	58.389
FB_1116812_L6_67_3	fp16_58	16	58.389
FB_0927441_L16_28_1	fp16_58	16	58.389
FB_0934542_L17_18_1	fp16_59	16	59.283
FB_0352053_L16_PA	fp16_59	16	59.283
FB_0883411_L13_49_1	fp16_59	16	59.283
FB_0883397_L13_49_1	fp16_59	16	59.283
FB_0934529_L17_18_1	fp16_59	16	59.283
FB_0363172_L16_PA	fp16_59	16	59.283
FB_0034558_L10_PA	fp16_59	16	59.283
FB_0366583_L16_PA	fp16_59	16	59.283
FB_0366056_L16_PA	fp16_60	16	60.317
FB_0366017_L16_PA	fp16_60	16	60.317
FB_1116817_L6_67_3	fp16_61	16	61.586
FB_0177167_L13_PA	fp16_61	16	61.586

FB_0350111_L16_PA	fp16_61	16	61.586
FB_0177171_L13_PA	fp16_62	16	62.767
FB_0366584_L16_PA	fp16_62	16	62.767
FB_1116824_L6_67_3	fp16_62	16	62.767
FB_0366582_L16_PA	fp16_62	16	62.767
FB_0952160_L2_21_1	fp16_62	16	62.767
FB_0350560_L16_PA	fp16_64	16	64.676
FB_0616463_L5_PA	fp16_65	16	65.356
FB_1107495_L10_76_3	fp16_65	16	65.356
FB_1025414_L8_35_1	fp16_65	16	65.356
FB_0614993_L5_PA	fp16_65	16	65.356
FB_1025413_L8_35_1	fp16_65	16	65.356
FB_1107500_L10_76_3	fp16_65	16	65.356
FB_0616473_L5_PA	fp16_65	16	65.356
FB_0363139_L16_PA	fp16_65	16	65.356
FB_0509160_L3_PA	fp16_65	16	65.356
FB_0931282_L17_14_1	fp16_65	16	65.356
FB_0931283_L17_14_1	fp16_66	16	66.187
FB_0616489_L5_PA	fp16_66	16	66.187
FB_0475998_L2_PA	fp16_66	16	66.187
FB_0363165_L16_PA	fp16_67	16	67.594
FB_0304652_L15_PA	fp16_67	16	67.594
FB_0363149_L16_PA	fp16_67	16	67.594
FB_0448914_L2_PA	fp16_67	16	67.594
FB_1116388_L5_99_3	fp16_67	16	67.594
FB_0304673_L15_PA	fp16_67	16	67.594
FB_0350132_L16_PA	fp16_67	16	67.594
FB_0350124_L16_PA	fp16_67	16	67.594
FB_0368056_L16_PA	fp16_67	16	67.594
FB_0509149_L3_PA	fp16_67	16	67.594
FB_0034564_L10_PA	fp16_67	16	67.594
FB_0616485_L5_PA	fp16_67	16	67.594
FB_0368065_L16_PA	fp16_67	16	67.594
FB_0034555_L10_PA	fp16_67	16	67.594
FB_0304650_L15_PA	fp16_67	16	67.594
FB_0931276_L17_14_1	fp16_67	16	67.594
RB_23405001_L16_PA	fp16_67	16	67.594
FB_0350133_L16_PA	fp16_67	16	67.594
FB_0931279_L17_14_1	fp16_67	16	67.594
RB_18500461_L16_37_2	fp16_67	16	67.594
FB_0368062_L16_PA	fp16_67	16	67.594
FB_0034751_L10_PA	fp16_67	16	67.594
FB_0350118_L16_PA	fp16_67	16	67.594
FB_0509152_L3_PA	fp16_67	16	67.594
FB_0616468_L5_PA	fp16_67	16	67.594
RB_9806895_L16_PA	fp16_67	16	67.594

RB_18500686_L16_37_2	fp16_67	16	67.594
FB_0368061_L16_PA	fp16_67	16	67.594
FB_1011231_L6_44_1	fp16_67	16	67.594
FB_0363422_L16_PA	fp16_67	16	67.594
RB_23308740_L16_PA	fp16_67	16	67.594
FB_0475987_L2_PA	fp16_67	16	67.594
FB_0304660_L15_PA	fp16_67	16	67.594
FB_0034760_L10_PA	fp16_67	16	67.594
FB_0931278_L17_14_1	fp16_67	16	67.594
FB_0363142_L16_PA	fp16_67	16	67.594
FB_0931292_L17_14_1	fp16_67	16	67.594
FB_0350114_L16_PA	fp16_67	16	67.594
FB_1116381_L5_99_3	fp16_68	16	68.84
FB_0995379_L5_42_1	fp16_68	16	68.84
FB_0928013_L17_13_1	fp17_00	17	0.438
FB_0368193_L17_PA	fp17_00	17	0.438
FB_0368278_L17_PA	fp17_00	17	0.438
RB_71128_L17_PA	fp17_00	17	0.438
FB_0368710_L17_PA	fp17_00	17	0.438
FB_0368723_L17_PA	fp17_00	17	0.438
RB_343464_L17_PA	fp17_00	17	0.438
RB_300897_L17_PA	fp17_00	17	0.438
RB_406763_L17_PA	fp17_00	17	0.438
FB_0928002_L17_13_1	fp17_00	17	0.438
FB_0369306_L17_PA	fp17_00	17	0.438
FB_0928037_L17_13_1	fp17_00	17	0.438
FB_0369304_L17_PA	fp17_00	17	0.438
FB_0928015_L17_13_1	fp17_00	17	0.438
FB_0928007_L17_13_1	fp17_00	17	0.438
GD_00091_L17_PA	fp17_00	17	0.438
FB_0928341_L17_13_1	fp17_00	17	0.438
GD_01974_L17_PA	fp17_00	17	0.438
FB_0369315_L17_PA	fp17_00	17	0.438
FB_0928346_L17_13_1	fp17_00	17	0.438
RB_413069_L17_PA	fp17_00	17	0.438
FB_0369312_L17_PA	fp17_00	17	0.438
RB_815140_L17_PA	fp17_00	17	0.438
FB_0368185_L17_PA	fp17_00	17	0.438
FB_0368188_L17_PA	fp17_00	17	0.438
RB_810662_L17_PA	fp17_00	17	0.438
RB_772407_L17_13_1	fp17_00	17	0.438
FB_0368284_L17_PA	fp17_00	17	0.438
RB_805127_L17_PA	fp17_00	17	0.438
FB_0368192_L17_PA	fp17_01	17	1.279
FB_0368190_L17_PA	fp17_01	17	1.279
FB_0928329_L17_13_1	fp17_01	17	1.279

FB_0928328_L17_13_1	fp17_01	17	1.279
RB_819070_L17_PA	fp17_02	17	2.503
RB_1640875_L17_PA	fp17_02	17	2.503
FB_0370191_L17_PA	fp17_02	17	2.503
FB_0370195_L17_PA	fp17_03	17	3.279
RB_2045677_L17_PA	fp17_04	17	4.725
RB_1334232_L17_PA	fp17_04	17	4.725
FB_0370202_L17_PA	fp17_04	17	4.725
FB_0370197_L17_PA	fp17_04	17	4.725
FB_0370217_L17_PA	fp17_04	17	4.725
FB_0370194_L17_PA	fp17_04	17	4.725
GD_00864_L17_PA	fp17_04	17	4.725
RB_1149469_L17_13_1	fp17_04	17	4.725
FB_1104480_L9_39_2	fp17_04	17	4.725
FB_0370461_L17_PA	fp17_04	17	4.725
FB_1104503_L9_39_2	fp17_04	17	4.725
FB_1104490_L9_39_2	fp17_04	17	4.725
FB_1104483_L9_39_2	fp17_04	17	4.725
GD_01205_L17_PA	fp17_04	17	4.725
RB_1318292_L17_PA	fp17_04	17	4.725
FB_0370204_L17_PA	fp17_04	17	4.725
FB_1077016_L17_24_2	fp17_04	17	4.725
FB_1077017_L17_24_2	fp17_04	17	4.725
FB_1104475_L9_39_2	fp17_04	17	4.725
FB_0370455_L17_PA	fp17_04	17	4.725
FB_0370454_L17_PA	fp17_05	17	5.601
FB_0371534_L17_PA	fp17_05	17	5.601
FB_0370778_L17_PA	fp17_05	17	5.601
FB_0370782_L17_PA	fp17_05	17	5.601
RB_1638129_L17_PA	fp17_05	17	5.601
RB_1604094_L17_PA	fp17_05	17	5.601
FB_1077020_L17_24_2	fp17_05	17	5.601
RB_1601079_L17_PA	fp17_05	17	5.601
FB_0370766_L17_PA	fp17_05	17	5.601
FB_0370764_L17_PA	fp17_05	17	5.601
FB_0370772_L17_PA	fp17_05	17	5.601
RB_2053599_L17_PA	fp17_05	17	5.601
FB_0371554_L17_PA	fp17_05	17	5.601
RB_1621844_L17_PA	fp17_05	17	5.601
FB_0371541_L17_PA	fp17_05	17	5.601
RB_2034824_L17_PA	fp17_05	17	5.601
RB_2073815_L17_PA	fp17_05	17	5.601
FB_0371538_L17_PA	fp17_06	17	6.562
FB_0370801_L17_PA	fp17_06	17	6.562
FB_0928593_L17_13_1	fp17_06	17	6.562
FB_0371536_L17_PA	fp17_06	17	6.562

FB_0371528_L17_PA	fp17_06	17	6.562
FB_0373001_L17_PA	fp17_06	17	6.562
FB_0372386_L17_PA	fp17_06	17	6.562
FB_0372378_L17_PA	fp17_06	17	6.562
RB_2688072_L17_PA	fp17_07	17	7.428
FB_0372389_L17_PA	fp17_07	17	7.428
RB_2378561_L17_PA	fp17_07	17	7.428
FB_0372399_L17_PA	fp17_07	17	7.428
RB_3543730_L17_PA	fp17_08	17	8.018
RB_3503210_L17_PA	fp17_08	17	8.018
RB_2741553_L17_PA	fp17_08	17	8.018
RB_3496763_L17_PA	fp17_08	17	8.018
GD_00508_L17_PA	fp17_09	17	9.562
FB_0375697_L17_PA	fp17_09	17	9.562
FB_0374760_L17_PA	fp17_10	17	10.962
RB_3135072_L17_PA	fp17_10	17	10.962
GD_01584_L17_PA	fp17_10	17	10.962
FB_0374774_L17_PA	fp17_10	17	10.962
FB_0374747_L17_PA	fp17_11	17	11.332
RB_3492127_L17_PA	fp17_11	17	11.332
FB_0375668_L17_PA	fp17_11	17	11.332
RB_4157765_L17_PA	fp17_11	17	11.332
RB_3878697_L17_PA	fp17_12	17	12.396
FB_0375945_L17_PA	fp17_12	17	12.396
RB_4101051_L17_PA	fp17_12	17	12.396
FB_1104894_L9_39_2	fp17_12	17	12.396
FB_0375671_L17_PA	fp17_12	17	12.396
RB_4152973_L17_PA	fp17_12	17	12.396
FB_1104895_L9_39_2	fp17_12	17	12.396
FB_0375955_L17_PA	fp17_12	17	12.396
FB_1104890_L9_39_2	fp17_12	17	12.396
FB_0375958_L17_PA	fp17_13	17	13.676
FB_0777709_L9_PA	fp17_13	17	13.676
FB_0377186_L17_PA	fp17_13	17	13.676
FB_0377153_L17_PA	fp17_13	17	13.676
FB_1098256_L6_60_2	fp17_13	17	13.676
FB_0377159_L17_PA	fp17_13	17	13.676
FB_1104904_L9_39_2	fp17_14	17	14.436
FB_1104901_L9_39_2	fp17_14	17	14.436
FB_0777728_L9_PA	fp17_14	17	14.436
FB_0777717_L9_PA	fp17_14	17	14.436
FB_0378102_L17_PA	fp17_14	17	14.436
FB_0378097_L17_PA	fp17_14	17	14.436
FB_0377179_L17_PA	fp17_15	17	15.69
FB_0377180_L17_PA	fp17_15	17	15.69
GD_00606_L17_PA	fp17_15	17	15.69

FB_0378266_L17_PA	fp17_15	17	15.69
FB_0378273_L17_PA	fp17_15	17	15.69
FB_0378277_L17_PA	fp17_15	17	15.69
FB_0377677_L17_PA	fp17_15	17	15.69
FB_0930267_L17_14_1	fp17_15	17	15.69
FB_0378099_L17_PA	fp17_15	17	15.69
FB_0930265_L17_14_1	fp17_15	17	15.69
FB_0378287_L17_PA	fp17_15	17	15.69
FB_0930270_L17_14_1	fp17_15	17	15.69
FB_0378291_L17_PA	fp17_16	17	16.652
RB_5995947_L17_PA	fp17_16	17	16.652
FB_0378276_L17_PA	fp17_16	17	16.652
FB_0378279_L17_PA	fp17_16	17	16.652
RB_5689554_L17_PA	fp17_16	17	16.652
FB_0930268_L17_14_1	fp17_16	17	16.652
FB_0930543_L17_14_1	fp17_16	17	16.652
FB_0378296_L17_PA	fp17_16	17	16.652
RB_5879958_L17_PA	fp17_16	17	16.652
RB_5757117_L17_PA	fp17_17	17	17.621
RB_5943747_L17_PA	fp17_17	17	17.621
RB_5701622_L17_PA	fp17_17	17	17.621
FB_0930473_L17_14_1	fp17_17	17	17.621
FB_0378882_L17_PA	fp17_17	17	17.621
FB_0378884_L17_PA	fp17_17	17	17.621
FB_0930470_L17_14_1	fp17_17	17	17.621
FB_0378885_L17_PA	fp17_17	17	17.621
FB_0378875_L17_PA	fp17_17	17	17.621
FB_0378883_L17_PA	fp17_17	17	17.621
FB_0930485_L17_14_1	fp17_17	17	17.621
FB_0378900_L17_PA	fp17_17	17	17.621
FB_0378904_L17_PA	fp17_17	17	17.621
FB_0378881_L17_PA	fp17_17	17	17.621
FB_0378899_L17_PA	fp17_17	17	17.621
FB_0930539_L17_14_1	fp17_17	17	17.621
RB_6590432_L17_PA	fp17_17	17	17.621
FB_0378879_L17_PA	fp17_17	17	17.621
FB_0379577_L17_PA	fp17_18	17	18.428
RB_5726495_L17_PA	fp17_18	17	18.428
FB_0379786_L17_PA	fp17_18	17	18.428
FB_0379818_L17_PA	fp17_18	17	18.428
FB_0379793_L17_PA	fp17_18	17	18.428
FB_0379788_L17_PA	fp17_18	17	18.428
FB_0379809_L17_PA	fp17_18	17	18.428
FB_0379797_L17_PA	fp17_18	17	18.428
FB_0379554_L17_PA	fp17_18	17	18.428
RB_5988456_L17_PA	fp17_18	17	18.428

FB_0930559_L17_14_1	fp17_18	17	18.428
FB_0930536_L17_14_1	fp17_18	17	18.428
FB_0930537_L17_14_1	fp17_18	17	18.428
FB_0379807_L17_PA	fp17_18	17	18.428
RB_6617432_L17_PA	fp17_18	17	18.428
RB_6635552_L17_PA	fp17_18	17	18.428
GD_01542_L17_PA	fp17_18	17	18.428
RB_6595951_L17_PA	fp17_19	17	19.986
FB_0380669_L17_PA	fp17_20	17	20.791
FB_0380673_L17_PA	fp17_20	17	20.791
FB_0381115_L17_PA	fp17_20	17	20.791
RB_7363032_L17_PA	fp17_20	17	20.791
FB_0380694_L17_PA	fp17_21	17	21.767
FB_0381678_L17_PA	fp17_21	17	21.767
RB_7407078_L17_PA	fp17_21	17	21.767
FB_0380679_L17_PA	fp17_21	17	21.767
FB_0380683_L17_PA	fp17_21	17	21.767
FB_0381686_L17_PA	fp17_21	17	21.767
RB_7402862_L17_PA	fp17_21	17	21.767
FB_0380687_L17_PA	fp17_21	17	21.767
FB_0381109_L17_PA	fp17_21	17	21.767
FB_0995529_L5_42_1	fp17_21	17	21.767
FB_0380680_L17_PA	fp17_21	17	21.767
FB_0381659_L17_PA	fp17_21	17	21.767
GD_02512_L17_PA	fp17_21	17	21.767
FB_0381658_L17_PA	fp17_21	17	21.767
FB_0382650_L17_PA	fp17_23	17	23.395
RB_8261983_L17_PA	fp17_25	17	25.934
RB_8655852_L17_PA	fp17_25	17	25.934
RB_8820322_L17_PA	fp17_26	17	26.602
RB_8806260_L17_PA	fp17_26	17	26.602
RB_8827345_L17_PA	fp17_26	17	26.602
FB_0491261_L2_PA	fp17_26	17	26.602
FB_0382645_L17_PA	fp17_26	17	26.602
RB_8234228_L17_PA	fp17_26	17	26.602
FB_0382662_L17_PA	fp17_26	17	26.602
FB_0382659_L17_PA	fp17_26	17	26.602
FB_0931806_L17_15_1	fp17_26	17	26.602
FB_0382652_L17_PA	fp17_26	17	26.602
FB_0384529_L17_PA	fp17_26	17	26.602
GD_00039_L17_15_1	fp17_26	17	26.602
FB_0382985_L17_PA	fp17_27	17	27.547
FB_0382647_L17_PA	fp17_27	17	27.547
FB_0382654_L17_PA	fp17_27	17	27.547
FB_0384543_L17_PA	fp17_27	17	27.547
FB_0384526_L17_PA	fp17_27	17	27.547

RB_9390133_L17_PA	fp17_28	17	28.085
FB_0384141_L17_PA	fp17_28	17	28.085
FB_0384153_L17_PA	fp17_28	17	28.085
FB_0384527_L17_PA	fp17_28	17	28.085
FB_0384539_L17_PA	fp17_28	17	28.085
FB_0384537_L17_PA	fp17_28	17	28.085
FB_0384158_L17_PA	fp17_28	17	28.085
FB_0384528_L17_PA	fp17_28	17	28.085
FB_0384145_L17_PA	fp17_30	17	30.9
FB_0386767_L17_PA	fp17_31	17	31.24
GD_00918_L17_PA	fp17_31	17	31.24
RB_11958448_L17_PA	fp17_31	17	31.24
FB_0385990_L17_PA	fp17_31	17	31.24
FB_0389300_L17_PA	fp17_31	17	31.24
GD_01044_L17_PA	fp17_31	17	31.24
RB_10872135_L17_PA	fp17_31	17	31.24
FB_0388809_L17_PA	fp17_31	17	31.24
RB_10388012_L17_PA	fp17_31	17	31.24
FB_0387348_L17_PA	fp17_31	17	31.24
FB_0385963_L17_PA	fp17_31	17	31.24
FB_0387349_L17_PA	fp17_31	17	31.24
FB_0386768_L17_PA	fp17_32	17	32.499
FB_0386775_L17_PA	fp17_32	17	32.499
RB_10385387_L17_PA	fp17_32	17	32.499
FB_0387753_L17_PA	fp17_33	17	33.458
FB_0387766_L17_PA	fp17_33	17	33.458
FB_0389915_L17_PA	fp17_33	17	33.458
RB_12093889_L17_PA	fp17_33	17	33.458
FB_0386769_L17_PA	fp17_33	17	33.458
RB_10935478_L17_PA	fp17_33	17	33.458
RB_12031565_L17_PA	fp17_33	17	33.458
RB_12762801_L17_PA	fp17_33	17	33.458
FB_0387775_L17_PA	fp17_33	17	33.458
FB_0387788_L17_PA	fp17_33	17	33.458
FB_0387793_L17_PA	fp17_33	17	33.458
FB_0387778_L17_PA	fp17_33	17	33.458
FB_0387794_L17_PA	fp17_33	17	33.458
FB_0390504_L17_PA	fp17_33	17	33.458
RB_10376441_L17_PA	fp17_34	17	34.448
FB_0385972_L17_PA	fp17_34	17	34.448
FB_0388787_L17_PA	fp17_34	17	34.448
FB_0388797_L17_PA	fp17_34	17	34.448
FB_0389304_L17_PA	fp17_34	17	34.448
FB_0388791_L17_PA	fp17_34	17	34.448
GD_01800_L17_PA	fp17_34	17	34.448
FB_0387786_L17_PA	fp17_34	17	34.448

FB_0389967_L17_PA	fp17_34	17	34.448
FB_0388776_L17_PA	fp17_34	17	34.448
FB_0389309_L17_PA	fp17_34	17	34.448
FB_0389305_L17_PA	fp17_34	17	34.448
FB_0389298_L17_PA	fp17_34	17	34.448
GD_00682_L17_PA	fp17_34	17	34.448
FB_0385970_L17_PA	fp17_34	17	34.448
FB_0388815_L17_PA	fp17_34	17	34.448
FB_0933297_L17_16_1	fp17_34	17	34.448
FB_0933277_L17_16_1	fp17_34	17	34.448
FB_0389904_L17_PA	fp17_34	17	34.448
RB_12618986_L17_PA	fp17_34	17	34.448
RB_11943171_L17_PA	fp17_34	17	34.448
RB_12623183_L17_PA	fp17_34	17	34.448
FB_0933284_L17_16_1	fp17_34	17	34.448
FB_0389993_L17_PA	fp17_34	17	34.448
GD_02172_L17_PA	fp17_34	17	34.448
FB_0389992_L17_PA	fp17_34	17	34.448
FB_0390000_L17_PA	fp17_34	17	34.448
RB_12549633_L17_PA	fp17_34	17	34.448
FB_0389909_L17_PA	fp17_34	17	34.448
FB_0389888_L17_PA	fp17_34	17	34.448
FB_0389889_L17_PA	fp17_34	17	34.448
FB_0389991_L17_PA	fp17_34	17	34.448
FB_0933278_L17_16_1	fp17_34	17	34.448
RB_12632221_L17_PA	fp17_35	17	35.723
FB_0390481_L17_PA	fp17_36	17	36.627
FB_0390489_L17_PA	fp17_36	17	36.627
FB_0390502_L17_PA	fp17_36	17	36.627
FB_0390491_L17_PA	fp17_36	17	36.627
FB_0390484_L17_PA	fp17_36	17	36.627
FB_0390505_L17_PA	fp17_36	17	36.627
FB_0391310_L17_PA	fp17_37	17	37.846
FB_0390508_L17_PA	fp17_37	17	37.846
FB_0390479_L17_PA	fp17_37	17	37.846
FB_0391323_L17_PA	fp17_37	17	37.846
FB_0391309_L17_PA	fp17_37	17	37.846
FB_0392204_L17_PA	fp17_39	17	39.488
FB_0392208_L17_PA	fp17_39	17	39.488
FB_0392193_L17_PA	fp17_39	17	39.488
FB_0392187_L17_PA	fp17_39	17	39.488
FB_0392182_L17_PA	fp17_39	17	39.488
GD_02572_L17_PA	fp17_39	17	39.488
RB_14005103_L17_PA	fp17_39	17	39.488
RB_14008824_L17_PA	fp17_39	17	39.488
FB_1067288_L14_55_2	fp17_39	17	39.488

FB_1112193_L16_40_3	fp17_39	17	39.488
RB_13980746_L17_PA	fp17_39	17	39.488
FB_1112184_L16_40_3	fp17_39	17	39.488
FB_1067276_L14_55_2	fp17_39	17	39.488
FB_0392422_L17_PA	fp17_39	17	39.488
FB_0392425_L17_PA	fp17_39	17	39.488
FB_1067277_L14_55_2	fp17_39	17	39.488
FB_0392441_L17_PA	fp17_39	17	39.488
FB_1112185_L16_40_3	fp17_39	17	39.488
FB_0392184_L17_PA	fp17_40	17	40.845
FB_0824240_L9_PA	fp17_40	17	40.845
FB_0392505_L17_PA	fp17_40	17	40.845
GD_00358_L17_PA	fp17_40	17	40.845
FB_0392512_L17_PA	fp17_40	17	40.845
FB_0824237_L9_PA	fp17_40	17	40.845
FB_0392421_L17_PA	fp17_41	17	41.277
FB_0392502_L17_PA	fp17_41	17	41.277
FB_0392503_L17_PA	fp17_43	17	43.323
FB_1078711_L17_28_2	fp17_43	17	43.323
FB_0858514_L12_35_1	fp17_44	17	44.706
FB_0245451_L14_PA	fp17_44	17	44.706
FB_0505337_L3_PA	fp17_44	17	44.706
FB_0200591_L13_PA	fp17_44	17	44.706
FB_0702449_L7_PA	fp17_44	17	44.706
FB_0313253_L15_PA	fp17_44	17	44.706
FB_0200613_L13_PA	fp17_44	17	44.706
FB_0200586_L13_PA	fp17_44	17	44.706
RB_15762340_L17_PA	fp17_44	17	44.706
FB_0394747_L17_PA	fp17_44	17	44.706
FB_0392504_L17_PA	fp17_44	17	44.706
RB_16478194_L17_PA	fp17_44	17	44.706
FB_0392499_L17_PA	fp17_44	17	44.706
FB_0824238_L9_PA	fp17_44	17	44.706
FB_1078740_L17_28_2	fp17_44	17	44.706
FB_1078745_L17_28_2	fp17_44	17	44.706
FB_1078715_L17_28_2	fp17_44	17	44.706
GD_01438_L17_PA	fp17_44	17	44.706
FB_0395205_L17_PA	fp17_44	17	44.706
FB_0395191_L17_PA	fp17_44	17	44.706
FB_0395196_L17_PA	fp17_44	17	44.706
FB_0394752_L17_PA	fp17_44	17	44.706
FB_0802273_L9_PA	fp17_44	17	44.706
FB_0858513_L12_35_1	fp17_45	17	45.819
FB_0038875_L10_PA	fp17_45	17	45.819
FB_0038879_L10_PA	fp17_45	17	45.819
RB_15760100_L17_PA	fp17_46	17	46.216

FB_0119063_L12_PA	fp17_46	17	46.216
FB_0505329_L3_PA	fp17_46	17	46.216
FB_0312705_L15_PA	fp17_46	17	46.216
FB_0313574_L15_PA	fp17_46	17	46.216
FB_0505306_L3_PA	fp17_46	17	46.216
FB_0394302_L17_PA	fp17_46	17	46.216
FB_0394315_L17_PA	fp17_46	17	46.216
RB_15764597_L17_PA	fp17_46	17	46.216
FB_0505541_L3_PA	fp17_46	17	46.216
FB_0505555_L3_PA	fp17_46	17	46.216
RB_15782830_L17_PA	fp17_46	17	46.216
FB_0733489_L8_PA	fp17_47	17	47.774
FB_0395777_L17_PA	fp17_47	17	47.774
FB_0244417_L14_PA	fp17_47	17	47.774
FB_0244418_L14_PA	fp17_47	17	47.774
GD_01964_L17_PA	fp17_47	17	47.774
FB_0802270_L9_PA	fp17_47	17	47.774
FB_0244421_L14_PA	fp17_47	17	47.774
FB_0244452_L14_PA	fp17_47	17	47.774
FB_0244424_L14_PA	fp17_47	17	47.774
FB_1047154_L9_27_1	fp17_47	17	47.774
FB_0802269_L9_PA	fp17_47	17	47.774
FB_0395782_L17_PA	fp17_47	17	47.774
FB_0395784_L17_PA	fp17_47	17	47.774
FB_0395766_L17_PA	fp17_47	17	47.774
RB_17325549_L17_PA	fp17_48	17	48.231
FB_0396411_L17_PA	fp17_48	17	48.231
FB_0396402_L17_PA	fp17_48	17	48.231
FB_0396409_L17_PA	fp17_48	17	48.231
FB_0396868_L17_PA	fp17_49	17	49.316
FB_0396867_L17_PA	fp17_49	17	49.316
FB_0396847_L17_PA	fp17_49	17	49.316
FB_0397625_L17_PA	fp17_49	17	49.316
FB_0397642_L17_PA	fp17_49	17	49.316
FB_0397897_L17_PA	fp17_50	17	50.721
FB_0732964_L8_PA	fp17_50	17	50.721
FB_0732983_L8_PA	fp17_50	17	50.721
FB_0732952_L8_PA	fp17_50	17	50.721
FB_0732967_L8_PA	fp17_50	17	50.721
FB_0665284_L6_PA	fp17_51	17	51.678
FB_0665268_L6_PA	fp17_51	17	51.678
FB_0665015_L6_PA	fp17_51	17	51.678
FB_0438366_L1_PA	fp17_51	17	51.678
FB_0665257_L6_PA	fp17_51	17	51.678
FB_0665258_L6_PA	fp17_51	17	51.678
FB_1006298_L6_37_1	fp17_51	17	51.678

FB_0665265_L6_PA	fp17_51	17	51.678
FB_0665012_L6_PA	fp17_51	17	51.678
FB_0665010_L6_PA	fp17_51	17	51.678
FB_0665035_L6_PA	fp17_51	17	51.678
FB_0624828_L5_PA	fp17_52	17	52.065
FB_0102605_L11_PA	fp17_52	17	52.065
FB_0193921_L13_PA	fp17_53	17	53.271
FB_0624539_L5_PA	fp17_53	17	53.271
FB_0438354_L1_PA	fp17_53	17	53.271
FB_0438852_L1_PA	fp17_53	17	53.271
FB_0193910_L13_PA	fp17_53	17	53.271
FB_0102608_L11_PA	fp17_53	17	53.271
FB_0102619_L11_PA	fp17_53	17	53.271
FB_0102615_L11_PA	fp17_53	17	53.271
FB_0624815_L5_PA	fp17_53	17	53.271
FB_0193904_L13_PA	fp17_53	17	53.271
FB_0624819_L5_PA	fp17_53	17	53.271
FB_0665255_L6_PA	fp17_53	17	53.271
FB_0193919_L13_PA	fp17_53	17	53.271
FB_0193923_L13_PA	fp17_53	17	53.271
FB_0438849_L1_PA	fp17_53	17	53.271
FB_0665036_L6_PA	fp17_53	17	53.271
FB_0624549_L5_PA	fp17_53	17	53.271
FB_0102600_L11_PA	fp17_53	17	53.271
FB_0624544_L5_PA	fp17_53	17	53.271
FB_0935827_L17_21_1	fp17_53	17	53.271
FB_0935831_L17_21_1	fp17_53	17	53.271
FB_0624821_L5_PA	fp17_53	17	53.271
FB_0193901_L13_PA	fp17_54	17	54.571
FB_0102606_L11_PA	fp17_54	17	54.571
FB_0438351_L1_PA	fp17_54	17	54.571
FB_0399627_L17_PA	fp17_54	17	54.571
FB_0399643_L17_PA	fp17_54	17	54.571
RB_20000116_L17_PA	fp17_54	17	54.571
FB_0399640_L17_PA	fp17_54	17	54.571
RB_19990934_L17_PA	fp17_54	17	54.571
FB_0665784_L6_PA	fp17_54	17	54.571
FB_0624844_L5_PA	fp17_54	17	54.571
FB_0624834_L5_PA	fp17_54	17	54.571
FB_0399644_L17_PA	fp17_54	17	54.571
FB_0935834_L17_21_1	fp17_54	17	54.571
GD_01298_L17_PA	fp17_54	17	54.571
FB_0438851_L1_PA	fp17_54	17	54.571
FB_0935742_L17_20_1	fp17_54	17	54.571
FB_1079006_L17_30_2	fp17_54	17	54.571
FB_0398756_L17_PA	fp17_54	17	54.571

FB_0398754_L17_PA	fp17_54	17	54.571
GD_00235_L17_PA	fp17_54	17	54.571
FB_0399637_L17_PA	fp17_54	17	54.571
RB_20010409_L17_PA	fp17_54	17	54.571
FB_0401483_L17_PA	fp17_55	17	55.37
FB_1114683_L3_102_3	fp17_55	17	55.37
RB_20028330_L17_PA	fp17_55	17	55.37
FB_0398757_L17_PA	fp17_55	17	55.37
FB_0399047_L17_PA	fp17_55	17	55.37
FB_0398054_L17_PA	fp17_55	17	55.37
FB_0935323_L17_20_1	fp17_55	17	55.37
FB_0398064_L17_PA	fp17_55	17	55.37
RB_20030687_L17_PA	fp17_55	17	55.37
FB_0398047_L17_PA	fp17_55	17	55.37
GD_00125_L17_PA	fp17_55	17	55.37
FB_0401458_L17_PA	fp17_55	17	55.37
FB_0935822_L17_21_1	fp17_55	17	55.37
FB_0398074_L17_PA	fp17_55	17	55.37
FB_1114681_L3_102_3	fp17_55	17	55.37
FB_0399619_L17_PA	fp17_55	17	55.37
FB_0398770_L17_PA	fp17_55	17	55.37
RB_21095267_L17_PA	fp17_55	17	55.37
FB_0398749_L17_PA	fp17_55	17	55.37
FB_0398761_L17_PA	fp17_55	17	55.37
FB_0936350_L17_21_1	fp17_55	17	55.37
FB_0402051_L17_PA	fp17_55	17	55.37
FB_0402081_L17_PA	fp17_55	17	55.37
FB_0401468_L17_PA	fp17_55	17	55.37
GD_01525_L17_PA	fp17_55	17	55.37
FB_0398065_L17_PA	fp17_55	17	55.37
FB_0402050_L17_PA	fp17_55	17	55.37
FB_0401470_L17_PA	fp17_55	17	55.37
FB_0402047_L17_PA	fp17_55	17	55.37
FB_0936353_L17_21_1	fp17_55	17	55.37
FB_0401456_L17_PA	fp17_55	17	55.37
FB_0936362_L17_21_1	fp17_55	17	55.37
FB_0401480_L17_PA	fp17_55	17	55.37
FB_0401464_L17_PA	fp17_55	17	55.37
RB_21201745_L17_PA	fp17_55	17	55.37
GD_00423_L17_PA	fp17_55	17	55.37
FB_0936375_L17_21_1	fp17_55	17	55.37
FB_0398062_L17_PA	fp17_55	17	55.37
FB_0936354_L17_21_1	fp17_55	17	55.37
FB_0840399_L11_29_1	fp17_55	17	55.37
FB_0402612_L17_PA	fp17_56	17	56.671
RB_20070315_L17_PA	fp17_56	17	56.671

RB_21775385_L17_PA	fp17_56	17	56.671
FB_0402631_L17_PA	fp17_56	17	56.671
FB_1112863_L17_40_3	fp17_56	17	56.671
RB_22813928_L17_PA	fp17_56	17	56.671
RB_22789618_L17_PA	fp17_56	17	56.671
FB_0937353_L17_22_1	fp17_57	17	57.38
RB_21188396_L17_PA	fp17_57	17	57.38
RB_21759969_L17_PA	fp17_57	17	57.38
FB_0402626_L17_PA	fp17_57	17	57.38
FB_0402623_L17_PA	fp17_57	17	57.38
FB_0402605_L17_PA	fp17_57	17	57.38
FB_0840400_L11_29_1	fp17_57	17	57.38
FB_0402610_L17_PA	fp17_58	17	58.612
FB_0187394_L13_PA	fp17_58	17	58.612
FB_0936361_L17_21_1	fp17_58	17	58.612
FB_0402591_L17_PA	fp17_58	17	58.612
RB_21762855_L17_PA	fp17_58	17	58.612
RB_21756554_L17_PA	fp17_58	17	58.612
FB_0840402_L11_29_1	fp17_58	17	58.612
FB_0402090_L17_PA	fp17_58	17	58.612
FB_0405232_L17_PA	fp17_58	17	58.612
FB_0402065_L17_PA	fp17_58	17	58.612
FB_0405230_L17_PA	fp17_59	17	59.369
FB_0402603_L17_PA	fp17_59	17	59.369
FB_1112855_L17_40_3	fp17_59	17	59.369
FB_1112873_L17_40_3	fp17_59	17	59.369
FB_1112861_L17_40_3	fp17_59	17	59.369
RB_24546944_L17_PA	fp17_59	17	59.369
FB_0405715_L17_PA	fp17_60	17	60.099
FB_0405728_L17_PA	fp17_60	17	60.099
RB_23178125_L17_PA	fp17_60	17	60.099
FB_0404463_L17_PA	fp17_60	17	60.099
FB_0406882_L17_PA	fp17_61	17	61.768
FB_0406117_L17_PA	fp17_62	17	62.222
FB_0405255_L17_PA	fp17_63	17	63.483
FB_0406116_L17_PA	fp17_63	17	63.483
GD_00618_L17_PA	fp17_63	17	63.483
RB_24829373_L17_PA	fp17_63	17	63.483
FB_0937354_L17_22_1	fp17_63	17	63.483
FB_0405249_L17_PA	fp17_63	17	63.483
RB_25284422_L17_PA	fp17_63	17	63.483
FB_0187371_L13_PA	fp17_63	17	63.483
RB_25283086_L17_PA	fp17_63	17	63.483
GD_00798_L17_PA	fp17_63	17	63.483
FB_0405233_L17_PA	fp17_63	17	63.483
GD_02678_L17_PA	fp17_63	17	63.483

FB_0405253_L17_PA	fp17_63	17	63.483
RB_25370424_L17_PA	fp17_64	17	64.88
FB_0406851_L17_PA	fp17_64	17	64.88
FB_0406852_L17_PA	fp17_64	17	64.88
FB_0406871_L17_PA	fp17_64	17	64.88
RB_25452621_L17_PA	fp17_64	17	64.88
RB_25607759_L17_PA	fp17_66	17	66.409
RB_25603780_L17_PA	fp17_66	17	66.409
FB_0408806_L17_PA	fp17_67	17	67.938
FB_0408412_L17_PA	fp17_67	17	67.938
FB_0408417_L17_PA	fp17_67	17	67.938
FB_0938197_L17_23_1	fp17_67	17	67.938
FB_0408394_L17_PA	fp17_67	17	67.938
FB_0408387_L17_PA	fp17_67	17	67.938
RB_26487167_L17_PA	fp17_67	17	67.938
FB_0408375_L17_PA	fp17_67	17	67.938
FB_0938201_L17_23_1	fp17_67	17	67.938
FB_0408805_L17_PA	fp17_68	17	68.581
FB_0408801_L17_PA	fp17_68	17	68.581
FB_1112979_L17_42_3	fp17_68	17	68.581
FB_0408802_L17_PA	fp17_68	17	68.581
FB_1112978_L17_42_3	fp17_68	17	68.581
FB_1112977_L17_42_3	fp17_68	17	68.581
RB_26729916_L17_23_1	fp17_68	17	68.581
FB_1112996_L17_42_3	fp17_68	17	68.581
RB_27113611_L17_PA	fp17_68	17	68.581

Supplementary Table S4

SSR	Chr	Seq. For.	Seq. Rev.
ch05g08	1	CCAAGACCAAGGCAACATTT	CCCTTCACCTCATTCTCACC
ch05e03	2	CGAATATTTTCACTCTGACTGGG	CAAGTTGTTGTACTGCTCCGAC
ch03g07	3	AATAAGCATTCAAAGCAATCCG	TTTTTCCAAATCGAGTTTCGTT
hi23g02	4	TTTTCCAGGATATACTACCCTTCC	GTTTCTTCGAGGTCAGGGTTTG
ch04e03	5	TTGAAGATGTTTGGCTGTGC	TGCATGTCTGTCTCCTCCAT
ch03d12	6	GCCCAGAAGCAATAAGTAAACC	ATTGCTCCATGCATAAAGGG
hi03a10	7	GGACCTGCTTCCCCTTATTC	CAGGGAACTTGTTTGATGG
ch01c06	8	TTCCCCATCATCGATCTCTC	AAACTGAAGCCATGAGGGC
ch01f03b	9	GAGAAGCAAATGCAAAACCC	CTCCCCGGCTCCTATTCTAC
ch02b03b	10	ATAAGGATACAAAAACCCTACACAG	GACATGTTTGGTTGAAAACTTG
ch04g07	11	CCCTAACCTCAATCCCCAAT	ATGAGGCAGGTGAAGAAGGA
ch01g12	12	CCCACCAATCAAAAATCACC	TGAAGTATGGTGGTGCGTTC
ch05h05	13	ACATGTCACTCCTACGCGG	GTGCAGTGATTAGCATTGCTGT
ch01g05	14	CATCAGTCTCTTGCACTGGAAA	GACAGAGTAAGCTAGGGCTAGGG
nz02b1	15	CCGTGATGACAAAGTGCATGA	ATGAGTTTGATGCCCTTGGA
ch04f10	16	GTAATGGAAATACAGTTTCACAA	TTAAATGCTTGGTGTGTTTTGC
gd96	17	CGGCGGAAAGCAATCACCT	GCCAGCCCTCTATGGTTCCAGA

Supplementary Table S5

GENE	*ANNOTATION*	*PRIMER FORWARD*	*PRIMER REVERSE*
Md_ACO	1-aminocyclopropane-1-carboxylate oxidase	AACGACTCATTCATCAGCCC	TTCAAATCTTGGTCCTTGG
Md_PG1	polygalacturonase	TCACCGGTGGGATAGCAACAT	GTCATCAGCACCATTCCCTTTAGC
Md_8283	houskeeping	CTCGTCGTCTTGTTCCCTGA	GCCTAAGGACAGGTGGTCTATG

Supplementary Table S6

CHR	*BP*	*P*	*SNP*	*Trait*
3	11.57717724	0.000914	RB_7171067_L3_PA	F Max
4	50.688	0.00094	FB_0588480_L4_PA	F Max
9	51.21080214	0.000695	FB_0202076_L13_PA	F Max
10	36.42745182	0.000938	FB_0026022_L10_PA	F Max
10	36.42745182	0.000938	FB_0831640_L10_40_1	F Max
10	36.42745182	0.000625	FB_0026051_L10_PA	F Max
14	14.72021931	0.000979	FB_0233257_L14_PA	F Max
14	15.814	0.000971	FB_0231494_L14_PA	F Max
14	16.0221504	0.000268	FB_0231503_L14_PA	F Max
14	16.416	0.000268	FB_0232191_L14_PA	F Max
14	17.018	0.000927	FB_0233245_L14_PA	F Max
15	54.91682743	0.000622	FB_0288397_L15_PA	F Max
1	49.556	0.000552	FB_0439066_L1_PA	Num Acoustic Peaks
1	49.556	0.00067	FB_0439059_L1_PA	Num Acoustic Peaks
2	43.56851494	0.000842	FB_0480288_L2_PA	Num Acoustic Peaks
2	44.22944471	0.000994	FB_0957128_L2_28_1	Num Acoustic Peaks
2	68.91351494	0.000545	FB_1086648_L2_52_2	Num Acoustic Peaks
2	70.11851494	0.000432	FB_0501809_L2_PA	Num Acoustic Peaks
2	71.93351494	0.000144	FB_0502410_L2_PA	Num Acoustic Peaks
2	73.8933603	2.17E-05	RB_39010781_L2_PA	Num Acoustic Peaks
3	32.327	0.000841	FB_0987796_L4_25_1	Num Acoustic Peaks
4	50.085	0.000361	FB_0587735_L4_PA	Num Acoustic Peaks
7	2.700179694	4.00E-04	FB_0687256_L7_PA	Num Acoustic Peaks
10	21.77438379	0.000634	FB_1052864_L10_57_2	Num Acoustic Peaks
10	26.3381206	0.000345	FB_0021346_L10_PA	Num Acoustic Peaks
10	32.74630461	0.00083	FB_0022307_L10_PA	Num Acoustic Peaks
10	36.42745182	0.000413	FB_0026022_L10_PA	Num Acoustic Peaks
10	36.42745182	0.000413	FB_0831640_L10_40_1	Num Acoustic Peaks
10	36.42745182	0.00038	FB_0026051_L10_PA	Num Acoustic Peaks
14	22.32309137	0.000257	RB_21013914_L14_PA	Num Acoustic Peaks
14	22.32309137	0.000277	FB_0240847_L14_PA	Num Acoustic Peaks
14	22.32309137	0.000257	FB_0238390_L14_PA	Num Acoustic Peaks
14	22.32309137	0.000257	FB_0240837_L14_PA	Num Acoustic Peaks
14	22.32309137	0.000257	RB_20380789_L14_41_1	Num Acoustic Peaks
15	69.016	0.000484	FB_0409141_L17_PA	Num Acoustic Peaks
15	70.354	0.000432	FB_0303543_L15_PA	Num Acoustic Peaks
15	70.354	0.00036	FB_0842164_L11_29_1	Num Acoustic Peaks
15	70.354	0.000432	FB_0116099_L12_PA	Num Acoustic Peaks
15	84.871	0.000119	FB_1071609_L15_84_2	Num Acoustic Peaks
